# Visible‐Light‐Mediated Heterocycle Functionalization via Geometrically Interrupted [2+2] Cycloaddition

**DOI:** 10.1002/anie.202009704

**Published:** 2020-10-08

**Authors:** Mihai V. Popescu, Aroonroj Mekereeya, Juan V. Alegre‐Requena, Robert S. Paton, Martin D. Smith

**Affiliations:** ^1^ Chemistry Research Laboratory University of Oxford 12 Mansfield Road Oxford OX1 3TA UK; ^2^ Department of Chemistry Colorado State University 1301 Center Ave Ft. Collins CO 80523-1872 USA

**Keywords:** density functional calculations, energy transfer, homogeneous catalysis, photochemistry, visible light

## Abstract

The [2+2] photocycloaddition is the most valuable and intensively investigated photochemical process. Here we demonstrate that irradiation of N‐acryloyl heterocycles with blue LED light (440 nm) in the presence of an Ir^III^ complex leads to efficient and high yielding fused γ‐lactam formation across a range of substituted heterocycles. Quantum calculations show that the reaction proceeds via cyclization in the triplet excited state to yield a 1,4‐diradical; intersystem crossing leads preferentially to the closed shell singlet zwitterion. This is geometrically restricted from undergoing recombination to yield a cyclobutane by the planarity of the amide substituent. A prototropic shift leads to the observed bicyclic products in what can be viewed as an interrupted [2+2] cycloaddition.

## Graphical abstract

Irradiation of N‐acryloyl heterocycles with blue LED light and an Ir^III^ complex leads to fused γ‐lactam formation. The reaction proceeds via cyclization in the triplet excited state to yield a 1,4‐diradical; intersystem crossing leads to a singlet zwitterion that is geometrically restricted from yielding a cyclobutane. Proton transfer leads to the products in an interrupted [2+2] cycloaddition.

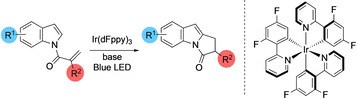

The [2+2] photocycloaddition is arguably the most important photochemical transformation in synthetic chemistry.^[1]^ In recent years, a number of visible‐light [2+2] processes that operate by electron or energy transfer have been disclosed. Elegant approaches by the groups of Bach and Yoon have enabled efficient construction of polycyclic cyclobutane architectures and have also led to advances in the enantioselective catalysis of such reactions.^[2]^ Within this field, the visible‐light‐enabled [2+2] photocycloaddition of indole substrates has received significant recent attention. You demonstrated that indoles bearing a C‐3 tethered alkene could undergo efficient and diastereoselective cycloaddition in the presence of an iridium sensitizer to yield fused cyclobutanes (Scheme 1).^[3]^ In a related transformation disclosed by Oderinde,^[4]^ alkenes tethered through an indole C‐2 amide were demonstrated to be viable substrates for the [2+2] cycloaddition under similar visible‐light conditions. We reasoned that distinct reactivity could be attained through *N*‐functionalization of heterocycles (such as indole) with an α‐aryl acryloyl derivative.^[5]^ This would provide an alkene with a triplet energy of about 50 kcal mol^−1^, similar in magnitude to the triplet energies of a number of excited state photocatalysts. We envisaged that excitation to the triplet (T_1_) state via triplet energy transfer (TET) from an appropriate sensitizer and addition to the indole alkene would generate a 1,4‐diradical intermediate, which in a conventional [2+2] process would generate cyclobutane products via intersystem crossing (ISC) to the singlet state. Once in the singlet state, this intermediate may adopt an open shell diradical or zwitterionic closed shell electronic configuration. Both types of electronic configuration have been recognised in thermal and photochemical cyclobutane formation and in other processes.^[6]^ However, the geometry of the 5,5‐bicyclic lactam ring system means that this route is challenging and hence can be interrupted via proton transfer to afford the functionalized aromatic heterocycle.^[7]^ Owing to the electron‐withdrawing nature of the amide group, we envisioned that this reaction intermediate could possess significant zwitterionic character, further facilitating the proposed proton transfer event.

**Scheme 1:**
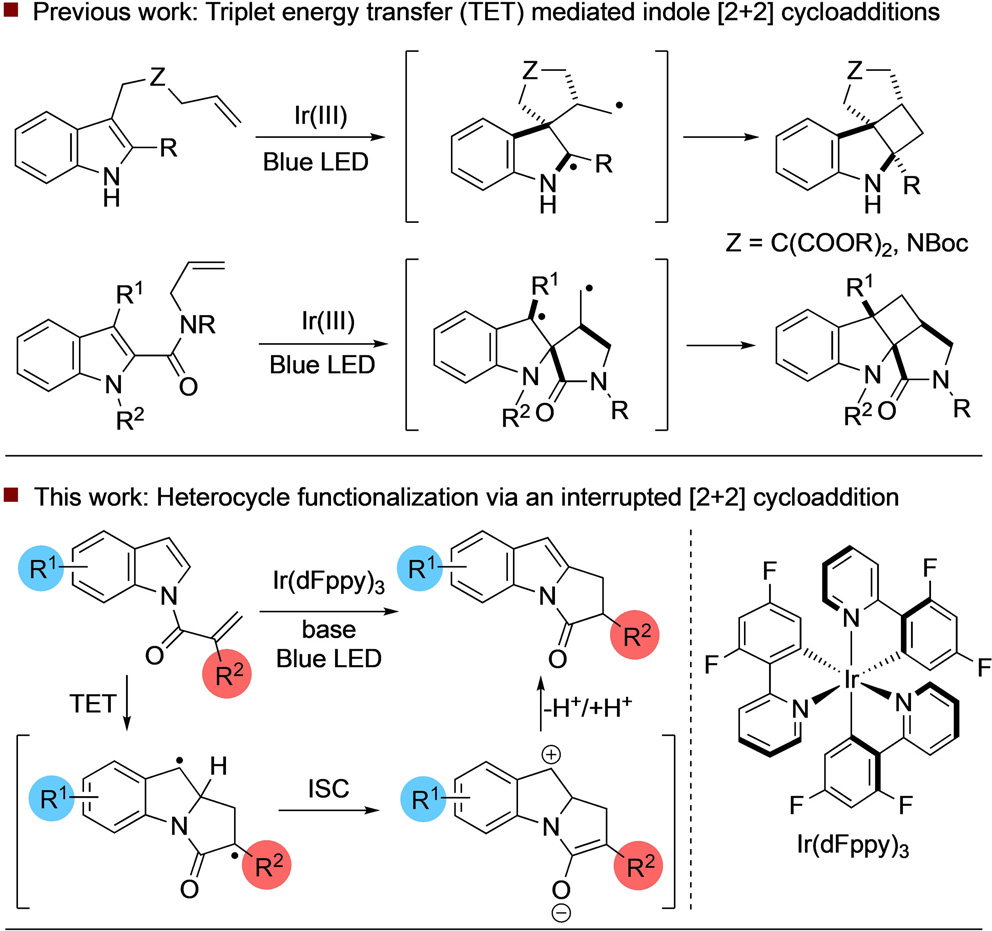
Previous work and approach to visible‐light‐mediated heterocycle functionalization.

The resulting pyrrolo[1,2‐a]indole scaffold is an important motif found in numerous natural products.^[8]^ Although photochemically mediated electron‐transfer processes have been used in the past to enable generation of these structures, there are, to our knowledge, no examples employing energy transfer.^[9]^ To probe the feasibility of this approach, we prepared a test acryloyl substrate **1** through *N*‐acylation of 5‐cyano indole and examined its reactivity in the presence of a range of photocatalysts under irradiation with blue LED light (Table 1). A ruthenium(II) catalyst proved to be ineffective (Table 1, entry 1), and hence we examined iridium photocatalysts with increasingly higher triplet energies (entries 2–6). [Ir(dtbbpy)(ppy)_2_]PF_6_ (entry 3) afforded a trace of product **2**. Significantly higher conversion was observed with *fac*‐Ir(ppy)_3_ (71 % conversion, entry 4) and similar results were obtained using [Ir(dF(CF_3_)ppy)_2_(dtbbpy)]PF_6_ (81 % yield), while Ir(dFppy)_3_ gave complete conversion and an 85 % yield of **2**. Furthermore, addition of a catalytic amount of NaOAc further increased the isolated yield to 93 %. With a working substrate synthesis and cyclization procedure in hand, we examined the scope and limitations of this visible light mediated process (Table 2).

**Table 1:** Optimization: photocyclization of 2‐acryloyl indole derivative.^[a]^ 
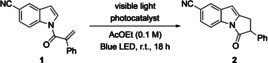

Entry^[b]^	Catalyst	^1^H NMR yield [%]^[b]^
1	none	0
2	Ru(bipy)_3_(PF_6_)_2_	0
3	[Ir(dtbbpy)(ppy)_2_]PF_6_	6
4	*fac*‐Ir(ppy)_3_	71
5	[Ir(dF(CF_3_)ppy)_2_(dtbbpy)]PF_6_	81
6	Ir(dFppy)_3_	92 (85)^[d]^
7^[c]^	Ir(dFppy)_3_	95 (93)^[d]^

[a] Reaction conditions: **1** (0.1 mmol), catalyst (1 mol.%), 35 W blue LED (440 nm), AcOEt ([**1**]=0.04 mol dm^−3^), r.t., 18 h. [b] ^1^H NMR yield measured vs. CH_2_Br_2_ as internal standard. [c] AcOEt ([**1**]=0.1 mol dm^−3^), addition of NaOAc (10 mol %). [d] Yields in parentheses are for isolated material. bipy=2,2′‐bipyridine; dtbbpy=4,4′‐di‐tert‐butyl‐2,2′‐bipyridine; ppy=2‐phenylpyridine; dF(CF_3_)ppy=2‐(2,4‐difluorophenyl)‐5‐(trifluoromethyl)pyridine.

**Table 2:** Scope of triplet‐energy‐transfer‐mediated heterocycle functionalization.^[a]^

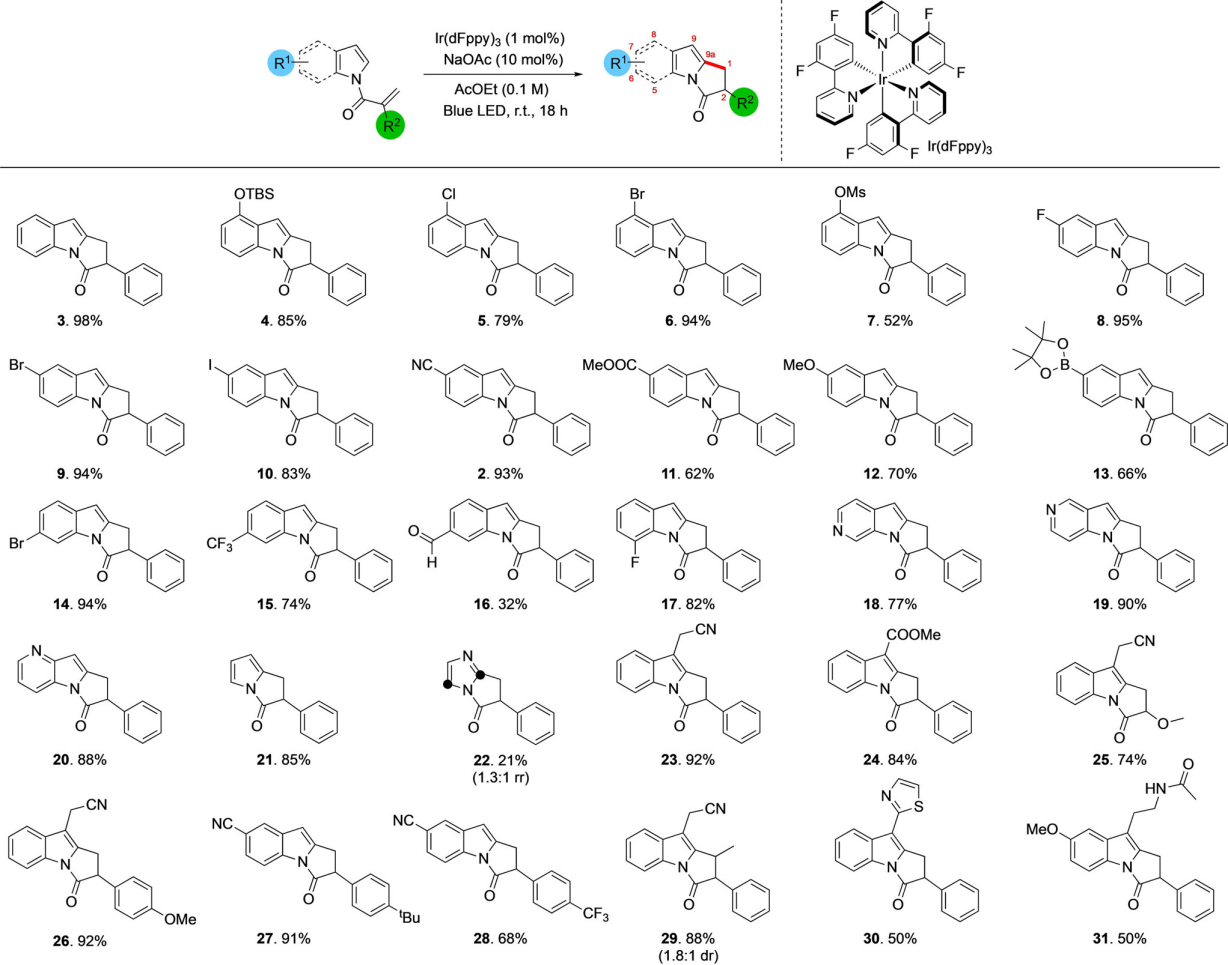

[a] Reaction conditions: 0.3 mmol indole, 0.01 equiv Ir(dFppy)_3_, 0.1 equiv NaOAc, 32 W blue LED, AcOEt (0.05 M); reaction time 18 h. Yields are for isolated material. Positions around the indole core are indicated in red numerals. d.r. (diasteroisomeric ratio) determined by ^1^H NMR spectroscopy. TBS=*tert*‐butyldimethylsilyl; Ms=methanesulfonyl.

We initially explored cyclization of substrates with different substituents on the indole. The parent substrate in which indole is *N*‐functionalized with 2‐phenyl acrylic acid cyclized smoothly to afford bicyclic lactam **3** in 98 % isolated yield. The reaction is remarkably tolerant of different substituents around the pyrroloindole core. Substrates with 8‐substituents cyclize successfully including electron‐donating groups such as *O*‐*tert*‐butyldimethylsilyl (**4**, 85 % yield) and electron‐withdrawing groups such as halogens (8‐chloro **5**, 79 % yield; 8‐bromo **6**, 94 % yield). More reactive groups such as 8‐*O*‐methanesulfonyl **7** also cyclize successfully, albeit in lower yield (52 %). Introducing substituents in the 7‐position does not have any deleterious effects on the reaction. Halogen substituted indoles cyclize in good to excellent yields: 7‐fluoro **8** (95 % yield), 7‐bromo **9** (94 % yield) and 7‐iodo **10** (83 % yield) groups are all well‐tolerated. Electron‐withdrawing groups such as a cyano **2** (93 % yield) or a methyl ester **11** (62 % yield) were both tolerated at C‐7 without incident, and electron rich pyrroloindoles were synthesized with equal facility (**12**, 70 % yield). Pyrroloindoles bearing pinacol boronic acid esters were also demonstrated to be viable substrates for the reaction, cyclizing in 66 % isolated yield to afford **13**. 6‐Bromo and 6‐trifluoromethyl substituted indole derivatives also cyclized effectively to afford product in good yields (**14**, 94 % yield and **15**, 74 % yield, respectively). We were also able to demonstrate that performing the reaction on a substrate bearing an aldehyde in the 6‐position was also possible, albeit affording product **16** in a moderate yield of 32 %; in this case the remaining material is likely consumed via polymerization of the starting acrylic acid derivative. 5‐Substitution on the pyrroloindole core was exemplified through the synthesis of the 5‐fluoro derivative **17** in 82 % yield. We were also able to perform the reaction efficiently on a series of azaindoles: 6‐azaindole derivative **18** (77 % yield), 5‐azaindole **19** (90 % yield) and 4‐azaindole **20** were all generated in good to excellent yields. The transformation is also tolerant of other changes to the backbone of the heterocycle; bicyclic pyrrole derivatives such as **21** could also be generated in 83 % yield while bicyclic imidazole derivative **22** was obtained in 21 % yield as a mixture of regioisomers. Substitution is also tolerated in the 9‐position of the pyrroloindole as demonstrated by the successful conversion of substrates bearing cyanomethyl **23** (92 % yield) and methyl ester groups (**24**, 84 % yield). We also probed the potential to make changes to the substrate via modification of the *N*‐acyl portion of the molecule. Replacement of the α‐phenyl group on the *N*‐acyl moiety by a methoxy ether was well tolerated and led to the desired photo‐cyclised product in high yield (**25**, 74 %), while modification of the phenyl group itself by the addition of a *p*‐OMe substituent also gave an excellent yield (**26**, 92 %). Similarly, addition of a *p*‐^t^Bu (**27**, 91 %) and *p*‐CF_3_ (**28**, 68 %) are well tolerated. Addition of an alkyl substituent at the terminal end of the alkene had no deleterious effect; however, the product was obtained with minimal control over diastereoselectivity (**29**, 88 %, 1.2:1 dr). Finally, we turned our attention to modifying more complex products containing indoles. Camalexin, a simple alkaloid that is cytotoxic against aggressive prostate cancer,^[10]^ and melatonin, an important sleep‐regulating hormone,^[11]^ were acylated and found to be compatible with the photochemical transformation, leading to the formation of their corresponding pyrrolo[1,2‐a]indole containing products **30** (50 % yield) and **31** (50 % yield) respectively.

To probe the practical utility of this transformation, a gram scale synthesis of 6‐bromo substrate **14** was performed. By starting with 4.6 mmol of the appropriate precursor, 1.39 g of **14** were obtained (93 %), thus demonstrating that the reaction is scalable without affecting the overall yield.

To probe the mechanistic profile of this transformation, we carried out computational studies with density functional theory (DFT,^[12]^ M06‐2X‐D3/Def2‐QZVPP//M06‐2X‐D3/6‐31++G(d,p);[^13^, ^14^, ^15^] in SMD ethyl acetate)^[16]^ in conjunction with experimental techniques (Figure 1). The calculated energy required to promote *N*‐acyl ^**1**^ **A** from the singlet (S_0_) to π‐π* triplet (T_1_) state is 52.2 kcal mol^−1^. This is well matched with the emissive energy of the Ir(dFppy)_3_ (60.1 kcal mol^−1^),^[17]^ [Ir(dF(CF_3_)ppy)_2_(dtbbpy)]PF_6_ (61.8 kcal mol^−1^)^[17]^ and *fac*‐Ir(ppy)_3_ (55.2 kcal mol^−1^)^[17]^ catalysts. To exclude an alternative photoredox catalytic cycle, we turned our attention to electrochemical studies. Square voltammetry performed on substrate **1** indicated a reduction potential of −1.62 V and an oxidation potential of 1.91 V (vs. SCE in MeCN). Both reduction and oxidation potentials are outside the redox capabilities of the optimal Ir(dFppy)_3_ catalyst (*E* ^1/2^(M^+^/M*)=−1.27 V; *E* ^1/2^(M*/M^−^)=0.35 V vs. SCE in MeCN).^[17]^ Furthermore, Stern–Volmer quenching experiments showed a clear substrate catalyst interaction, thus making energy transfer the most probable mechanistic candidate.

**Figure 1:**
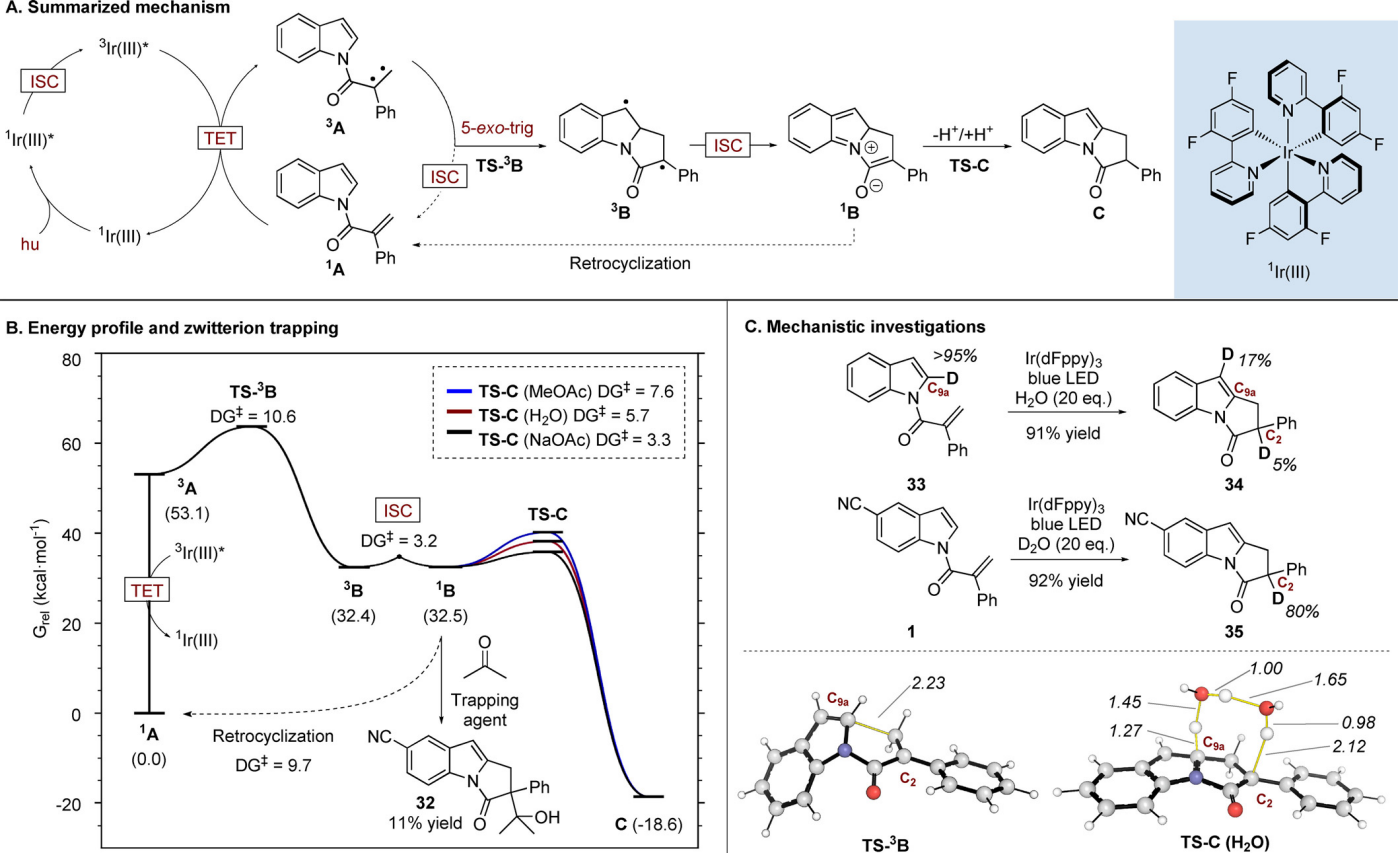
A) Summary of the proposed reaction mechanism. B) Reaction energy profile of the main pathway and retro‐cyclization using Boltzmann weighted G values in kcal mol^−1^, along with the trapping experiment carried out with acetone (conditions: Ir(dFppy)_3_ (1 mol %), corresponding indole (0.1 M in AcOEt), blue LEDs, r.t., 16 h., acetone (0.4 M)). C) Top: Deuteration experiments (conditions: Ir(dFppy)_3_ (1 mol %), corresponding indole (0.1 M in AcOEt), blue LEDs, r.t., 16 h). Bottom: calculated transition structures of the carbon–carbon bond‐forming cyclization, and the water‐assisted H shuttle mechanism (distances shown in Å). Key bonds involved in TSs are highlighted in yellow.

Our proposed mechanism is pictured in Figure 1. After singlet‐to‐triplet excitation of the initial substrate by the excited state catalyst (^**1**^ **A** to ^**3**^ **A**), a 5‐*exo*‐trig cyclization takes place to produce intermediate ^**3**^ **B**. ^**3**^ **B** undergoes an intersystem crossing (ISC) and generates the corresponding singlet species ^**1**^ **B**; this species is better described as a closed‐shell singlet zwitterion rather than a singlet diradical (⟨Ŝ^2^⟩=0 in the wavefunction stability checks). This zwitterionic species undergoes a proton transfer to form final product **C**. The reaction activation barriers of the main pathway, which range from 3.2 to 10.6 kcal mol^−1^ (Figure 1 B) are easily accessible at room temperature. The barrier to ISC is particularly low owing to the small structural reorganization needed from ^**3**^ **B** to reach the geometry observed in the ISC (Supporting Information, Figure S7). The calculated energy profile also indicates the presence of a retrocyclization process (from ^**1**^ **B** back to ^**1**^ **A**) with an activation barrier of 9.7 kcal mol^−1^; this is somewhat higher than the barrier required to form the reaction product **C** (3.3 to 7.6 kcal mol^−1^). When we performed the reaction on substrate **1** in the presence of acetone, the adduct **32** was isolated (11 % yield), along with the pyrroloindole product, consistent with the presence of an enolate‐like intermediate. The last step of the proposed mechanism involves transformation of the zwitterion ^**1**^ **B** into the final product **C**. We reasoned initially that this could occur via stepwise deprotonation/reprotonation events or by a direct hydrogen shift from C_9a_ of ^**1**^ **B** to C_2_. To probe this, we subjected deuterated substrate **33** to the reaction conditions in the presence of 20 equivalents of H_2_O (Figure 1 C). Under these conditions only a small amount of deuterium in the C_2_ position was observed in **34**. This result implies that the H atom from C_9a_ is not directly transferred to the C_2_ position, which agrees with the prohibitively high calculated barrier of the intramolecular H‐shift (31.7 kcal mol^−1^; Supporting Information, Figure S6 A). We did, however, observe 17 % incorporation of deuterium at C_9_, which is consistent with a minor 1,2‐shift pathway; this was also observed computationally (8.9 kcal mol^−1^; Supporting Information, Figure S6 B). Conversely, when using D_2_O as co‐solvent with a non‐deuterated indole **1**, the C_2_ position of product **35** showed significant deuterium incorporation (80 %); control experiments also demonstrated that the pyrroloindole **2** is unreactive under these conditions and no deuterium incorporation is observed. This is consistent with a pathway whereby the C_2_ hydrogen comes from solvent. We probed this with further calculation and found that the stepwise solvent mediated deprotonation/reprotonation pathway is feasible with a barrier of 7.6 kcal mol^−1^ (Supporting Information, Figure S6 C). We also discovered an alternative lower energy pathway that could operate in the presence of water (5.7 kcal mol^−1^, Figure 1 C). In this scenario, the water co‐solvent acts to shuttle a proton from C_9a_ to C_2_ leading to the product. However, when a base such as AcONa is used or the concentration of water as a cosolvent is low, other pathways might become the preferred routes to promote this 1,3‐shift (Supporting Information, Figure S6 C,D).

In conclusion, we have discovered an operationally simple and functional‐group‐tolerant synthesis of pyrroloindoles that proceeds in the presence of visible light and an iridium(III) sensitizer. The reaction proceeds via cyclization in the triplet excited state to yield a 1,4‐diradical; intersystem crossing leads preferentially to a closed shell singlet zwitterion that is geometrically restricted from undergoing recombination to yield a cyclobutane, leading to the preferential formation of a 5,5‐bicyclic lactam ring system.

## Conflict of interest

The authors declare no conflict of interest.

## Supporting information

As a service to our authors and readers, this journal provides supporting information supplied by the authors. Such materials are peer reviewed and may be re‐organized for online delivery, but are not copy‐edited or typeset. Technical support issues arising from supporting information (other than missing files) should be addressed to the authors.

Supplementary
