# Supplementary material for: Visible‐Light‐Mediated Heterocycle Functionalization via Geometrically Interrupted [2+2] Cycloaddition
Source: Angew Chem Int Ed Engl. 2020 Oct 8;59(51):23020–4. doi: 10.1002/anie.202009704 (PMC7891567; doi:10.1002/anie.202009704)
Supplement: Supplementary file 1 — Supplementary [file ANIE-59-23020-s001.pdf]

Angewandte Chemie
Eine Zeitschrift der Gesellschaft Deutscher Chemiker

Supporting Information

Visible-Light-Mediated Heterocycle Functionalization via Geometrically Interrupted [2+2] Cycloaddition

Mihai V. Popescu, Aroonroj Mekereeya, Juan V. Alegre-Requena, Robert S. Paton, and Martin D. Smith**

anie_202009704_sm_miscellaneous_information.pdf

Contents

1. General Information 2
2. General Procedures 4
3. Reaction Optimisation 5
4. Unsuccessful transformations.. 5
5. Characterisation of compounds.. 6
6. Deuterium Labelling and Trapping experiments 35
7. Large Scale Synthesis 38
8. Stern-Volmer Quenching 39
9. Square voltammetry 41
10. NMR Spectra 42
11. Computational Studies.. 119

1. General Information

All reagents and solvents were used as supplied commercially without further purification unless stated otherwise. Dry dichloromethane, tetrahydrofuran and diethyl ether were purified through activated alumina columns employing the method of Grubbs *et al*. Water was purified using an Elix® UV-10 system. HPLC grade solvents for photochemical reactions were degassed by sparging the solvent with argon or nitrogen gas for at least 30 minutes. Petrol ether (PE) 40–60 refers to the fraction of petroleum ether which boils in the range 40–60 °C. Tris[2-(4,6-difluorophenyl)pyridinato-C^2,*N*]iridium (III) ($Ir(Fppy)_3$) was purchased from Sigma-Aldrich and used as received.

Photochemical reactions were performed using a Kessil PR440 blue LED lamp. Optimization reactions were performed in sealed microwave vials, whilst larger scale reactions were performed in sealed Schlenk tubes. In both cases the LED lamp was placed approximately 1 cm away from the edge of the reaction vessel. Cooling was provided by a stream of nitrogen, which kept the reaction at ambient temperature (approximately 25 °C).

Thin Layer Chromatography (TLC) was performed using pre-coated aluminium-backed Merck TLC Silica Gel 60 F_{254} plates and visualised using UV irradiation (λ = 254 nm) or staining with potassium permanganate solution. Flash column chromatography was performed with Merck Geduran® Si 60 Silicagel (40–63 µm particle size) or with Sigma-Aldrich Silica gel (technical grade, pore size 60 Å, 40–63 µm particle size). All solvents used for chromatographic purification were HPLC grade or equivalent and supplied by Sigma-Aldrich.

All Nuclear Magnetic Resonance (NMR) spectra were recorded on Bruker AVIIIHD 400 nanobay spectrometers at room temperature in solutions of $CDCl_3$ unless otherwise stated and the deuterated solvent acted as the deuterium lock. ^{1}H NMR spectra were recorded at 400 MHz, ^{13}C NMR spectra at 101 MHz with broadband proton decoupling and ^{19}F NMR spectra at 377 MHz. Residual protic solvent signal acted as an internal reference for ^{1}H NMR and the deuterated solvent carbon signal acted as an internal reference for ^{13}C NMR ($CDCl_3$: ^{1}H NMR = 7.26 ppm, ^{13}C NMR = 77.16 ppm). ^{19}F NMR spectra were reference externally to $CFCl_3$. Chemical shifts, δ, are given in parts per million (ppm) to the nearest 0.01 ppm for ^{1}H and ^{19}F and 0.1 ppm for ^{13}C NMR. The multiplicity of a signal is reported as such: s–singlet, d–doublet, t–triplet, q–quartet, quint–quintet, m–multiplet, br–broad, or combinations thereof. Coupling constants, *J*, are reported as observed in Hz. For inseparable mixtures of diastereomers, only peaks corresponding to the major diastereomer are reported. Additional 2D NMR experiments (COSY, HSQC, HMBC and NOESY) were used to assist in structural assignment in conjunction with the carbon shift predicting algorithm CASCADE[1].

Fourier-transform infrared (FT-IR) spectra were recorded from evaporated films on a Bruker Tensor 27 spectrometer equipped with a Pike Miracle Attenuated Total Reflectance (ATR) sampling accessory. Absorption maxima are quoted in wavenumbers, ν_{max}/cm^{-1}, for the range 3500–600 cm^{-1}.

High resolution mass spectrometry (HRMS) using electrospray ionization (ESI) was carried out on a Thermo Exactive orbitrap spectrometer equipped with a Waters Equity LC system. HRMS using chemical ionization (CI) or electron ionization (EI) was carried out on a Waters GCT system equipped with a Time of Flight

1. Y. Guan, R. S. Paton, CASCADE, doi:10.3281/zenodo.3369360 (http://nova.chem.colostate.edu/cascade)

(TOF) spectrometer. Low resolution mass spectrometry was carried out using ESI and was performed on a Micromass LCT Premier Spectrometer. In both cases the mass reported is that containing the most abundant isotopes.

Melting points were determined using a Reichert melting point apparatus or a Leica VMTG heated-stage microscope equipped with a Testo 720 thermometer and are reported uncorrected. All compounds were crystallised from chloroform ($CHCl_3$) unless stated otherwise.

Systematic names were generated using ChemDraw 16.0 in accordance with the guidelines specified by the International Union of Pure and Applied Chemistry (IUPAC).

2. General Procedures

Phase-transfer method [**A1**]

To a solution of carboxylic acid (1.0 equiv.) and oxalyl chloride (1.2 equiv.) in anhydrous CH_2Cl_2 (2 mL), was added one drop of anhydrous DMF. The reaction mixture was stirred at room temperature for 3 h. Once the reaction completed, anhydrous toluene (4 mL), tetrabutylammonium bromide (0.34 equiv.), freshly ground KOH (2.4 equiv.) and indole or *N*-heterocycle (1.0 equiv.) were added to the reaction mixture. The reaction was further stirred at room temperature until completion (indicated by TLC). Water (15 mL) was then added to the reaction mixture. The aqueous layer was extracted with EtOAc (3 × 10 mL). The combined organic layer was washed with brine (5 mL), dried over $MgSO_4$, and concentrated *in vacuo*.

Amidation via NEt_3/DMAP/oxalyl chloride [**A2**]

To a solution of carboxylic acid (1.0 equiv.) and oxalyl chloride (1.2 equiv.) in anhydrous CH_2Cl_2 (2.4 mL), was added one drop of anhydrous DMF. The reaction mixture was stirred at room temperature for 3 h. Once the reaction completed, anhydrous NEt_3 (1.67 equiv.) was added dropwise via syringe under a strong flow of N_2 (in some cases, an ice bath was required to prevent a thermal runaway), followed by an addition of DMAP (0.17 equiv.). After approx. 1 min, an indole or *N*-heterocycle (0.83 equiv.) was added, and the reaction mixture was then stirred overnight and monitored by TLC. After completion, water (20 mL) was added, and the aqueous layer was extracted with EtOAc (3 × 15 mL). The combined organic layer was washed with brine (10 mL), dried over $MgSO_4$, and concentrated *in vacuo*.

Amidation via DCC coupling [**A3**]

Carboxylic acid (1.2 equiv.), indole (1.0 equiv.), DMAP (0.12 equiv.) and DCC (1.2 equiv.) were dissolved in anhydrous CH_2Cl_2 (11.3 mL). The reaction was kept stirring at room temperature overnight to reach completion. The reaction mixture was then filter through a plug of silica. The filtrate was concentrated *in vacuo*, the crude residue was then purified by flash column chromatography.

Photocyclisation *via* triplet energy transfer [**B1**]

Amide substrate (1.0 equiv.), $Ir(dFppy)_3$ (0.01 equiv.) and NaOAc (0.1 equiv.) were transferred to a glass vial. The vial was sealed and flushed with argon for 5 min. The vial was then charged with degassed EtOAc (10 mL/mmol amide). The reaction mixture was then irradiated with blue light and stirred while maintaining at room temperature by a stream of N_2. Once the reaction was completed, the excess solvent was evaporated.

3. Reaction Optimisation

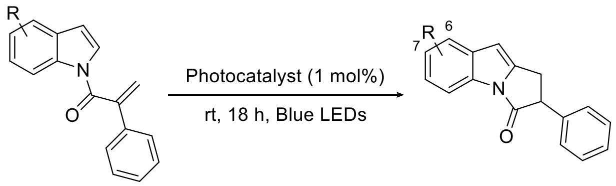

Entry	R	Catalyst	Solvent	Base	Conversion	Yield[a]
1	7-CN	N/A	AcOEt (0.04 M)	N/A	N/A	0%
2	7-CN	$Ru(bipy)_3(PF_6)_2$	AcOEt (0.04 M)	N/A	4%	0%
3	7-CN	$[Ir(ppy)_2(dtbbpy)]PF_6$	AcOEt (0.04 M)	N/A	50%	6%
4	7-CN	$Ir(ppy)_3$	AcOEt (0.04 M)	N/A	100%	71%
5	7-CN	$[Ir(dF(CF_3)ppy)_2(dtbbpy)]PF_6$	AcOEt (0.04 M)	N/A	100%	81%
6	7-CN	$Ir(dFppy)_3$	AcOEt (0.04 M)	N/A	100%	92% (85%)[b]
8	7-CN	$Ir(dFppy)_3$	MeCN (0.04 M)	N/A	100%	74%
9	7-CN	$Ir(dFppy)_3$	PhMe (0.04 M)	N/A	100%	50%
10	7-CN	$Ir(dFppy)_3$	Acetone (0.04 M)	N/A	100%	64%
11	6-Cl	$Ir(dFppy)_3$	AcOEt (0.04 M)	N/A	100%	55% (55%)[b]
12	6-Cl	$Ir(dFppy)_3$	AcOEt (0.04 M)	KOAc (1.0 eq)	100%	74%
13	6-Cl	$Ir(dFppy)_3$	AcOEt (0.04 M)	NaOAc (1.0 eq)	100%	83%
14	6-Cl	$Ir(dFppy)_3$	AcOEt (0.04 M)	K_3PO_4 (1.0 eq)	100%	32%
15	6-Cl	$Ir(dFppy)_3$	AcOEt (0.04 M)	NaOAc (0.5 eq)	100%	82%
16	6-Cl	$Ir(dFppy)_3$	AcOEt (0.04 M)	NaOAc (0.1 eq)	100%	82%
17	6-Cl	$Ir(dFppy)_3$	AcOEt (0.025 M)	NaOAc (0.1 eq)	100%	75%
18	6-Cl	$Ir(dFppy)_3$	AcOEt (0.08 M)	NaOAc (0.1 eq)	100%	79%
19	6-Cl	$Ir(dFppy)_3$	AcOEt (0.1 M)	NaOAc (0.1 eq)	100%	81% (79%)[b]
20	7-CN	$Ir(dFppy)_3$	AcOEt (0.1 M)	NaOAc (0.1 eq)	100%	95% (93%)[b]

Table S1: *Reaction conditions:* ***1*** *(0.1 mmol), catalyst (1 mol.%), 35W blue LED (440 nm), r.t., 18 h; [a] ^1H NMR yield measured vs CH_2Br_2 as internal standard; [b] yields in parentheses are for isolated material.*

4. Unsuccessful transformations

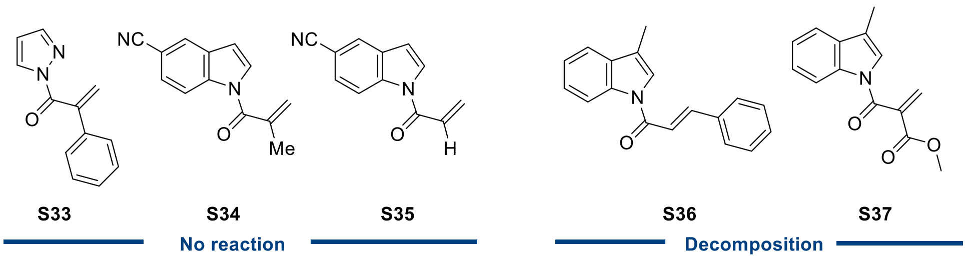

Scheme S1*: Substrates synthesis that proved unsuccessful under the optimised reaction conditions*

Compounds **S36** and **S37** were prepared according to their corresponding literature procedures.[2]

2. D. V. Patil, M. A. Cavitt, S. France, *Org. Lett.*, **2011**, *13*, 5820-5823.

5. Characterisation of compounds

1-(2-phenylacryloyl)-1H-indole-5-carbonitrile (**1**)

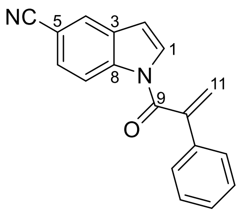

Synthesised from atropic acid (222 mg, 1.5 mmol) and indole (142 mg, 1.0 mmol) using a modified **General Procedure A1** where toluene was replaced by CH_2Cl_2. The resulting crude material was purified by column chromatography (10% AcOEt/pentane) to give the titled compound as a white solid (155 mg, 0.57 mmol, 57%); **m.p.** 144 °C; δ_H (400 MHz, $CDCl_3$): 8.64 (1H, dt, *J* = 8.6, 0.8 Hz, H7), 7.90 (1H, dd, *J* = 1.6, 0.7 Hz, H4), 7.65 (1H, dd, *J* = 8.6, 1.6 Hz, H6), 7.49 – 7.35 (6H, m, H1, Ph*H*), 6.59 (1H, dd, *J* = 3.8, 0.8 Hz, H2), 6.13 (1H, s, H11), 5.76 (1H, s, H11); δ_C (100 MHz, $CDCl_3$): 168.62, 143.99, 137.51, 135.04, 131.03, 129.51, 129.33, 129.30, 129.14, 128.45, 126.21, 125.77, 119.88, 119.60, 117.57, 108.62, 107.73; **IR** (thin film) ν_{max}/cm^{-1}: 2227, 1684, 1540; **HRMS** *m/z* (ESI$^+$) found $[M+H]^+$ 273.1024; $C_{18}H_{13}N_2O^+$ requires 273.1028.

3-oxo-2-phenyl-2,3-dihydro-1H-pyrrolo[1,2-a]indole-7-carbonitrile (**2**)

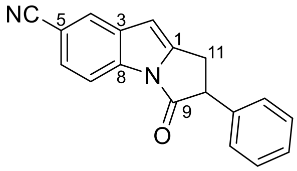

Synthesised from compound 1 (81.7 mg, 0.3 mmol) using **General Procedure B1**. The resulting crude material was purified by column chromatography (15% AcOEt/pentane) to give the tiled compound as a white solid (76.0 mg, 0.28 mmol, 93%); **m.p.** 163 °C; δ_H (400 MHz, $CDCl_3$): 8.09 (1H, dt, *J* = 8.4, 0.8 Hz, H7), 7.81 (1H, dd, *J* = 1.6, 0.7 Hz, H4), 7.48 (1H, dd, *J* = 8.3, 1.5 Hz, H6), 7.37 – 7.12 (5H, m, Ph*H*), 6.36 (1H, td, *J* = 1.6, 0.8 Hz, H2), 4.36 (1H, dd, *J* = 9.3, 4.9 Hz, H10), 3.69 (1H, ddd, *J* = 17.8, 9.2, 1.5 Hz, H11), 3.26 (1H, ddd, *J* = 17.7, 4.9, 1.6 Hz, H11). δ_C (100 MHz, $CDCl_3$): 171.94, 144.08, 137.66, 135.47, 132.49, 129.40, 128.22, 127.78, 126.98, 125.57, 119.72, 114.63, 107.83, 100.52, 52.69, 29.69; **IR** (thin film) ν_{max}/cm^{-1}: 2220, 1733, 1585, 1462, 1383, 1350; **HRMS** *m/z* (ESI$^+$) found $[M+H]^+$ 273.1024; $C_{18}H_{13}N_2O^+$ requires 273.1022.

1-(1H-indol-1-yl)-2-phenylprop-2-en-1-one (**S1**)

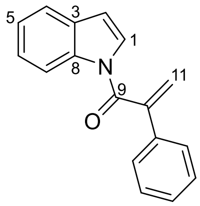

Synthesised from atropic acid (0.5 g, 3.375 mmol) and indole (264 mg, 2.25 mmol) using **General Procedure A1**. The resulting crude material was purified by column chromatography (2.5% Et_2O/pentane) to give the titled compound as a colourless oil (239 mg, 0.97 mmol, 43%); **δ_H** (400 MHz, $CDCl_3$): 8.57 (1H, dq, *J* = 8.2, 0.9 Hz, H7), 7.57 (1H, dt, *J* = 7.6, 1.1 Hz, H4), 7.54 – 7.45 (2H, m, H5, H6), 7.45 – 7.28 (6H, m, H1, Ph*H*), 6.55 (1H, dd, *J* = 3.8, 0.8 Hz, H2), 6.08 (1H, s, H11), 5.70 (1H, s, H11); **δ_C** (100 MHz, $CDCl_3$): 168.66, 144.52, 135.67, 135.46, 131.05, 129.20, 129.16, 127.07, 126.23, 125.27, 124.32, 121.02, 118.60, 116.84, 109.26.; **IR** (thin film) ν_{max}/cm^{-1}: 1689, 1536, 1471, 1379; **HRMS** *m/z* (ESI$^+$) found $[M+H]^+$ 248.1071; $C_{17}H_{14}NO^+$ requires 248.1070.

2-phenyl-1,2-dihydro-3H-pyrrolo[1,2-a]indol-3-one (**3**)

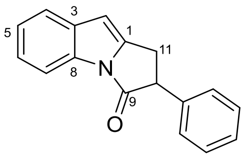

Synthesised from compound **3** (74.2 mg, 0.30 mmol) using **General Procedure B1**. The resulting crude material was purified by flash column chromatography (2.5→5% Et_2O/petroleum ether) to yield the titled compound **3** as an off-white solid (72.6 mg, 0.29 mmol, 98%); **m.p.** 160-162 °C; **δ_H** (400 MHz, $CDCl_3$) 8.07 (1H, m, H7), 7.51 (1H, m, H4), 7.39-7.18 (7H, m, Ph*H*, H5, H6), 6.31 (1H, s, H2), 4.31 (1H, dd, *J* = 9.5, 5.0 Hz, H10), 3.63 (1H, dd, *J* = 17.4, 5.0 Hz, H11), 3.21 (1H, dd, *J* = 17.4, 9.5 Hz, H11); **δ_C** (100 MHz, $CDCl_3$) 29.63, 52.81, 100.71, 113.90, 120.74, 123.52, 124.36, 127.78, 127.82, 129.18, 130.75, 135.52, 138.50, 141.83, 171.89; **IR** (thin film) ν_{max}/cm^{-1} 1729, 1365; **HRMS** *m/z* (ESI$^+$) found $[M+H]^+$ 248.1071; $C_{17}H_{14}NO^+$ requires 248.1075.

1-(4-((*tert*-butyldimethylsilyl)oxy)-1H-indol-1-yl)-2-phenylprop-2-en-1-one (**S2**)

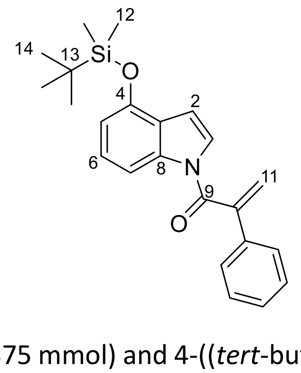

Synthesised from atropic acid (0.5 g, 3.375 mmol) and 4-((*tert*-butyldimethylsilyl)oxy)-1H-indole (556 mg, 2.25 mmol) using **General Procedure A1**. The resulting crude material was purified by column chromatography (3% Et_2O/pentane) to give the titled compound as a colourless oil (249 mg, 0.65 mmol,

29%); **δ$_H$** (400 MHz, $CDCl_3$): 8.17 (1H, dt, *J* = 8.3, 0.8 Hz, H7), 7.51 – 7.45 (2H, m, Ph*H*), 7.41 – 7.33 (3H, m, Ph*H*), 7.25 (1H, t, *J* = 8.3 Hz, H6), 7.20 (1H, d, *J* = 3.8 Hz, H1), 6.74 (1H, dd, *J* = 7.9, 0.8 Hz, H5), 6.60 (1H, dd, *J* = 3.8, 0.8 Hz, H2), 6.07 (1H, s, H11), 5.69 (1H, s, H11), 1.04 (9H, s, H14), 0.24 (6H, s, H12); **δ$_C$** (100 MHz, $CDCl_3$): ^{13}C NMR (101 MHz, $CDCl_3$) δ 168.71, 148.79, 144.62, 137.28, 135.51, 129.17, 129.14, 126.26, 126.12, 125.61, 124.10, 118.55, 113.86, 110.41, 106.67, 25.86, 18.42, -4.17; **IR** (thin film) ν_{max}/cm^{-1}: 1695, 1481, 1419, 1344, 1246; **HRMS** *m/z* (ESI^+) found $[M+H]^+$ 378.1884; $C_{23}H_{28}NO_2Si^+$ requires 378.1884.

8-((*tert*-butyldimethylsilyl)oxy)-2-phenyl-1,2-dihydro-3H-pyrrolo[1,2-a]indol-3-oneone (**4**)

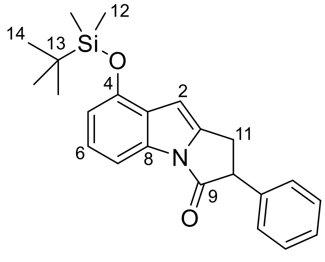

Synthesised from compound **S2** (113.3 mg, 0.3 mmol) using **General Procedure B1**. The resulting crude material was purified by column chromatography (2.5% Et_2O/pentane) to give the tiled compound as a white solid (96.2 mg, 0.26 mmol, 85%); **m.p.** 117-120 °C; **δ$_H$** (400 MHz, $CDCl_3$): 7.72 (1H, dt, *J* = 8.0, 0.8 Hz, H7), 7.40 – 7.25 (5H, m, Ph*H*), 7.15 (1H, t, *J* = 8.0 Hz, H6), 6.74 (1H, dd, *J* = 7.9, 0.8 Hz, H5), 6.41 (1H, td, *J* = 1.6, 0.7 Hz, H2), 4.38 (1H, dd, *J* = 9.2, 4.8 Hz, H10), 3.69 (1H, ddd, *J* = 17.4, 9.2, 1.5 Hz, H11), 3.25 (1H, ddd, *J* = 17.4, 4.8, 1.6 Hz, H11), 1.07 (9H, s, H14), 0.26 (6H, d, *J* = 1.5 Hz, H12); **δ$_C$** (100 MHz, $CDCl_3$): 172.03, 148.41, 140.27, 138.61, 132.35, 129.23, 128.36, 127.87, 124.48, 114.16, 107.69, 98.26, 53.01, 29.68, 25.90, 18.45, -4.13; **IR** (thin film) ν_{max}/cm^{-1}: 1742, 1691, 1591, 1480, 1142; **HRMS** *m/z* (ESI^+) found $[M+H]^+$ 378.1885; $C_{23}H_{28}NO_2Si^+$ requires 378.1884.

1-(4-chloro-1H-indol-1-yl)-2-phenylprop-2-en-1-one (**S3**)

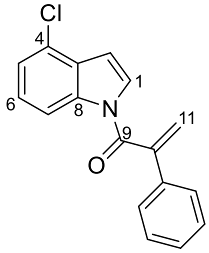

Synthesised from atropic acid (0.5 g, 3.375 mmol) and 4-chloroindole (0.54 mL, 2.25 mmol) using **General Procedure A1**. The resulting crude material was purified by column chromatography (2% Et_2O/pentane) to give the titled compound as a white solid (409 mg, 0.97 mmol, 43%); **m.p.** 39 °C; **δ$_H$** (400 MHz, $CDCl_3$): 8.56 – 8.38 (1H, m, H7), 7.58 – 7.28 (7H, m, H1, H5, H6, Ph*H*), 6.67 (1H, dd, *J* = 3.8, 0.8 Hz, H2), 6.10 (1H, s, H11), 5.72 (1H, s, H11); **δ$_C$** (100 MHz, $CDCl_3$): 168.66, 144.27, 136.36, 135.27, 129.77, 129.35, 129.28, 129.24, 129.22, 127.60, 126.24, 126.22, 126.06, 124.10, 119.21, 115.31, 107.25; **IR** (thin film) ν_{max}/cm^{-1}: 1682, 1494, 1314, 1176; **HRMS** *m/z* (ESI^+) found $[M+H]^+$ 282.0680; $C_{17}H_{13}NClO_1^+$ requires 287.0680.

8-chloro-2-phenyl-1,2-dihydro-3H-pyrrolo[1,2-a]indol-3-one (**5**)

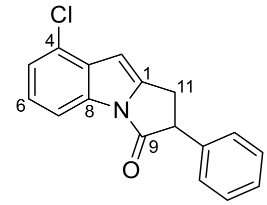

Synthesised from compound **S3** (84.5 mg, 0.3 mmol) using **General Procedure B1**. The resulting crude material was purified by column chromatography (5% AcOEt/pentane) to give the tiled compound as a white solid (66.8 mg, 0.24 mmol, 79%); **m.p.** 85-86 °C; δ_H (400 MHz, $CDCl_3$): 7.99 (1H, dt, *J* = 7.9, 0.9 Hz, H7), 7.43 – 7.18 (7H, m, H5, H6, Ph*H*), 6.51 (1H, q, *J* = 1.5 Hz, H2), 4.40 (1H, dd, *J* = 9.2, 4.7 Hz, H10), 3.73 (1H, ddd, *J* = 17.6, 9.2, 1.5 Hz, H11), 3.30 (1H, ddd, *J* = 17.6, 4.8, 1.6 Hz, H11); δ_C (100 MHz, $CDCl_3$): 172.01, 142.54, 138.14, 134.10, 131.44, 129.33, 128.06, 127.80, 125.65, 124.42, 124.25, 112.42, 99.14, 52.82, 29.73; **IR** (thin film) ν_{max}/cm^{-1}: 1718, 1608, 1585 1424, 1136; **HRMS** *m/z* (ESI^+) found $[M+H]^+$ 282.0680; $C_{17}H_{13}ClNO_2^+$ requires 282.0681.

1-(4-bromo-1H-indol-1-yl)-2-phenylprop-2-en-1-one (**S4**)

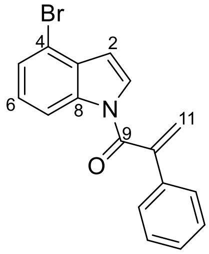

Synthesised from atropic acid (0.5 g, 3.375 mmol) and 4-bromoindole (442 mg, 2.25 mmol) using **General Procedure A1**. The resulting crude material was purified by column chromatography (3% Et_2O/pentane) to give the titled compound as a white solid (535 mg, 1.64 mmol, 73%); **m.p.** 33-34 °C; δ_H (400 MHz, $CDCl_3$): 8.51 (1H, dd, *J* = 8.4, 1.1 Hz, H7), 7.51 – 7.42 (3H, m, H5, Ph*H*), 7.42 – 7.33 (4H, m, H1, Ph*H*), 7.26 (1H, t, *J* = 8.0 Hz, H6), 6.62 (1H, dd, *J* = 3.8, 0.7 Hz, H2), 6.10 (1H, s, H11), 5.72 (1H, s, H11); δ_C (100 MHz, $CDCl_3$): 168.70, 144.26, 136.04, 135.27, 131.68, 129.36, 129.25, 127.62, 127.22, 126.32, 126.22, 119.24, 115.84, 114.76, 108.97; **IR** (thin film) ν_{max}/cm^{-1}: 1682, 1532, 1419, 1345, 1174; **HRMS** *m/z* (ESI^+) found $[M+H]^+$ 326.0175; $C_{17}H_{13}NBrO^+$ requires 326.0175.

8-bromo-2-phenyl-1,2-dihydro-3H-pyrrolo[1,2-a]indol-3-one (**6**)

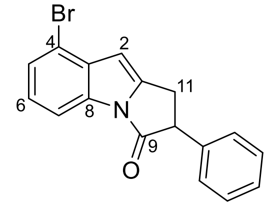

Synthesised from compound **S4** (97.9 mg, 0.3 mmol) using **General Procedure B1**. The resulting crude material was purified by column chromatography (5% AcOEt/pentane) to give the tiled compound as a white solid (92.2 mg, 0.28 mmol, 94%); **m.p.** 101-102 °C; δ_H (400 MHz, $CDCl_3$): 7.96 (1H, dt, *J* = 8.0, 0.9 Hz, H7), 7.38 (1H, dd, *J* = 7.9, 0.8 Hz, H5), 7.34 – 7.17 (5H, m, Ph*H*), 7.08 (1H, t, *J* = 7.9 Hz, H6), 6.39 (1H, td, *J* =

1.6, 0.7 Hz, H2), 4.32 (1H, dd, J = 9.2, 4.7 Hz, H10), 3.65 (1H, ddd, J = 17.6, 9.2, 1.5 Hz, H11), 3.22 (1H, ddd, J = 17.6, 4.7, 1.6 Hz, H11); **δ_C** (100 MHz, $CDCl_3$): 171.74, 142.27, 137.81, 135.72, 130.80, 129.02, 127.75, 127.48, 127.00, 124.37, 113.77, 112.61, 100.52, 52.46, 29.43; **IR** (thin film) ν_{max}/cm^{-1}: 1724, 1421, 1156; **HRMS** *m/z* (ESI^+) found $[M+H]^+$ 326.0176; $C_{17}H_{13}BrNO_2^+$ requires 326.0175.

1-(2-phenylacryloyl)-1H-indol-4-yl methanesulfonate (**S5**)

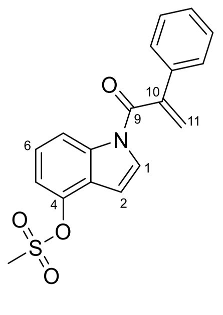

Synthesised from atropic acid (421 mg, 2.84 mmol) and 1H-indol-4-yl methanesulfonate (500 mg, 2.37 mmol) using **General Procedure A2**. The resulting crude residue was purified by column chromatography (15% Et_2O/petroleum ether) to obtain the desired compound as a thick yellow oil (523 mg, 1.53 mmol, 65%); **δ_H** (400 MHz, $CDCl_3$) 7.62 (1H, d, J = 8.0 Hz, H1), 7.49-7.44 (3H, m, H2, Ph*H*), 7.39-7.34 (3H, m, Ph*H*), 7.32 (1H, d, J = 8.0 Hz, H5), 7.19 (1H, t, J = 8.0Hz, H6), 5.99 (1H, s, H11), 5.61 (1H, s, H11), 3.17 (3H, s, C*H₃*); **δ_C** (100 MHz, $CDCl_3$) 38.64, 102.57, 111.60, 112.98, 121.67, 122.88, 123.41, 126.98, 127.19, 127.73, 128.44, 135.89, 137.38, 142.91, 144.02, 166.76; **IR** (thin film) ν_{max}/cm^{-1}: 1684, 1627, 1461, 1170; **HRMS** *m/z* (ESI^+) found $[M+H]^+$ 342.0803; $C_{18}H_{16}NO_4S^+$ requires 342.0800.

3-oxo-2-phenyl-2,3-dihydro-1H-pyrrolo[1,2-a]indol-8-yl methanesulfonate (**7**)

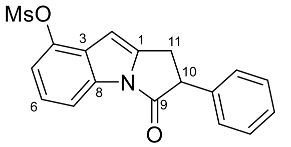

Synthesised from compound **S5** (102.4 mg, 0.3 mmol) using **General Procedure B1**. The resulting crude material was purified by flash column chromatography (10% Et_2O/petroleum ether) to obtain the desired compound as a pale yellow oil (53.2 mg, 0.156 mmol, 52%); **δ_H** (500 MHz, $CDCl_3$) 8.05 (1H, d, J = 7.9 Hz, H7), 7.41-7.21 (7H, m, Ph*H*, H5, H6), 6.54 (1H, s, H3), 4.40 (1H, dd, J = 9.2, 4.9 Hz, H10), 3.71 (1H, ddd, J = 17.7, 9.2, 1.5 Hz, H11), 3.29 (1H, ddd, J = 17.7, 4.9, 1.6 Hz, H11), 3.21 (3H, s, C*H₃*); **δ_C** (125 MHz, $CDCl_3$) 29.60, 37.66, 52.70, 97.66, 113.18, 117.47, 124.21, 127.77, 127.99, 129.23, 132.40, 137.86, 141.11, 143.18, 171.93; **IR** (thin film) ν_{max}/cm^{-1}: 1726, 1583, 1185; **HRMS** *m/z* (ESI^+) found $[M+H]^+$ 342.0803; $C_{18}H_{16}NO_4S^+$ requires 342.0800.

1-(5-fluoro-1H-indol-1-yl)-2-phenylprop-2-en-1-one (**S6**)

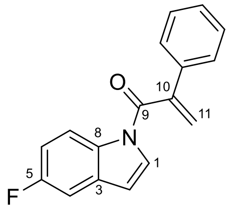

Synthesised from atropic acid (658 mg, 4.44 mmol) and 5-fluoroindole (500 mg, 3.70 mmol) using **General Procedure A2**. The resulting crude material was purified by flash column chromatography (10% EtOAc/petroleum ether) to obtain the desired compound as a white solid (516 mg, 1.95 mmol, 53%); **m.p.** = 126-127 °C; **δ_H** (400 MHz, $CDCl_3$): 8.53 (1H, dd, *J* = 9.0, 4.8 Hz, H4), 7.51-7.45 (2H, m, *o*-Ph*H*), 7.42-7.35 (3H, m, *p*-Ph*H*, *m*-Ph*H*), 7.34 (1H, d, *J* = 3.8 Hz, H1), 7.22 (1 H, dd, *J* = 8.8, 2.5 Hz, H7), 7.12 (1H, dt, *J* = 8.8, 2.5 Hz, H6), 6.50 (1H, dd, *J* = 3.8, 0.7 Hz, H2), 6.09 (1H, s, H11), 5.71 (1H, s, H11); **δ_C** (100 MHz, $CDCl_3$): 106.67 (d, *J* = 23.9 Hz), 108.90 (d, *J* = 3.8Hz), 112.91 (d, *J* = 24.9 Hz), 117.84 (d, *J* = 9.1Hz), 118.90, 126.20, 128.58, 129.21, 129.28, 132.04, 132.14, 135.38, 144.23, 160.10 (d, *J* = 241.0Hz), 168.38; **δ_F** (376 MHz, $CDCl_3$): -118.52; **IR** (thin film) ν_{max}/cm^{-1}: 1728, 1623, 1585; **HRMS** *m/z* (ESI^+) found $[M+H]^+$ 266.0980; $C_{17}H_{13}NOF^+$ requires 266.0981.

7-floro-2-phenyl-1,2-dihydro-3H-pyrrolo[1,2-a]indol-3-one (**8**)

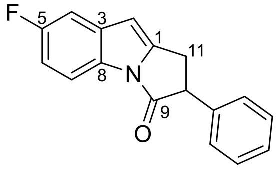

Synthesised from compound **S6** (79.6 mg, 0.3 mmol) using **General Procedure B1**. The resulting crude material was purified by column chromatography (5% AcOEt/pentane) to give the tiled compound as a white solid (75.9 mg, 0.25 mmol, 83%); **m.p.** 148 °C; **δ_H** (400 MHz, $CDCl_3$): 8.02 (1H, dd, *J* = 8.8, 4.7 Hz, H7), 7.41 – 7.27 (5H, m, Ph*H*), 7.20 (1H, dd, *J* = 9.2, 2.5 Hz, H4), 7.02 (1H, td, *J* = 9.0, 2.5 Hz, H6), 6.33 (1H, q, *J* = 1.3 Hz, H2), 4.38 (1H, dd, *J* = 9.1, 4.7 Hz, H10), 3.71 (1H, ddd, *J* = 17.6, 9.2, 1.5 Hz, H11), 3.27 (1H, ddd, *J* = 17.6, 4.7, 1.6 Hz, H11); **δ_C** (100 MHz, $CDCl_3$): 171.62, 160.30 (d, *J* = 240.4 Hz), 143.67, 138.30, 136.63 (d, *J* = 10.1 Hz), 129.29, 127.99, 127.81, 127.24, 114.71 (d, *J* = 9.8 Hz), 111.36 (d, *J* = 25.5 Hz), 106.65 (d, *J* = 24.2 Hz), 100.74 (d, *J* = 3.8 Hz), 52.64, 29.74; **δ_F** (376 MHz, $CDCl_3$): -117.86 (td, *J* = 9.4, 4.8 Hz); **IR** (thin film) ν_{max}/cm^{-1}: 1726, 1497, 1165; **HRMS** *m/z* (ESI^+) found $[M+H]^+$ 266.0975; $C_{17}H_{13}FNO_2^+$ requires 266.0976.

1-(5-bromo-1H-indol-1-yl)-2-phenylprop-2-en-1-one (**S7**)

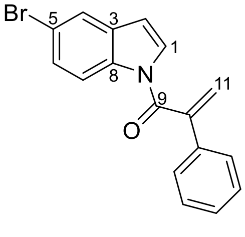

Synthesised from atropic acid (4.0 g, 27.0 mmol) and 5-bromoindole (3.38 g, 18.0 mmol) using **General Procedure A1**. The resulting crude material was purified by column chromatography (4% Et_2O/pentane)

to give the titled compound as an off-white solid (3.46 g, 10.62 mmol, 59%); **m.p.** 75-76 °C; **δ_H** (400 MHz, $CDCl_3$): 8.43 (1H, d, *J* = 8.8 Hz, H7), 7.70 (1H, d, *J* = 2.0 Hz, H4), 7.55 – 7.41 (3H, m, H6, Ph*H*), 7.42 – 7.33 (3H, m, Ph*H*), 7.30 (1H, d, *J* = 3.8 Hz, H1), 6.47 (1H, dd, *J* = 3.8, 0.8 Hz, H2), 6.09 (1H, s, H11), 5.71 (1H, s, H11); **δ_C** (100 MHz, $CDCl_3$): 168.49, 144.23, 135.30, 134.38, 132.76, 129.33, 129.23, 128.17, 128.11, 126.21, 123.74, 119.11, 118.17, 117.61, 108.37. **IR** (thin film) ν_{max}/cm^{-1}: 1693, 1407, 1331; **HRMS** *m/z* (ESI^+) found $[M+H]^+$ 326.0176; $C_{17}H_{13}NBrO^+$ requires 326.0175.

7-bromo-2-phenyl-1,2-dihydro-3H-pyrrolo[1,2-a]indol-3-one (**9**)

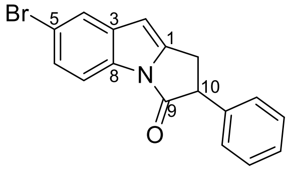

Synthesised from compound **S7** (97.9 mg, 0.3 mmol) using **General Procedure B1**. The resulting crude material was purified by column chromatography (5% AcOEt/pentane) to give the tiled compound as a white solid (92.0 mg, 0.28 mmol, 94%); **m.p.** 112 °C; **δ_H** (400 MHz, $CDCl_3$): 7.87 (1H, dt, *J* = 8.5, 0.7 Hz, H7), 7.61 (1H, d, *J* = 1.8 Hz, H4), 7.35 – 7.16 (6H, m, Ph*H*, H6), 6.26 – 6.20 (1H, m, H2), 4.30 (1H, dd, *J* = 9.2, 4.8 Hz, H10), 3.63 (1H, ddd, *J* = 17.6, 9.2, 1.5 Hz, H11), 3.20 (1H, ddd, *J* = 17.6, 4.8, 1.6 Hz, H11); **δ_C** (100 MHz, $CDCl_3$): 172.10, 143.49, 138.48, 137.51, 129.76, 129.63, 128.35, 128.12, 126.82, 123.89, 118.06, 115.47, 100.45, 53.03, 30.01; **IR** (thin film) ν_{max}/cm^{-1}: 1731, 1584, 1449, 1384, 1349; **HRMS** *m/z* (ESI^+) found $[M+H]^+$ 326.0175; $C_{17}H_{13}BrNO_2^+$ requires 326.0175.

1-(5-iodo-1H-indol-1-yl)-2-phenylprop-2-en-1-one (**S8**)

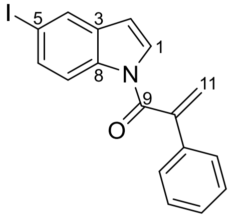

Synthesised from atropic acid (1.0 g, 6.75 mmol) and 5-iodoindole (1.09 g, 4.5 mmol) using **General Procedure A1** where toluene was replaced by CH_2Cl_2. The resulting crude material was purified by column chromatography (2% Et_2O/pentane) to give the titled compound as a white solid (494 mg, 1.31 mmol, 29%); **m.p.** 63 °C; **δ_H** (400 MHz, $CDCl_3$): 8.31 (1H, dt, *J* = 8.7, 0.7 Hz, H7), 7.91 (1H, dd, *J* = 1.7, 0.5 Hz, H4), 7.67 (1H, dd, *J* = 8.7, 1.7 Hz, H6), 7.48 – 7.41 (2H, m, Ph*H*), 7.41 – 7.34 (3H, m, Ph*H*), 7.26 (1H, d, *J* = 3.8 Hz, H1), 6.46 (1H, dd, *J* = 3.8, 0.8 Hz, H2), 6.09 (1H, s, H11), 5.71 (1H, s, H11); **δ_C** (100 MHz, $CDCl_3$): 168.54, 144.25, 135.29, 134.94, 133.79, 133.28, 129.92, 129.33, 129.23, 127.82, 126.20, 119.12, 118.56, 108.14, 88.47; **IR** (thin film) ν_{max}/cm^{-1}: 1691, 1537, 1442, 1366; **HRMS** *m/z* (ESI^+) found $[M+H]^+$ 374.0037; $C_{17}H_{13}NIO^+$ requires 374.0036.

7-iodo-2-phenyl-1,2-dihydro-3H-pyrrolo[1,2-a]indol-3-one (**10**)

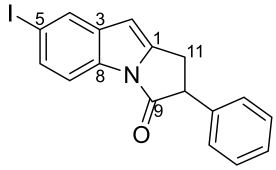

Synthesised from compound **S8** (112.0 mg, 0.3 mmol) using **General Procedure B1**. The resulting crude material was purified by column chromatography (5% AcOEt/pentane) to give the tiled compound as a white solid (93.3 mg, 0.25 mmol, 83%); **m.p.** 141-145 °C; **δ_H** (400 MHz, $CDCl_3$): 7.82 (1H, d, *J* = 1.6 Hz, H4), 7.77 (1H, dd, *J* = 8.5, 0.7 Hz, H7), 7.50 (1H, dd, *J* = 8.5, 1.6 Hz, H6), 7.34 – 7.17 (5H, m, Ph*H*), 6.22 (1H, q, *J* = 1.1 Hz, H2), 4.30 (1H, dd, *J* = 9.2, 4.8 Hz, H10), 3.63 (1H, ddd, *J* = 17.6, 9.2, 1.5 Hz, H11), 3.20 (1H, ddd, *J* = 17.6, 4.8, 1.6 Hz, H11); **δ_C** (100 MHz, $CDCl_3$): 71.85, 142.79, 138.16, 137.69, 132.17, 129.94, 129.73, 129.32, 128.04, 127.85, 127.81, 115.58, 99.85, 88.65, 52.76, 29.65; **IR** (thin film) ν_{max}/cm^{-1}: 1722, 1587, 1443, 1391, 1351; **HRMS** *m/z* (ESI^+) found $[M+H]^+$ 374.0038; $C_{17}H_{13}INO^+$ requires 374.0036.

methyl 1-(2-phenylacryloyl)-1H-indole-5-carboxylate (**S9**)

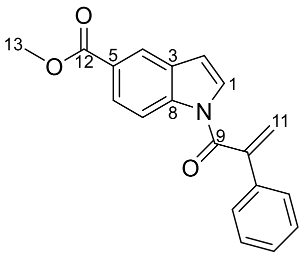

Synthesised from atropic acid (0.5 g, 3.375 mmol) and methyl 1H-indole-5-carboxylate (394 mg, 2.25 mmol) using **General Procedure A1**. The resulting crude material was purified by column chromatography (7.5% AcOEt/pentane) to give the titled compound as a white solid (162 mg, 0.54 mmol, 24%); **m.p.** 171-173 °C; **δ_H** (400 MHz, $CDCl_3$): 8.58 (1H, dt, *J* = 8.8, 0.7 Hz, H7), 8.29 (1H, dd, *J* = 1.8, 0.7 Hz, H4), 8.09 (1H, dd, *J* = 8.7, 1.7 Hz, H6), 7.51 – 7.43 (2H, m, Ph*H*), 7.43 – 7.32 (4H, m, H1, Ph*H*), 6.60 (1H, dd, *J* = 3.8, 0.7 Hz, H2), 6.10 (1H, s, H11), 5.74 (1H, s, H11), 3.95 (3H, s, H13); **δ_C** (100 MHz, $CDCl_3$): 168.64, 167.41, 144.31, 138.32, 135.27, 130.87, 129.37, 129.25, 129.23, 128.25, 126.60, 126.25, 123.23, 119.28, 116.45, 109.48, 52.25; **IR** (thin film) ν_{max}/cm^{-1}: 1708, 1695, 1493, 1209; **HRMS** *m/z* (ESI^+) found $[M+H]^+$ 306.1126; $C_{19}H_{16}NO_3^+$ requires 306.1125.

methyl 3-oxo-2-phenyl-2,3-dihydro-1H-pyrrolo[1,2-a]indole-7-carboxylate (**11**)

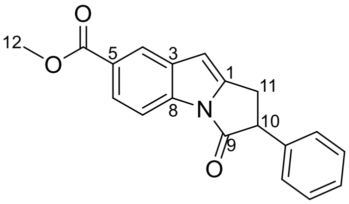

Synthesised from compound **S9** (91.6 mg, 0.3 mmol) using **General Procedure B1**. The resulting crude material was purified by column chromatography (10% AcOEt/pentane) to give the tiled compound as a

white solid (56.5 mg, 0.19 mmol, 62%); **m.p.** 171-173 °C; **δ_H** (400 MHz, $CDCl_3$): 8.27 (1H, dd, *J* = 1.6, 0.7 Hz, H4), 8.09 (1H dt, *J* = 8.6, 0.7 Hz, H7), 7.99 (1H, dd, *J* = 8.5, 1.6 Hz, H6), 7.40 – 7.24 (5H, m, Ph*H*), 6.42 (1H, td, *J* = 1.6, 0.7 Hz, H2), 4.39 (1H, dd, *J* = 9.2, 4.9 Hz, H10), 3.93 (3H, s, H12), 3.71 (1H, ddd, *J* = 17.6, 9.3, 1.5 Hz, H11), 3.29 (1H, ddd, *J* = 17.6, 4.9, 1.7 Hz, H11); **δ_C** (100 MHz, $CDCl_3$): 171.99, 167.52, 142.99, 138.08, 135.32, 133.30, 129.32, 128.05, 127.82, 126.29, 125.08, 123.05, 113.55, 101.20, 52.78, 52.28, 29.71; **IR** (thin film) ν_{max}/cm^{-1}: 1739, 1709, 1381, 1295; **HRMS** *m/z* (ESI^+) found $[M+H]^+$ 306.1125; $C_{19}H_{16}NO_3^+$ requires 306.1125.

1-(5-methoxy-1H-indol-1-yl)-2-phenylprop-2-en-1-one (**S10**)

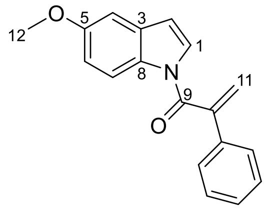

Synthesised from atropic acid (0.5 g, 3.375 mmol) and indole (331 mg, 2.25 mmol) using **General Procedure A1**. The resulting crude material was purified by column chromatography (7% Et_2O/pentane) to give the titled compound as a colourless oil (253 mg, 0.90 mmol, 40%); **δ_H** (400 MHz, $CDCl_3$): 8.45 (1H, dt, *J* = 8.9, 0.8 Hz, H7), 7.50 – 7.45 (2H, m, Ph*H*), 7.40 – 7.33 (3H, m, Ph*H*), 7.28 (1H, d, *J* = 3.8 Hz, H1), 7.05 – 6.98 (2H, m, H4, H6), 6.47 (1H, dd, *J* = 3.8, 0.8 Hz, H2), 6.06 (1H, s, H11), 5.68 (1H, s, H11), 3.87 (3H, s, H12); **δ_C** (100 MHz, $CDCl_3$): 168.32, 157.04, 144.44, 135.56, 132.11, 130.35, 129.18, 129.14, 127.76, 126.23, 118.51, 117.61, 113.54, 109.21, 103.95, 55.85; **IR** (thin film) ν_{max}/cm^{-1}: 1684, 1470, 1244; **HRMS** *m/z* (ESI^+) found $[M+H]^+$ 278.1178; $C_{18}H_{16}NO_2^+$ requires 287.1176.

7-methoxy-2-phenyl-1,2-dihydro-3H-pyrrolo[1,2-a]indol-3-one (**12**)

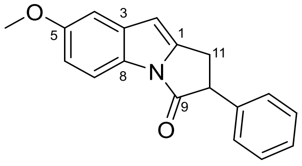

Synthesised from compound **S10** (83.2 mg, 0.30 mmol) using **General Procedure B1**. The crude material was purified by flash column chromatography (5% Et_2O/petroleum ether) to obtain the titled compound **12** as a white solid (58.1 mg, 0.21 mmol, 70%); **m.p.** 165-166 °C; **δ_H** (400 MHz, $CDCl_3$): 7.96 (1H, d, *J* = 8.7 Hz, H7), 7.39-7.32 (2H, m, *o*-Ph*H*), 7.32-7.24 (3H, m, *p*-Ph*H*, *m*-Ph*H*), 7.01 (1H, d, *J* = 2.5 Hz, H4), 6.89 (1H, dd, *J* = 8.7, 2.5 Hz, H6), 6.28 (1H, s, H2), 4.33 (1H, dd, *J* = 9.1, 4.7 Hz, H10), 3.85 (3H, s, C*H₃*), 3.65 (1H, ddd, *J* = 17.5, 9.1, 1.5 Hz, H11), 3.21 (1H, ddd, *J* = 17.5, 4.7, 1.5 Hz, H11); **δ_C** (100 MHz, $CDCl_3$): 29.72, 52.69, 55.81, 100.74, 103.94, 111.81, 114.49, 125.48, 127.80, 129.18, 136.66, 138.61, 142.80, 157.18, 171.48; **IR** (thin film) ν_{max}/cm^{-1}: 1723, 1445; **HRMS** *m/z* (ESI^+) found $[M+H]^+$ 278.1185; $C_{18}H_{16}NO_2^+$ requires 278.1181.

2-phenyl-1-(6-(4,4,5,5-tetramethyl-1,3,2-dioxaborolan-2-yl)-1H-indol-1-yl)prop-2-en-1-one (**S11**)

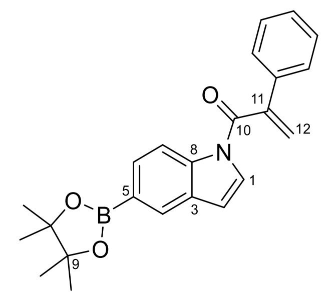

A solution of 1-(5-bromo-1H-indol-1-yl)-2-phenylprop-2-en-1-one (300 mg, 0.920 mmol, 1.0 equiv.) and B_2pin_2 (350 mg, 1.38 mmol, 1.5 equiv.) in anhydrous dioxane (1.63 mL) was treated with KOAc (151 mg, 1.54 mmol, 1.7 equiv.). The suspension was purged with Ar for 15 min, treated with $PdCl_2$(dppf) (33.6 mg, 0.046 mmol, 5 mol%), then purged with Ar for another 15 min. The reaction was stirred at 80 °C overnight. After cooling down, the mixture was filtered through a plug of silica gel (elution with 100 mL of EtOAc). The filtrate was concentrated *in vacuo*, and purified by flash column chromatography (25% EtOAc/petroleum ether) to afford the titled compound as a colourless liquid (191 mg, 0.512 mmol, 56%); **δ_H** (400 MHz, $CDCl_3$): 9.00 (1H, s, H4), 7.71 (1H, d, *J* = 7.8 Hz, H6), 7.49 (1H, d, *J* = 7.8 Hz, H7), 7.44-7.37 (2H, m, *o*-Ph*H*), 7.32-7.22 (4H, m, *p*-Ph*H*, *m*-Ph*H*, H1), 6.45 (1H, d, *J* = 3.9 Hz, H2), 5.98 (1H, s, H12), 5.60 (1H, s, H12), 1.31 (12H, s, C*H*$_3$); **δ_C** (100 MHz, $CDCl_3$): 24.93, 83.78, 109.08, 118.55, 120.28, 123.07, 125.78 (br, s), 126.08, 128.06, 128.99, 129.04, 130.22, 133.40, 135.32, 144.34, 168.28; **δ_B** (128 MHz, $CDCl_3$): 30.59; **IR** (thin film) ν_{max}/cm^{-1}: 1722, 1656, 1562; **HRMS** *m/z* (ESI$^+$) found $[M+H]^+$ 374.1930; $C_{23}H_{25}NO_3B^+$ requires 374.1927.

2-phenyl-6-(4,4,5,5-tetramethyl-1,3,2-dioxaborolan-2-yl)-1,2-dihydro-3H-pyrrolo[1,2-a]indol-3-one (**13**)

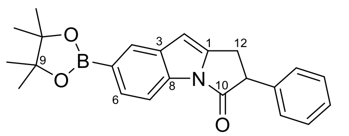

Synthesised from compound **S11** using **General Procedure B1**. The crude material was purified by column chromatography (15% EtOAc/petroleum ether) to obtain the titled compound **13** as a thick colourless liquid (64 mg, 0.17 mmol, 66%); **δ_H** (400 MHz, $CDCl_3$): 8.76 (1H, s, H4), 7.74 (1H, d, *J* = 7.8 Hz, H6), 7.69-7.67 (2H, m, *o*-Ph*H*), 7.50 (1H, d, *J* = 7.8 Hz, H7), 7.38-7.35 (3H, m, *p*-Ph*H*, *m*-Ph*H*), 6.39 (1H, s, H2), 4.21 (1H, dd, *J* = 8.8, 4.5 Hz, H11), 3.45 (1H, ddd, *J* = 16.8, 8.8, 1.4 Hz, H12'), 3.00 (1H, ddd, *J* = 16.8, 4.5, 1.4 Hz, H12''); **δ_C** (100 MHz, $CDCl_3$): 25.08, 29.63, 52.80, 83.91, 109.21, 120.39, 123.19, 125.64, 127.77, 127.81, 128.19, 129.17, 130.33, 134.72, 135.43, 138.49, 171.88; **δ_B** (128 MHz, $CDCl_3$) 31.35; **IR** (thin film) ν_{max}/cm^{-1}: 1738, 1622, 1547; **HRMS** *m/z* (ESI$^+$) found $[M+H]^+$ 374.1930; $C_{23}H_{25}NO_3B^+$ requires 374.1927.

1-(6-bromo-1H-indol-1-yl)-2-phenylprop-2-en-1-one (**S12**)

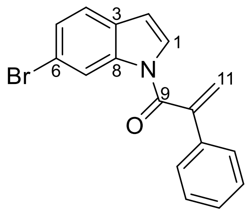

Synthesised from atropic acid (0.5 g, 3.375 mmol) and 6-bromoindole (422 mg, 2.25 mmol) using **General Procedure A1**. The resulting crude material was purified by column chromatography (3% Et_2O/pentane) to give the titled compound as a white solid (350 mg, 1.08 mmol, 48%); **m.p.** 75-76 °C; **δ_H** (400 MHz, $CDCl_3$): 8.78 (1H, dt, *J* = 1.6, 0.7 Hz, H7), 7.56 – 7.29 (7H, m, H4, H5, Ph*H*), 7.26 (1H, d, *J* = 3.8 Hz, H1), 6.49 (1H, dd, *J* = 3.8, 0.8 Hz, H2), 6.09 (1H, s, H11), 5.71 (1H, s, H11); **δ_C** (100 MHz, $CDCl_3$): 168.52, 144.21, 136.30, 135.27, 129.81, 129.34, 129.27, 129.23, 127.58, 127.51, 126.20, 122.04, 119.96, 119.09, 118.98, 108.92; **IR** (thin film) ν_{max}/cm^{-1}: 1692, 1496, 1317, 1347, 1200; **HRMS** *m/z* (ESI$^+$) found [M+H]$^+$ 326.0177; $C_{17}H_{13}NBrO^+$ requires 326.0175.

6-bromo-2-phenyl-1,2-dihydro-3H-pyrrolo[1,2-a]indol-3-one (**14**)

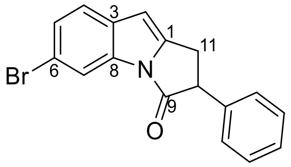

Synthesised from compound **S12** (97.9 mg, 0.3 mmol) using **General Procedure B1**. The resulting crude material was purified by column chromatography (5% Et_2O/pentane) to give the tiled compound as a white solid (92.9 mg, 0.28 mmol, 94%); **m.p.** 135-137 °C; **δ_H** (400 MHz, $CDCl_3$): 8.18 (1H, dt, *J* = 1.6, 0.9 Hz, H7), 7.37 – 7.16 (7H, m, Ph*H*, H4, H5), 6.25 (1H, td, *J* = 1.6, 0.7 Hz, 1H, H2), 4.30 (dd, *J* = 9.2, 4.8 Hz, H10), 3.61 (1H, ddd, *J* = 17.5, 9.2, 1.5 Hz, H11), 3.17 (1H, ddd, *J* = 17.6, 4.8, 1.6 Hz, H11). **δ_C** (100 MHz, $CDCl_3$): 171.77, 142.30, 138.20, 134.32, 131.32, 129.30, 128.02, 127.83, 127.80, 127.78, 127.62, 121.91, 117.08, 116.98, 100.58, 52.73, 29.75; **IR** (thin film) ν_{max}/cm^{-1}: 1732, 1602, 1441; **HRMS** *m/z* (ESI$^+$) found [M+H]$^+$ 326.0177; $C_{17}H_{13}BrNO_2^+$ requires 326.0175.

2-phenyl-1-(6-(trifluoromethyl)-1H-indol-1-yl)prop-2-en-1-one (**S13**)

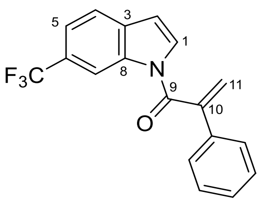

Synthesised from atropic acid (107 mg, 0.72 mmol) and 6-(trifluoromethyl)-1H-indole (111 mg, 0.60 mmol) using **General Procedure A2**. The crude residue was purified by flash column chromatography (10→20% EtOAc/petroleum ether) to afford the titled product as a pale-yellow oil (150 mg, 0.47 mmol, 79%). **δ_H** (400 MHz, $CDCl_3$): 8.93 (1H, ap. d, *J* = 0.8 Hz, H7), 7.66 (1H, d, *J* = 8.3 Hz, H4), 7.58 (1H, dd, *J* = 8.3, 1.6 Hz, H5), 7.52-7.46 (2H, m, *o*-Ph*H*), 7.45 (1H, d, *J* = 3.8 Hz, H1), 7.43-7.35 (3H, m, *p*-Ph*H*, *m*-Ph*H*), 6.60 (1H, dd,

J = 3.8, 0.8 Hz, H2), 6.12 (1H, s, H11), 5.75 (1H, s, H11); **δ$_C$** (100 MHz, $CDCl_3$): 108.75, 114.30 (q, J = 4.4 Hz), 119.43, 121.04 (q, J = 3.6 Hz), 121.34, 124.86 (q, J = 272.0 Hz), 126.18, 127.26 (q, J = 32.1 Hz), 129.23, 129.37, 129.48, 133.55, 134.85, 135.18, 144.06, 168.58; **δ$_F$** (376 MHz, $CDCl_3$): -60.90; **IR** (thin film) ν_{max}/cm^{-1}: 1712, 1642; **HRMS** *m/z* (ESI^+) found $[M+H]^+$ 316.0951; $C_{18}H_{13}F_3NO^+$ requires 316.0949.

2-phenyl-1-(6-(trifluoromethyl)-1H-indol-1-yl)prop-2-en-1-one (**15**)

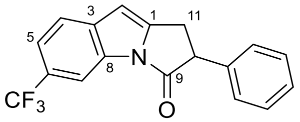

Synthesised from compound **S13** (81.0 mg, 0.3 mmol) using **General Procedure B1**. The resulting crude material was purified by column chromatography (20% AcOEt/petroleum ether) to give the tiled compound as a white solid (60.0 mg, 0.22 mmol, 74%); **m.p.** 125 °C; **δ$_H$** (400 MHz, $CDCl_3$): 8.38 (1H, dt, J = 1.7, 0.8 Hz, H7), 7.64 (1H, d, J = 8.3 Hz, H4), 7.55 (1H, dd, J = 8.5, 1.6 Hz, H5), 7.42 – 7.27 (5H, m, Ph*H*), 6.43 (1H, t, J = 1.2 Hz, H2), 4.43 (1H, dd, J = 9.2, 4.7 Hz, H10), 3.76 (1H, ddd, J = 17.7, 9.2, 1.5 Hz, H11), 3.32 (1H, ddd, J = 17.6, 4.8, 1.6 Hz, H11); **δ$_C$** (100 MHz, $CDCl_3$): ^{13}C NMR (101 MHz, $CDCl_3$) δ 171.85, 144.66, 138.00, 129.36, 128.12 (2C, s), 127.79, 121.23 (2C, m), 121.07 (2C, s), 111.36, 100.66, 52.69, 29.88; **δ$_F$** (376 MHz, $CDCl_3$): -60.96; **IR** (thin film) ν_{max}/cm^{-1}: 1736, 1448, 1318; **HRMS** *m/z* (ESI^+) found $[M+H]^+$ 316.0949; $C_{18}H_{14}NO_2^+$ requires 316.0949.

1-(2-phenylacryloyl)-1H-indole-6-carbaldehyde (**S14**)

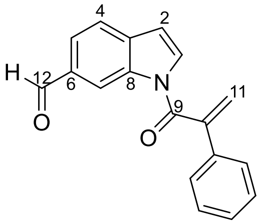

Synthesised from atropic acid (0.5 g, 3.375 mmol) and 1H-indole-6-carbaldehyde (327 mg, 2.25 mmol) using **General Procedure A1**. The resulting crude material was purified by column chromatography (15% AcOEt/pentane) to give the titled compound as an off-white solid (405 mg, 1.46 mmol, 65%); **m.p**: 103-105 °C; **δ$_H$** (400 MHz, $CDCl_3$): 10.12 (1H, s, H12), 9.04 (1H, dt, J = 1.5, 0.7 Hz, H7), 7.89 (1H, dd, J = 8.1, 1.4 Hz, H4), 7.67 (1H, d, J = 8.1 Hz, H4), 7.53 – 7.44 (3H, m, Ph*H*), 7.44 – 7.34 (3H, m, H1, Ph*H*), 6.61 (1H, dd, J = 3.7, 0.8 Hz, H2), 6.13 (1H, s, H11), 5.76 (1H, s, H11); **δ$_C$** (100 MHz, $CDCl_3$): 192.31, 168.53, 144.07, 135.94, 135.38, 135.19, 133.95, 130.65, 129.44, 129.34, 129.29, 126.22, 124.11, 121.58, 120.61, 119.58, 109.16. **IR** (thin film) ν_{max}/cm^{-1}: 1732, 1674, 1453, 1365, 1327; **HRMS** *m/z* (ESI^+) found $[M+H]^+$ 276.1020; $C_{18}H_{14}NO_2^+$ requires 276.1019.

3-oxo-2-phenyl-2,3-dihydro-1H-pyrrolo[1,2-a]indole-6-carbaldehyde (**16**)

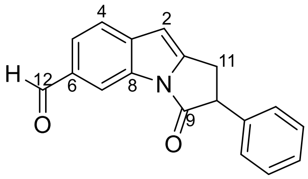

Synthesised from compound **S14** (82.6 mg, 0.3 mmol) using **General Procedure B2**. The resulting crude material was purified by column chromatography (15% AcOEt/pentane) to give the tiled compound as a white solid (26.8 mg, 0.1 mmol, 32%); **m.p.** 179 °C; **δ_H** (400 MHz, $CDCl_3$): 9.98 (1H, s, H12), 8.48 (1H, dt, *J* = 1.6, 0.8 Hz, H7), 7.79 (1H, dd, *J* = 8.2, 1.5 Hz, H5), 7.58 (1H, d, *J* = 8.2 Hz, H4), 7.35 – 7.18 (5H, m, Ph*H*), 6.38 (1H, q, *J* = 1.3 Hz, H2), 4.37 (1H, dd, *J* = 9.2, 4.7 Hz, H10), 3.70 (1H, ddd, *J* = 17.9, 9.2, 1.5 Hz, H11), 3.26 (1H, ddd, *J* = 17.8, 4.8, 1.6 Hz, H11); **δ_C** (100 MHz, $CDCl_3$): 191.89, 171.81, 146.36, 140.60, 137.86, 132.47, 130.42, 129.37, 128.14, 127.79, 124.70, 121.24, 116.87, 101.18, 52.62, 30.05; **IR** (thin film) ν_{max}/cm^{-1}: 1732, 1674, 1452, 1364, 1327; **HRMS** *m/z* (ESI^+) found $[M+H]^+$ 276.1019; $C_{18}H_{14}NO_2^+$ requires 256.1019.

1-(7-fluoro-1H-indol-1-yl)-2-phenylprop-2-en-1-one (**S15**)

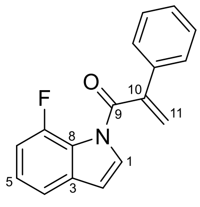

Synthesised from atropic acid (658 mg, 5.33mmol) and 7-fluoroindole (500 mg, 3.70 mmol) using **General Procedure A2**. The resulting crude material was purified by flash column chromatography (10% EtOAc/petroleum ether) to obtain the desired compound as a white solid (755 mg, 2.85 mmol, 77%); **m.p.** 133-135 °C; **δ_H** (400 MHz, $CDCl_3$) 7.43-7.37 (2H, m, *o*-Ph*H*), 7.30 (1H, d, *J* = 3.7 Hz, H2), 7.28-7.19 (4H, m, H4, *p*-Ph*H*, *m*-Ph*H*), 7.10 (1H, td, *J* = 8.1, 4.2 Hz, H5), 6.95 (1 H, dd, *J* = 12.0, 8.2 Hz, H6), 6.46 (1H, dd, *J* = 3.7, 1.9 Hz, H3), 5.99 (1H, s, H11), 5.69 (1H, s, H11); **δ_C** (100 MHz, $CDCl_3$) 108.74 (d, *J* = 1.8 Hz), 111.87 (d, *J* = 20.9 Hz), 116.90 (d, *J* = 3.7 Hz), 121.91, 122.62 (d, *J* = 11.1 Hz), 124.76 (d, *J* = 6.9 Hz), 126.84, 128.64, 128.90, 129.01, 134.87 (d, *J* = 4.0 Hz), 135.67, 144.37, 150.69 (d, *J* = 253.2 Hz), 166.86; **δ_F** (376 MHz, $CDCl_3$) -112.08; **IR** (thin film) ν_{max}/cm^{-1}: 1730, 1613, 1570; **HRMS** *m/z* (ESI^+) found $[M+H]^+$ 266.0980; $C_{17}H_{13}NOF^+$ requires 266.0981.

5-fluoro-2-phenyl-1,2-dihydro-3H-pyrrolo[1,2-a]indol-3-one (**17**)

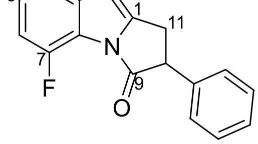

Synthesised from compound **S15** (79.6 mg, 0.3 mmol) using **General Procedure B1**. The resulting crude material was purified by column chromatography (5% AcOEt/pentane) to give the tiled compound as a white solid (65.6 mg, 0.25 mmol, 82%); **m.p.** 125 °C; **δ_H** (400 MHz, $CDCl_3$): 7.42 – 7.27 (6H, m, H4, Ph*H*),

7.22 (1H, td, *J* = 7.9, 4.3 Hz, H6), 7.01 (1H, ddd, *J* = 10.9, 8.0, 1.0 Hz, H5), 6.41 (1H, q, *J* = 1.7 Hz, H2), 4.40 (1H, dd, *J* = 9.3, 4.9 Hz, H10), 3.72 (1H, ddd, *J* = 17.4, 9.3, 1.5 Hz, H11), 3.31 (1H, ddd, *J* = 17.5, 4.9, 1.6 Hz, H11); **δ_C** (100 MHz, $CDCl_3$):170.19, 150.80 (d, *J* = 253.4 Hz), 143.39, 138.99 (d, *J* = 4.8 Hz), 138.31, 129.23, 127.96, 127.83, 125.27 (d, *J* = 6.4 Hz), 118.36 (d, *J* = 13.7 Hz), 116.42 (d, *J* = 3.7 Hz), 110.51 (d, *J* = 18.7 Hz), 101.13 (d, *J* = 1.9 Hz), 52.42, 29.39; **δ_F** (376 MHz, $CDCl_3$): -117.64 (ddd, *J* = 10.8, 4.5, 2.1 Hz); **IR** (thin film) ν_{max}/cm^{-1}: 1747, 1642, 1432, 1274; **HRMS** *m/z* (ESI^+) found $[M+H]^+$ 266.0976; $C_{17}H_{13}FNO_2^+$ requires 266.0976.

2-phenyl-1-(1H-pyrrolo[2,3-c]pyridin-1-yl)prop-2-en-1-one (**S16**)

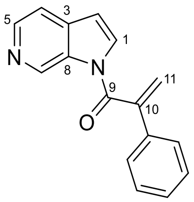

Synthesised from atropic acid (0.376 g, 2.54 mmol) and 6-azaindole (0.25 g, 2.12 mmol) using **General Procedure A2**. The resulting crude material was purified by flash column chromatography (10% EtOAc/petroleum ether) to obtain the desired compound as a white solid (228 mg, 0.92 mmol, 43%); **m.p.** 137 °C; **δ_H** (400 MHz, $CDCl_3$): 9.65 (1H, s, H6), 8.31 (1H, d, *J* = 5.3 Hz, H4), 7.29 (3H, m, *o*-Ph*H*, H5), 7.27 (1H, d, *J* = 3.6 Hz, H1), 7.18 (3H, m, *p*-Ph*H*, *m*-Ph*H*), 6.36 (1H, d, *J* = 3.6 Hz, H2), 5.94 (1H, s, H11), 5.56 (1H, S, H11); **δ_C** (100 MHz, $CDCl_3$): 107.80, 115.44, 119.59, 125.91, 128.96, 129.14, 129.79, 132.38, 134.77, 136.23, 138.38, 143.31, 143.42, 167.73; **IR** (thin film) ν_{max}/cm^{-1}: 1726, 1635, 1549; **HRMS** *m/z* (ESI^+) found $[M+H]^+$ 294.1230; $C_{16}H_{13}N_2O^+$ requires 294.1028.

7-phenyl-6,7-dihydro-8H-pyrido[4,3-b]pyrrolizin-8-one (**18**)

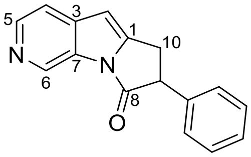

Synthesised from compound **S16** (74.5 mg, 0.3 mmol) using **General Procedure B1**. The resulting crude material was purified by column chromatography (100% AcOEt) to give the tiled compound as a white solid (57.6 mg, 0.23 mmol, 77%); **m.p.** 141-144 °C; **δ_H** (400 MHz, $CDCl_3$): 9.35 (1H, d, *J* = 1.0 Hz, H6), 8.47 (1H, d, *J* = 5.4 Hz, H5), 7.48 (1H, dd, *J* = 5.4, 1.1 Hz, H4), 7.43 – 7.27 (5H, m, Ph*H*), 6.39 (1H, q, *J* = 1.3 Hz, H2), 4.44 (1H, dd, *J* = 9.1, 4.7 Hz, H9), 3.78 (1H, ddd, *J* = 17.8, 9.2, 1.4 Hz, H10), 3.35 (1H, ddd, *J* = 17.8, 4.8, 1.6 Hz, H10); **δ_C** (100 MHz, $CDCl_3$): 171.41, 145.75, 143.93, 141.09, 137.69, 136.01, 129.37, 128.17, 127.98, 127.76, 115.66, 99.91, 52.47, 30.01; **IR** (thin film) ν_{max}/cm^{-1}: 1725, 1599, 1452; **HRMS** *m/z* (ESI^+) found $[M+H]^+$ 249.1024; $C_{16}H_{13}N_2O^+$ requires 249.1022;

2-phenyl-1-(1H-pyrrolo[3,2-c]pyridin-1-yl)prop-2-en-1-one (**S17**)

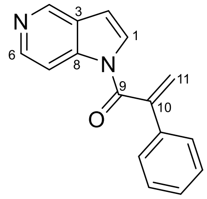

Synthesised from atropic acid (752 mg, 5.08 mmol) and 5-azaindole (500 mg, 4.23 mmol) using **General Procedure A2**. The resulting crude material was purified by flash column chromatography (10% EtOAc/petroleum ether) to obtain the desired compound as a white solid (786 mg, 3.17 mmol, 75%); **m.p.** 132-134 °C; δ_H (400 MHz, $CDCl_3$): 8.91 (1H, s, H4), 8.57 (1H, d, *J* = 5.7 Hz, H6), 8.35 (1H, d, *J* = 5.7 Hz, H7), 7.48-7.44 (2H, m, *o*-Ph*H*), 7.42-7.36 (3H, m, *p*-Ph*H*, *m*-Ph*H*), 7.34 (1H, d, *J* = 3.8 Hz, H1), 6.62 (1H, d, *J* = 3.8 Hz, H2), 6.13 (1H, s, H11), 5.76 (1H, S, H11); δ_C (100 MHz, $CDCl_3$): 107.41, 111.40, 119.86, 126.24, 127.10, 127.70, 129.32, 129.49, 135.01, 140.01, 143.89, 143.99, 145.29, 168.74; **IR** (thin film) ν_{max}/cm^{-1}: 1723, 1639, 1543; **HRMS** *m/z* (ESI$^+$) found $[M+H]^+$ 294.1230; $C_{16}H_{13}N_2O^+$ requires 294.1028.

7-phenyl-7,8-dihydro-6H-pyrido[3,4-b]pyrrolizin-6-one (**19**)

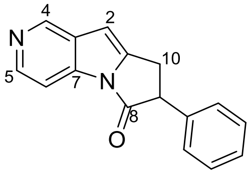

Synthesised from compound **S17** (74.5 mg, 0.3 mmol) using **General Procedure B1**. The resulting crude material was purified by column chromatography (70% AcOEt/DCM) to give the tiled compound as a white solid (97.2 mg, 0.27 mmol, 90%); **m.p.** 170 °C; δ_H (400 MHz, $CDCl_3$): 8.90 (1H, d, *J* = 1.1 Hz, H4), 8.50 (1H, d, *J* = 5.5 Hz, H5), 7.94 (1H, dt, *J* = 5.5, 1.0 Hz, H6), 7.43 – 7.25 (5H, m, Ph*H*), 6.43 (1H, q, *J* = 1.2 Hz, H2), 4.43 (1H, dd, *J* = 9.3, 4.9 Hz, H9), 3.75 (1H, ddd, *J* = 17.7, 9.3, 1.5 Hz, H10), 3.33 (1H, ddd, *J* = 17.7, 5.0, 1.7 Hz, H10); δ_C (100 MHz, $CDCl_3$): 172.45, 144.03, 143.84, 142.83, 138.03, 135.17, 131.85, 129.68, 128.49, 128.10, 109.07, 99.22, 53.19, 30.02; **IR** (thin film) ν_{max}/cm^{-1}: 1734, 1583, 1384; **HRMS** *m/z* (ESI$^+$) found $[M+H]^+$ 249.1024; $C_{16}H_{13}N_2O^+$ requires 249.1022.

2-phenyl-1-(1H-pyrrolo[3,2-b]pyridin-1-yl)prop-2-en-1-one (**S18**)

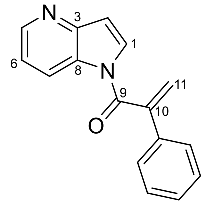

Synthesised from atropic acid (752 mg, 5.08 mmol) and 4-azaindole (0.5 g, 4.23 mmol) using **General Procedure A2**. The resulting crude material was purified by flash column chromatography (10% EtOAc/petroleum) to obtain the desired compound as a white solid (796 mg, 3.21 mmol, 76%); **m.p.** 132-133 °C; δ_H (400 MHz, $CDCl_3$): 8.73 (1H, ddd, *J* = 8.3, 1.8, 0.8 Hz, H5), 8.57 (1H, dd, *J* = 4.8, 1.5 Hz, H7), 7.53 (1H, d, *J* = 3.9 Hz, H1), 7.49-7.41 (2H, m, *o*-Ph*H*), 7.40-7.32 (3H, m, *p*-Ph*H*, *m*-Ph*H*), 7.28 (1H, dd, *J* = 8.3, 4.8 Hz, H6), 6.72 (1H, dd, *J* = 3.9, 0.8 Hz, H2), 6.11 (1H, s, H11), 5.72 (1H, s, H11); δ_C (100 MHz, $CDCl_3$):

110.06, 119.61, 119.66, 123.81, 126.15, 129.14, 129.20, 129.37, 130.03, 135.08, 143.60, 146.88, 149.39, 168.55; **IR** (thin film) ν_{max}/cm^{-1}: 1722, 1640, 1545; **HRMS** *m/z* (ESI^+) found $[M+H]^+$ 294.1230; $C_{16}H_{13}N_2O^+$ requires 294.1028.

7-phenyl-7,8-dihydro-6H-pyrido[2,3-b]pyrrolizin-6-one (**20**)

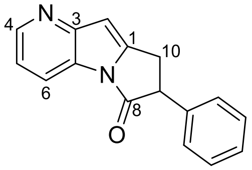

Synthesised from compound **S18** (74.5 mg, 0.3 mmol) using **General Procedure B1**. The resulting crude material was purified by column chromatography (100% AcOEt) to give the tiled compound as a white solid (65.4 mg, 0.26 mmol, 88%); **m.p.** 135-136 °C; **δ_H** (400 MHz, $CDCl_3$): 8.56 (1H, dd, *J* = 4.9, 1.5 Hz, H4), 8.29 (1H, dt, *J* = 8.1, 1.0 Hz, H6), 7.43 – 7.25 (5H, m, Ph*H*), 7.20 (1H, dd, *J* = 8.1, 4.9 Hz, H5), 6.59 (1H, q, *J* = 1.3 Hz, H2), 4.37 (1H, dd, *J* = 9.2, 4.7 Hz, H9), 3.80 (1H, ddd, *J* = 17.8, 9.2, 1.5 Hz, H10), 3.38 (1H, ddd, *J* = 17.8, 4.7, 1.6 Hz, H10); **δ_C** (100 MHz, $CDCl_3$): 172.38, 153.55, 146.83, 145.15, 137.87, 129.35, 128.12, 127.79, 124.57, 120.71, 118.23, 102.07, 51.83, 30.28; **IR** (thin film) ν_{max}/cm^{-1}: 1737, 1607, 1407; **HRMS** *m/z* (ESI^+) found $[M+H]^+$ 249.1024; $C_{16}H_{13}N_2O^+$ requires 249.1022.

2-phenylacrylamide (**S19**)

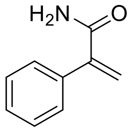

The procedure was based on a modified literature procedure.[23] To a solution of atropic acid (500 mg, 3.37 mmol, 1.0 equiv.) and oxalyl chloride (0.35 mL, 4.1 mmol, 1.2 equiv.) in anhydrous CH_2Cl_2 (6.7 mL), was added a drop of anhydrous DMF. The reaction was then stirred at room temperature for 3 h. The excess amount of solvent and oxalyl chloride were removed *in vacuo*, and (*ca.* 20%) aqueous solution of NH_3 (0.54 mL) was added to the residue under stirring at 0 °C. The reaction was stirred at room temperature for another 1 h. The reaction mixture was extracted with EtOAc (15 mL × 3), then combined organic layer was then washed with brine, dried over Na_2SO_4, and concentrated *in vacuo*. The solid residue was purified by crystallisation (10% EtOAc/petroleum ether) to afford the titled compound as a colourless crystalline solid (367 mg, 2.49 mmol, 74%); **m.p.** 121-123 °C; **δ_H** (400MHz, $CDCl_3$): 7.42-7.36 (5H, m, Ph*H*), 6.23 (1H, s, C*H'*H''), 5.67 (1H, s, CH'*H''*), 5.63 (2H, br. s, N*H₂*); **δ_C** (100 MHz, $CDCl_3$): 128.32, 128.75, 128.89, 137.25, 144.13, 169.03; Data are consistent with literature values.[4]

3. Y. Nai and J. Xu, *Helvetica Chemica Acta*, **2013**, *96*, 1355-1365.
4. M. Schade, G. Manolikakes and P. Knochel, *Org. Lett.*, **2010**, *12*, 3648-3650.

2-phenyl-1-(1H-pyrrol-1-yl)prop-2-en-1-one (**S20**)

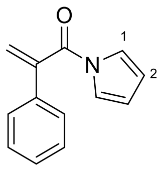

The procedure was based on a modified literature procedure.[4] To a solution of 2-phenylacrylamide (367 mg, 2.49 mmol, 1.0 equiv.) in acetic acid (2.85 mL) was added 2,5-dimethoxytetrahydrofurane (355 mL, 2.74 mmol, 1.1 equiv.). The reaction mixture was heated to 100 °C for 6 h. After cooling, the mixture was then extracted with Et_2O (15 mL × 3). The combined organic phase was washed with brine, dried over Na_2SO_4, and concentrated *in vacuo*. The resulting crude material was purified by flash column chromatography (0→5% EtOAc/petroleum ether) to yield the titled compound as a pale yellow liquid (174 mg, 0.880 mmol, 35%); **δ_H** (400 MHz, $CDCl_3$) 7.44-7.33 (5H, m, Ph*H*), 7.30, 2H, ap. s, H1), 6.27 (2H, ap. s, H2), 6.08 (1H, ap. s, C*H'*H''), 5.70 (1H, ap. s, CH'*H''*); **δ_C** (100 MHz, $CDCl_3$) 113.28, 119.68, 120.28, 126.09, 128.31, 128.88, 137.45, 142.99, 167.19; Data are consistent with literature values.[5]

2-phenyl-1,2-dihydro-3H-pyrrolizin-3-one (**21**)

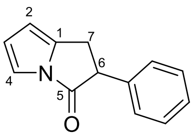

Synthesised from compound **S20** (59.2 mg, 0.3 mmol) using **General Procedure B1**. The crude residue was purified by flash column chromatography (5% EtOAc/petroleum ether) to afford the) to obtain the titled compound **21** as a colourless solid (50.2 mg, 0.26 mmol, 85%); **m.p.** 78-80 °C; **δ_H** (400 MHz, $CDCl_3$): 7.32-7.26 (2H, m, *o*-Ph*H*), 7.26-7.21 (1H, m, *p*-Ph*H*), 7.20-7.16 (2H, m, *m*-Ph*H*), 7.04 (1H, d, *J* = 3.0 Hz, H4), 6.46 (1H, t, *J* = 3.0 Hz, H3), 5.97 (1H, ap. dd, *J* = 3.0, 1.4 Hz, H2), 4.24 (1H, dd, *J* = 8.8, 4.5 Hz, H6), 3.48 (1H, ddd, *J* = 16.8, 8.8, 1.4 Hz, H7'), 3.03 (1H, ddd, *J* = 16.8, 4.5, 1.4 Hz, H7''); **δ_C** (100 MHz, $CDCl_3$): 29.58, 52.92, 104.79, 111.63, 119.48, 127.79 (3 × Ph*C*), 129.15, 137.92, 138.33, 172.31; **IR** (thin film) ν_{max}/cm^{-1}: 1729, 1567, 1285; **HRMS** *m/z* (ESI^+) found $[M+H]^+$ 198.0915; $C_{13}H_{12}NO^+$ requires 198.0919. Data are consistent with literature values.[6]

1-(1H-imidazol-1-yl)-2-phenylprop-2-en-1-one (**S21**)

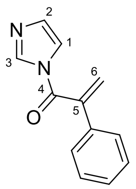

Synthesised from atropic acid (1.31 g, 8.81 mmol) and imidazole (500 mg, 7.34 mmol) using **General Procedure A1**. The crude residue was purified by flash column chromatography (10→20%

5. M. Morita, L. Drouin, R. Motoki, Y. Kimura, I. Fujimori, M. Kanai and M. Shibasaki, *J. Am. Chem. Soc.* **2009**, *131*, 3858–3859.
6. M. Simic, G. Tasic, P. Jovanovic, M. Petkovic and V. Savic, *Org. Biol. Chem.* **2018**, *16*, 2125-2133.

EtOAc/petroleum ether) to afford the titled product as a yellow oil (227 mg, 1.15 mmol, 16%); **δ$_H$** (500 MHz, $CDCl_3$) 7.93 (1H, s, H3), 7.37 (1H, s, H2), 7.32-7.26 (5H, m, 5 × Ph*H*), 6.97 (1H, s, H1), 6.04 (1H, s, H6'), 5.72 (1H, s, H6''); **δ$_C$** (125 MHz, $CDCl_3$) 117.06, 122.26, 126.40, 129.10, 129.40, 131.11, 134.71, 137.74, 142.72, 165.96; **IR**(thin film) ν_{max}/cm^{-1} 1712, 1642; **HRMS** *m/z* (ESI$^+$) found [M+H]$^+$ 199.0874; $C_{12}H_{11}N_2O^+$ requires 199.0871.

6-phenyl-6,7-dihydro-5H-pyrrolo[1,2-a]imidazol-5-one (major, **22A**) and 6-phenyl-6,7-dihydro-5H-pyrrolo[1,2-c]imidazol-5-one (minor, **22B**)

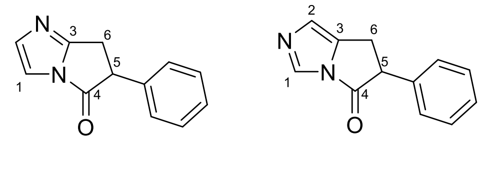

1.26 major, **22A** 1 minor, **22B**

Synthesised from compound **S21** (110.2 mg, 0.56 mmol) using **General Procedure B1**. The crude residue was purified by flash column chromatography (20% EtOAc/petroleum ether) to give an inseparable mixture of **22A** and **22B** (1.26:1) as a pale yellow oil (23.5 mg, 0.12 mmol, overall 21%). Data for the major isomer: **δ$_H$** (500 MHz, $CDCl_3$) 7.36-7.14 (7H, m, 5 × Ph*H*, H1 and H2), 4.41 (1H, dt, *J* = 8.7 and 4.6 Hz, H5), 3.60 (1H, dd, *J* = 17.8 and 8.7 Hz, H6'), 3.17 (1H, dd, *J* = 17.8 and 4.2 Hz, H6''); **δ$_C$** (125 MHz, $CDCl_3$) 30.60, 53.35, 111.37, 127.73, 128.36, 129.42, 136.70, 137.44, 157.18, 170.89. Data for the minor isomer: **δ$_H$** (500 MHz, $CDCl_3$); 7.91 (1H, s, H1), 6.81 (1H, s, H2), 4.41 (1H, dt, *J* = 8.7 and 4.6 Hz, H5), 3.53 (1H, ddd, *J* = 16.9, 8.7 and 1.4 Hz, H6'), 3.10 (1H, ddd, *J* = 16.9, 4.6, and 1.4 Hz, H6''); **δ$_C$** (125 MHz, $CDCl_3$) 27.41, 54.41, 122.67, 127.77, 128.34, 129.04, 129.42, 135.54, 136.82, 169.81.

2-(1-(2-phenylacryloyl)-1H-indol-3-yl)acetonitrile (**S22**)

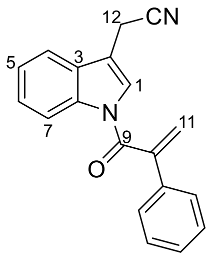

Synthesised from atropic acid (367 mg, 2.48 mmol) and 2-(1H-indol-3-yl)acetonitrile (352 mg, 2.25 mmol) using **General Procedure A3**. The resulting crude material was purified by column chromatography (10% AcOEt/pentane) to give the titled compound as a white solid (351 mg, 1.23 mmol, 54%); **m.p.** 94 °C; **δ$_H$** (400 MHz, $CDCl_3$): 8.57 (1H, dt, *J* = 8.2, 0.9 Hz, H7), 7.54 (1H, ddd, *J* = 7.8, 1.3, 0.8 Hz, H4), 7.51 – 7.44 (3H, m, H5, H6, Ph*H*), 7.43 – 7.32 (5H, m, H1, Ph*H*), 6.11 (1H, s, H11), 5.70 (1H, s, H11), 3.71 (2H, d, *J* = 1.3 Hz, H12); **δ$_C$** (100 MHz, $CDCl_3$): 168.32, 144.10, 136.07, 135.12, 129.42, 129.27, 129.11, 126.34, 126.21, 125.23, 124.68, 118.96, 118.40, 117.24, 116.79, 111.63, 14.52; **IR** (thin film) ν_{max}/cm^{-1}: 1678, 1372, 1224; **HRMS** *m/z* (ESI$^+$) found [M+H]$^+$ 287.1180; $C_{19}H_{15}N_2O^+$ requires 287.1179.

2-(3-oxo-2-phenyl-2,3-dihydro-1H-pyrrolo[1,2-a]indol-9-yl)acetonitrile (**23**)

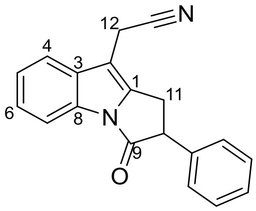

Synthesised from compound **S22** (85.9 mg, 0.3 mmol) using **General Procedure B1**. The resulting crude material was purified by column chromatography (20% AcOEt/pentane) to give the titled compound as a yellow solid (79.4 mg, 0.83 mmol, 92%); **m.p.** 106 °C; **δ_H** (400 MHz, $CDCl_3$): 8.20 – 8.06 (1H, m, H7), 7.55 – 7.46 (1H, m, H4), 7.44 – 7.22 (7H, m, Ph*H*, H5, H6), 4.43 (1H, dd, *J* = 9.1, 4.8 Hz, H10), 3.90 – 3.73 (3H, m, H11, H12), 3.37 (ddt, *J* = 17.4, 4.6, 1.2 Hz, H11); **δ_C** (100 MHz, $CDCl_3$): 171.54, 139.55, 137.95, 133.57, 130.52, 129.34, 128.10, 127.81, 124.78, 124.63, 118.19, 116.84, 114.31, 102.07, 52.54, 28.81, 13.48; **IR** (thin film) ν_{max}/cm^{-1}: 1735, 1629, 1456, 1359; **HRMS** *m/z* (ESI$^+$) found [M+H]$^+$ 287.1180; $C_{19}H_{15}N_2O^+$ requires 287.1179.

methyl 1-(2-phenylacryloyl)-1H-indole-3-carboxylate (**S23**)

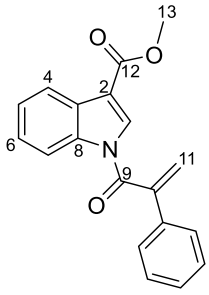

Synthesised from atropic acid (0.5 g, 3.375 mmol) and methyl 1H-indole-3-carboxylate (394 mg, 2.25 mmol) using **General Procedure A1**. The resulting crude material was purified by column chromatography (7.5% AcOEt/pentane) to give the titled compound as a white solid (449 mg, 1.46 mmol, 65%); **m.p.** 85-86 °C; **δ_H** (400 MHz, $CDCl_3$): 8.59 – 8.52 (1H, m, H7), 8.20 – 8.13 (1H, m, H4), 8.02 (1H, s, H1), 7.53 – 7.33 (7H, m, H5, H6, Ph*H*), 6.16 (1H, s, H11), 5.73 (1H, s, H11), 3.89 (3H, s, H13); **δ_C** (100 MHz, $CDCl_3$): 168.77, 164.41, 143.63, 136.09, 134.92, 132.93, 129.52, 129.29, 127.98, 126.25, 126.14, 125.39, 121.86, 119.69, 116.70, 114.04, 51.70; **IR** (thin film) ν_{max}/cm^{-1}: 1682, 1717, 1701, 1622, 1192; **HRMS** *m/z* (ESI$^+$) found [M+H]$^+$ 306.1126; $C_{19}H_{16}NO_3^+$ requires 306.1125.

methyl 3-oxo-2-phenyl-2,3-dihydro-1H-pyrrolo[1,2-a]indole-9-carboxylate (**24**)

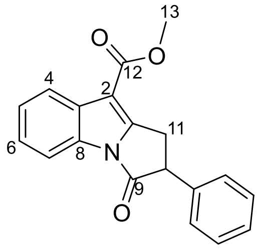

Synthesised from compound **S23** (91.6 mg, 0.3 mmol) using **General Procedure B1**. The resulting crude material was purified by column chromatography (10% AcOEt/pentane) to give the tiled compound as a pale orange solid (77.1 mg, 0.25 mmol, 84%); **m.p.** 135-137 °C; **δ_H** (400 MHz, $CDCl_3$): 8.13 (1H, ddd, *J* = 7.7, 1.4, 0.7 Hz, H7), 8.11 – 8.06 (1H, ddd, *J* = 7.8, 1.4, 0.7 Hz, H4), 7.45 – 7.27 (7H, m, Ph*H*, H5, H6), 4.42 (1H, dd, *J* = 9.0, 4.4 Hz, H10), 4.03 – 3.92 (4H, m, H11, H13), 3.55 (1H, dd, *J* = 19.2, 4.4 Hz, H11); **δ_C** (100 MHz, $CDCl_3$): 172.39, 164.76, 150.55, 137.51, 132.16, 130.66, 129.37, 128.16, 127.78, 125.57, 124.68, 121.79, 113.87, 106.30, 51.93, 51.56, 31.17; **IR** (thin film) ν_{max}/cm^{-1}: 1742, 1716, 1591, 1457, 1158; **HRMS** *m/z* (ESI^+) found $[M+H]^+$ 306.1126; $C_{19}H_{16}NO_3^+$ requires 306.1125.

2-(1-(2-methoxyacryloyl)-1H-indol-3-yl)acetonitrile (**S24**)

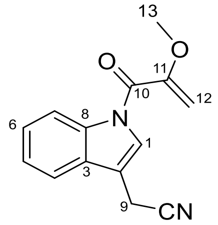

Synthesised from 2-Methoxyacrylic acid[7] (210 mg, 2.06 mmol) and 2-(1H-indol-3-yl)acetonitrile (277 mg, 1.10 mmol) using **General Procedure A3**. The resulting crude material was purified by column chromatography (10% Et_2O/petroleum ether) to afford the titled compound as a pale yellow oil (148 mg, 0.616 mmol, 32%); **δ_H** (400 MHz, $CDCl_3$): 8.40 (1H, d, *J* = 7.8 Hz, H7), 7.67 (1H, s, H1), 7.51 (1H, d, *J* = 7.8 Hz, H4), 7.42 (1H, td, *J* = 7.5, 1.2 Hz, H6), 7.35 (1H, td, *J* = 7.5, 1.2 Hz, H5), 5.12 (1H, d, *J* =3.3 Hz, H12), 4.77 (1H, d, *J* = 3.3 Hz, H12), 3.80 (3H, s, H13), 3.77 (2H, s, H9); **δ_C** (100 MHz, $CDCl_3$): 14.51, 55.88, 93.45, 111.32, 117.00, 117.07, 118.19, 124.52, 125.32, 126.05, 128.75, 136.42, 155.42, 162.95; **IR** (thin film) ν_{max}/cm^{-1}: 2265, 1732, 1628, 1578; **HRMS** *m/z* (ESI^+) found $[M+H]^+$ 241.0977; $C_{14}H_{13}N_2O_2^+$ requires 241.0977.

2-(2-methoxy-3-oxo-2,3-dihydro-1H-pyrrolo[1,2-a]indol-8-yl)acetonitrile (**25**)

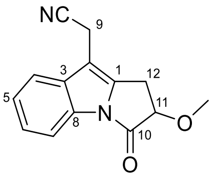

Synthesised from compound **S24** (50.0 mg, 0.21 mmol) using **General Procedure B1**. The crude material was purified by flash column chromatography (10% Et_2O/petroleum ether) to afford the desired

7. R. B. Bates and S. Caldera, *J. Org. Chem.* **1993**, *58*, 6920 – 6921.

compound as a pale yellow oil (37.0 mg, 0.15 mmol, 74%); **δ$_H$** (400 MHz, $CDCl_3$): 8.31 (1H, d, *J* = 7.8 Hz, H7), 7.54 (1H, d, *J* = 7.7 Hz, H4), 7.43 (1H, td, *J* = 7.7 and 1.1 Hz, H6), 7.32 (1H, td, *J* = 7.7 and 1.1 Hz, H5), 4.51 (1H, dd, *J* = 5.0, 1.6 Hz, H11), 3.81 (3H, s, C*H$_3$*), 3.78 (2H, d, *J* = 1.3 Hz, C*H$_2$*), 3.26 (1H, dd, *J* = 17.8, 5.0 Hz, H12), 3.14 (1H, dd, *J* = 17.8, 1.6 Hz, H12); **δ$_C$** (100 MHz, $CDCl_3$): 14.49, 35.07, 55.94, 93.08, 111.53, 117.07, 117.13, 117.91, 124.49, 125.86, 128.83, 136.18, 136.78, 163.69; **IR** (thin film) ν_{max}/cm^{-1}: 2268, 1724, 1627, 1567; **HRMS** *m/z* (ESI$^+$) found $[M+H]^+$ 241.0977; $C_{14}H_{13}N_2O_2^+$ requires 241.0977.

2-(1-(2-(4-methoxyphenyl)acryloyl)-1H-indol-3-yl)acetonitrile (**S25**)

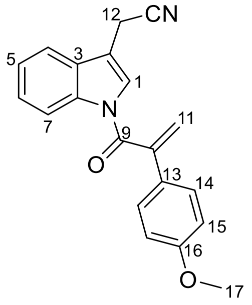

Synthesised from 2-(4-methoxyphenyl)acrylic acid[8] (441 mg, 2.48 mmol) and 2-(1H-indol-3-yl)acetonitrile (352 mg, 2.25 mmol) using **General Procedure A3**. The resulting crude material was purified by column chromatography (20% AcOEt/pentane) followed by recrystallization from minimum amount of hot isopropanol to give the titled compound as a white solid (327 mg, 1.04 mmol, 46%); **m.p.** 124 °C; **δ$_H$** (400 MHz, $CDCl_3$): 8.56 (1H, dt, *J* = 8.3, 0.9 Hz, H7), 7.54 (1H, ddd, *J* = 7.8, 1.3, 0.7 Hz, H4), 7.47 (1H, ddd, *J* = 8.4, 7.2, 1.3 Hz, H6), 7.43 – 7.35 (4H, m, H5, H1, H14), 6.99 – 6.84 (2H, m, H15), 5.99 (1H, s, H11), 5.57 (1H, s, H11), 3.81 (3H, s, H17), 3.71 (2H, d, *J* = 1.3 Hz, H12); **δ$_C$** (100 MHz, $CDCl_3$): 168.64, 160.52, 143.46, 136.03, 129.10, 127.59, 127.53, 126.27, 125.33, 124.61, 118.38, 117.21, 116.84, 116.73, 114.63, 111.47, 55.49, 14.51; **IR** (thin film) ν_{max}/cm^{-1}: 1683, 1606, 1514, 1500, 1372; **HRMS** *m/z* (ESI$^+$) found $[M+H]^+$ 317.1285; $C_{20}H_{17}N_2O_2^+$ requires 317.1285.

2-(2-(4-methoxyphenyl)-3-oxo-2,3-dihydro-1H-pyrrolo[1,2-a]indol-9-yl)acetonitrile (**26**)

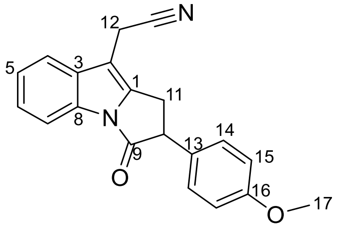

Synthesised from compound **S25** (94.9 mg, 0.3 mmol) using **General Procedure B1**. The resulting crude material was purified by column chromatography (30% AcOEt/pentane) to give the tiled compound as a yellow solid (84.1 mg, 0.28 mmol, 92%); **m.p.** 127 °C; **δ$_H$** (400 MHz, $CDCl_3$): 8.15 – 8.03 (1H, m, H7), 7.55 – 7.45 (1H, m, H4), 7.43 – 7.32 (2H, m, H5, H6), 7.25 – 7.16 (2H, m, H14), 6.97 – 6.82 (2H, m, H15), 4.36 (1H, dd, *J* = 9.3, 4.9 Hz, H10), 3.84 – 3.74 (6H, m, H11, H12, H17), 3.31 (1H, ddt, *J* = 17.8, 5.3, 1.3 Hz, H11); **δ$_C$** (100 MHz, $CDCl_3$): 171.86, 159.39, 139.60, 133.53, 130.51, 129.92, 128.89, 124.71, 124.56, 118.15, 116.86,

8. N. K. Rana, V. K. Singh, *Org. Lett.* **2011**, *13*, 6520-6523.

114.71, 114.27, 101.98, 55.47, 51.80, 28.84, 13.44; **IR** (thin film) ν_{max}/cm^{-1}: 1724, 1516, 1455; **HRMS** *m/z* (ESI$^+$) found [M+H]$^+$ 317.1285; $C_{20}H_{17}N_2O_2^+$ requires 317.1285.

1-(2-(4-(*tert*-butyl)phenyl)acryloyl)-1H-indole-5-carbonitrile (**S26**)

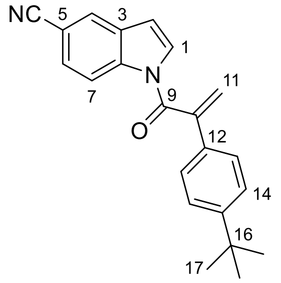

Synthesised from 2-(4-(*tert*-butyl)phenyl)acrylic acid[6] (306.4 mg, 1.5 mmol) and 1H-indole-5-carbonitrile (142 mg, 1.0 mmol) using **General Procedure A2**. The resulting crude material was purified by column chromatography (15% Et_2O/pentane) to give the titled compound as a white solid (240.3 mg, 0.73 mmol, 73%); **m.p.** 103-104 °C; **δ_H** (400 MHz, $CDCl_3$): 8.64 (1H, dt, *J* = 8.6, 0.8 Hz, H7), 7.90 (1H, dd, *J* = 1.7, 0.7 Hz, H4), 7.65 (1H, dd, *J* = 8.5, 1.7 Hz, H6), 7.44 (1H, d, *J* = 3.8 Hz, H1), 7.43 – 7.35 (4H, m, H13, H14), 6.59 (1H, dd, *J* = 3.8, 0.8 Hz, H2), 6.09 (1H, s, H11), 5.68 (1H, s, H11), 1.31 (9H, s, H17); **δ_C** (100 MHz, $CDCl_3$): 168.84, 152.87, 143.80, 137.53, 132.13, 131.09, 129.29, 128.40, 126.26, 125.94, 125.74, 119.62, 118.75, 117.59, 108.52, 107.71, 34.88, 31.31; **IR** (thin film) ν_{max}/cm^{-1}: 2227, 1702, 1460, 1365, 1337; **HRMS** *m/z* (ESI$^+$) found [M+H]$^+$ 329.1649; $C_{22}H_{21}N_2O^+$ requires 329.1648.

2-(4-(*tert*-butyl)phenyl)-3-oxo-2,3-dihydro-1H-pyrrolo[1,2-a]indole-7-carbonitrile (**27**)

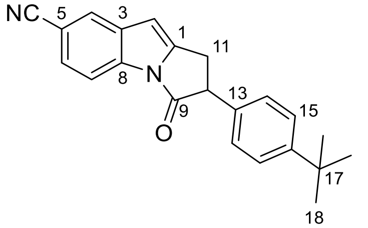

Synthesised from compound **S26** (98.0 mg, 0.3 mmol) using **General Procedure B1**. The resulting crude material was purified by column chromatography (10% AcOEt/pentane) to give the tiled compound as an off-white solid (89.0 mg, 0.27 mmol, 91%); **m.p.** 149 °C; **δ_H** (400 MHz, $CDCl_3$): 8.15 (1H, dt, *J* = 8.3, 0.8 Hz, H7), 7.87 (1H, dd, *J* = 1.6, 0.7 Hz, H4), 7.54 (1H, dd, *J* = 8.3, 1.5 Hz, H6), 7.46 – 7.34 (2H, m, H15), 7.26 – 7.18 (2H, m, H14), 6.43 (1H, td, *J* = 1.5, 0.7 Hz, H2), 4.41 (1H, dd, *J* = 9.2, 4.9 Hz, H10), 3.74 (1H, ddd, *J* = 17.7, 9.2, 1.5 Hz, H11), 3.34 (1H, ddd, *J* = 17.7, 4.9, 1.6 Hz, H11), 1.31 (9H, s, H18); **δ_C** (100 MHz, $CDCl_3$): 172.19, 151.21, 144.24, 135.46, 134.52, 132.49, 127.41, 126.91, 126.32, 125.52, 119.74, 114.60, 107.75, 100.42, 52.27, 34.70, 31.40, 29.60; **IR** (thin film) ν_{max}/cm^{-1}: 2221, 1737, 1438, 1384, 1348; **HRMS** *m/z* (ESI$^+$) found [M+H]$^+$ 329.1649; $C_{22}H_{21}N_2O^+$ requires 329.1648.

1-(2-(4-(trifluoromethyl)phenyl)acryloyl)-1H-indole-5-carbonitrile (**S27**)

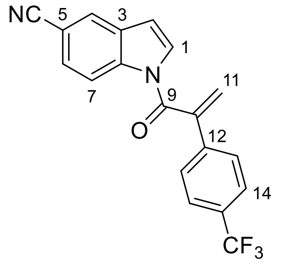

Synthesised from 2-(4-(trifluoromethyl)phenyl)acrylic acid[6] (324.3 mg, 1.50 mmol) and 1H-indole-5-carbonitrile (142 mg, 1.0 mmol) using **General Procedure A2**. The resulting crude material was purified by column chromatography (20% AcOEt/pentane) to give the titled compound as a yellow oil (60.4 mg, 0.18 mmol, 18%); **δ_H** (400 MHz, $CDCl_3$): 8.63 (1H, dt, *J* = 8.6, 0.8 Hz, H7), 7.91 (1H, dd, *J* = 1.6, 0.7 Hz, H4), 7.70 – 7.55 (5H, m, H6, H13, H14), 7.40 (1H, d, *J* = 3.8 Hz, H1), 6.64 (1H, dd, *J* = 3.8, 0.8 Hz, H2), 6.24 (1H, s, H11), 5.88 (1H, s, H11); **δ_C** (100 MHz, $CDCl_3$): 167.75, 142.82, 138.46, 137.54, 131.11, 128.77, 128.66, 126.73, 126.33 (q, *J* = 3.8 Hz), 125.89, 122.16, 119.46, 117.61, 109.10, 108.08; **δ_F** (376 MHz, $CDCl_3$): -62.87; **IR** (thin film) ν_{max}/cm^{-1}: 2227, 1699, 1461, 1325; **HRMS** *m/z* (ESI^+) found $[M-H]^-$ 339.0750; $C_{19}H_{10}N_2OF_3^-$ requires 339.0751.

3-oxo-2-(4-(trifluoromethyl)phenyl)-2,3-dihydro-1H-pyrrolo[1,2-a]indole-7-carbonitrile (**28**)

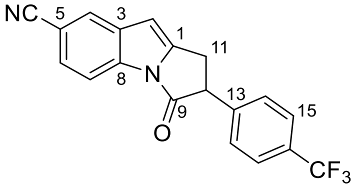

Synthesised from compound **S27** (34.0 mg, 0.1 mmol) using **General Procedure B1**. The resulting crude material was purified by column chromatography (20% AcOEt/pentane) to give the tiled compound as an off white (23.2 mg, 0.07 mmol, 68%); **m.p.** 160 °C; **δ_H** (400 MHz, $CDCl_3$): 8.15 (1H, dd, *J* = 8.4, 0.8 Hz, H7), 7.92 – 7.84 (1H, m, H4), 7.65 (2H, d, *J* = 8.1 Hz, H15), 7.56 (1H, dd, *J* = 8.4, 1.5 Hz, H6), 7.44 (2H, d, *J* = 8.1 Hz, H14), 6.46 (1H, q, *J* = 1.3 Hz, H12), 4.51 (1H, dd, *J* = 9.3, 5.1 Hz, H12), 3.80 (1H, ddd, *J* = 17.7, 9.3, 1.5 Hz, H11), 3.34 (1H, ddd, *J* = 17.7, 5.1, 1.7 Hz, H11); **δ_C** (100 MHz, $CDCl_3$): 170.99, 143.41, 141.43, 135.47, 132.47, 130.76, 130.44, 128.31, 127.17, 126.36 (q, *J* = 3.8 Hz), 125.66, 119.57, 114.66, 108.09, 100.93, 52.35, 29.37; **δ_F** (376 MHz, $CDCl_3$): -62.73; **IR** (thin film) ν_{max}/cm^{-1}: 2225, 1744, 1463, 1325; **HRMS** *m/z* (ESI^+) found $[M+H]^+$ 341.0896; $C_{19}H_{12}N_2OF_3^-$ requires 341.0896.

(E/Z)-2-phenylbut-2-enoic acid (**S28**)

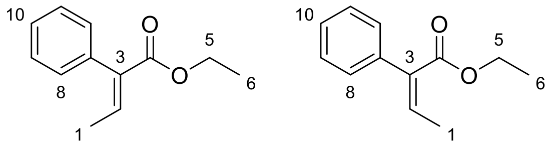

E : *Z*
10 : 1

To a suspension of ethyltriphenylphosphonium bromide (1.78 g, 10 mmol, 1 equiv.) in anhydrous THF (20 mL) cooled to –78 °C, KHMDS (10 mL, 1M in THF, 10 mmol, 1 equiv.) was added dropwise and the resulting mixture was stirred at –78 °C for 15 minutes. The reaction was then allowed to worm to room temperature and stirred for an additional 1 h when it was cooled back to –78 °C. A solution of ethyl 2-oxo-2-phenylacetate (3.71 g, 10 mmol, 1 equiv.) in anhydrous THF (5 mL) was added dropwise and the reaction was further stirred for 1 h at –78 °C, when the reaction was allowed to warm to room temperature over 16 h. The reaction was then quenched with 1M HCl, extracted with CH_2Cl_2, dried of $MgSO_4$ and evaporated. The resulting crude oil was purified by column chromatography (5% Et_2O/pentane) to give the titled compound as a clear liquid (1.05 g, 5.5 mmol, 55%); **δ_H** (400 MHz, $CDCl_3$): 7.41 – 7.28 (6H, m, Ph*H*), 7.21 – 7.14 (5H, m, $H2^{E}$, Ph*H*), 6.27 (1H, q, J = 7.2 Hz, $H2^{z}$), 4.31 (2H, q, J = 7.1 Hz, $H5^{Z}$), 4.21 (2H, q, J = 7.1 Hz, $H5^{E}$), 2.05 (3H, d, J = 7.2 Hz, $H1^{Z}$), 1.75 (3H, d, J = 7.2 Hz, $H1^{E}$), 1.33 (3H, t, J = 7.1 Hz, $H6^{Z}$), 1.26 (t, J = 7.1 Hz, $H6^{E}$); **δ_C** (100 MHz, $CDCl_3$): 167.28, 139.75, 135.28, 135.12, 134.46, 129.92, 128.36, 128.05, 127.54, 127.43, 127.22, 60.84, 16.07, 15.57, 14.40, 14.36. Data in accordance to literature.[9]

(E/Z)-2-phenylbut-2-enoic acid (**S29**)

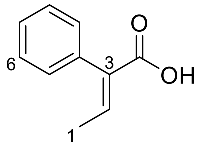

A solution of **S25** (920 mg, 4.84 mmol, 1 equiv.), $LiOH \cdot H_2O$ (1.02 g, 24.2 mmol, 5 equiv.) in mixture of THF (9.7 mL) and H_2O (9.7 mL) was heated to 80 °C for 24 h. The reaction was quenched with 1M HCl, extracted with Et_2O, dried of $MgSO_4$ and evaporated. The resulting oil was triturated with pentane to give an off-white solid of the titled compound (697 mg, 4.21 mmol, 89%); **δ_H** (400 MHz, $CDCl_3$): 7.42 – 7.28 (4H, m, H2, Ph*H*), 7.22 – 7.16 (2H, m, Ph*H*), 1.78 (3H, d, J = 7.2 Hz, H1). **δ_C** (100 MHz, $CDCl_3$): 172.54, 142.93, 134.55, 134.29, 129.99, 129.95, 128.29, 128.21, 128.08, 127.77, 15.92. Data in accordance with literature.[10]

9. F. N. Palmer, F. Lach, C. Poriel, A. G. Pepper, M. C. Bagley, A. M. Z. Slawin and C. J. Moody, *Org. Biol. Chem.* **2005**, *3*, 3805 – 3811.
10. T. Fujihara, T. Xu, K. Semba, J. Terao and Y. Tsuji, *Angew. Chem. Int. Ed.* **2011**, *50*, 523-527.

(*E*/*Z*)-2-(1-(2-phenylbut-2-enoyl)-1H-indol-3-yl)acetonitrile (**S30**)

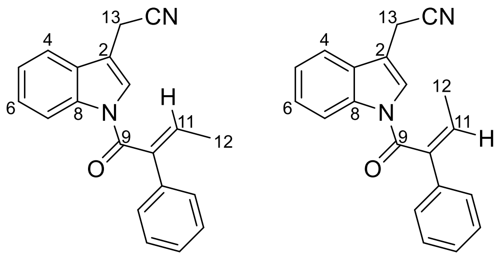

A : B

1 : 2.4

Synthesised from (*E*)-2-phenylbut-2-enoic acid (401 mg, 2.47 mmol) and 2-(1H-indol-3-yl)acetonitrile (352 mg, 2.25 mmol) using **General Procedure A3**. The resulting crude material was purified by column chromatography (10% AcOEt/pentane) to give the titled compound as a yellow oil (252 mg, 0.83 mmol, 37%); **δ_H** (400 MHz, $CDCl_3$): 8.48 (1H, dt, *J* = 8.3, 0.9 Hz, H7^A), 7.58 – 7.28 (m, 15H, H4^A, H5^A, H6^A, Ph*H*^{A}, H4^B, H5^B, H6^B, H7^B, Ph*H*^{B}), 6.48 (2H, m, H1^A, H1^B), 3.71 (2H, d, *J* = 1.3 Hz, H12^B), 3.70 (2H, d, *J* = 1.3 Hz, H13^A), 2.00 (3H, d, *J* = 7.2 Hz, H12^A), 1.88 (3H, d, *J* = 7.2 Hz, H12^B); **δ_C** (100 MHz, $CDCl_3$): 168.82, 137.68, 136.34, 135.74, 135.30, 134.55, 129.20, 129.15, 129.04, 128.97, 128.94, 128.81, 128.47, 128.43, 128.39, 126.32, 126.00, 125.55, 125.36, 124.71, 124.64, 124.27, 118.36, 118.19, 117.06, 116.94, 116.84, 112.01, 110.79, 15.86, 15.30, 14.53, 14.50; **IR** (thin film) ν_{max}/cm^{-1}: 1678, 1372, 1224; **HRMS** *m/z* (ESI^+) found $[M+H]^+$ 301.1336; $C_{20}H_{17}N_2O^+$ requires 301.1335.

2-(1-methyl-3-oxo-2-phenyl-2,3-dihydro-1H-pyrrolo[1,2-a]indol-9-yl)acetonitrile (**29**)

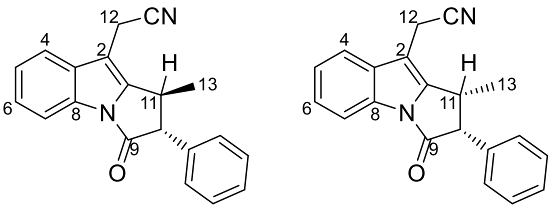

A : B

1.2 : 1

Synthesised from compound **S30** (90.1 mg, 0.3 mmol) using **General Procedure B1**. The resulting crude material was purified by column chromatography (2.5% Et_2O/pentane) to give the tiled compound as a pale yellow solid (78.9 mg, 0.26 mmol, 88%); **m.p.** 112-114 °C; **δ_H** (400 MHz, $CDCl_3$, mixture of isomers): 8.21 – 8.11 (1H, m, H7^B), 8.15 – 8.07 (1H, m, H7^A), 7.61 – 7.51 (2H, m, H4^A, H4^B), 7.44 – 7.33 (10H, m, Ph*H*^{A}, H5^A, H6^A, Ph*H*^{B}, H5^B, H6^B), 7.29 – 7.22 (2H, m, Ph*H*^{A}, Ph*H*^{B}), 7.22 – 7.15 (2H, m, Ph*H*^{A}, Ph*H*^{B}), 4.64 (1H, d, *J* = 8.6 Hz, H10^B), 4.05 – 3.93 (1H, m, H11^B), 3.91 (1H, d, *J* = 4.7 Hz, H10^A), 3.88 – 3.71 (4H, m, H12^A, H12^B), 3.72 – 3.60 (1H, m, H11^A), 1.65 (3H, d, *J* = 7.0 Hz, H13^A), 1.04 (3H, d, *J* = 7.4 Hz, H13^B); **δ_C** (100 MHz, $CDCl_3$): 171.46, 171.07, 144.88, 144.06, 137.24, 134.48, 133.66, 133.51, 130.24, 130.20, 129.84, 129.35, 128.92, 128.16, 128.06, 127.99, 124.81, 124.80, 124.76, 124.74, 118.37, 118.30, 116.91, 114.44, 114.38, 102.09, 101.93, 62.09, 57.45, 37.62, 32.47, 19.38, 17.39, 13.17, 13.10; **IR** (thin film) ν_{max}/cm^{-1}: 1724, 1457, 1395; **HRMS** *m/z* (ESI^+) found $[M+H]^+$ 301.1336; $C_{20}H_{17}N_2O^+$ requires 301.1335.

2-phenyl-1-(3-(thiazol-2-yl)-1H-indol-1-yl)prop-2-en-1-one (**S31**)

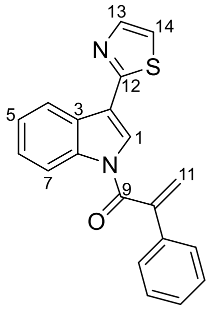

Synthesised from atropic acid (183.4 mg, 1.24 mmol) and camalexin (225 mg, 1.12 mmol)[11] using **General Procedure A3**. The resulting crude material was purified by column chromatography (10% AcOEt/pentane) to give the titled compound as a white solid (276.5 mg, 0.83 mmol, 74%); **m.p.** 139-140 °C; **δ_H** (400 MHz, $CDCl_3$): 8.67 – 8.57 (1H, m, H7), 8.33 – 8.24 (1H, m, H4), 7.95 (1H, s, H1), 7.88 (1H, d, *J* = 3.3 Hz, H13), 7.54 – 7.44 (4H, m, H5, H6, Ph*H*) 7.42 – 7.31 (4H, m, H14, Ph*H*), 6.16 (1H, s, H11), 5.75 (1H, s, H11); **δ_C** (100 MHz, $CDCl_3$): ^{13}C NMR (101 MHz, $CDCl_3$) δ 168.61, 160.79, 143.90, 143.37, 136.30, 135.08, 129.42, 129.25, 127.90, 126.30, 126.28, 125.94, 125.19, 121.16, 119.23, 117.90, 117.52, 116.99; **IR** (thin film) ν_{max}/cm^{-1}: 1686, 1616, 1472, 1374, 1194; **HRMS** *m/z* (ESI^+) found $[M+H]^+$ 331.0899; $C_{20}H_{15}N_2SO^+$ requires 331.0900.

2-phenyl-9-(thiazol-2-yl)-1,2-dihydro-3H-pyrrolo[1,2-a]indol-3-one (**30**)

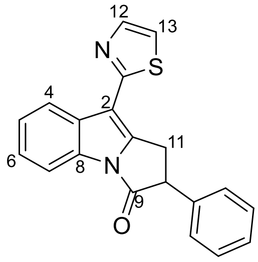

Synthesised from compound **S31** (99.1 mg, 0.3 mmol) using **General Procedure B1**. The resulting crude material was purified by column chromatography (10% AcOEt/pentane) to give the tiled compound as an off-white solid (49.5 mg, 0.15 mmol, 50%); **m.p.** 157-160 °C; **δ_H** (400 MHz, $CDCl_3$): 8.39 – 8.32 (1H, m, H4), 8.19 – 8.12 (1H, m, H7), 7.93 (1H, d, *J* = 3.3 Hz, H12), 7.51 – 7.29 (8H, m, Ph*H*, H5, H6, H13), 4.50 (1H, dd, *J* = 9.1, 4.5 Hz, H10), 3.99 (1H, dd, *J* = 18.5, 9.1 Hz, H11), 3.55 (1H, dd, *J* = 18.5, 4.5 Hz, H11); **δ_C** (100 MHz, $CDCl_3$): 171.99, 161.14, 143.23, 142.22, 137.91, 132.08, 130.87, 129.36, 128.11, 127.87, 125.39, 124.74, 121.49, 117.22, 114.06, 109.89, 52.55, 31.10; **IR** (thin film) ν_{max}/cm^{-1}: 1728, 1597, 1492, 1350; **HRMS** *m/z* (ESI^+) found $[M+H]^+$ 331.0900; $C_{20}H_{15}N_2OS^+$ requires 331.0900.

11. M. Pedras, C. Soledade and A. Abdoli, *Bioorg. Med. Chem.* **2018**, *26*, 4461-4469.

N-(2-(5-methoxy-1-(2-phenylacryloyl)-1H-indol-3-yl)ethyl)acetamide (**S32**)

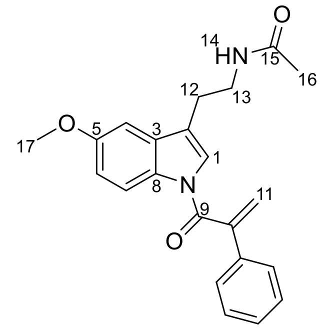

To a solution of atropic acid (0.5 g, 3.37 mmol, 1.5 equiv.) and oxalyl chloride (0.35 mL, 2.7 mmol, 1.2 equiv.) in anhydrous CH_2Cl_2 (6.8 mL), was added one drop of anhydrous DMF. The reaction mixture was stirred at room temperature for 3 h. Once the reaction completed, anhydrous toluene (14.5 mL), tetrabutylammonium bromide (363 mg, 1.13 mmol, 0.5 equiv.), freshly ground KOH (455 mg, 8.11 mmol, 3.6 equiv.) and melatonin (523 mg, 2.25 mmol, 1 equiv.) were added to the reaction mixture. The reaction was heated to 40 °C and further stirred for 16 h. The resulting mixture was then cooled to room temperature, concentrated and purified by column chromatography (25% MeCN/pentane) to give the titled compound as a viscous oil (178 mg, 0.50 mmol, 22%); **δ_H** (400 MHz, $CDCl_3$): 8.47 – 8.40 (1H, m, H7), 7.49 – 7.45 (2H, m, Ph*H*), 7.41 – 7.31 (3H, m, Ph*H*), 7.08 (1H, d, *J* = 1.1 Hz, H4), 7.06 – 6.97 (2H, m, H4, H6), 6.05 (1H, s, H11), 5.66 (1H, s, H11), 5.40 (1H, s, H14), 3.88 (3H, s, H17), 3.49 (2H, q, *J* = 6.6 Hz, H13), 2.80 (2H, td, *J* = 6.9, 1.1 Hz, H12), 1.85 (3H, s, H16); **δ_C** (100 MHz, $CDCl_3$): 170.14, 167.88, 157.15, 144.46, 135.54, 132.08, 130.68, 129.19, 129.15, 126.18, 124.81, 119.60, 118.29, 117.88, 113.68, 102.18, 55.94, 38.90, 25.25, 23.40; **IR** (thin film) ν_{max}/cm^{-1}: 3290, 1679, 1551, 1475, 1384, 1204; **HRMS** *m/z* (ESI^+) found $[M+H]^+$ 363.1704; $C_{22}H_{23}N_2O_3^+$ requires 363.1703.

N-(2-(7-methoxy-3-oxo-2-phenyl-2,3-dihydro-1H-pyrrolo[1,2-a]indol-9-yl)ethyl)acetamide (**31**)

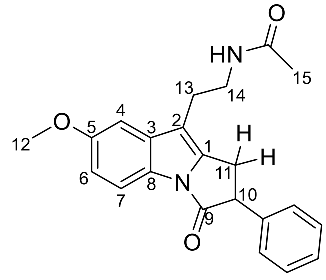

Synthesised from compound **S32** (108.7 mg, 0.3 mmol) using **General Procedure B1**. The resulting crude material was purified by column chromatography (50% MeCN/toluene) to give the tiled compound as a yellow solid (54.7 mg, 0.15 mmol, 50%); **m.p.** 151-154 °C; **δ_H** (400 MHz, $CDCl_3$): 7.88 (1H, dd, *J* = 8.7, 0.5 Hz, H7), 7.34 – 7.14 (5H, m, *Ph*H), 6.92 (1H, d, *J* = 2.3 Hz, H4), 6.83 (1H, dd, *J* = 8.8, 2.4 Hz, H6), 5.60 (1H, s, N*H*), 4.23 (1H, dd, *J* = 9.2, 4.6 Hz, H10), 3.81 (3H, s, H12), 3.59 – 3.40 (3H, m, H11, H14), 3.08 (1H, dd, *J* = 17.3, 4.6 Hz, H11), 2.81 (2H, td, *J* = 6.9, 1.8 Hz, H13), 1.85 (3H, s, H15); **δ_C** (100 MHz, $CDCl_3$): 171.13, 170.24, 157.35, 139.42, 138.66, 136.69, 129.25, 127.89, 127.71, 125.46, 114.77, 111.91, 110.63, 102.33, 55.96, 52.64, 39.17, 29.03, 24.48, 23.48.; **IR** (thin film) ν_{max}/cm^{-1}: 3271, 1728, 1634, 1473, 1232; **HRMS** *m/z* (ESI^+) found $[M+H]^+$ 363.1704; $C_{22}H_{23}N_2O_3^+$ requires 363.1703.

1-(1H-imidazol-1-yl)-2-phenylprop-2-en-1-one (**S33**)

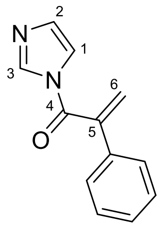

Synthesised from atropic acid (1.31 g, 8.81 mmol) and imidazole (500 mg, 7.34 mmol) using **General Procedure A1**. The crude residue was purified by flash column chromatography (10→20% EtOAc/petroleum ether) to afford the titled product as a yellow oil (227 mg, 1.15 mmol, 16%); **δ_H** (500 MHz, $CDCl_3$) 7.93 (1H, s, H3), 7.37 (1H, s, H2), 7.32-7.26 (5H, m, 5 × Ph*H*), 6.97 (1H, s, H1), 6.04 (1H, s, H6'), 5.72 (1H, s, H6''); **δ_C** (125 MHz, $CDCl_3$) 117.06, 122.26, 126.40, 129.10, 129.40, 131.11, 134.71, 137.74, 142.72, 165.96; **IR** (thin film) ν_{max}/cm^{-1} 1712, 1642; **HRMS** *m/z* (ESI$^+$) found [M+H]$^+$ 199.0874; $C_{12}H_{11}N_2O^+$ requires 199.0871.

1-acryloyl-1H-indole-5-carbonitrile (**S34**)

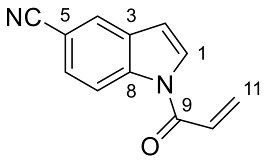

To a suspension of 1H-indole-5-carbonitrile (142.2 mg, 1.0 mmol, 1.0 equiv.), tetrabutylammonium bromide (161.2 mg, 0.5 mmol, 0.5 equiv.) and NaOH (60 mg, 1.5 mmol, 1.5 equiv.) in CH_2Cl_2, was added acryloyl chloride (0.12 mL, 1.5 mmol, 1.5 equiv.). The reaction was stirred at room temperature for 16 h, concentrated. The resulting residue was purified by column chromatography (7% acetone/pentane) to give the titled compound as an off-white solid (80 mg, 0.41 mmol, 41%); **m.p.** >300 °C (decomposition); **δ_H** (400 MHz, $CDCl_3$): 8.61 (1H, dt, *J* = 8.7, 0.8 Hz, H7), 7.92 (1H, dd, *J* = 1.7, 0.7 Hz, H4), 7.72 – 7.60 (2H, m, H1, H6), 6.96 (1H, dd, *J* = 16.8, 10.4 Hz, H10), 6.78 – 6.69 (2H, m, H2, H11, H2), 6.13 (1H, dd, *J* = 10.4, 1.3 Hz, H11); **δ_C** (100 MHz, $CDCl_3$): 163.99, 137.77, 133.75, 130.71, 128.41, 127.36, 126.83, 125.83, 119.64, 117.76, 108.98, 107.55; **IR** (thin film) ν_{max}/cm^{-1}: 2224, 1691, 1610, 1404, 1202; **HRMS** *m/z* (ESI$^+$) found [M+H]$^+$ 197.0712; $C_{12}H_9N_2O^+$ requires 197.0709.

3-oxo-2-phenyl-2,3-dihydro-1H-pyrrolo[1,2-a]indole-7-carbonitrile (**S35**)

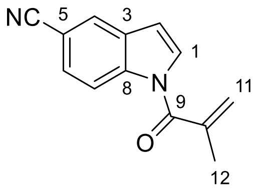

To a solution of trimethylsilyl methacrylate (0.36 mL, 2.0 mmol, 1.5 equiv.) and oxalyl chloride (0.32 mL, 2.4 mmol, 1.8 equiv.) in anhydrous CH_2Cl_2 (6 mL), was added one drop of anhydrous DMF. The reaction mixture was stirred at room temperature for 3 h. Once the reaction completed, anhydrous CH_2Cl_2 (12 mL), tetrabutylammonium bromide (322 mg, 1.0 mmol, 0.5 equiv.), freshly ground NaOH (288 mg, 7.2 mmol,

3.6 equiv.) and 1H-indole-5-carbonitrile (284.2 mg, 2.0 mmol, 1 equiv.) were added to the reaction mixture. The resulting mixture was stirred at room temperature for 16 h, concentrated and purified by column chromatography (5% acetone/pentane) to give the titled compound as a white solid (72 mg, 0.34 mmol, 17%, NMR slightly contaminated with "H-grease"); **m.p.** 69 °C; **δ_H** (400 MHz, $CDCl_3$): 8.49 (dt, J = 8.6, 0.8, Hz, 1H, H7), 7.92 (dd, J = 1.7, 0.7 Hz, 1H, H4), 7.68 – 7.52 (m, 2H, H1 and H2), 6.67 (dd, J = 3.8, 0.8 Hz, 1H, H11), 5.79 (d, J = 1.6 Hz, 1H, H11), 5.53 (d, J = 1.0 Hz, H11), 2.18 (dd, J = 1.6, 1.0 Hz, 3H, H12); **δ_C** (100 MHz, $CDCl_3$):169.60, 139.47, 137.61, 131.04, 129.28, 128.12, 125.76, 123.51, 119.67, 117.38, 107.98, 107.39, 19.94; **IR** (thin film) ν_{max}/cm^{-1}: 2223, 1698, 1459, 1363, 1331; **HRMS** *m/z* (ESI^+) found $[M+H]^+$ 211.0869; $C_{13}H_{11}N_2O^+$ requires 211.0866.

6. Deuterium Labelling and Trapping experiments

Synthesis of 2-deuterium indole was prepared according to literature procedure:[9]

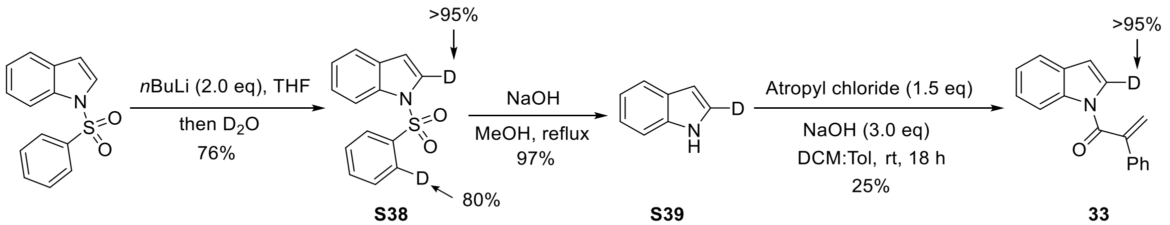

Scheme S2:** Synthesis of compound **33

1-((phenyl-2-d)sulfonyl)-1H-indole-2-d (**S38**)

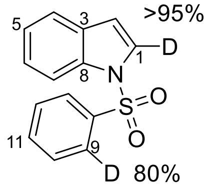

To a solution of 1-(phenylsulfonyl)-1H-indole (2.0 g, 7.77 mmol, 1 equiv.) in anhydrous THF (90 mL) at -78 °C, *n*BuLi (9.72 mL, 1.6 M, 15.55 mmol, 2 equiv.) was added dropwise and the resulting mixture was stirred for 3 h at -78 °C. The reaction was the quenched with D_2O (2 mL, 101 mmol, 13 equiv.) and was allowed to slowly worm up to room temperature over the course of 1 h. The reaction was then extracted with CH_2Cl_2, dried and evaporated. The resulting solid was purified by recrystallization from minimum amount of hot hexane to give the titled compound as a white solid (1.52 g, 5.91 mmol, 76%); $\boldsymbol{\delta_H}$ (400 MHz, $CDCl_3$): 8.00 – 7.93 (1H, m), 7.86 – 7.81 (1H, m), 7.52 – 7.43 (2H, m), 7.42 – 7.34 (2H, m), 7.27 (1H, ddd, *J* = 8.4, 7.2, 1.3 Hz), 7.23 – 7.15 (0.2H, m), 6.62 (1H, d, *J* = 0.8 Hz); $\boldsymbol{\delta_C}$ (100 MHz, $CDCl_3$): 138.39, 138.31, 134.94, 133.93, 130.87, 129.37, 129.26, 126.86, 126.84, 126.60, 126.51, 126.35, 126.21, 125.92, 124.76, 123.50, 121.54, 113.64, 109.19. Data are consistent with literature values.[12]

1H-indole-2-d (**S39**)

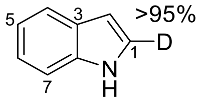

To a solution of **S38** (1.3 g, 5.0 mmol, 1 equiv.) in methanol (14.3 mL) was added an aqueous solution of sodium hydroxide (18.2 mL, 2M, 36.4 mmol, 7.28 equiv.). The mixture was refluxed for 16 h, when it was allowed to cool to room temperature and extracted with AcOEt. The organic layer was separated, dried, and evaporated. The resulting residue was dissolved in $CHCl_3$, filtered and evaporated to give the titled compound as an off-white solid (576 mg, 4.88 mmol, 97%); $\boldsymbol{\delta_H}$ (400 MHz, $CDCl_3$): 8.09 (1H, s, N*H*), 7.69 (1H, dq, *J* = 7.8, 1.0 Hz, H4), 7.41 (1H, dq, *J* = 8.1, 1.0 Hz, H7), 7.23 (1H, ddd, *J* = 8.2, 7.0, 1.2 Hz, H6), 7.15

12. Maresh, J. J.; Giddings, L.-A.; Friedrich, A.; Loris, E. A.; Panjikar, S.; Trout, B. L.; Stöckigt, J.; Peters, B.; O'Connor, S. E. J. Am. Chem. Soc. **2007**, *130*, 710.

(1H, ddd, *J* = 8.0, 7.0, 1.1 Hz, H5), 6.58 (1H, dd, *J* = 2.1, 0.9 Hz, H2); **δ_C** (100 MHz, $CDCl_3$): 135.87, 127.98, 124.32, 124.04, 123.82, 122.10, 120.86, 119.97, 119.95, 111.15, 102.58. Data are consistent with literature values.[9]

1-(1H-indol-1-yl-2-d)-2-phenylprop-2-en-1-one (**33**)

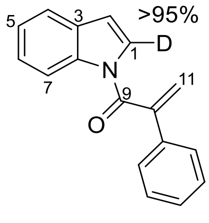

Synthesised from atropic acid (0.5 g, 3.375 mmol) and 1H-indole-2-d (331 mg, 2.25 mmol) using **General Procedure A1**. The reaction mixture was then concentrated and purified by FCC (2.5% Et_2O/Pentane) to give the titled compound as a clear oil (141 mg, 0.56 mmol, 25%); **m.p.** 148 °C; **δ_H** (400 MHz, $CDCl_3$): 8.57 (1H, dd, *J* = 8.2, 1.0 Hz, H7), 7.57 (1H, dt, *J* = 7.8, 1.0 Hz, H4), 7.52 – 7.46 (2H, m, H5, H6), 7.44 – 7.30 (5H, m, Ph*H*), 6.54 (1H, d, *J* = 0.8 Hz, H2), 6.08 (1H, s, H11), 5.70 (1H, s, H11); **δ_C** (100 MHz, $CDCl_3$): 168.63, 144.52, 135.65, 135.47, 131.05, 129.20, 129.16, 126.23, 125.26, 124.31, 121.02, 118.61, 116.84, 109.08; **IR** (thin film) ν_{max}/cm^{-1}: 1735, 1584, 1383, 1347; **HRMS** *m/z* (ESI$^+$) found [M+H]$^+$ 249.1133; $C_{17}H_{13}{}^2HNO^+$ requires 249.1133.

2-phenyl-1,2-dihydro-3H-pyrrolo[1,2-a]indol-3-one-2,9-d_2 (**34**)

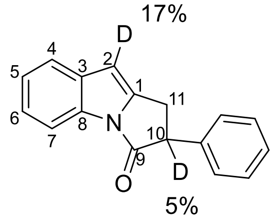

Compound **33** (74.5 mg, 0.3 mmol, 1 equiv.) and Ir(dFppy)$_3$ (2.3 mg, 0.003 mmol, 0.01 equiv.) were added to a dry microwave vial. The container was sealed, evacuated and black-filled with argon three times. Ethyl acetate (5 mL) and H_2O (2 mL) were placed in vial. The container was sealed and vigorously shaken. The layers were allowed to separate and the organic phase was transferred to a dry container and sparged with argon for 30 minutes. The deoxygenated solution (3 mL) was then transferred to the microwave vial and the suspension was stirred until homogeneous. The container was irradiated with blue LED light for 16 h, when the reaction was concentrated and purified by column chromatography (10% Et_2O/pentane) to give the titled compound as a white solid (68 mg, 0.27 mmol, 91%); **m.p.** 68 °C; **δ_H** (400 MHz, $CDCl_3$): 8.11 – 7.96 (1H, m, H7), 7.53 – 7.46 (1H, m, H4), 7.34 – 7.21 (7H, m, Ph*H*, H5, H6), 6.30 (0.83H, d, *J* = 1.8 Hz, H2), 4.32 (0.95H, dd, *J* = 9.2, 4.8 Hz, H10), 3.64 (1H, ddd, *J* = 17.5, 9.2, 1.5 Hz, H11), 3.21 (1H, ddd, J = 17.4, 4.8, 1.6 Hz, H11); **δ_C** (100 MHz, $CDCl_3$): 171.90, 141.84, 138.54, 135.55, 130.81, 129.23, 127.87, 127.82, 124.40, 123.57, 120.77, 113.96, 100.76, 52.87, 29.69; **IR** (thin film) ν_{max}/cm^{-1}: 1688, 1517, 1344; **HRMS** *m/z* (ESI$^+$) found [M+H]$^+$ 249.1126; $C_{17}H_{13}{}^2HNO^+$ requires 249.1133.

7-isocyano-2-phenyl-1,2-dihydro-3H-pyrrolo[1,2-a]indol-3-one-2-d (**35**)

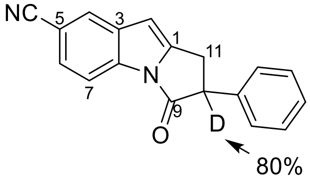

Compound **1** (74.5 mg, 0.3 mmol, 1 equiv.) and Ir(dFppy)$_3$ (2.3 mg, 0.003 mmol, 0.01 equiv.) were added to a dry microwave vial. The container was sealed, evacuated and black-filled with argon three times. Ethyl acetate (5 mL) and D_2O (2 mL) were placed in a dry vial. The container was sealed and vigorously shaken. The layers were allowed to separate and the organic phase was transferred to a dry container and sparged with argon for 30 minutes. The deoxygenated solution (3 mL) was then transferred to the microwave vial and the suspension was stirred until homogeneous. The container was irradiated with blue LED light for 16 h, when the reaction was concentrated and purified by column chromatography (15% AcOEt/pentane) to give the titled compound as a white solid (75.4 mg, 0.27 mmol, 92%); **m.p.** 163 °C; **δ_H** (400 MHz, $CDCl_3$): 8.16 (1H, dt, *J* = 8.3, 0.8 Hz, H7), 7.88 (1H, dd, *J* = 1.6, 0.7 Hz, H4), 7.55 (1H, dd, *J* = 8.3, 1.5 Hz, H6), 7.42 – 7.26 (5H, m, Ph*H*), 6.44 (1H, td, *J* = 1.6, 0.7 Hz, H2), 4.43 (0.2H, dd, *J* = 9.2, 4.9 Hz, H10), 3.81-3.72 (1H, m, H11), 3.37-3.30 (1H, m, H11); **δ_C** (100 MHz, $CDCl_3$): 171.96, 144.11, 144.08, 137.65, 137.63, 135.46, 132.47, 129.39, 128.21, 127.77, 127.75, 126.96, 125.55, 119.73, 114.62, 107.79, 100.51, 52.68, 52.54, 52.33, 52.13, 29.68, 29.58; **IR** (thin film) ν_{max}/cm^{-1}: 2220, 1735, 1584, 1383, 1347; **HRMS** *m/z* (ESI$^+$) found [M+H]$^+$ 274.1088; $C_{18}H_{12}{}^2HN_2O^+$ requires 249.1085.

2-(2-hydroxypropan-2-yl)-3-oxo-2-phenyl-2,3-dihydro-1H-pyrrolo[1,2-a]indole-7-carbonitrile (**32**)

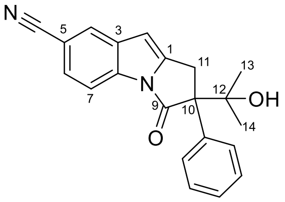

Synthesised from compound **1** (27.2 mg, 0.10 mmol) and degassed anhydrous acetone (2.5 mL) was used as solvent using **General Procedure A1**. The resulting crude material was purified by column chromatography (5% Et_2O/petroleum ether) to give the tiled compound as a minor product in the form of colourless oil (3.63 mg, 0.011 mmol, 11%); **δ_H** (400 MHz, $CDCl_3$) 8.20 (1H, d , *J* = 8.3 Hz, H7), 7.82 (1H, d, *J* = 1.5 Hz, H4), 7.76 (2H, m, 2 × *m*-Ph*H*), 7.55 (1H, dd, *J* =8.3 and 1.5 Hz, H6), 7.39 (2H, m, 2 × *o*-Ph*H*), 7.33 (1H, m, *p*-Ph*H*), 6.32 (1H, t, *J* = 1.5 Hz, H2), 3.98 (1H, dd, *J* =17.8 and 1.5 Hz, H11'), 3.69 (1H, dd, *J* = 17.8 and 1.5 Hz, H11''), 1.43 (1H, s, O*H)*, 1.30 (3H, s, H14), 1.26 (3H, s, H13); **δ_C** (100 MHz, $CDCl_3$) 173.12, 143.33, 138.12, 135.48, 132.47, 128.50, 128.25, 127.95, 126.83, 125.41, 119.75, 114.62 107.83, 99.63, 75.12, 66.06, 32.48, 27.07, 25.45; **IR** (thin film) ν_{max}/cm^{-1} 3426, 1725, 1372, 1091; **HRMS** *m/z* (ESI$^+$) found [M+H]$^+$ 331.3948; $C_{21}H_{19}N_2O_2^+$ requires 331.3950.

7. Large Scale Synthesis

Amide substrate **S7** (1.5 g, 4.6 mmol, 1 equiv.), Ir(dFppy)$_3$ (35 mg, 0.046 mmol, 0.01 equiv.) and NaOAc (38 mg, 0.46 mmol, 0.1 equiv.) were transferred to an appropriately sized Schlenk tube. The Schlenk tube was evacuated and backfilled with Ar three times. The reaction vessel was then charged with EtOAc (46 mL) that was previously sparged with Ar for 15 min. The reaction mixture was then irradiated with blue light and stirred while maintaining at room temperature by a stream of N_2. Once the reaction was completed, the excess solvent was evaporated and the resulting solid was purified by column chromatography (5% AcOEt/pentane) to give the tiled compound as a white solid (1.39 g, 4.28 mmol, 93%). Characterization data are consistent with small scale reaction.

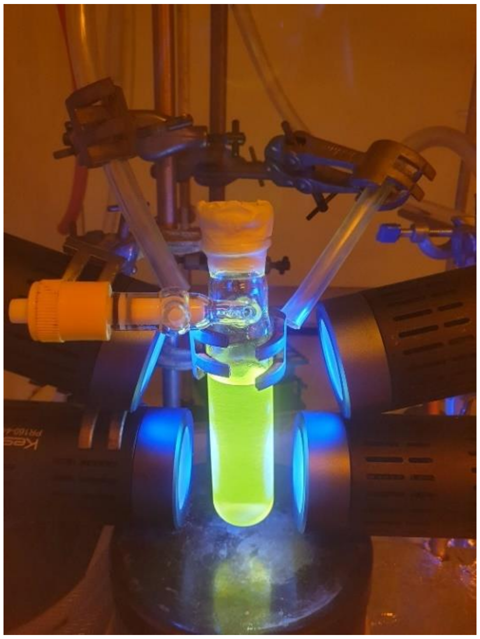

Figure S1: *Experimental setup as viewed through a blue light filter.*

8. Stern-Volmer Quenching

A stock solution of Ir(dFppy)$_3$ (2.0 mg) in EtOAc (10.0 mL; 0.26 mM) was prepared by adding argon sparged EtOAc to a volumetric flask under argon containing the photocatalyst. A stock solution of substrate **1** (10.9 mg) in EtOAc (2.0 mL; 20 mM) was prepared in a similar manned by adding argon sparged EtOAc to a vial containing substrate **1** sealed with a septum under an atmosphere of argon.

Samples were prepared in 4.0 mL quartz cuvettes equipped with a septum under an argon atmosphere by adding 2.0 mL of the photocatalyst stock solution which was diluted with a further 1.0 mL of argon sparged EtOAc (final concentration of photocatalyst = 0.175 mM). The resulting solution was excited at 400 nm and three emission spectra recorded from 430 to 700 nm. To this solution was then added 50 µL of substrate **1** stock solution under argon, and three emission spectra recorded as before. This process was then repeated five further times.

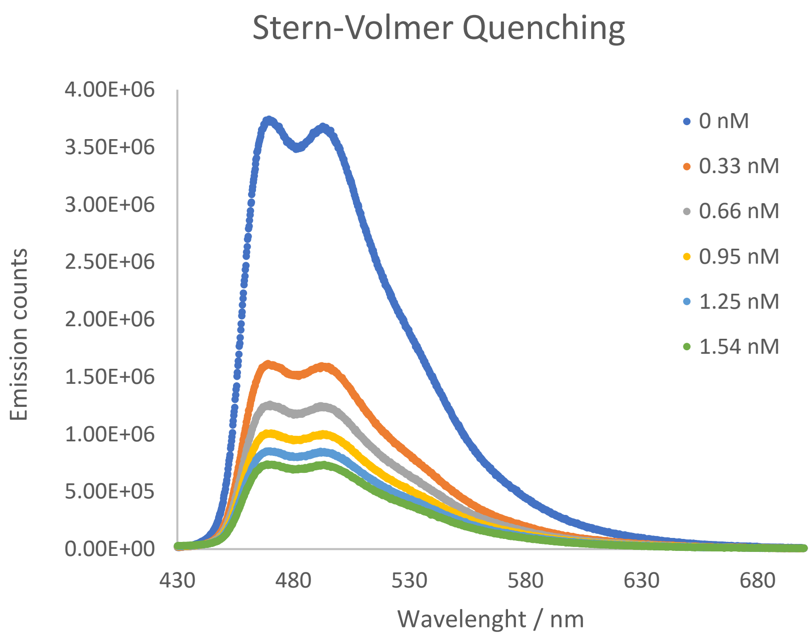

Figure S2*. Stacked emission spectra of Ir(dFppy)$_3$ on addition of increasing amounts of substrate* ***2****.*

By using the Stern-Volmer relationship:

$$\frac{I_0}{I} = k_q[Q] + 1$$

Where I_0 is the emission of the photocatalyst in the absence of quencher, and I is the emission of the photocatalyst at a known quencher concentration, a graph plotting (I_0/I) against quencher concentration ([Q]) should give a straight line with y intercept of 1 and a gradient quantifying the rate of quenching (k_q). **Figure S3**.

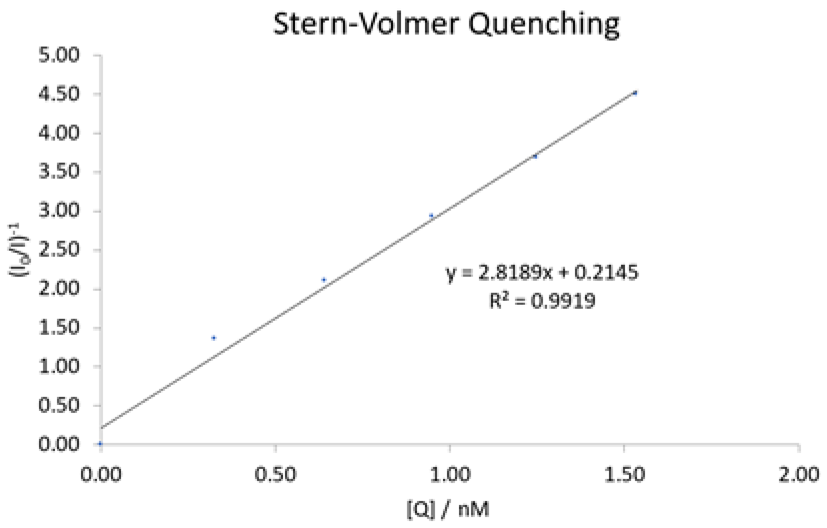

Figure S3. Stern-Volmer plot of $(I_0/I)^{-1}$ against quencher concentration

9. Square voltammetry

Voltammetry measurements were made using an Autolab PGSTAT 12 with a 3 mm glassy carbon working electrode, platinum wire counter electrode and Ag/$AgNO_3$ (0.01 M in acetonitrile) reference electrode. Voltammograms were referenced to the Fc/Fc^+ couple (0.40 V vs SCE) as an internal reference. Square wave voltammograms were acquired with a 5 mV step potential, 50 mV modulation amplitude and 2 Hz frequency. The supporting electrolyte, (tetra-*n*-butylammonium hexafluorophosphate, Bu_4NPF_6, TBAP) was prepared as a 0.1 M solution in dry MeCN. A stock solution of **1** (2.7 mg) in dry MeCN (1.0 mL; 10 mM) was prepared and then further diluted with the electrolyte solution to a concentration of 1 mM.

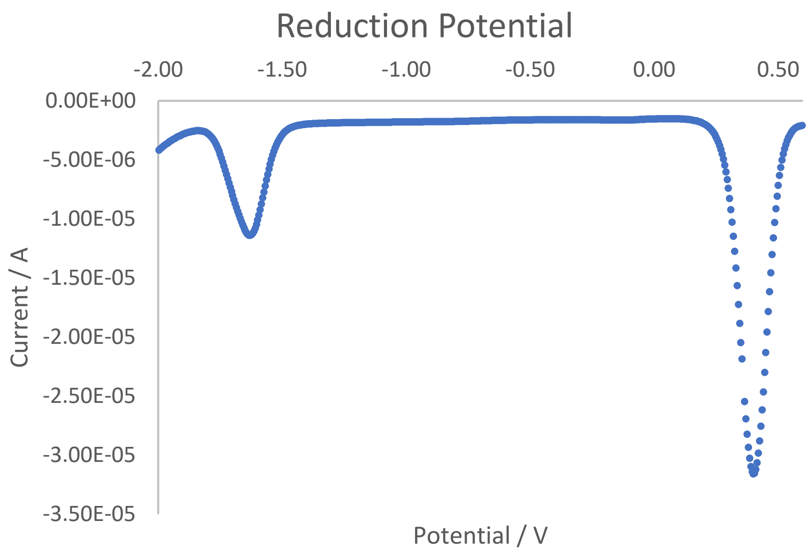

Figure S4: *Reduction of compound **1***

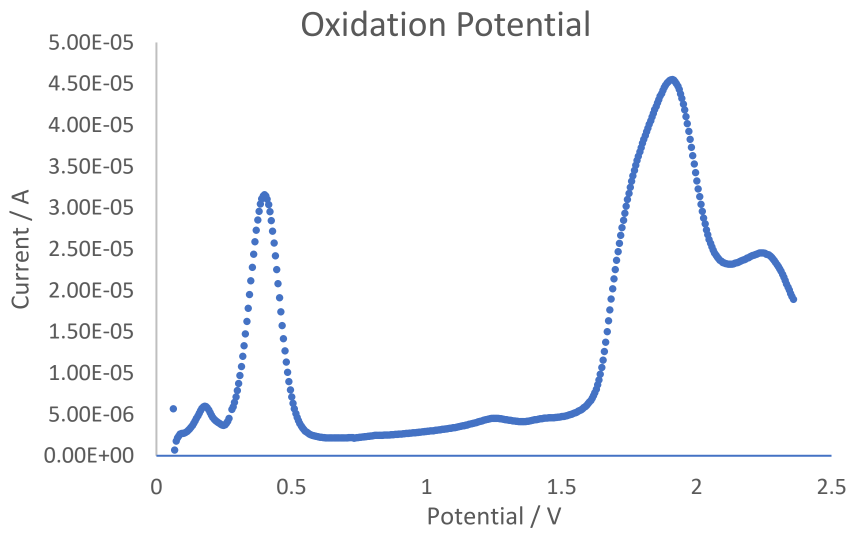

Figure S5: *Oxidation of compound **1***

10. NMR Spectra

^{1}H-NMR: 1-(2-phenylacryloyl)-1H-indole-5-carbonitrile (**1**)

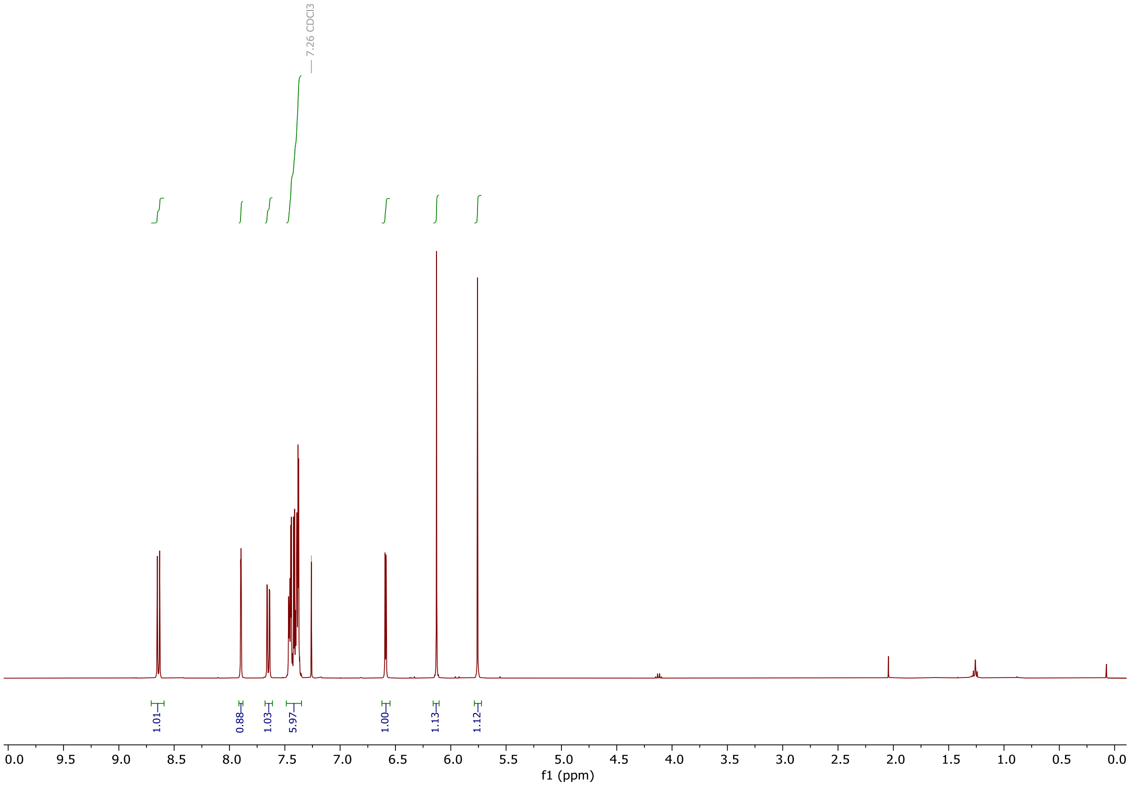

^{13}C-NMR: 1-(2-phenylacryloyl)-1H-indole-5-carbonitrile (**1**)

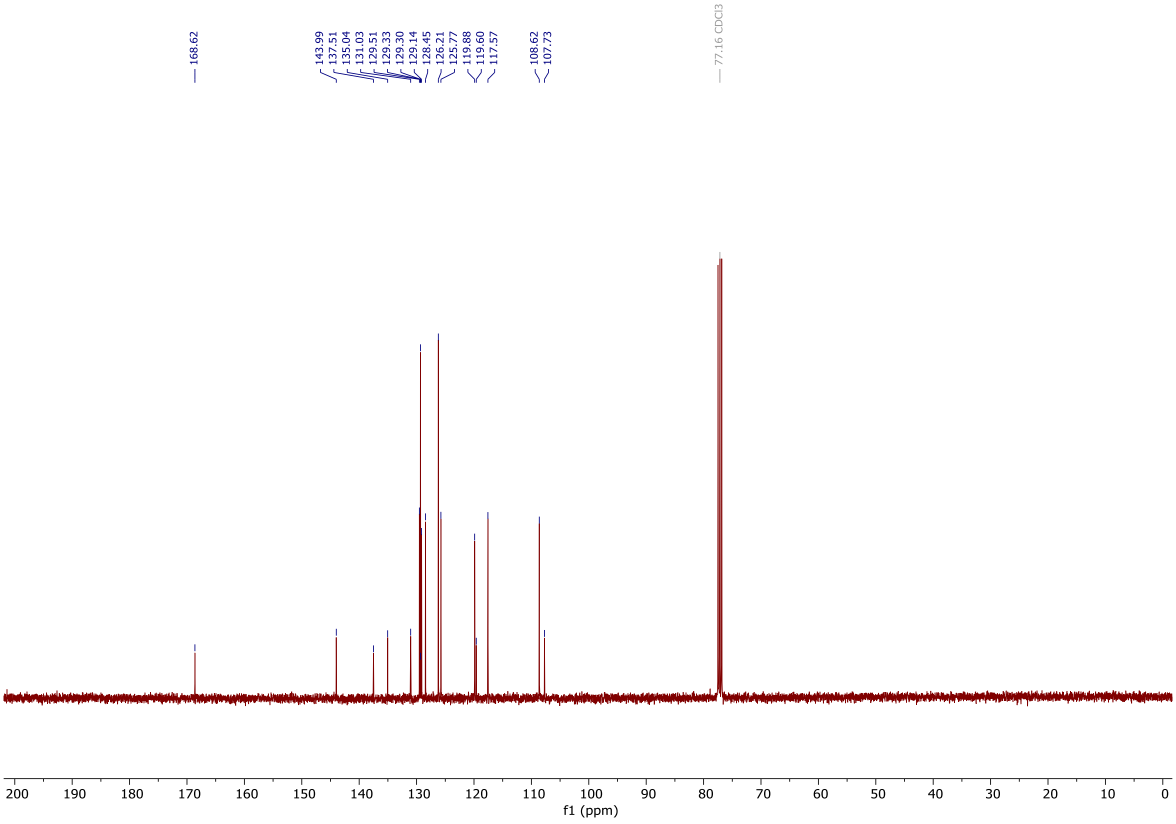

^{1}H-NMR: 3-oxo-2-phenyl-2,3-dihydro-1H-pyrrolo[1,2-a]indole-7-carbonitrile (**2**)

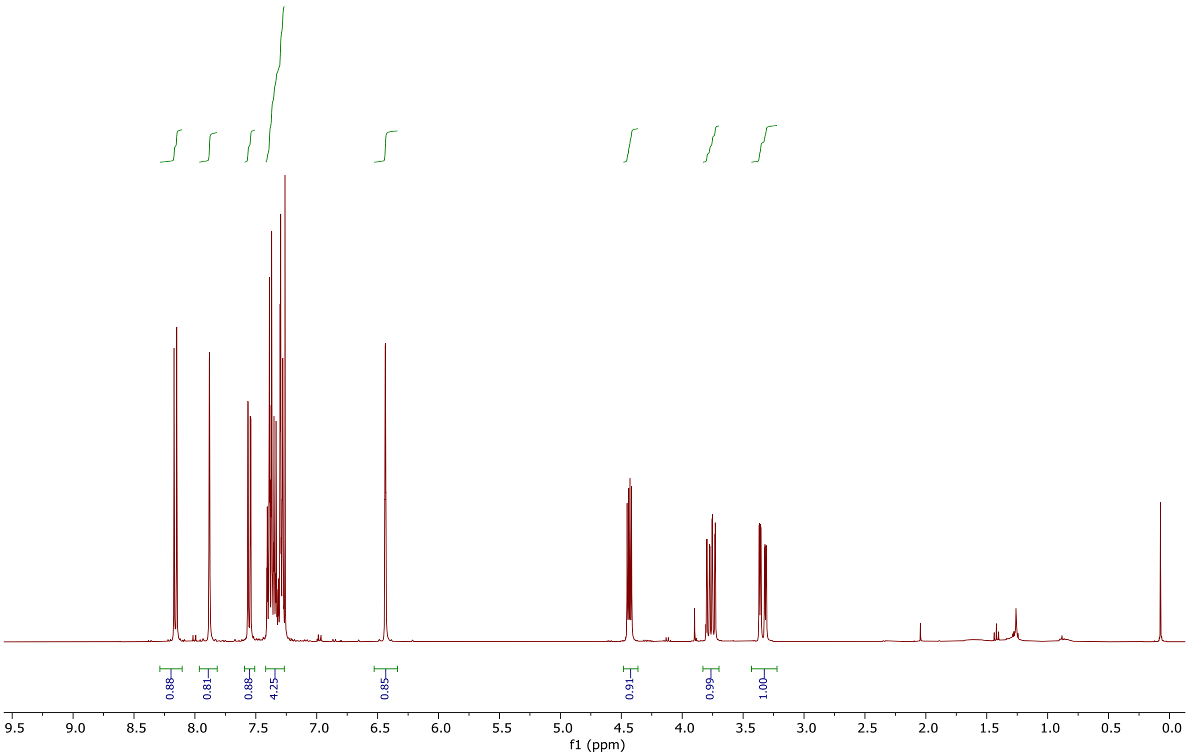

^{13}C-NMR: 3-oxo-2-phenyl-2,3-dihydro-1H-pyrrolo[1,2-a]indole-7-carbonitrile (**2**)

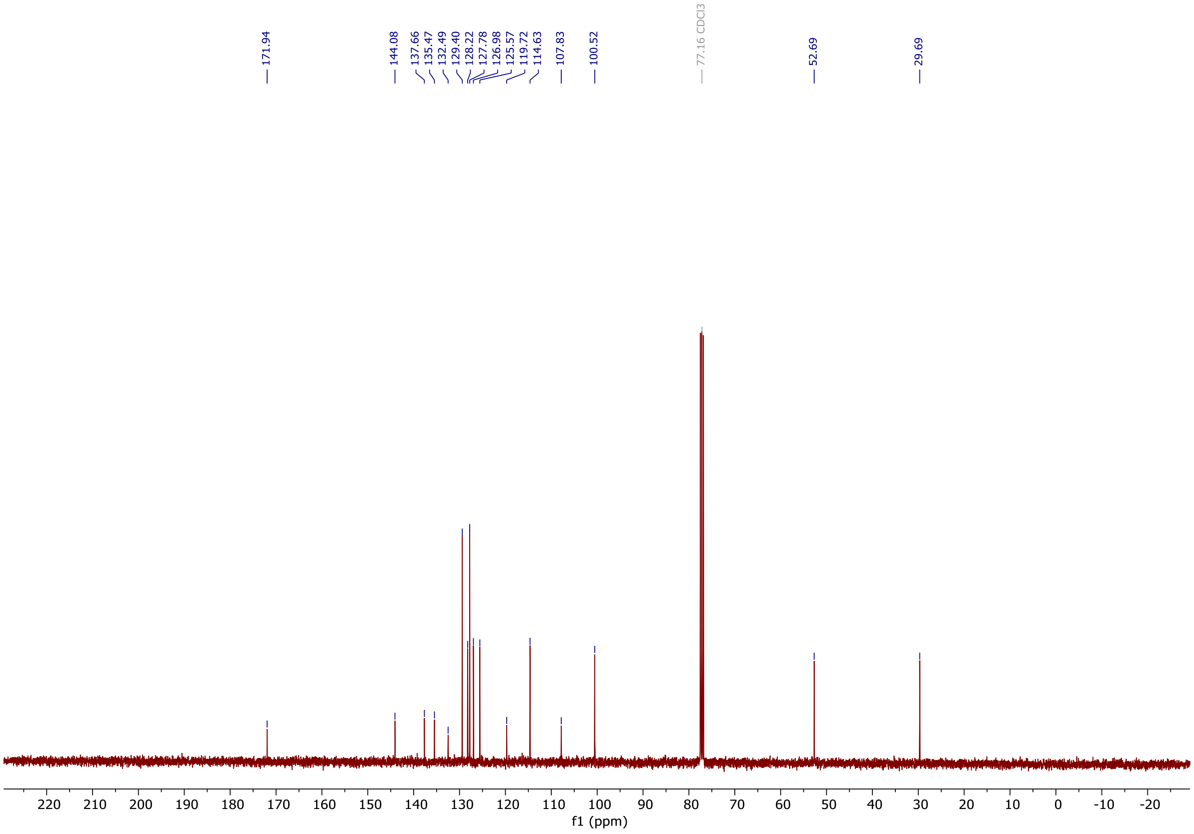

^{1}H-NMR: 1-(1H-indol-1-yl)-2-phenylprop-2-en-1-one (**S1**)

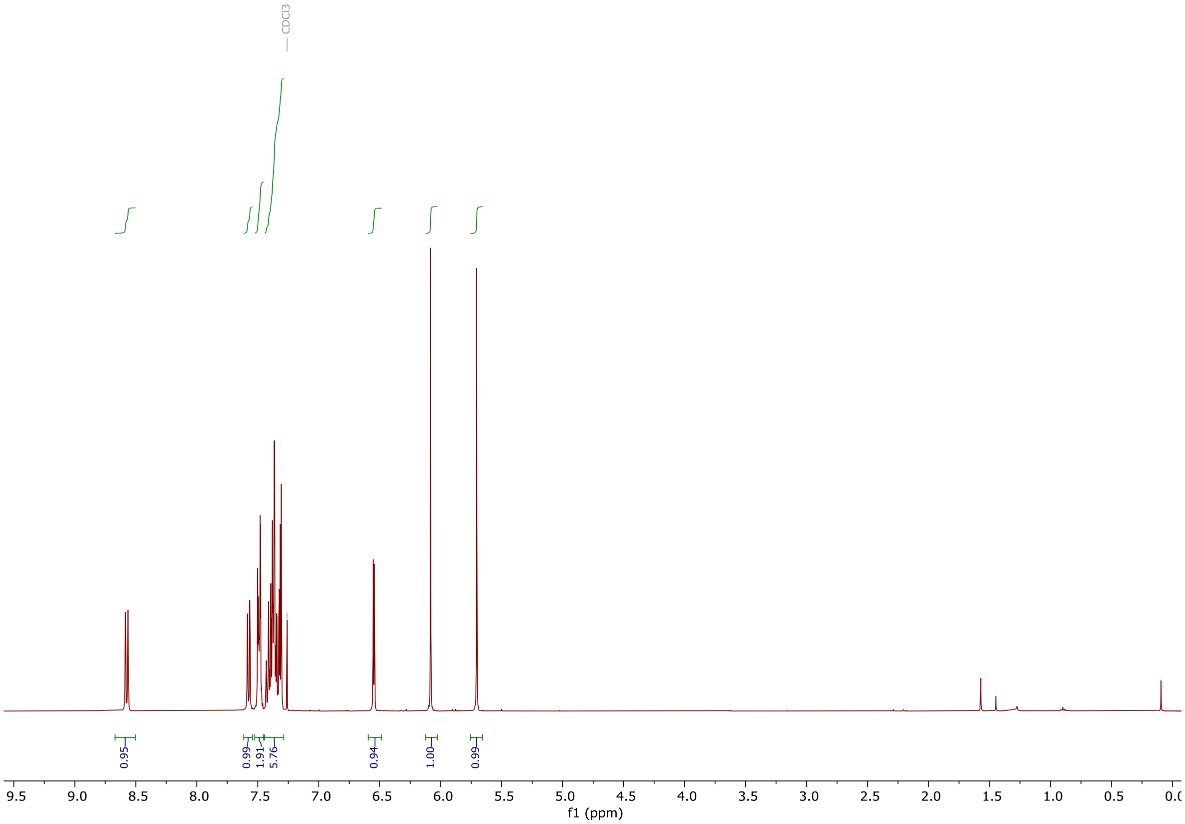

^{13}C-NMR: 1-(1H-indol-1-yl)-2-phenylprop-2-en-1-one (**S1**)

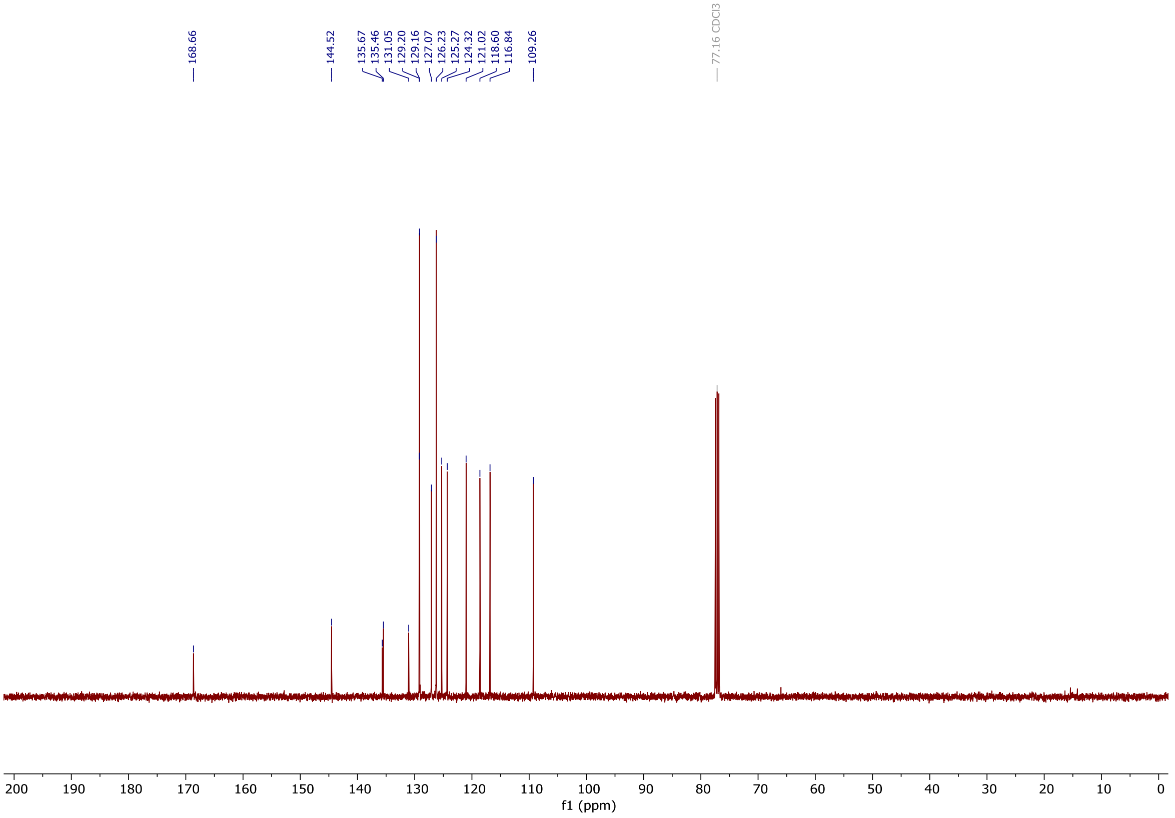

^{1}H-NMR: 2-phenyl-1,2-dihydro-3H-pyrrolo[1,2-a]indol-3-one (**3**)

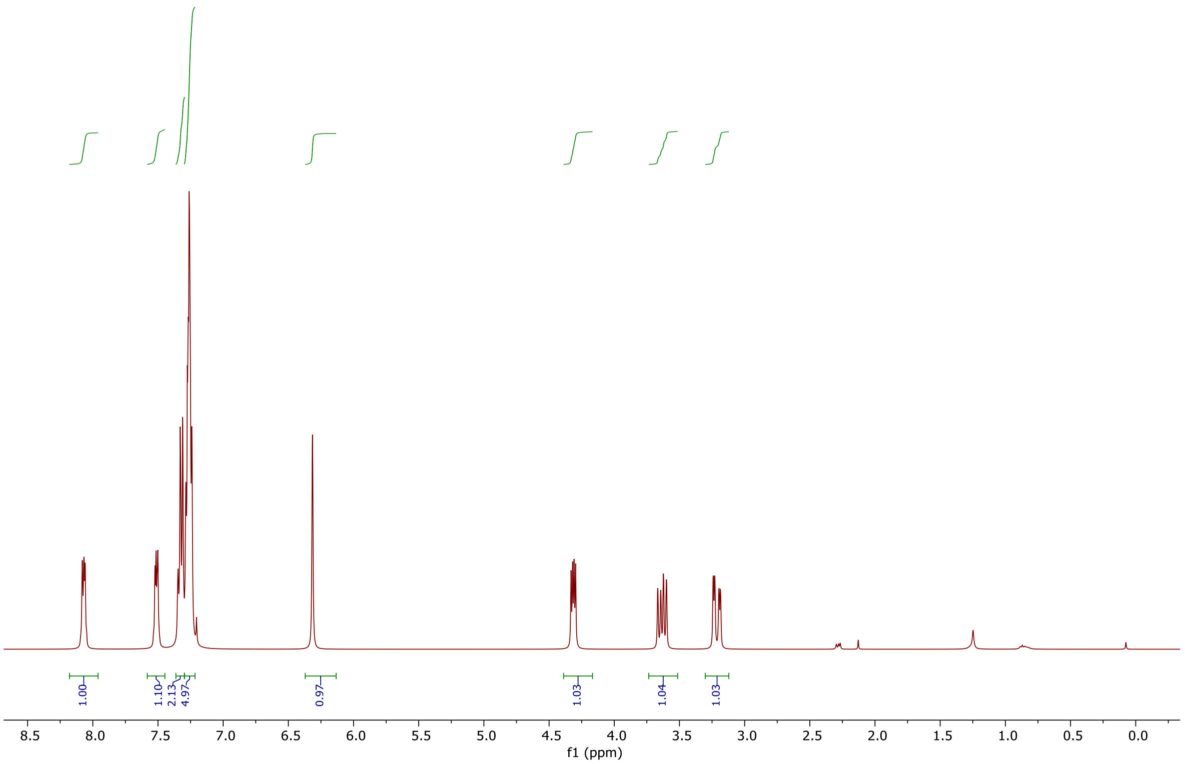

^{13}C-NMR: 2-phenyl-1,2-dihydro-3H-pyrrolo[1,2-a]indol-3-one (**3**)

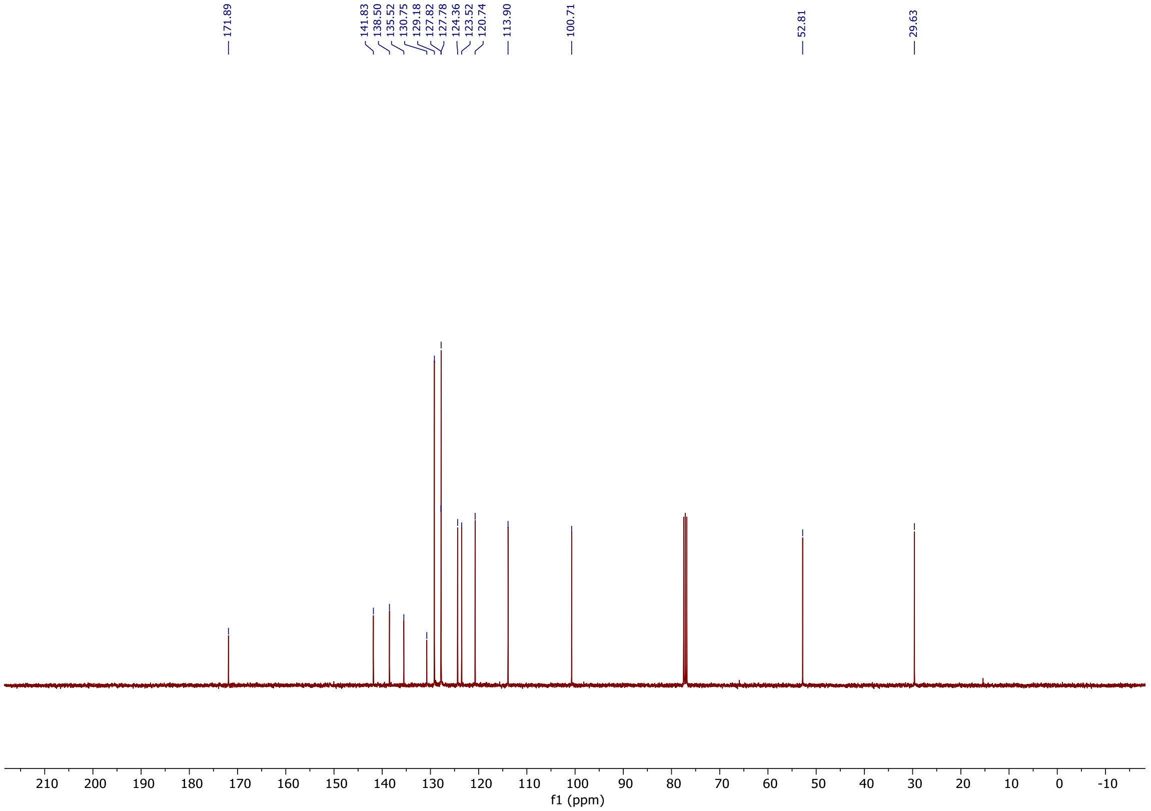

¹H-NMR: 1-(4-((tert-butyldimethylsilyl)oxy)-1H-indol-1-yl)-2-phenylprop-2-en-1-one (**S2**)

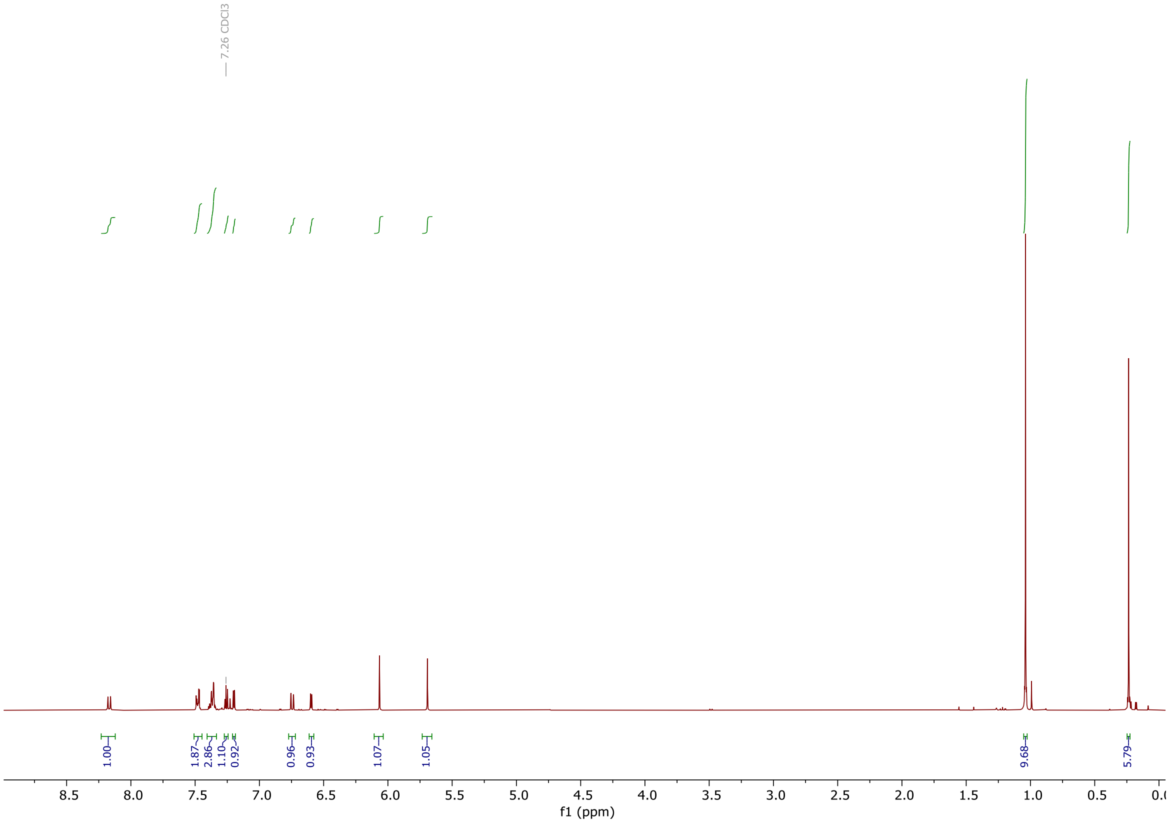

¹³C-NMR: 1-(4-((tert-butyldimethylsilyl)oxy)-1H-indol-1-yl)-2-phenylprop-2-en-1-one (**S2**)

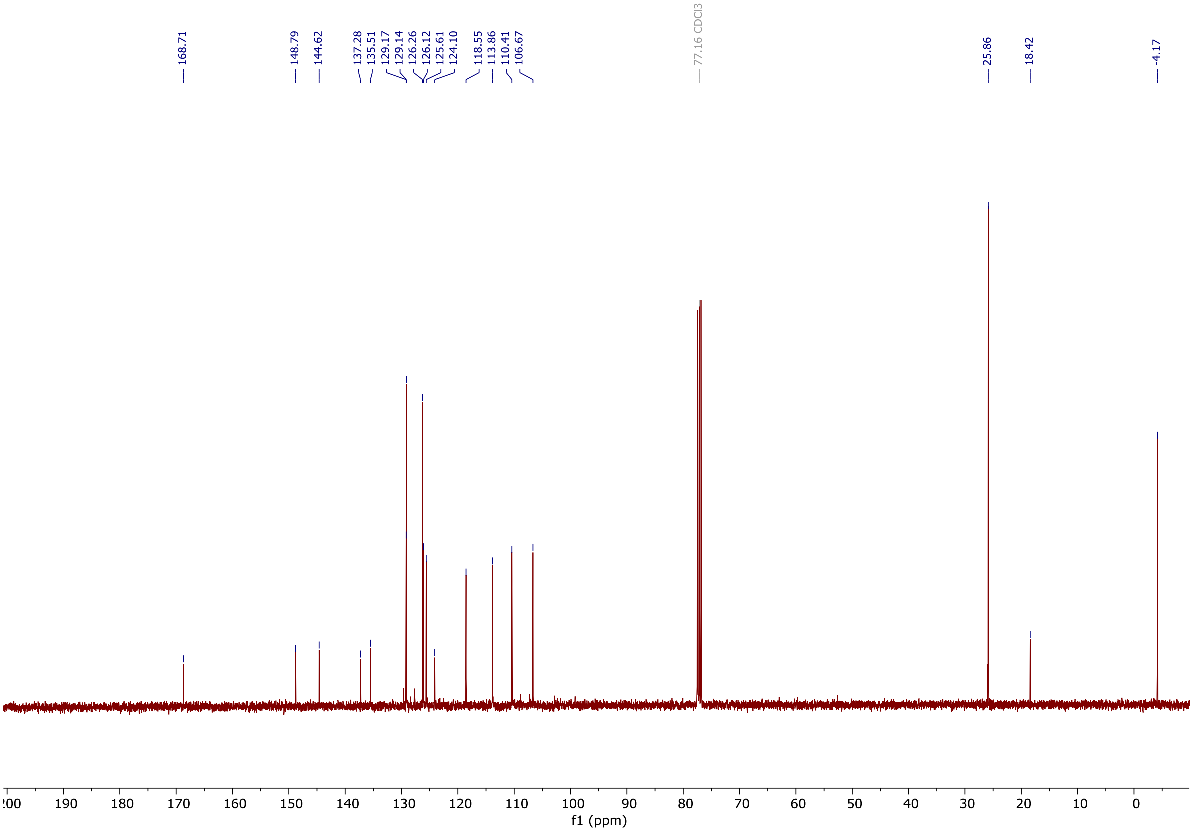

^{1}H-NMR: 8-((tert-butyldimethylsilyl)oxy)-2-phenyl-1,2-dihydro-3H-pyrrolo[1,2-a]indol-3-oneone (**4**)

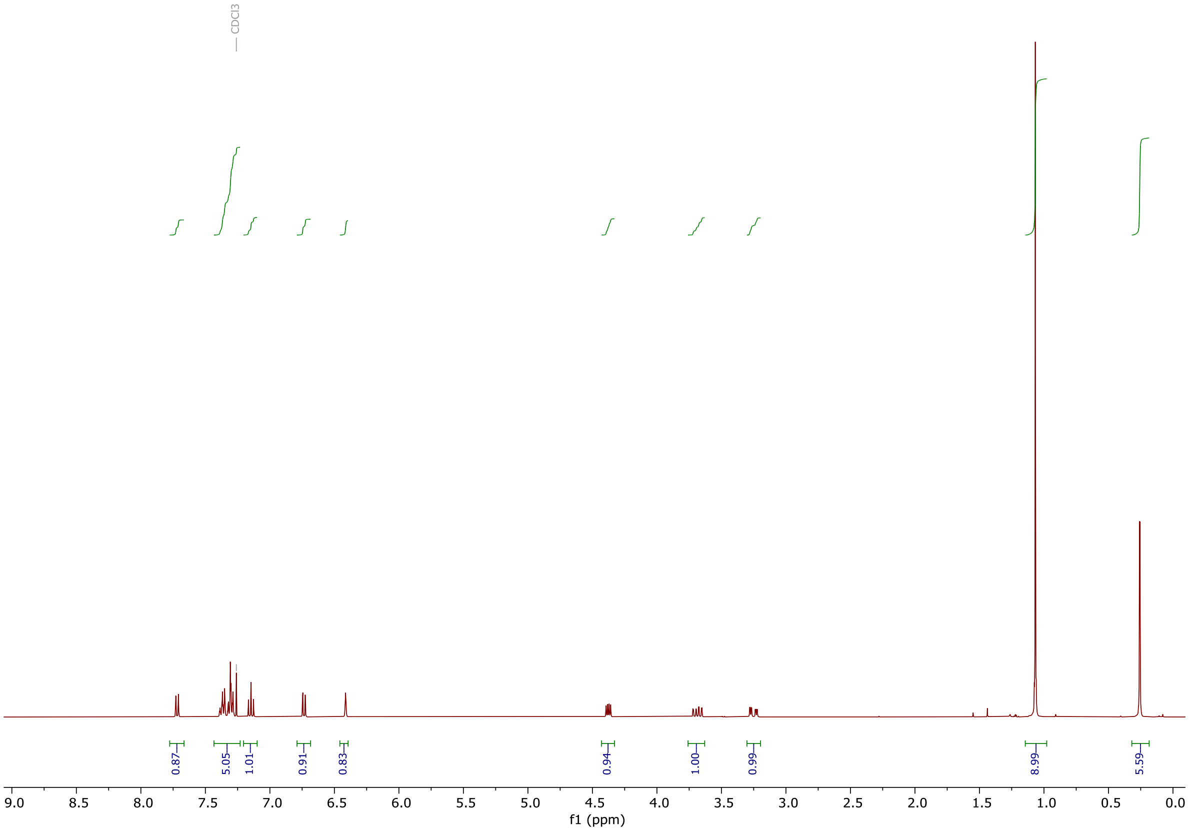

^{13}C-NMR: 8-((tert-butyldimethylsilyl)oxy)-2-phenyl-1,2-dihydro-3H-pyrrolo[1,2-a]indol-3-oneone (**4**)

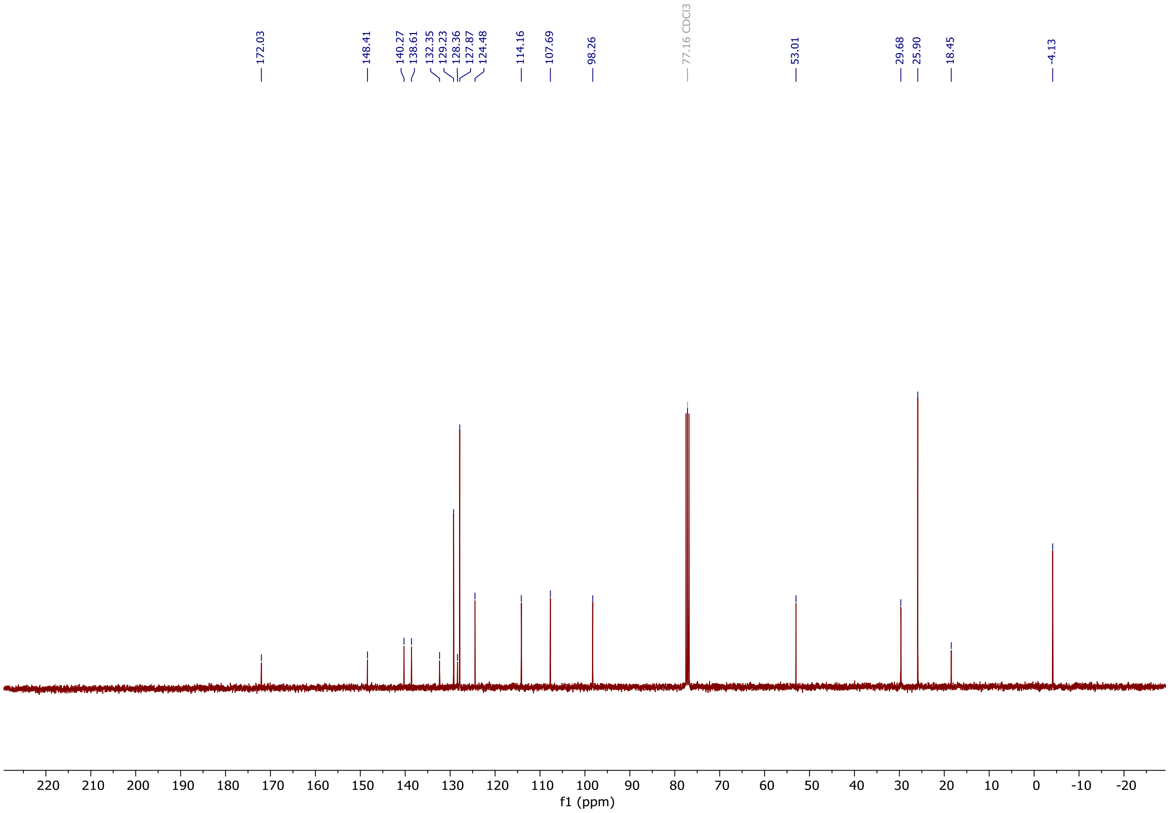

^{1}H-NMR: 1-(4-chloro-1H-indol-1-yl)-2-phenylprop-2-en-1-one (**S3**)

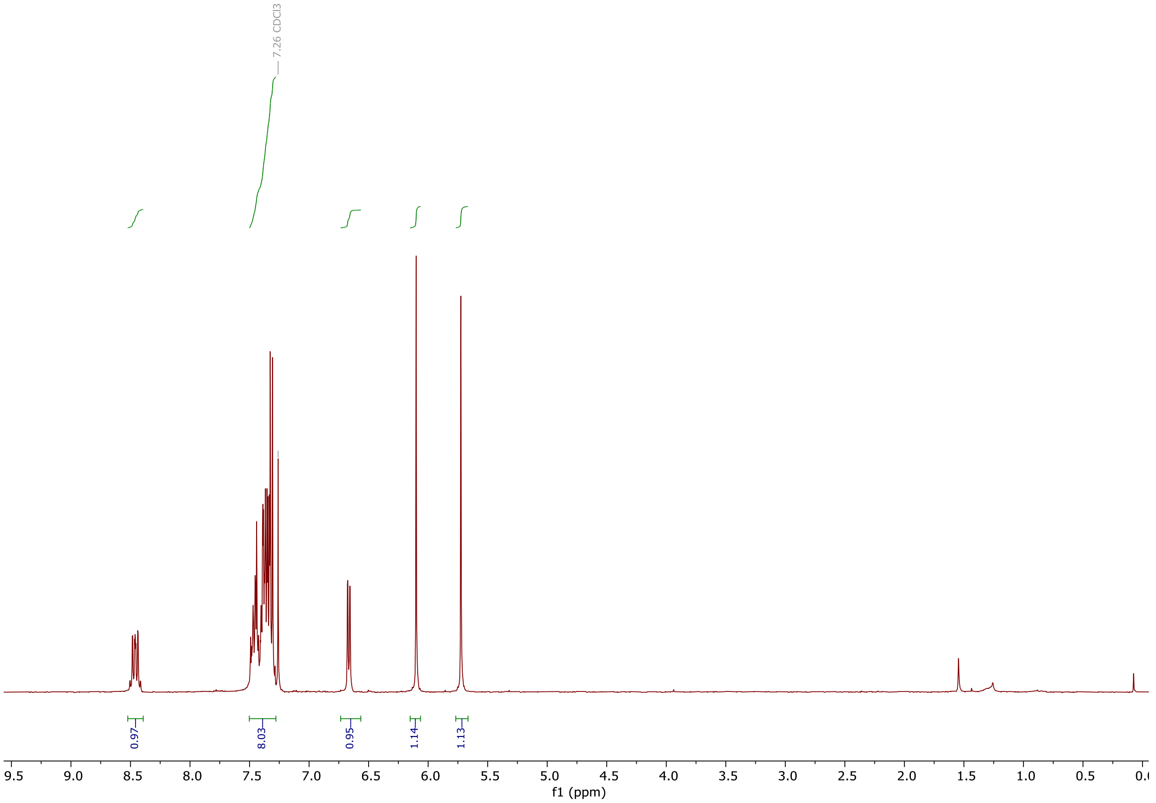

^{13}C-NMR: 1-(4-chloro-1H-indol-1-yl)-2-phenylprop-2-en-1-one (**S3**)

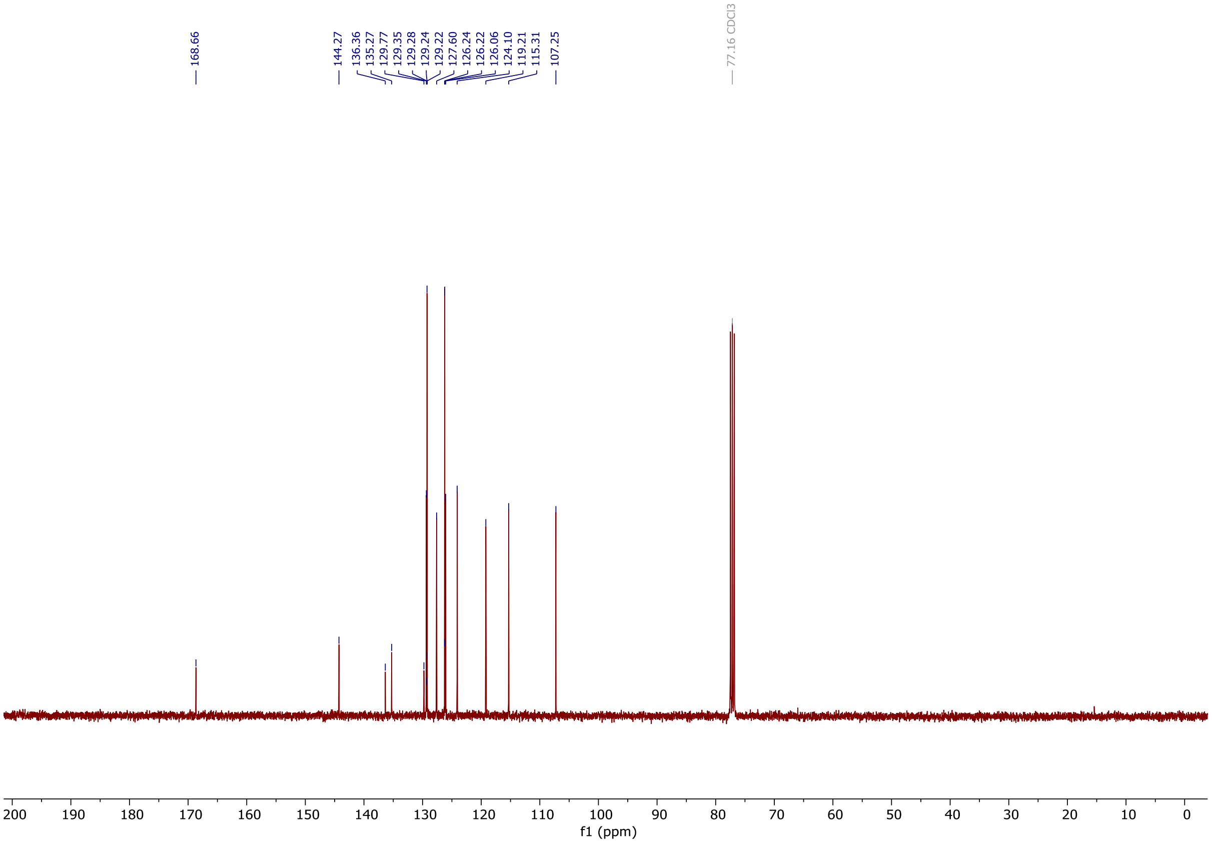

^{1}H-NMR: 8-chloro-2-phenyl-1,2-dihydro-3H-pyrrolo[1,2-a]indol-3-one (**5**)

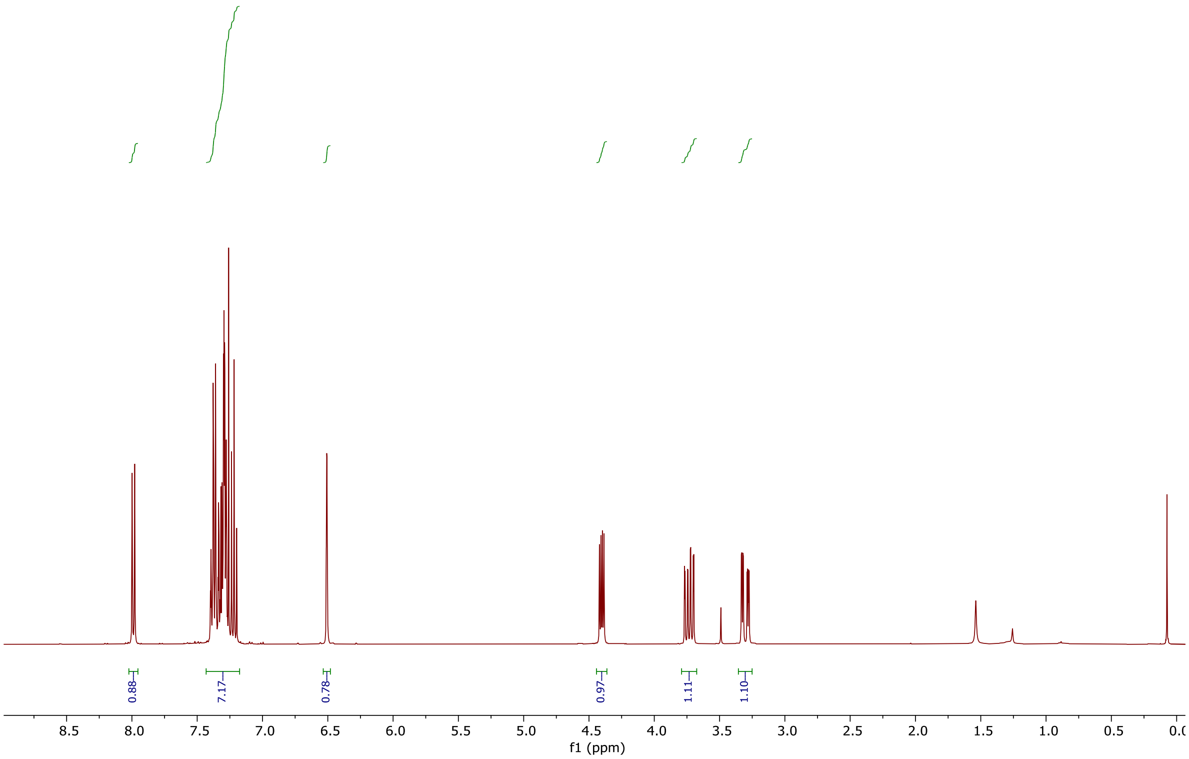

^{13}C-NMR: 8-chloro-2-phenyl-1,2-dihydro-3H-pyrrolo[1,2-a]indol-3-one (**5**)

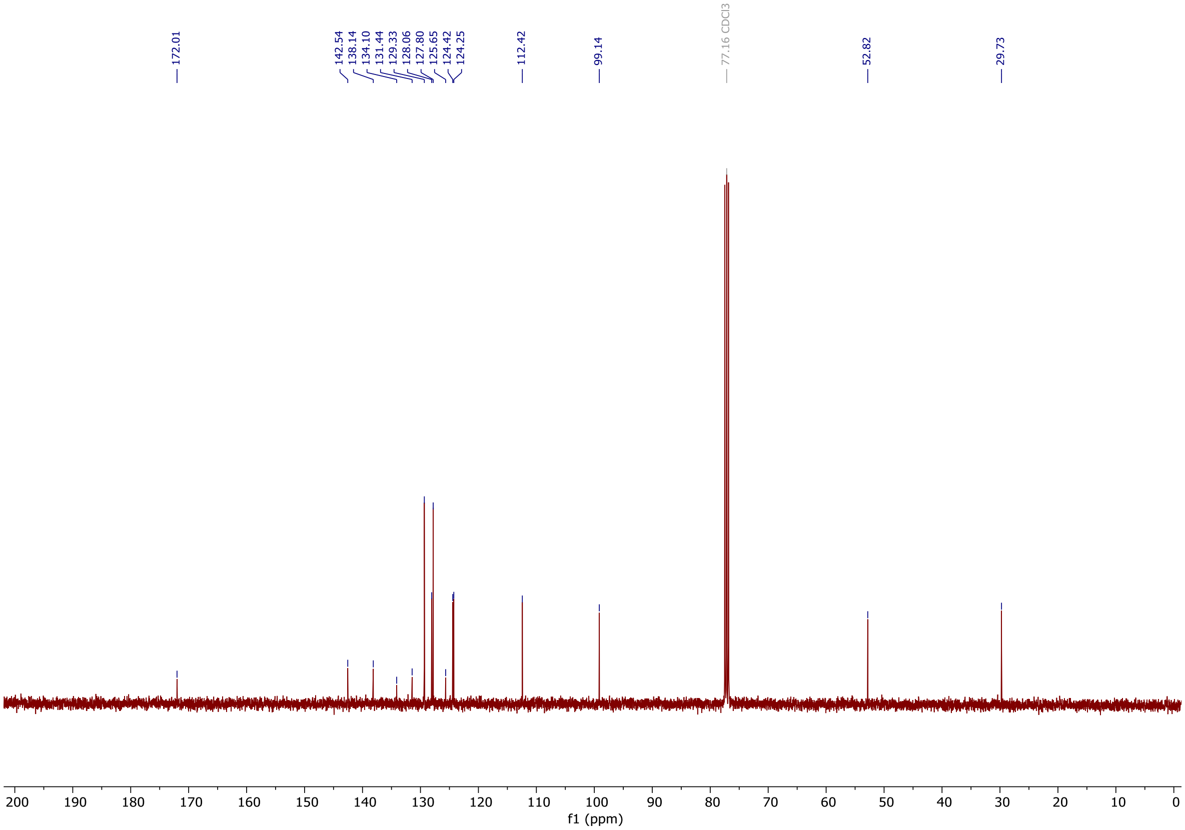

¹H-NMR: 1-(4-bromo-1H-indol-1-yl)-2-phenylprop-2-en-1-one (**S4**)

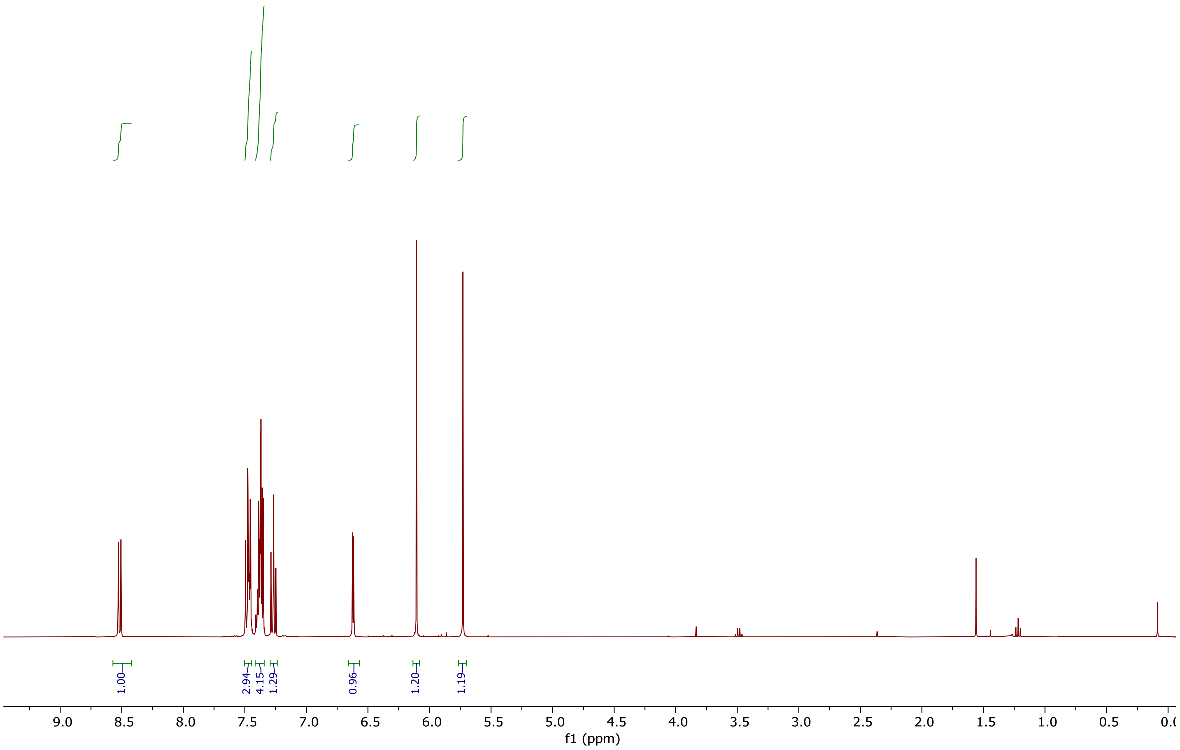

¹³C-NMR: 1-(4-bromo-1H-indol-1-yl)-2-phenylprop-2-en-1-one (**S4**)

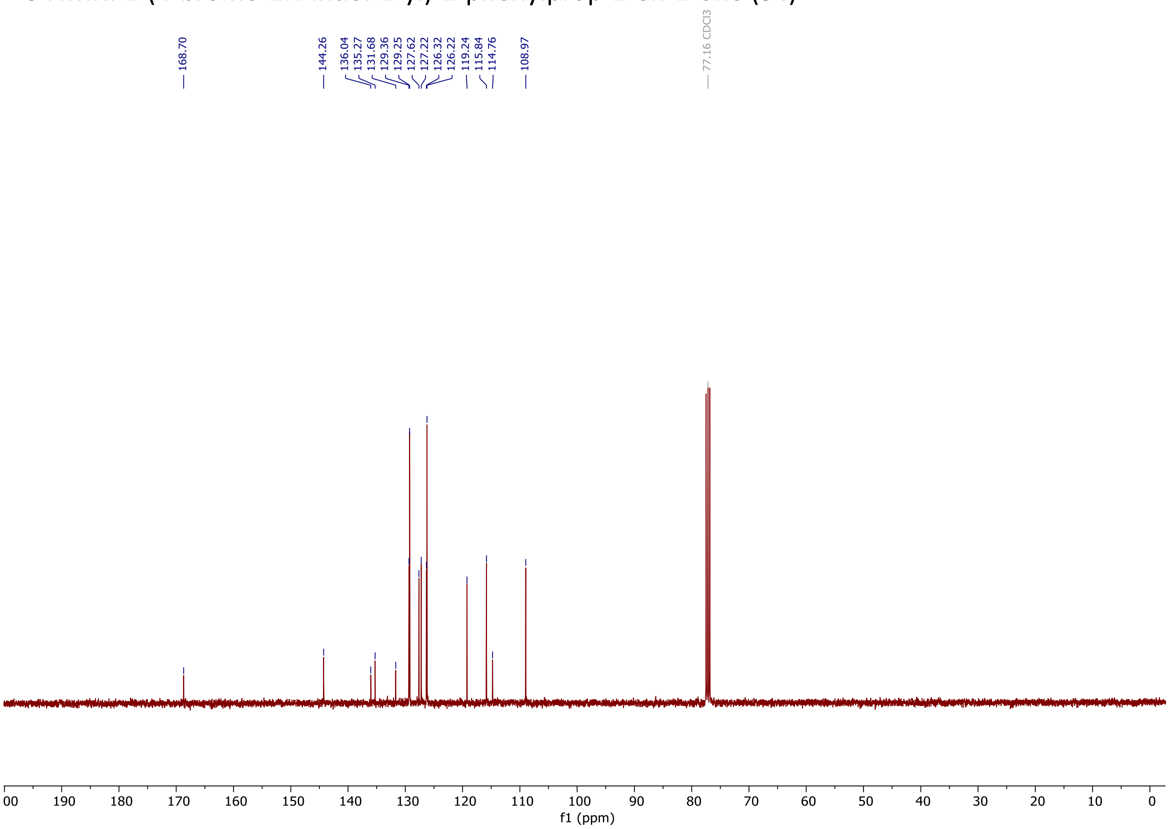

¹H-NMR: 8-bromo-2-phenyl-1,2-dihydro-3H-pyrrolo[1,2-a]indol-3-one (**6**)

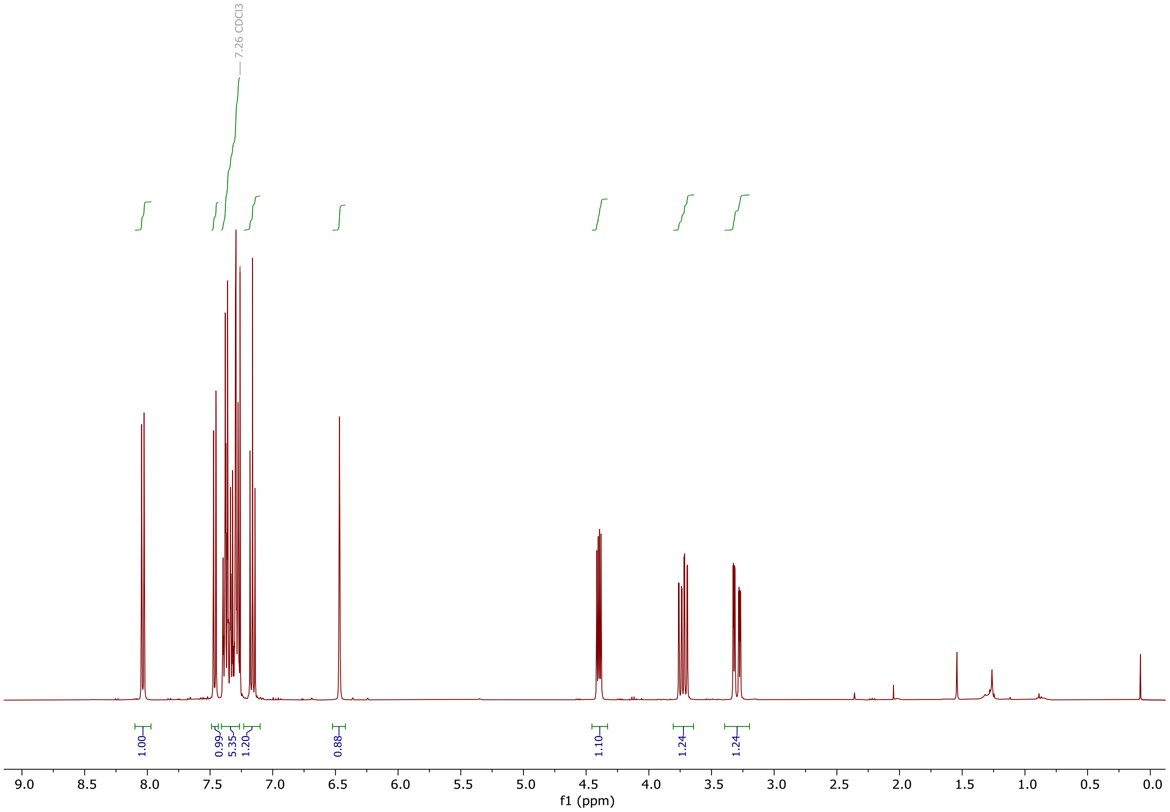

¹³C-NMR: 8-bromo-2-phenyl-1,2-dihydro-3H-pyrrolo[1,2-a]indol-3-one (**6**)

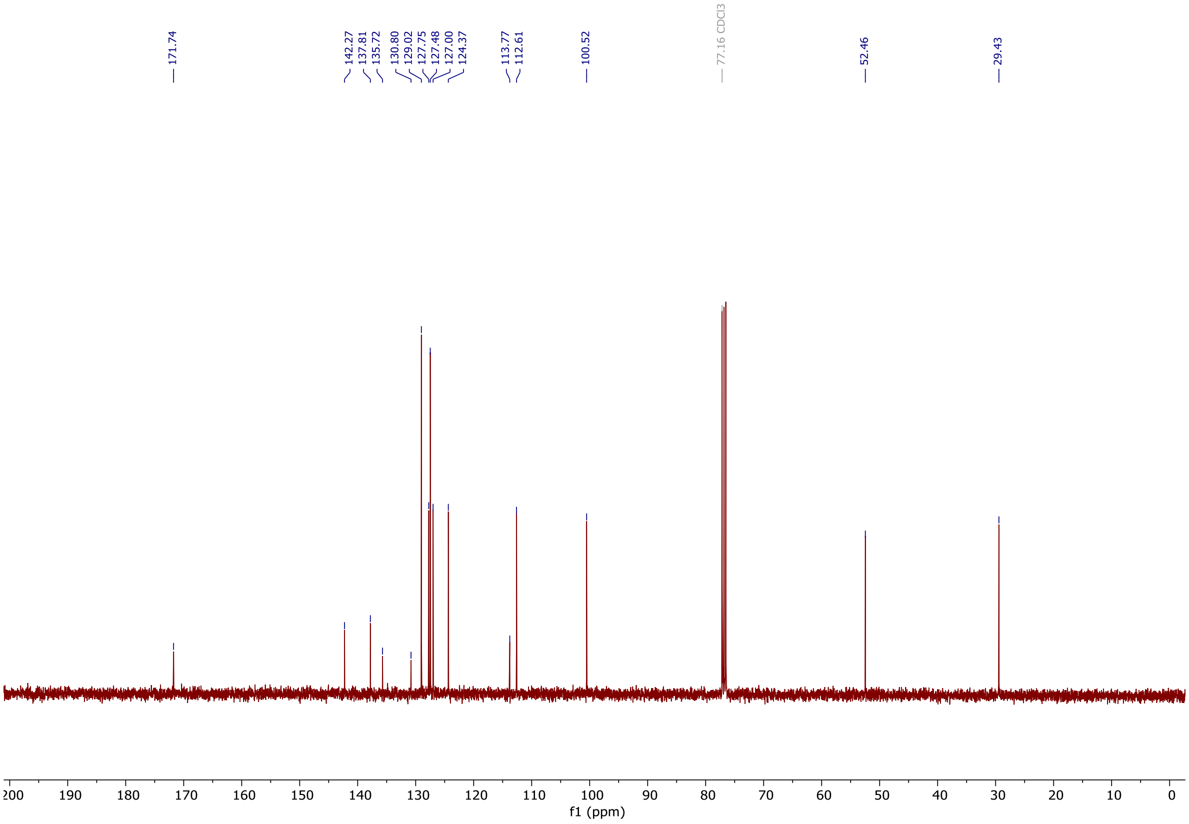

^{1}H-NMR: 1-(2-phenylacryloyl)-1H-indol-4-yl methanesulfonate (**S5**)

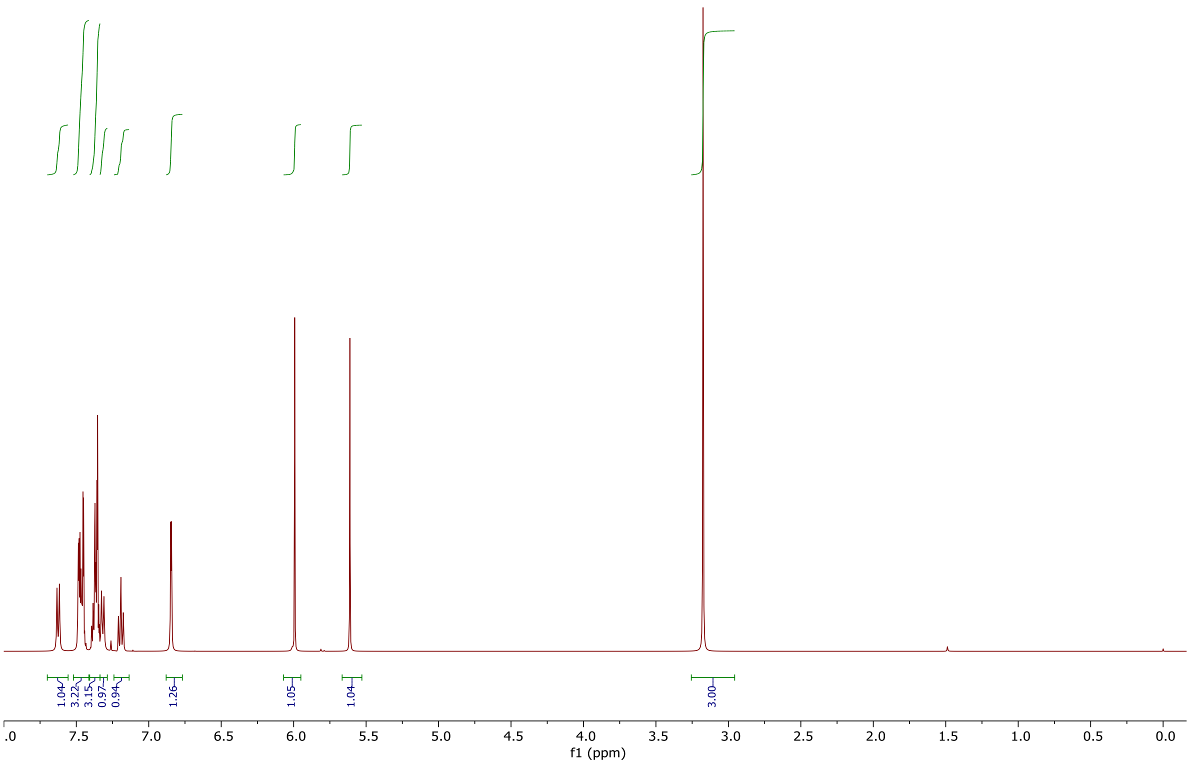

^{13}C-NMR: 1-(2-phenylacryloyl)-1H-indol-4-yl methanesulfonate (**S5**)

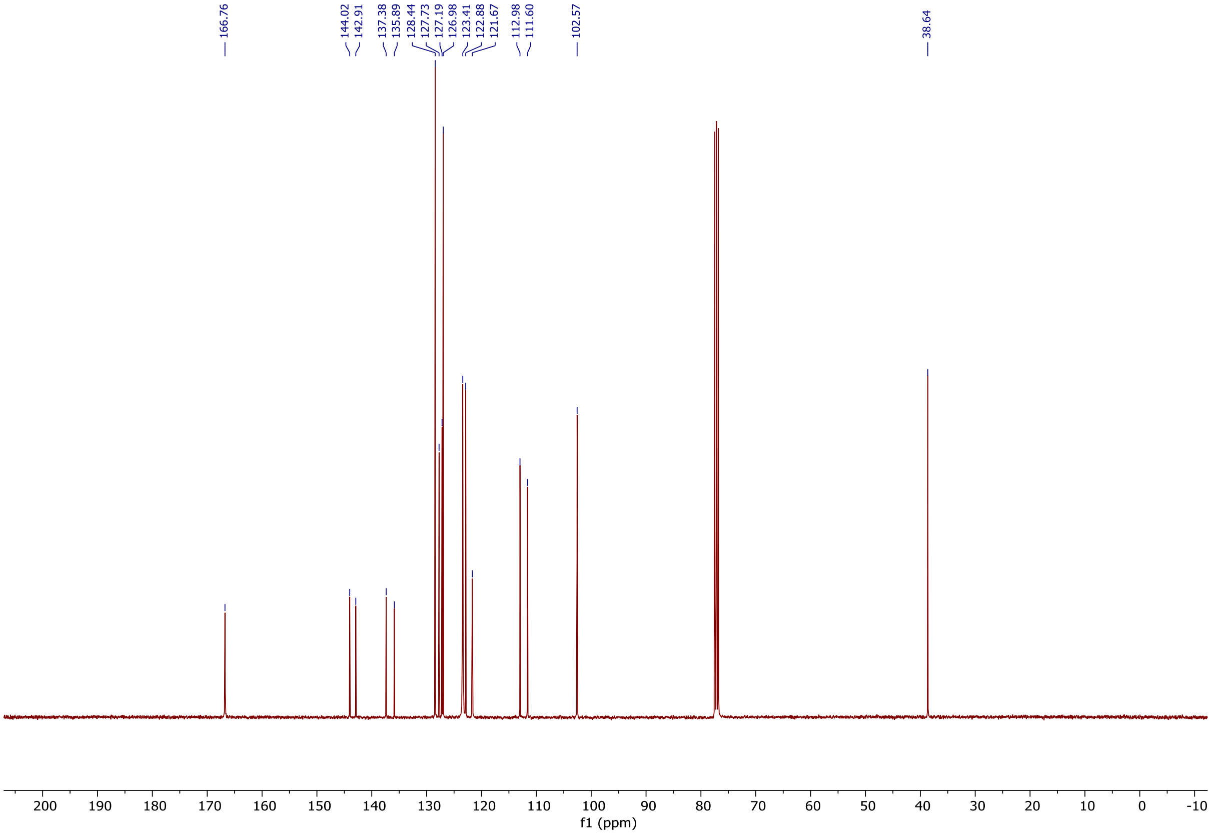

^{1}H-NMR: 3-oxo-2-phenyl-2,3-dihydro-1H-pyrrolo[1,2-a]indol-8-yl methanesulfonate (**7**)

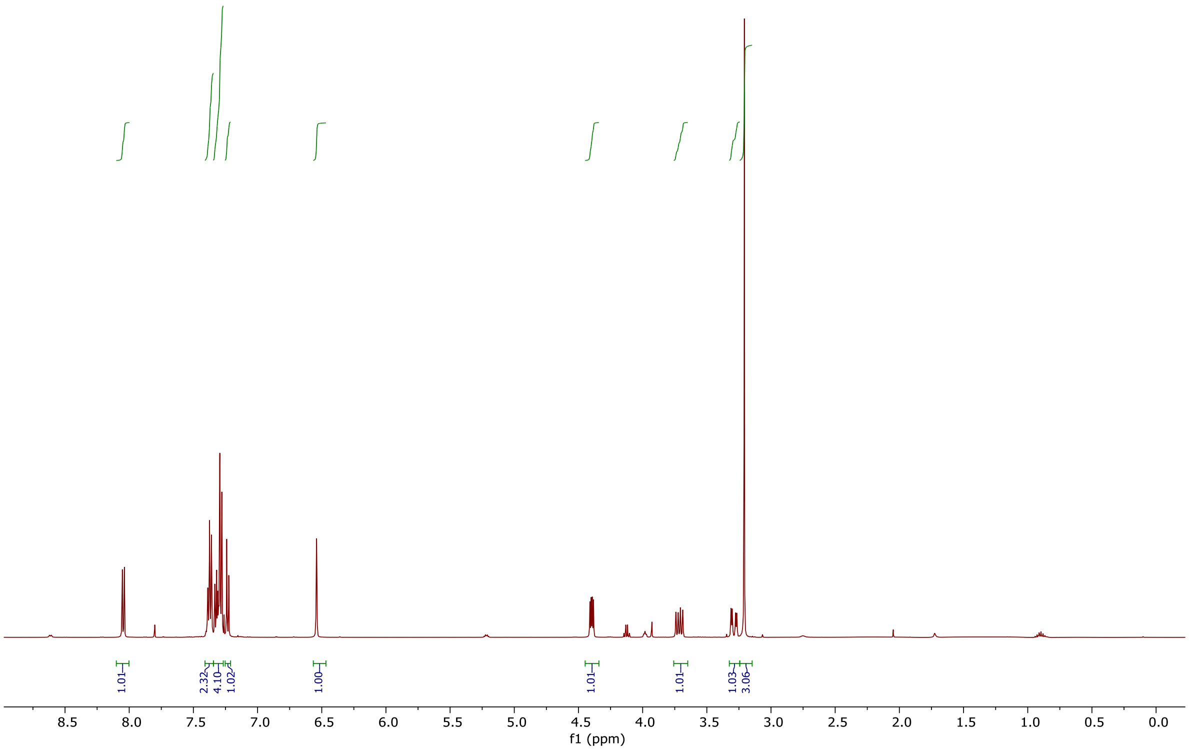

^{13}C-NMR: 3-oxo-2-phenyl-2,3-dihydro-1H-pyrrolo[1,2-a]indol-8-yl methanesulfonate (**7**)

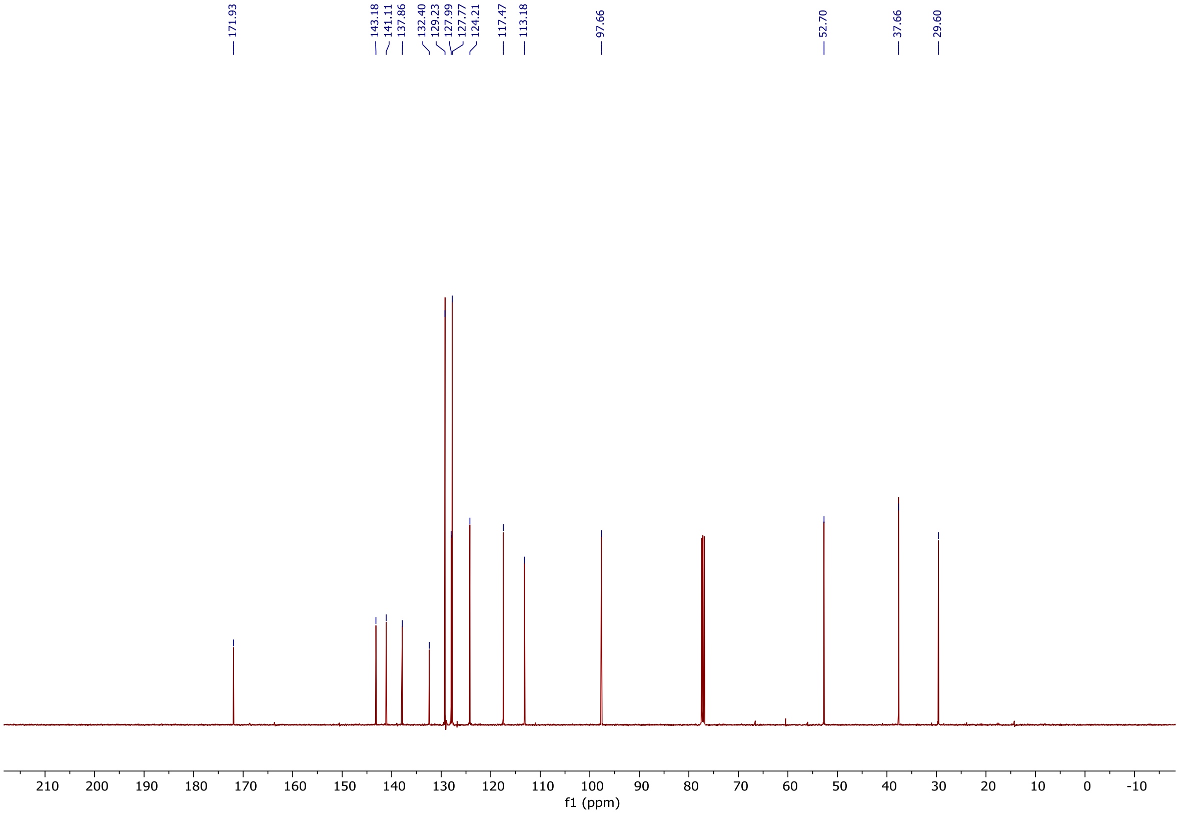

^{1}H-NMR: 1-(5-fluoro-1H-indol-1-yl)-2-phenylprop-2-en-1-one (**S6**)

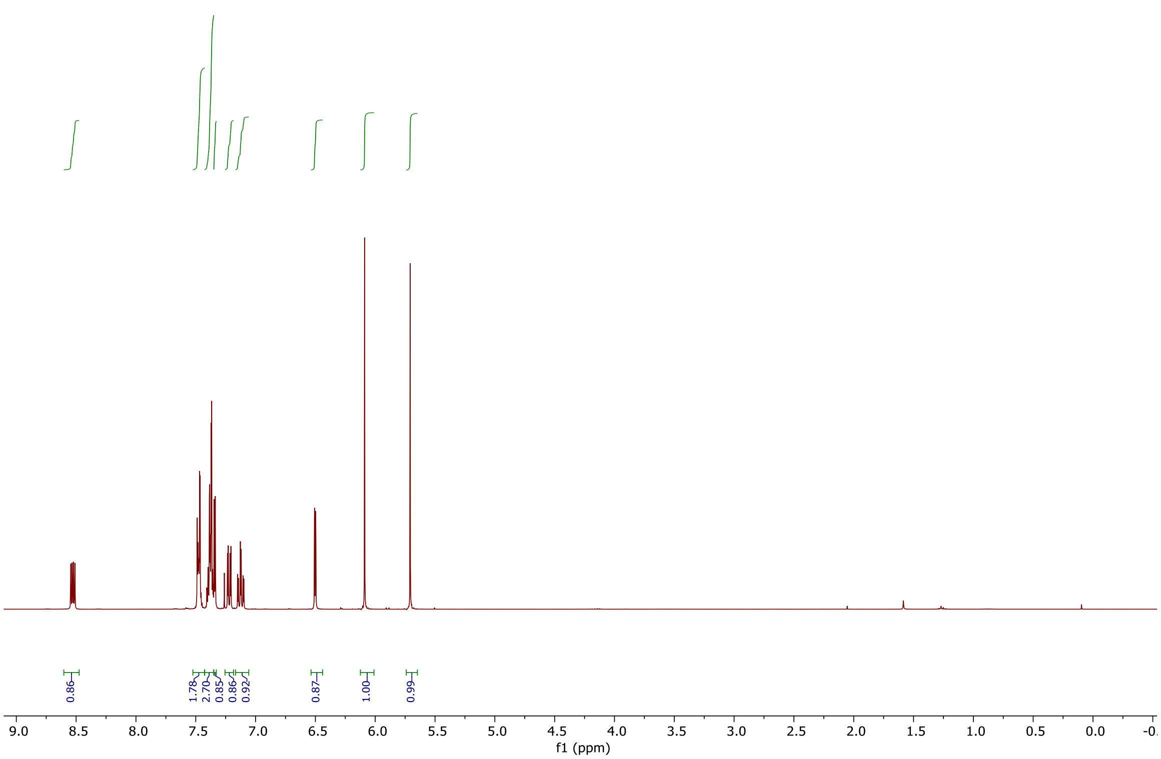

^{13}C-NMR: 1-(5-fluoro-1H-indol-1-yl)-2-phenylprop-2-en-1-one (**S6**)

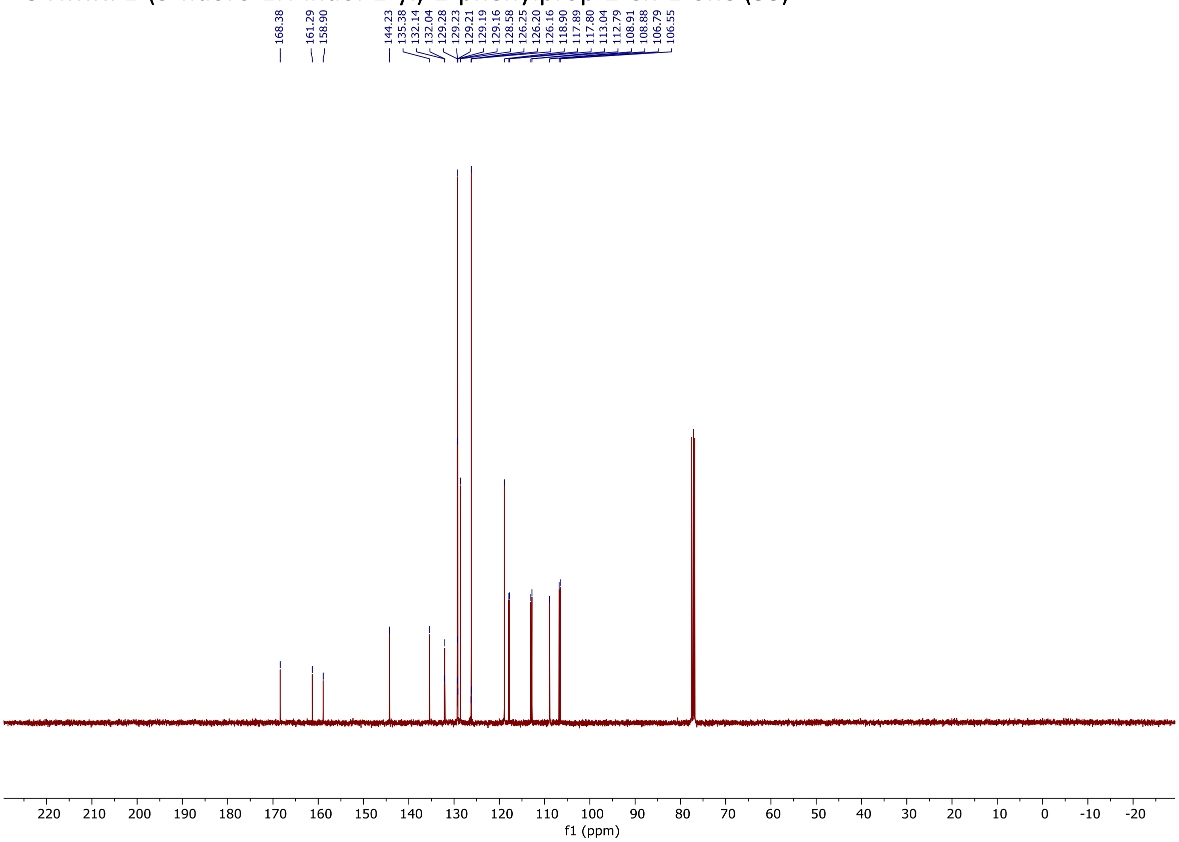

^{19}H-NMR: 1-(5-fluoro-1H-indol-1-yl)-2-phenylprop-2-en-1-one (**S6**)

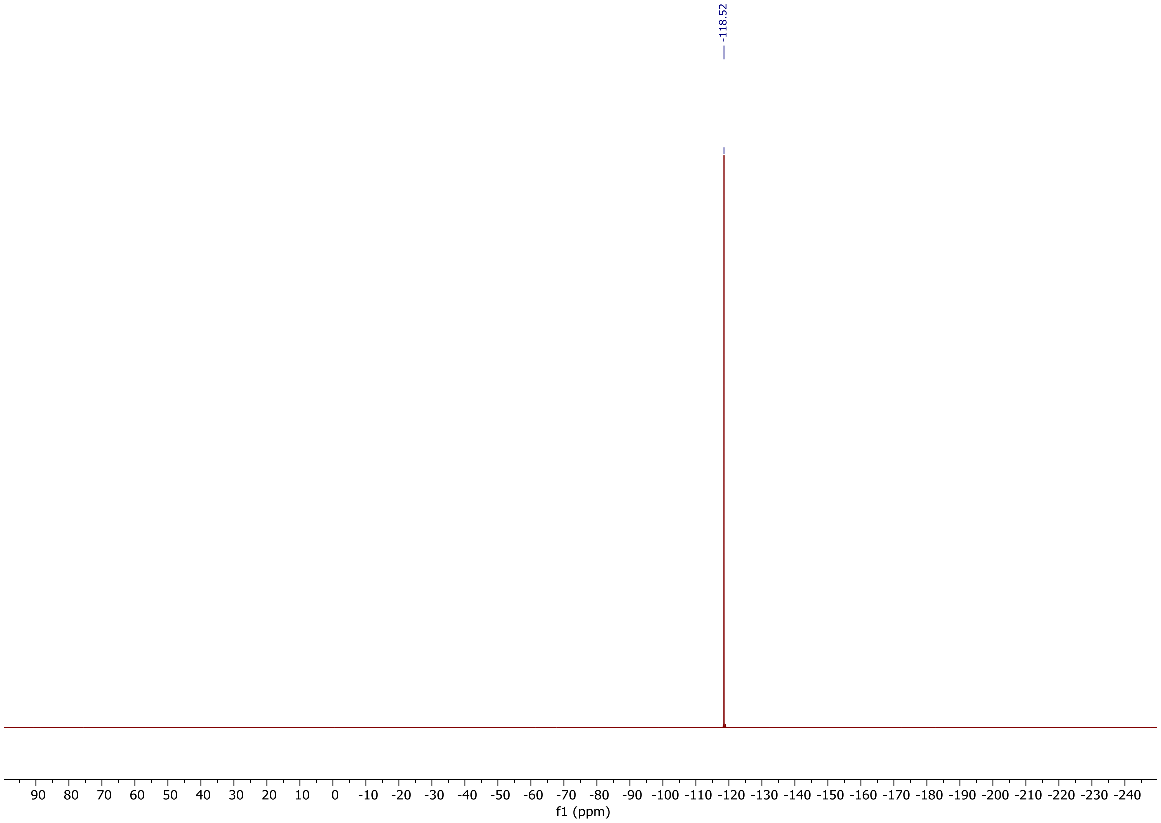

^{1}H-NMR: 7-floro-2-phenyl-1,2-dihydro-3H-pyrrolo[1,2-a]indol-3-one (**8**)

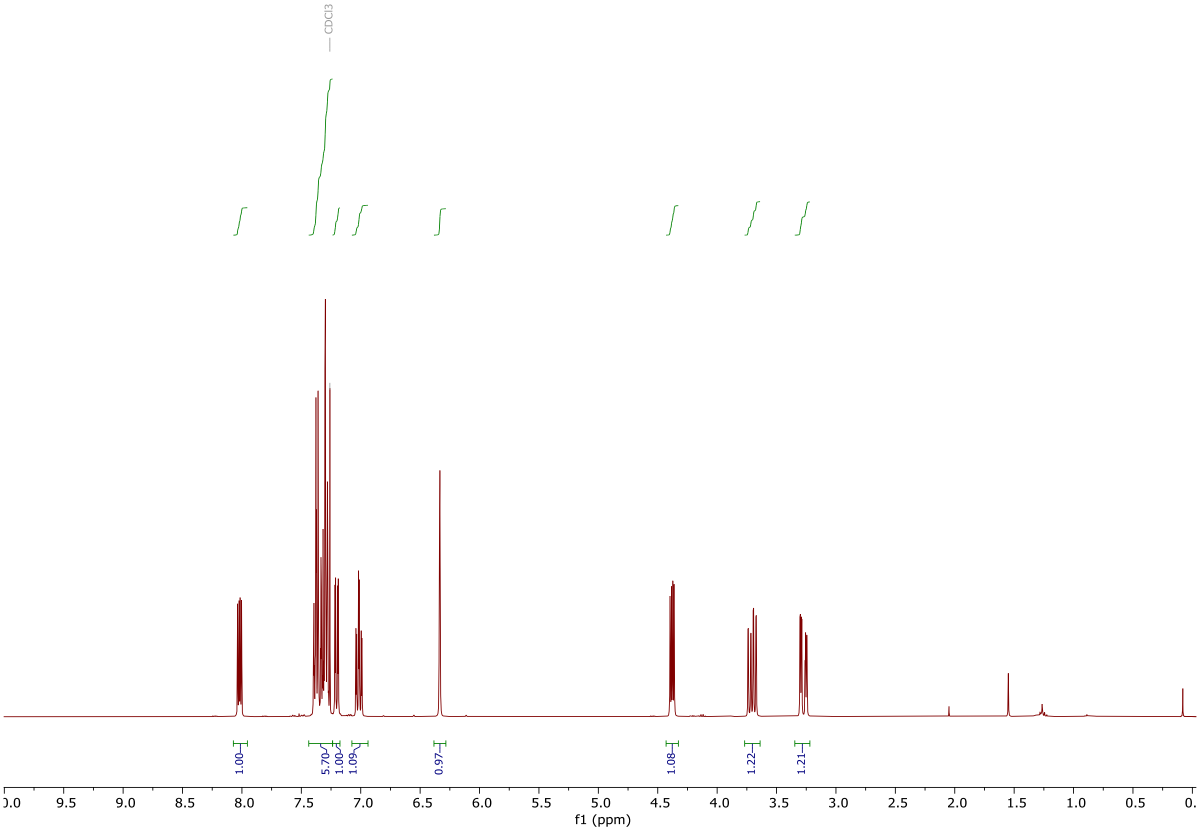

^{13}C-NMR: 7-floro-2-phenyl-1,2-dihydro-3H-pyrrolo[1,2-a]indol-3-one (**8**)

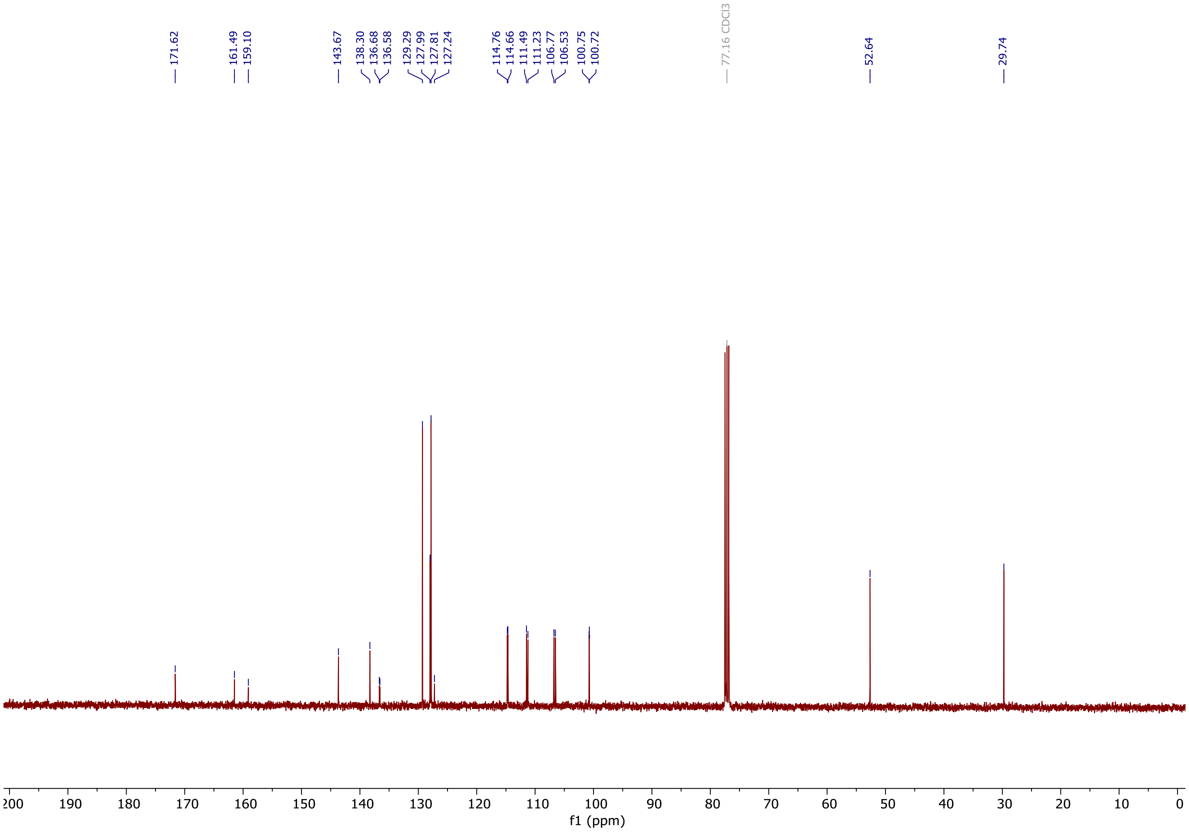

^{19}H-NMR: 7-floro-2-phenyl-1,2-dihydro-3H-pyrrolo[1,2-a]indol-3-one (**8**)

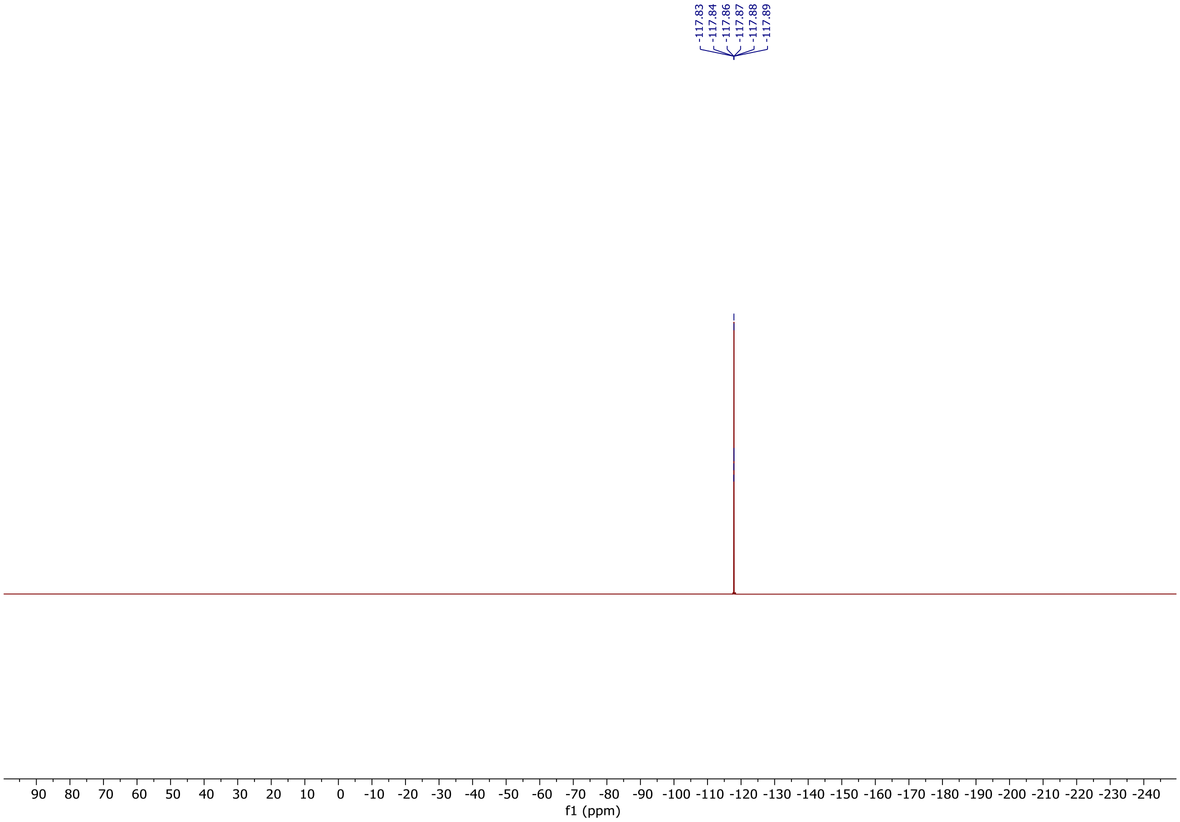

^{1}H-NMR: 1-(5-bromo-1H-indol-1-yl)-2-phenylprop-2-en-1-one (**S7**)

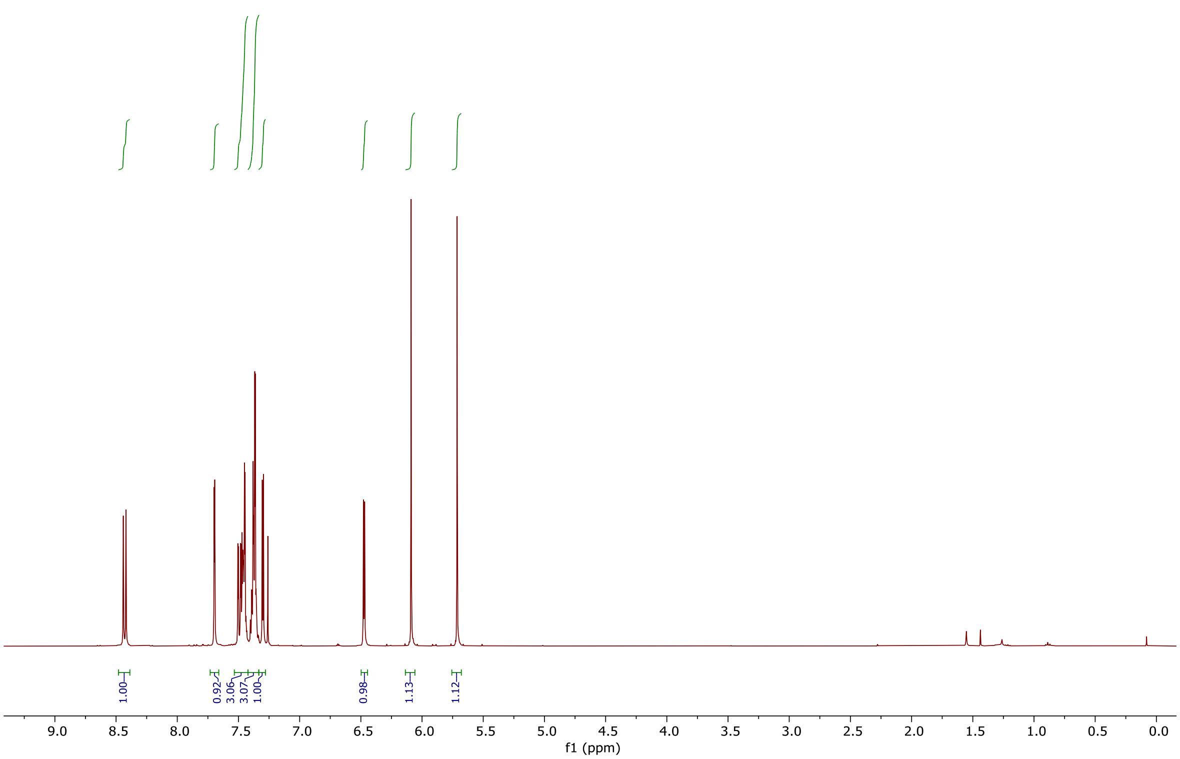

^{13}C-NMR: 1-(5-bromo-1H-indol-1-yl)-2-phenylprop-2-en-1-one (**S7**)

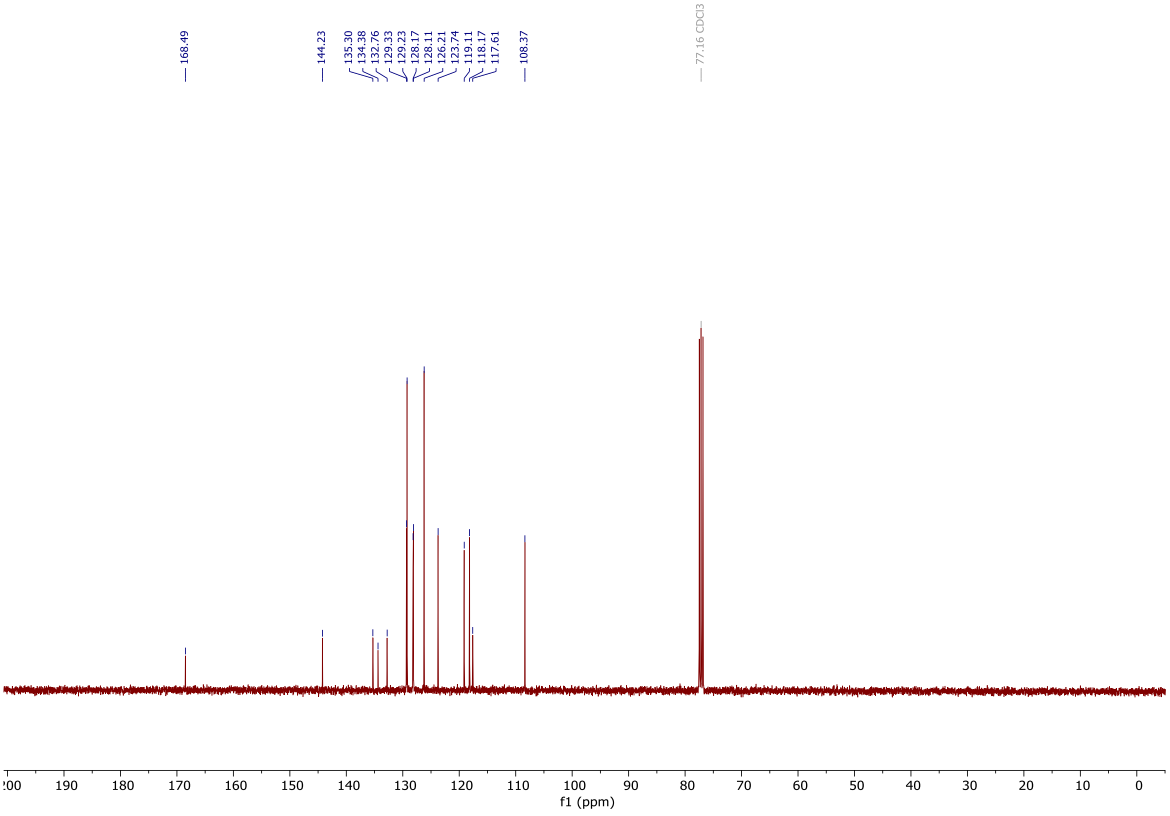

^{1}H-NMR: 7-bromo-2-phenyl-1,2-dihydro-3H-pyrrolo[1,2-a]indol-3-one (**9**)

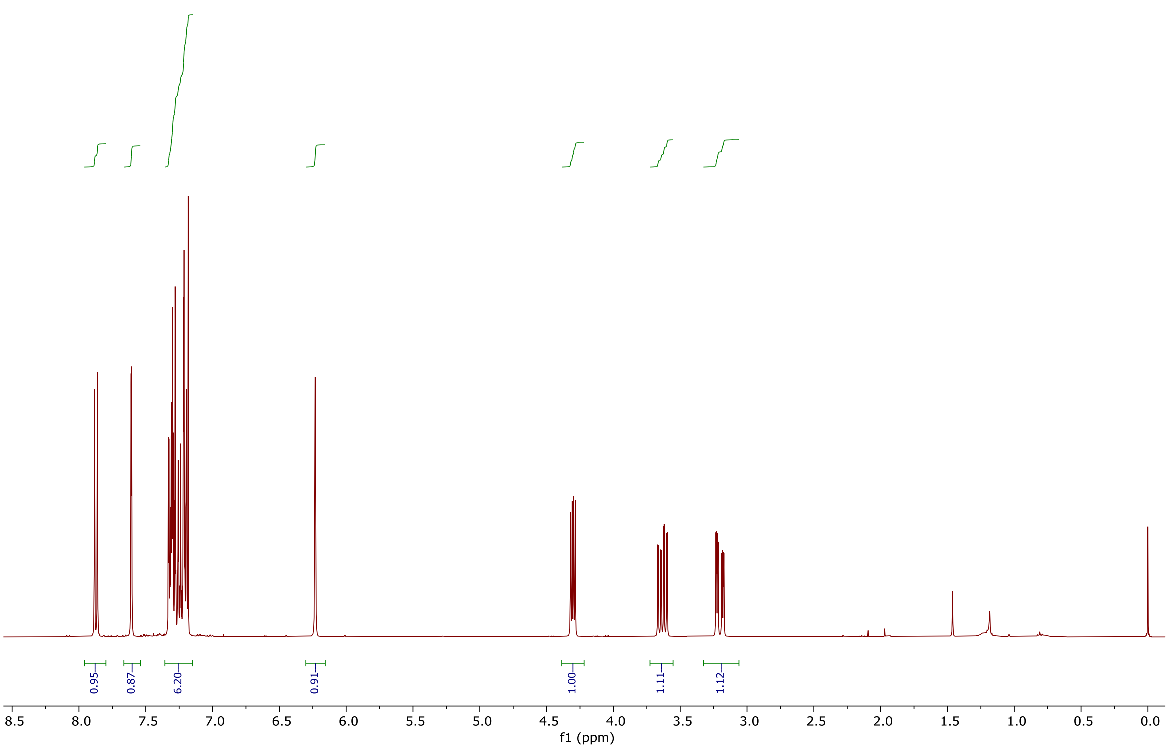

^{13}C-NMR: 7-bromo-2-phenyl-1,2-dihydro-3H-pyrrolo[1,2-a]indol-3-one (**9**)

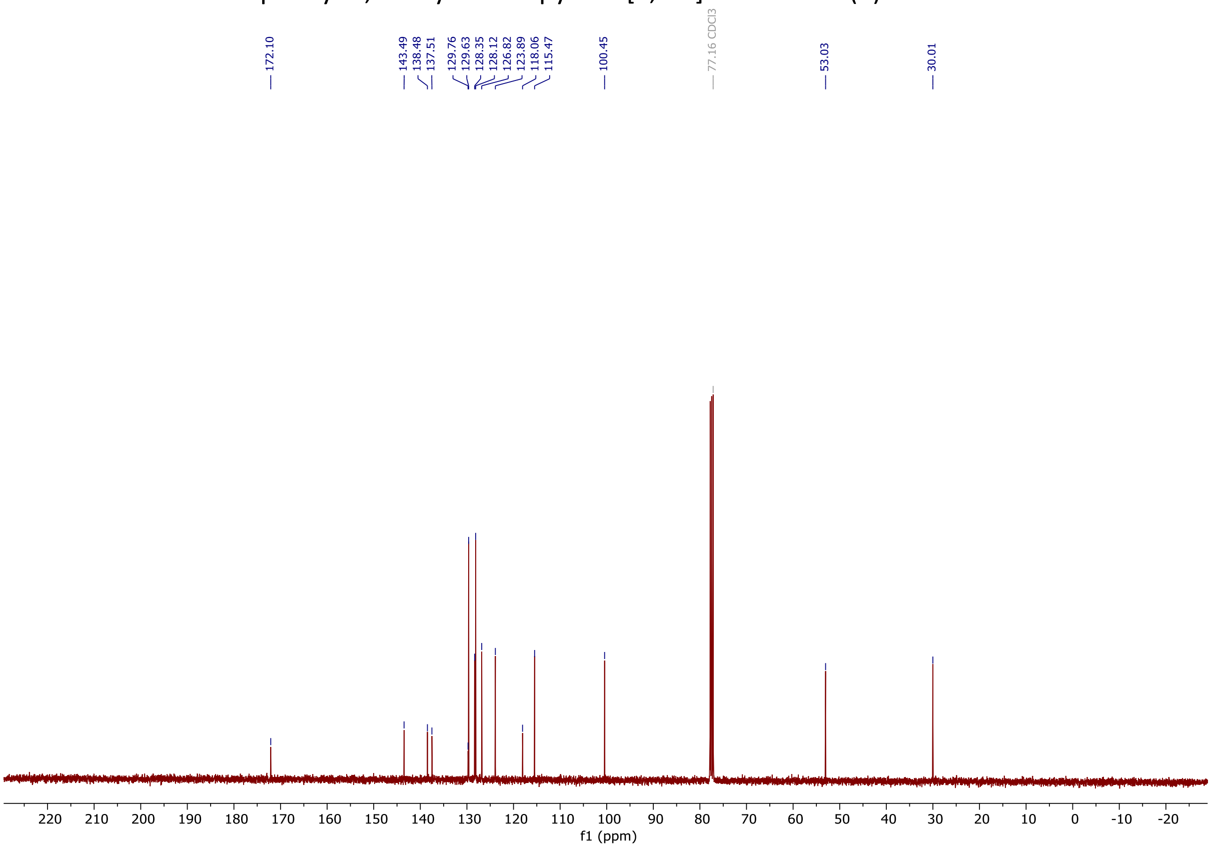

^{1}H-NMR: 1-(5-iodo-1H-indol-1-yl)-2-phenylprop-2-en-1-one (**S8**)

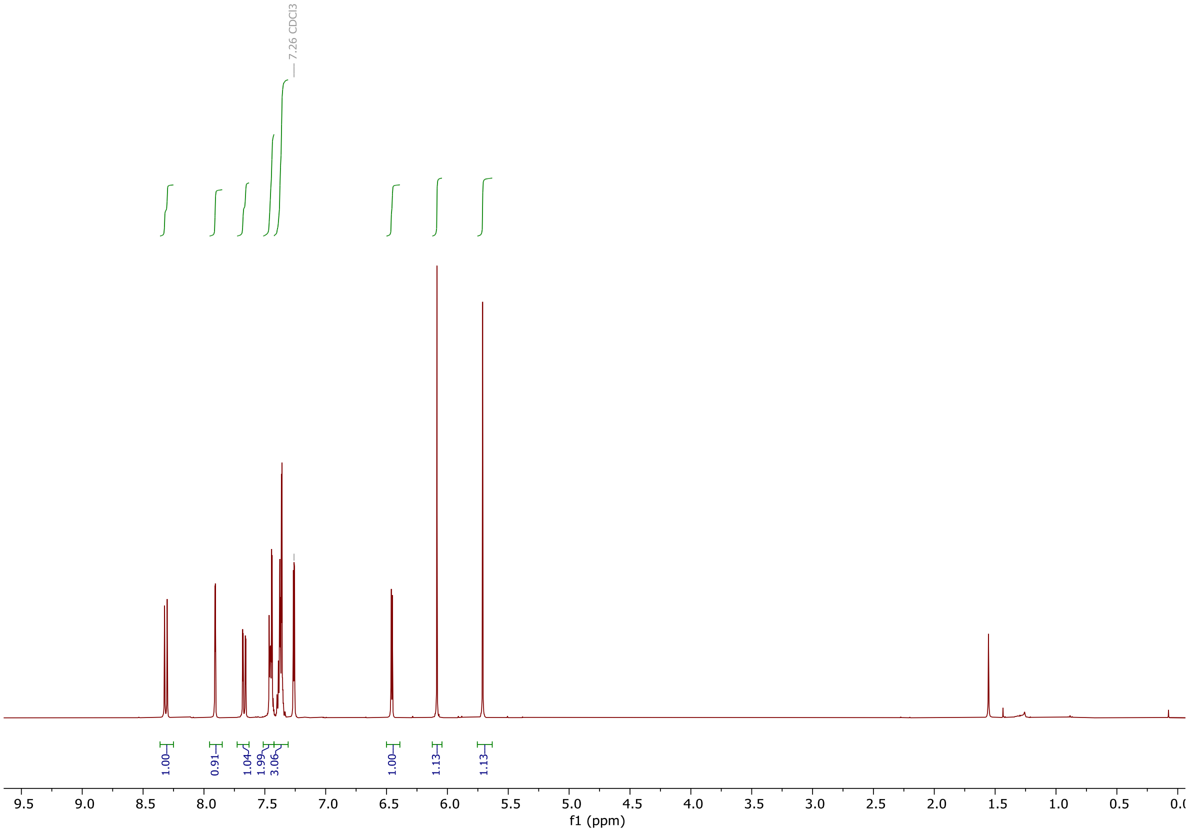

^{13}C-NMR: 1-(5-iodo-1H-indol-1-yl)-2-phenylprop-2-en-1-one (**S8**)

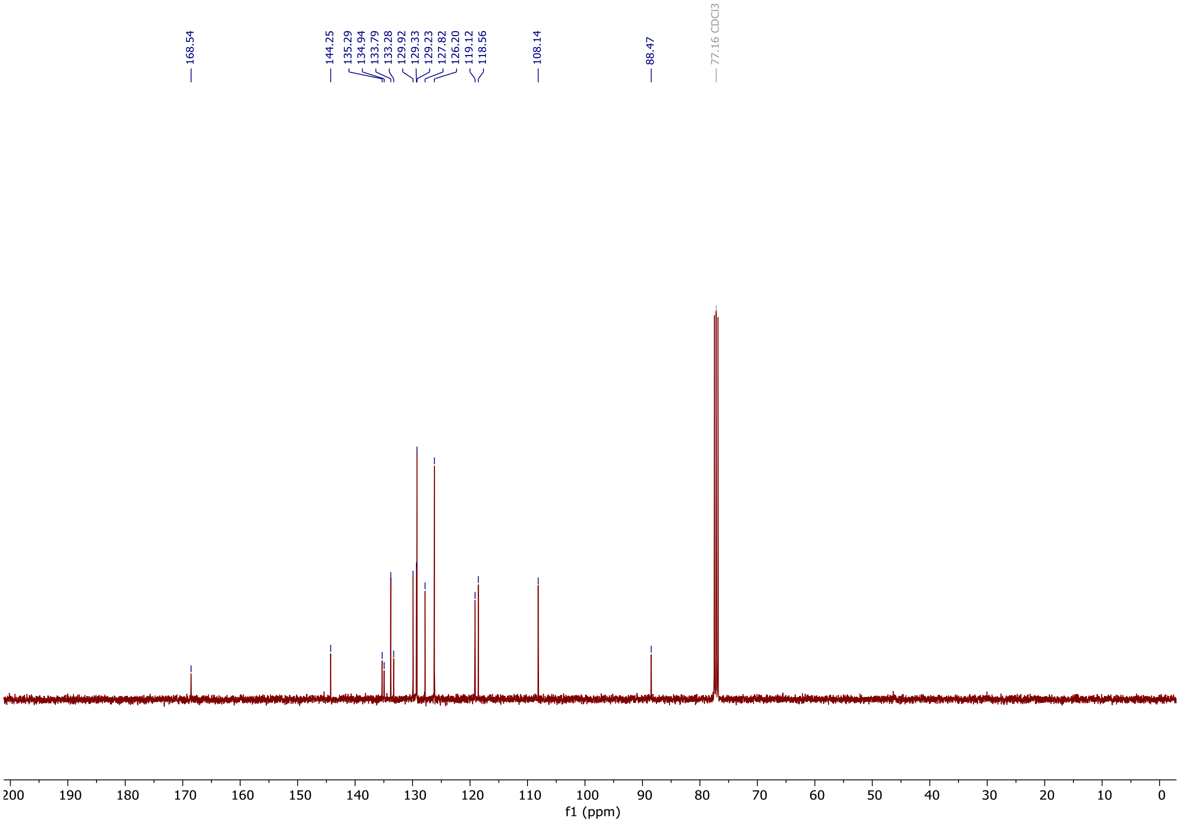

^{1}H-NMR: 7-iodo-2-phenyl-1,2-dihydro-3H-pyrrolo[1,2-a]indol-3-one (**10**)

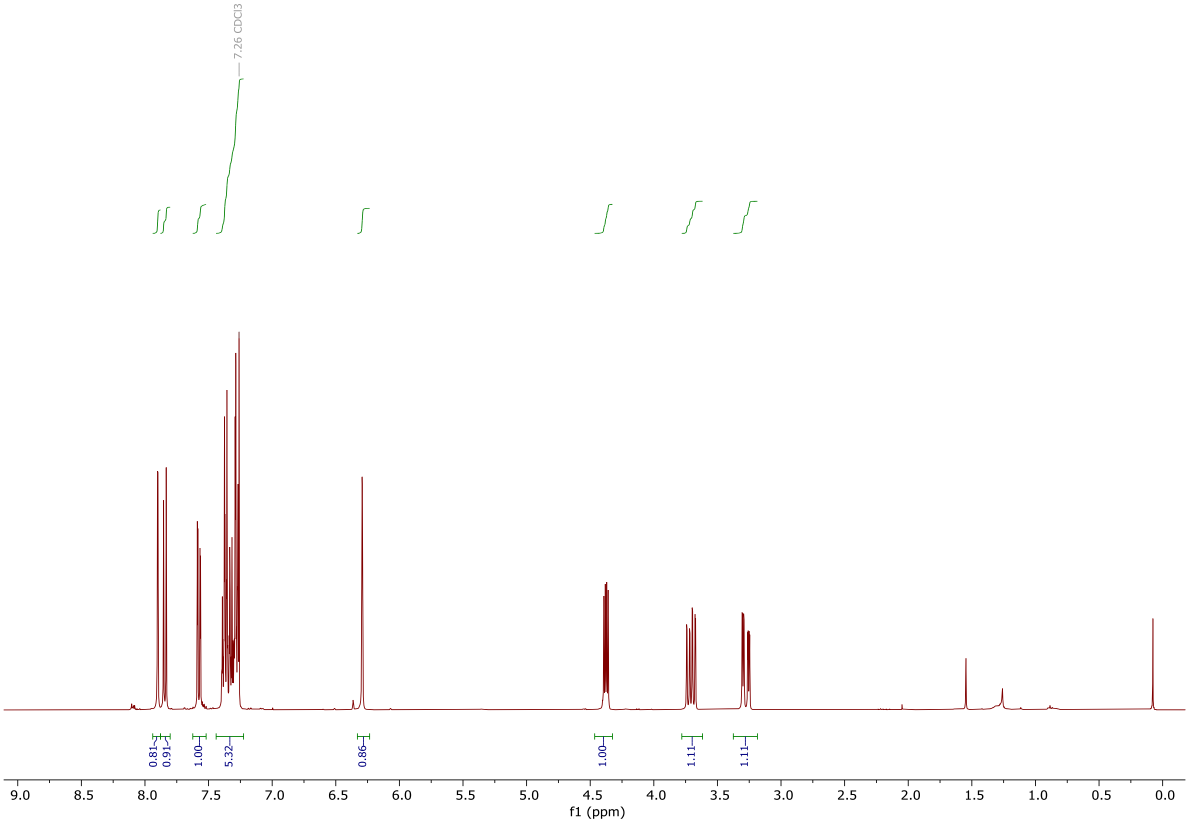

^{13}C-NMR: 7-iodo-2-phenyl-1,2-dihydro-3H-pyrrolo[1,2-a]indol-3-one (**10**)

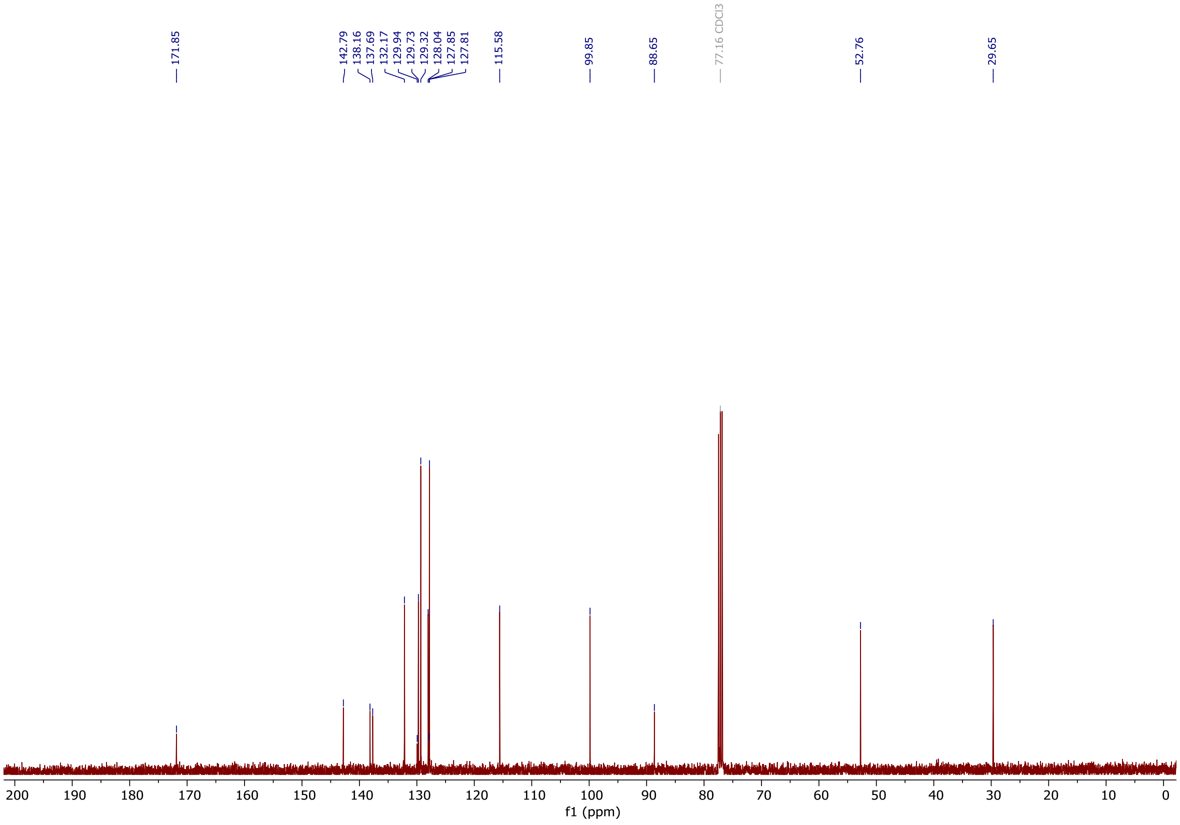

^{1}H-NMR: methyl 1-(2-phenylacryloyl)-1H-indole-5-carboxylate (**S9**)

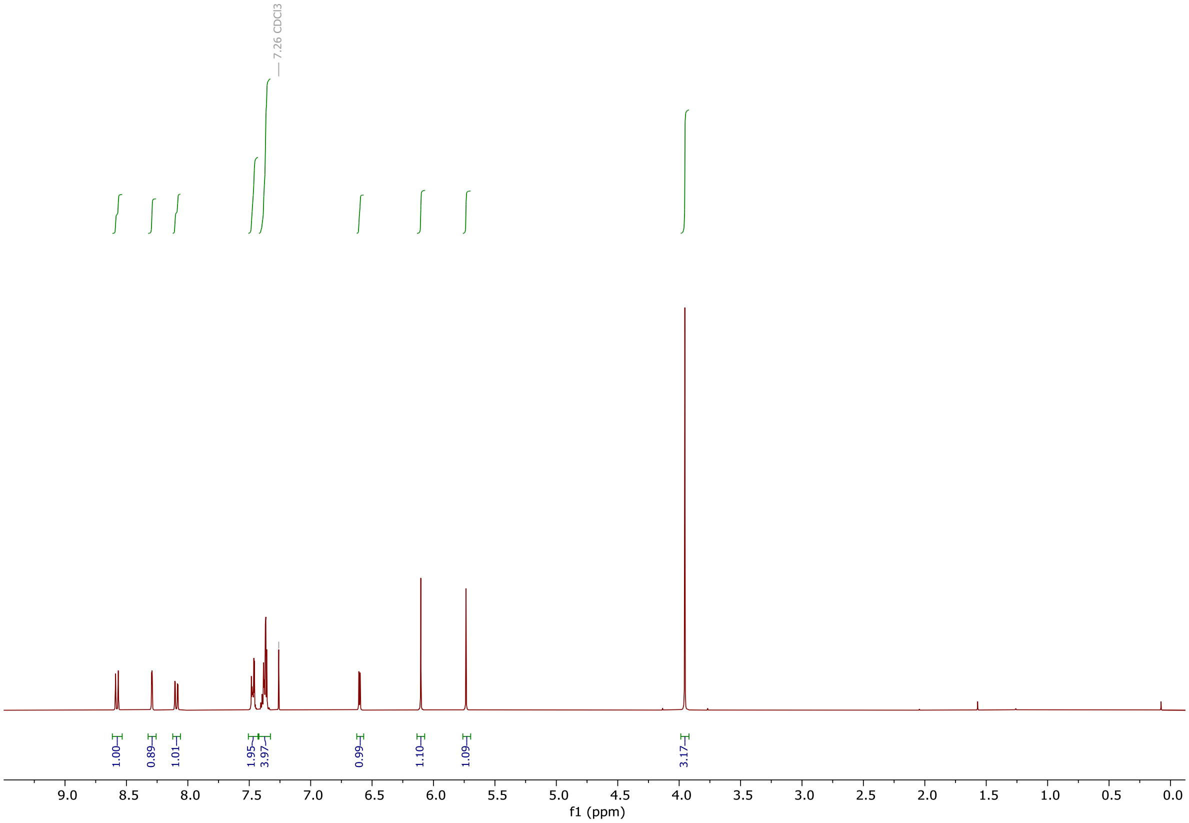

^{13}C-NMR: methyl 1-(2-phenylacryloyl)-1H-indole-5-carboxylate (**S9**)

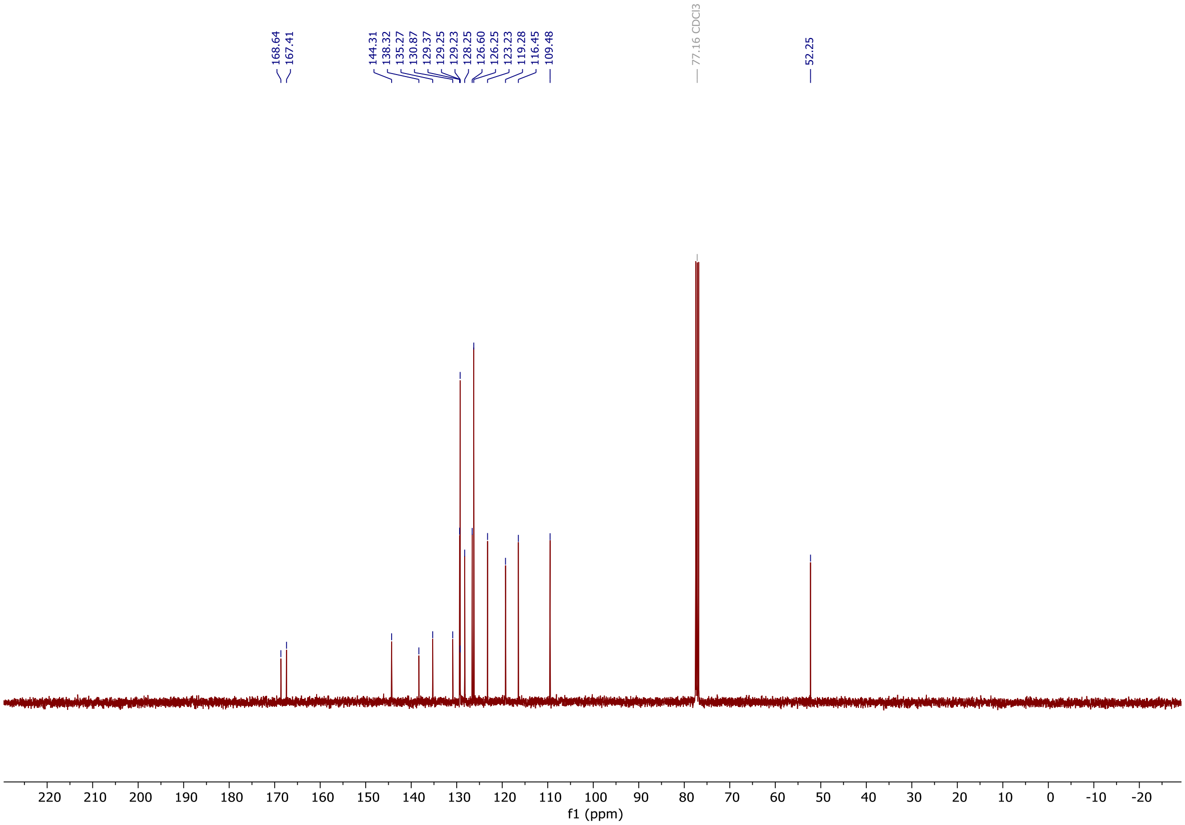

^{1}H-NMR: methyl 3-oxo-2-phenyl-2,3-dihydro-1H-pyrrolo[1,2-a]indole-7-carboxylate (**11**)

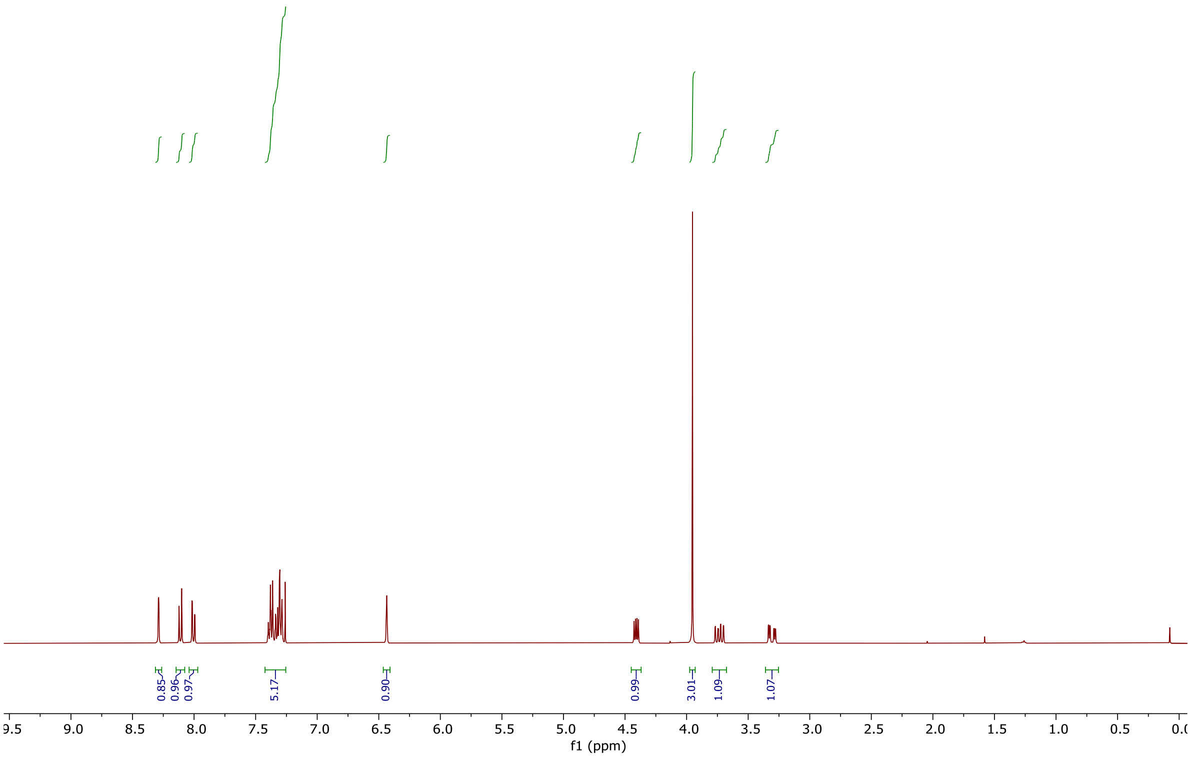

^{13}C-NMR: methyl 3-oxo-2-phenyl-2,3-dihydro-1H-pyrrolo[1,2-a]indole-7-carboxylate (**11**)

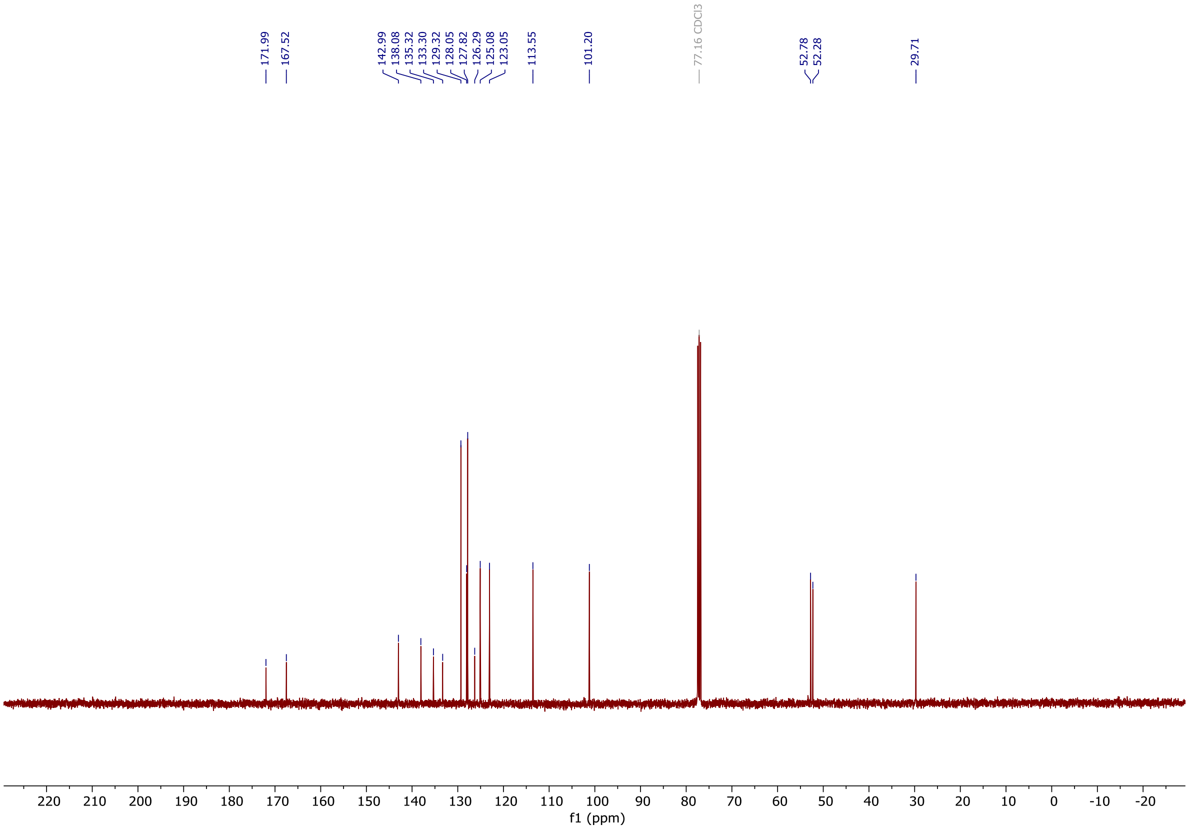

^{1}H-NMR: 1-(5-methoxy-1H-indol-1-yl)-2-phenylprop-2-en-1-one (**S10**)

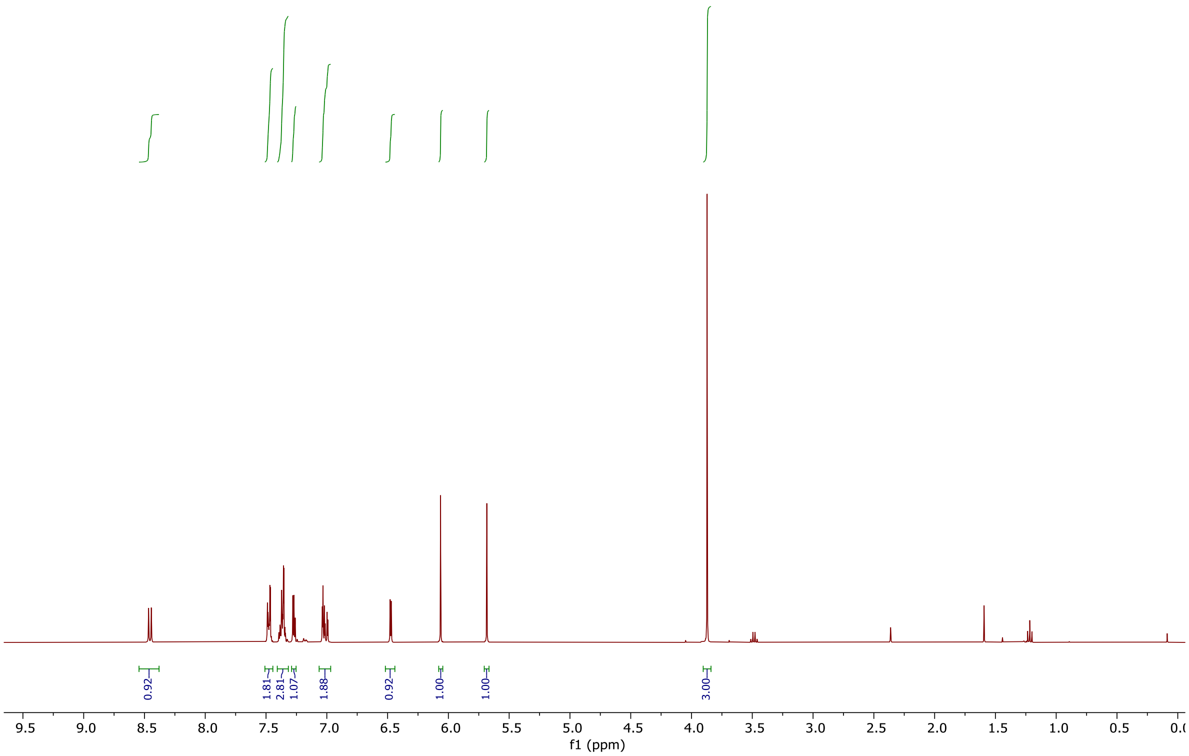

^{13}C-NMR: 1-(5-methoxy-1H-indol-1-yl)-2-phenylprop-2-en-1-one (**S10**)

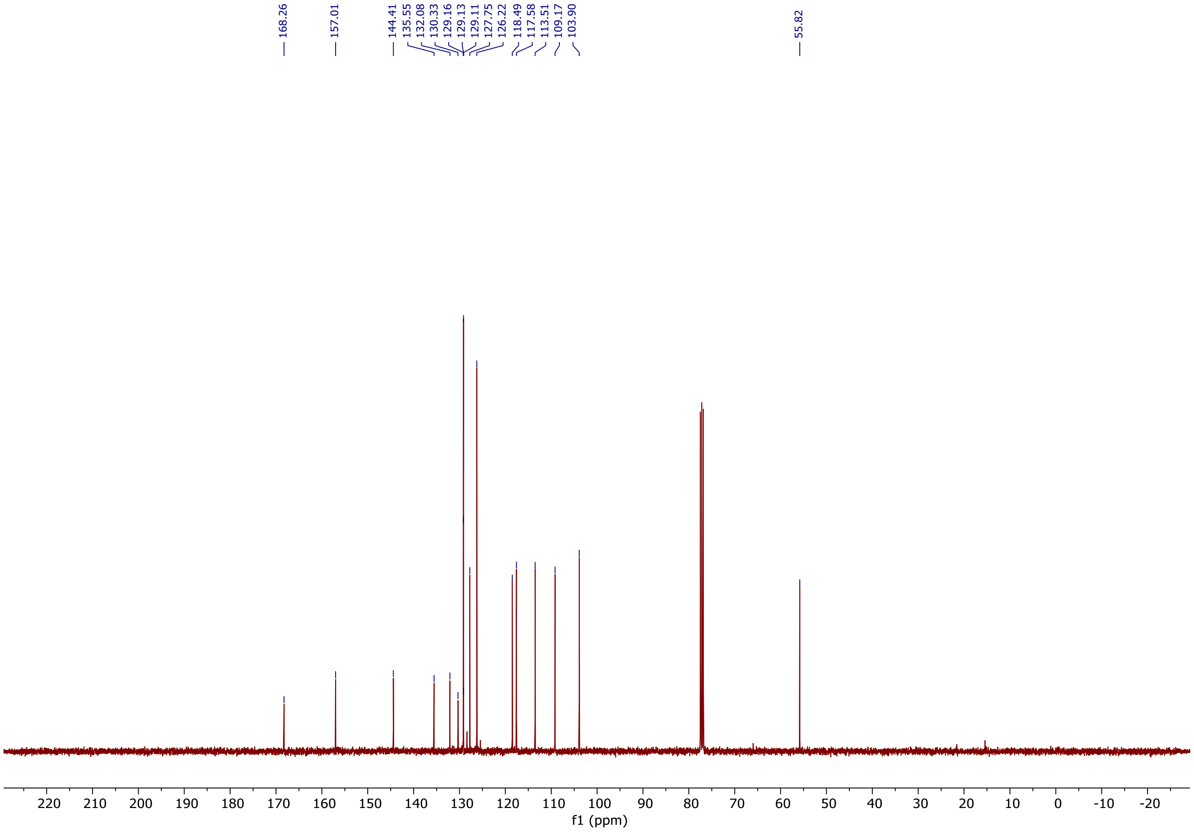

^{1}H-NMR: 7-methoxy-2-phenyl-1,2-dihydro-3H-pyrrolo[1,2-a]indol-3-one (**12**)

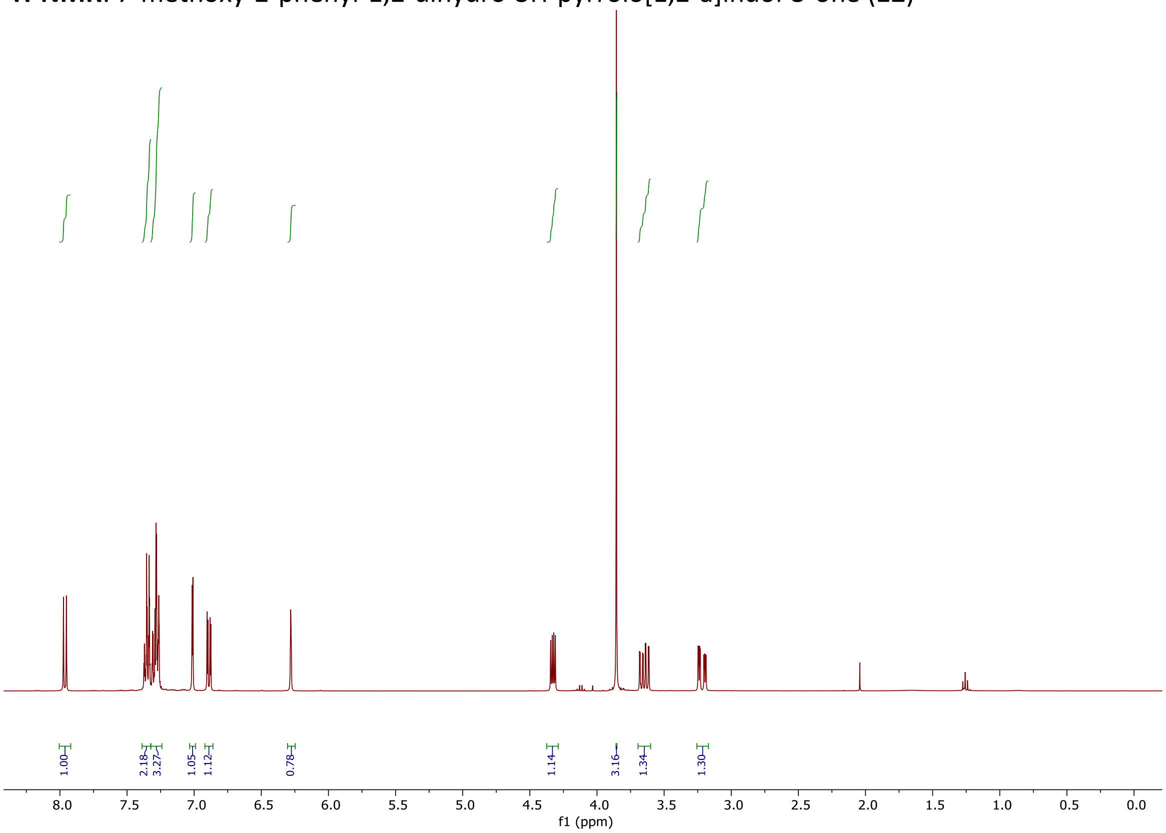

^{13}C-NMR: 7-methoxy-2-phenyl-1,2-dihydro-3H-pyrrolo[1,2-a]indol-3-one (**12**)

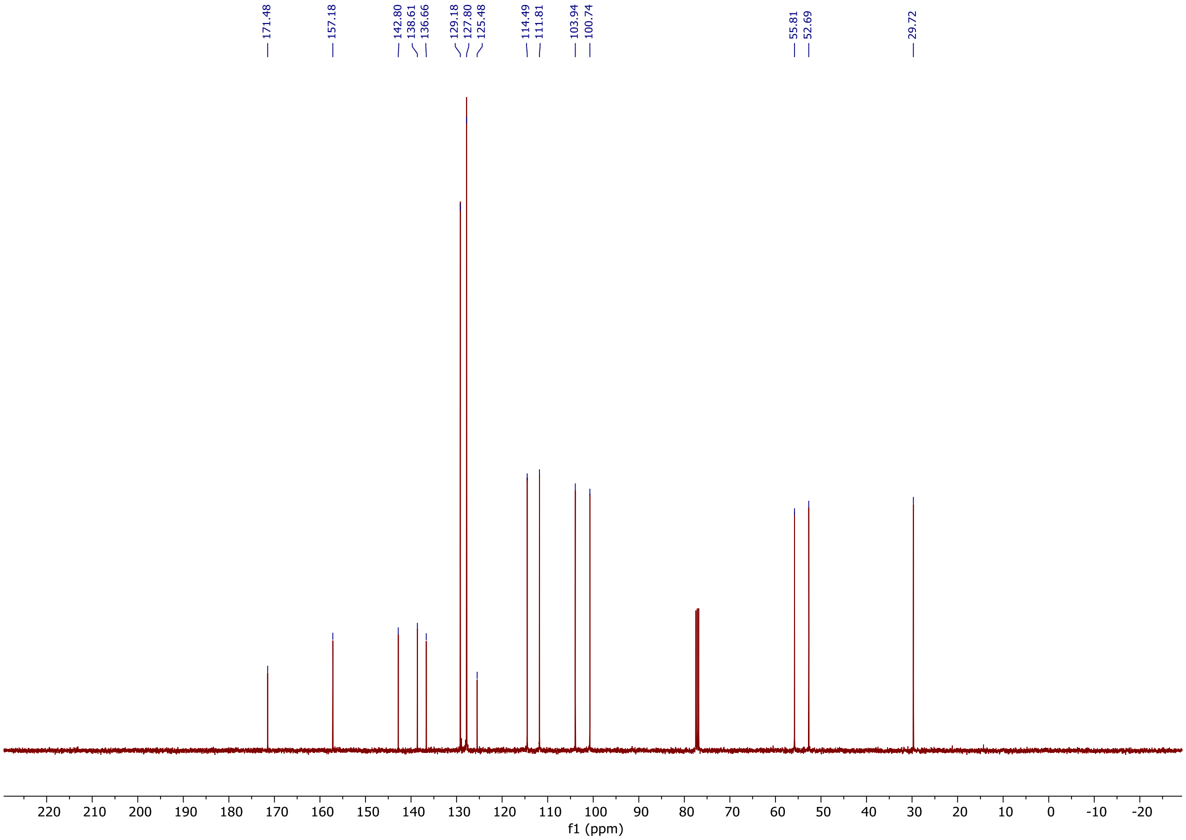

^{1}H-NMR: 2-phenyl-1-(6-(4,4,5,5-tetramethyl-1,3,2-dioxaborolan-2-yl)-1H-indol-1-yl)prop-2-en-1-one (**S11**)

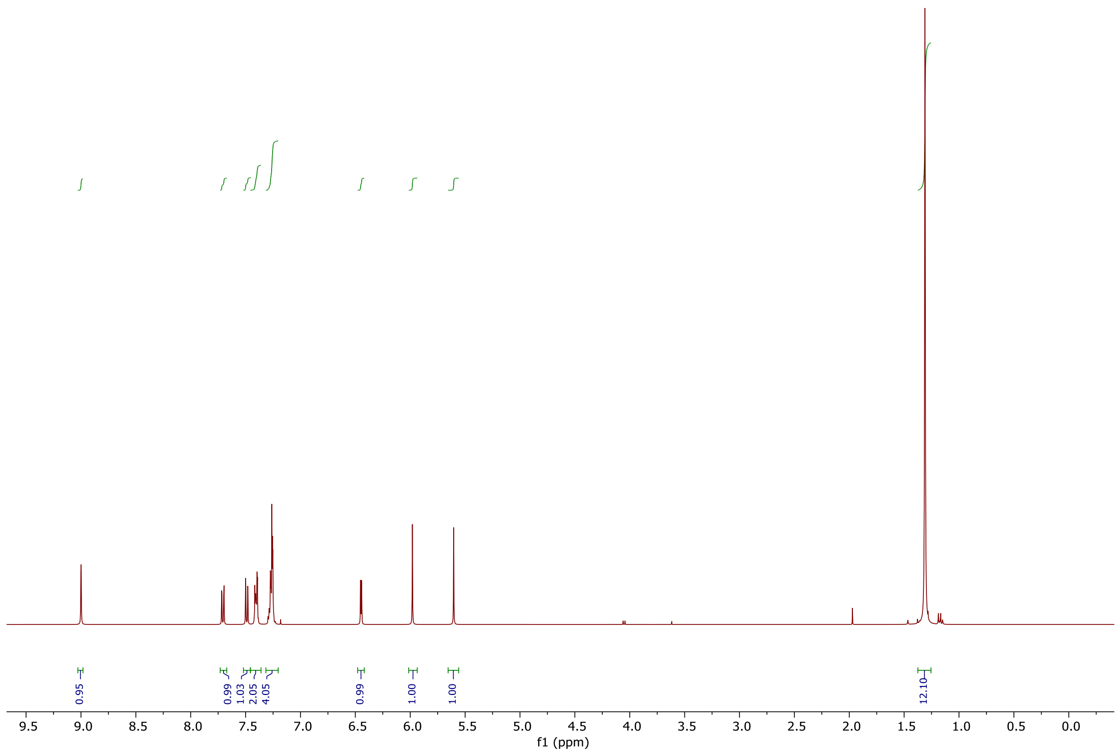

^{13}C-NMR: 2-phenyl-1-(6-(4,4,5,5-tetramethyl-1,3,2-dioxaborolan-2-yl)-1H-indol-1-yl)prop-2-en-1-one (**S11**)

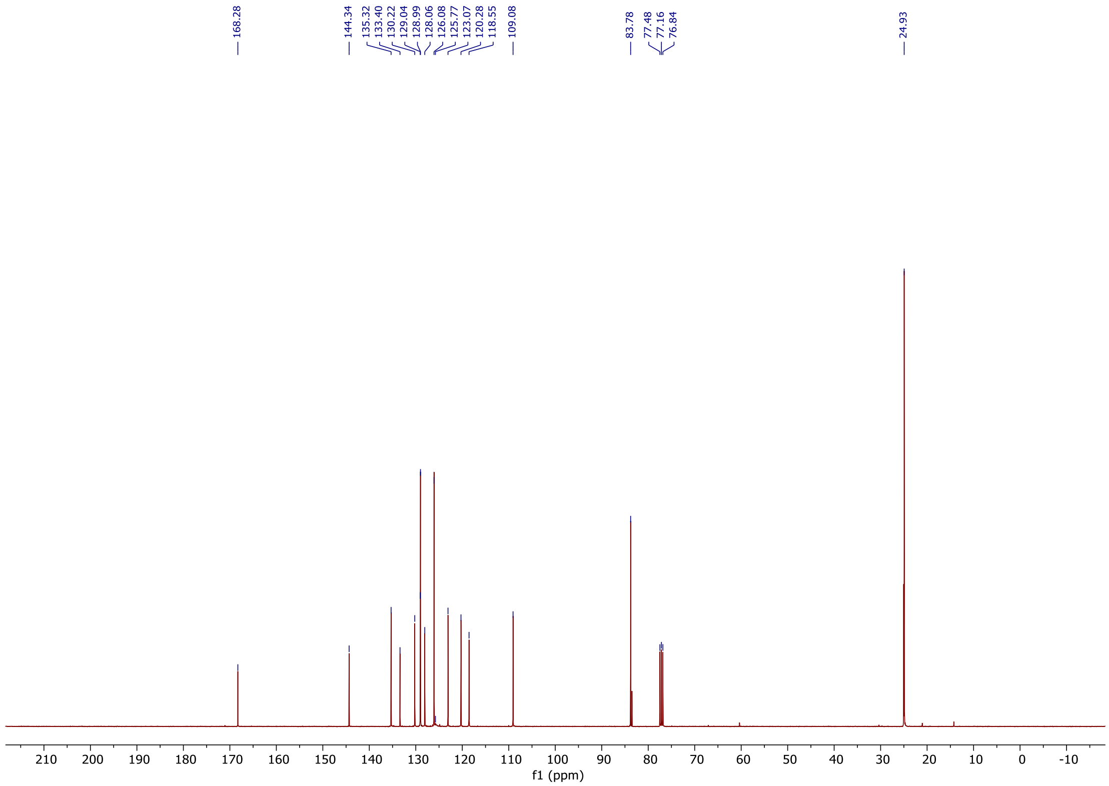

^{11}B-NMR: 2-phenyl-1-(6-(4,4,5,5-tetramethyl-1,3,2-dioxaborolan-2-yl)-1H-indol-1-yl)prop-2-en-1-one (**S11**)

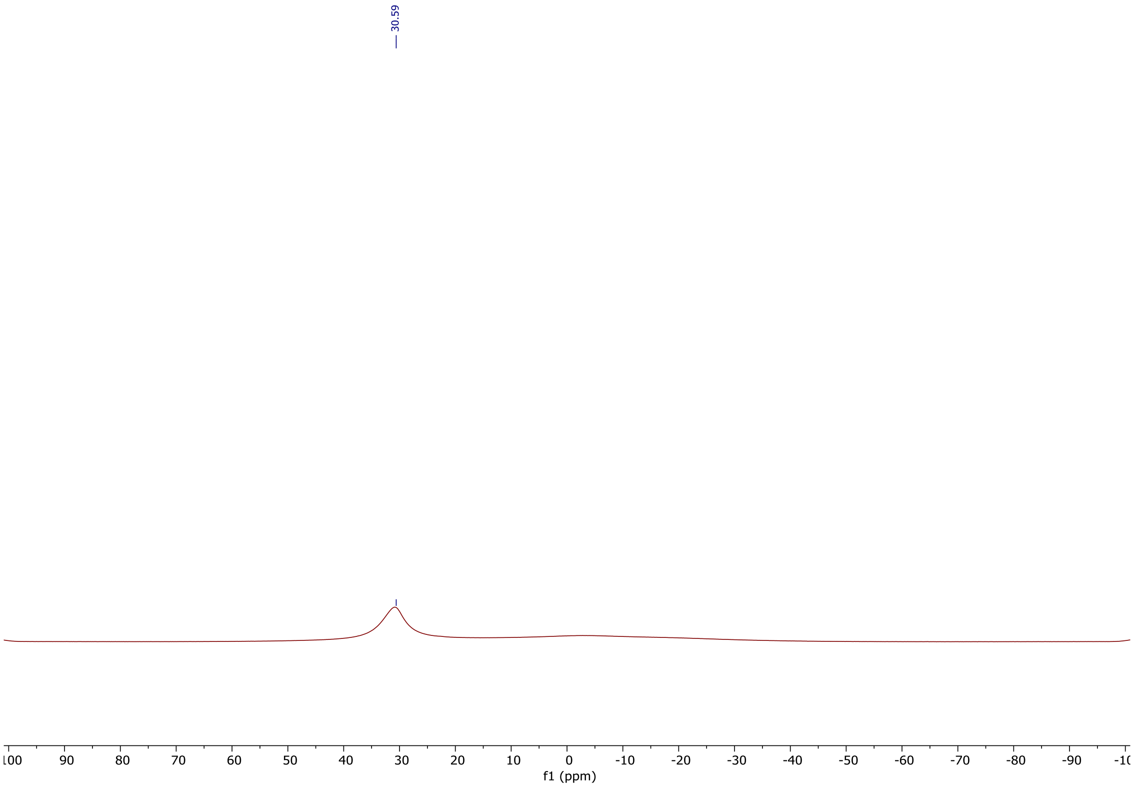

^{1}H-NMR: 2-phenyl-6-(4,4,5,5-tetramethyl-1,3,2-dioxaborolan-2-yl)-1,2-dihydro-3H-pyrrolo[1,2-a]indol-3-one (**13**)

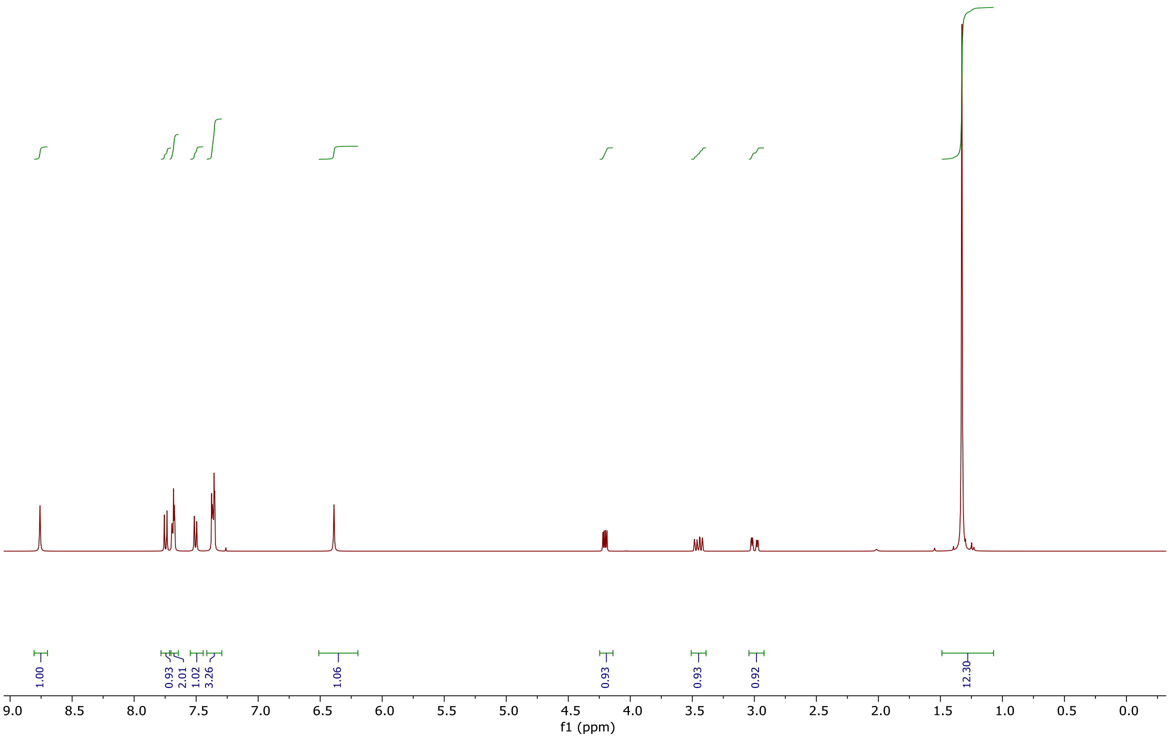

^{13}C-NMR: 2-phenyl-6-(4,4,5,5-tetramethyl-1,3,2-dioxaborolan-2-yl)-1,2-dihydro-3H-pyrrolo[1,2-a]indol-3-one (**13**)

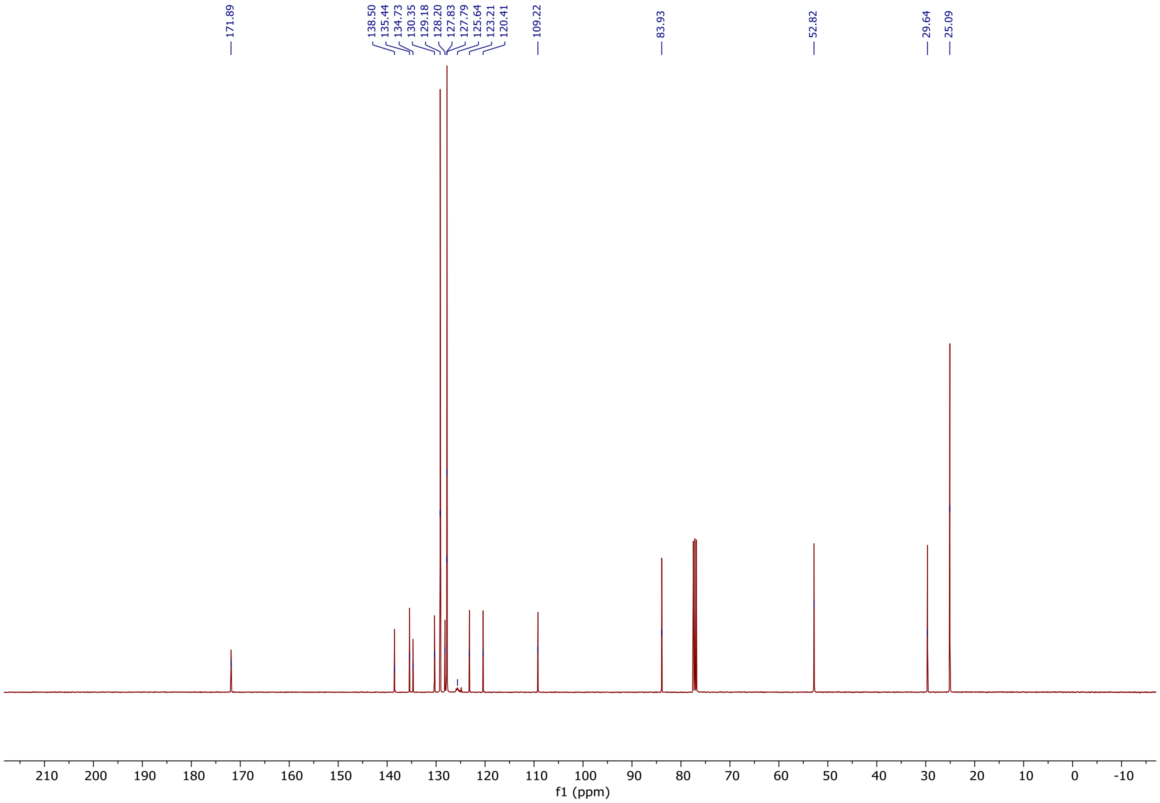

^{11}B-NMR: 2-phenyl-6-(4,4,5,5-tetramethyl-1,3,2-dioxaborolan-2-yl)-1,2-dihydro-3H-pyrrolo[1,2-a]indol-3-one (**13**)

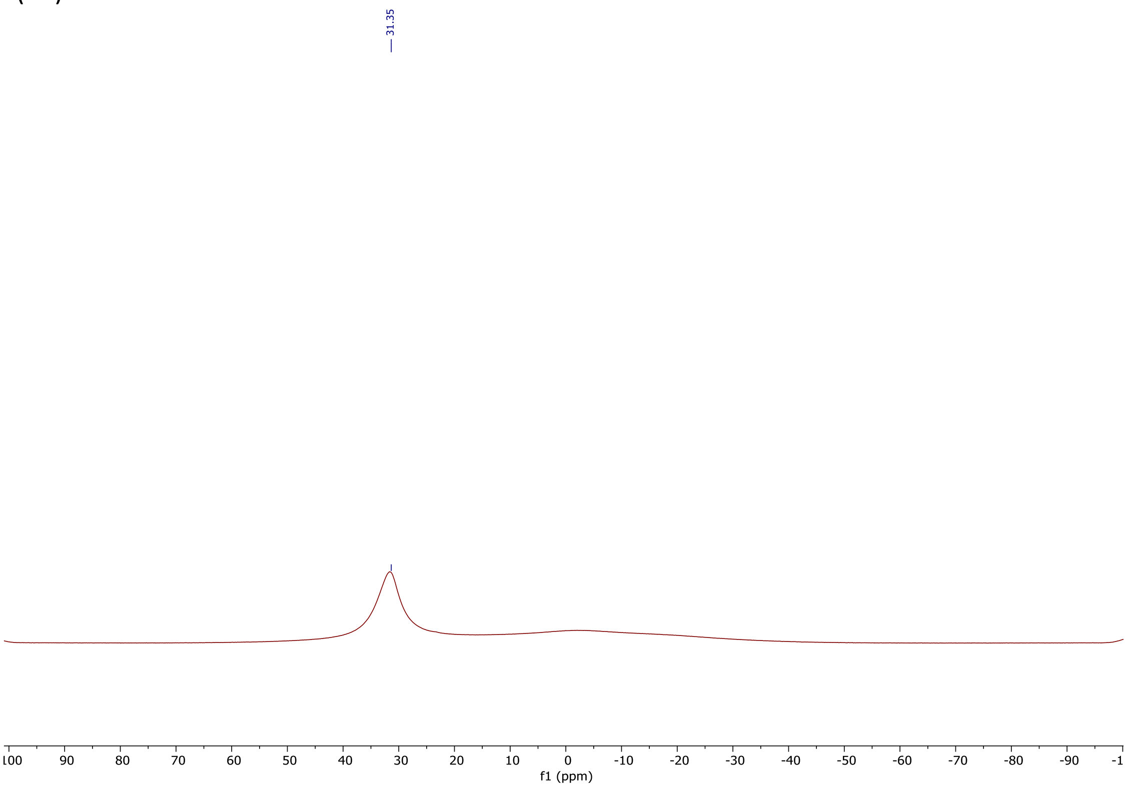

^{1}H-NMR: 1-(6-bromo-1H-indol-1-yl)-2-phenylprop-2-en-1-one (**S12**)

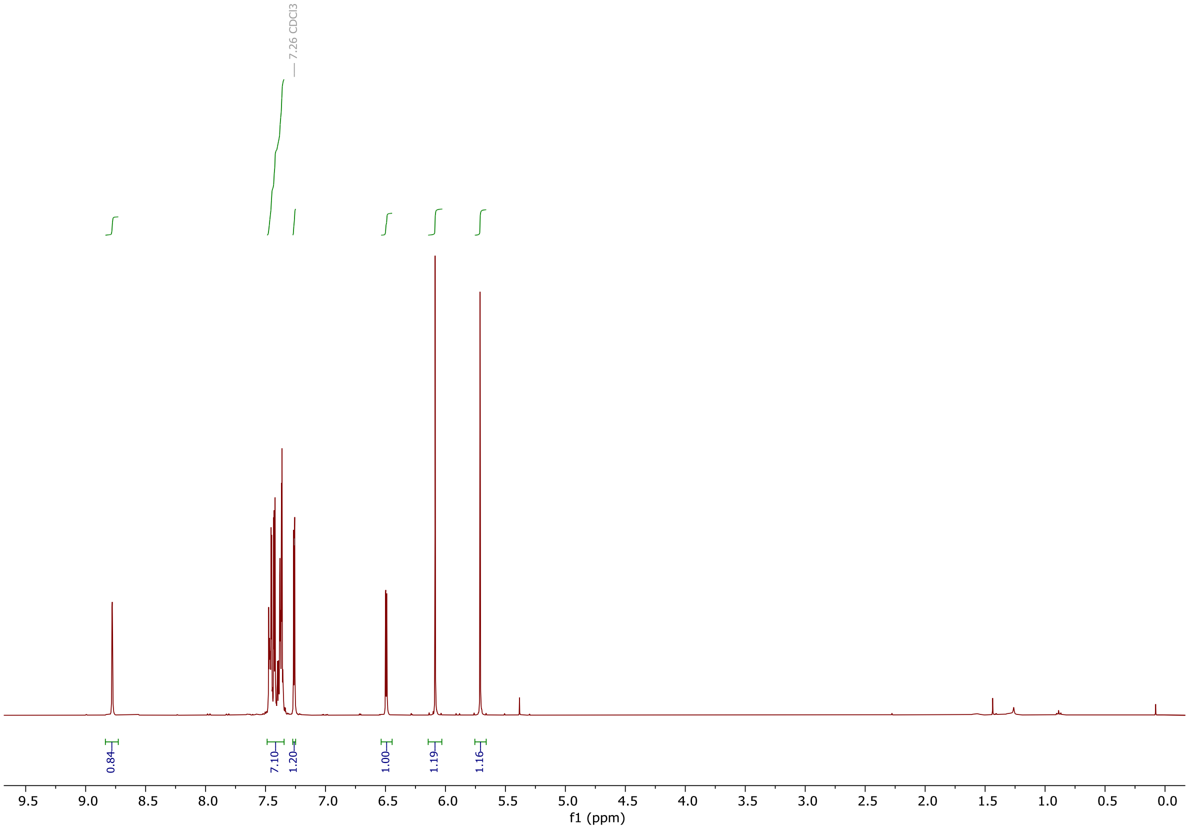

^{13}C-NMR: 1-(6-bromo-1H-indol-1-yl)-2-phenylprop-2-en-1-one (**S12**)

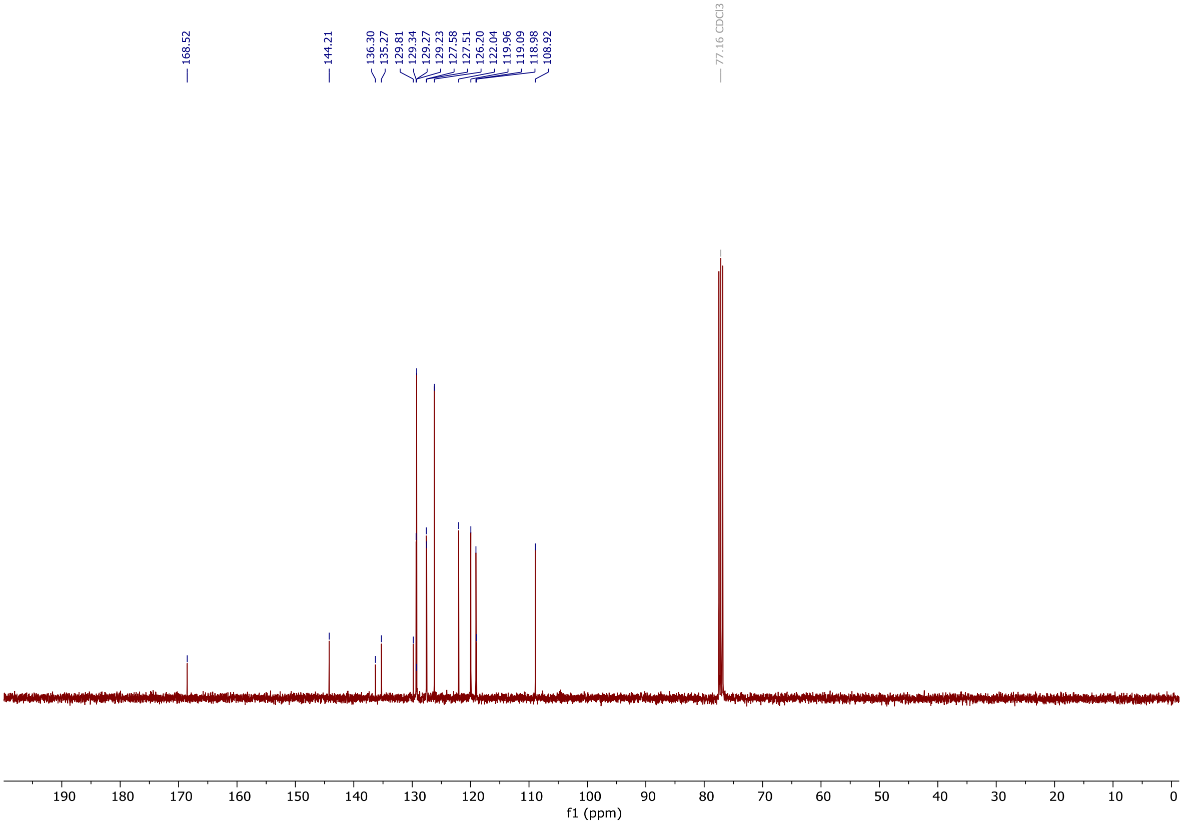

^{1}H-NMR: 6-bromo-2-phenyl-1,2-dihydro-3H-pyrrolo[1,2-a]indol-3-one (**14**)

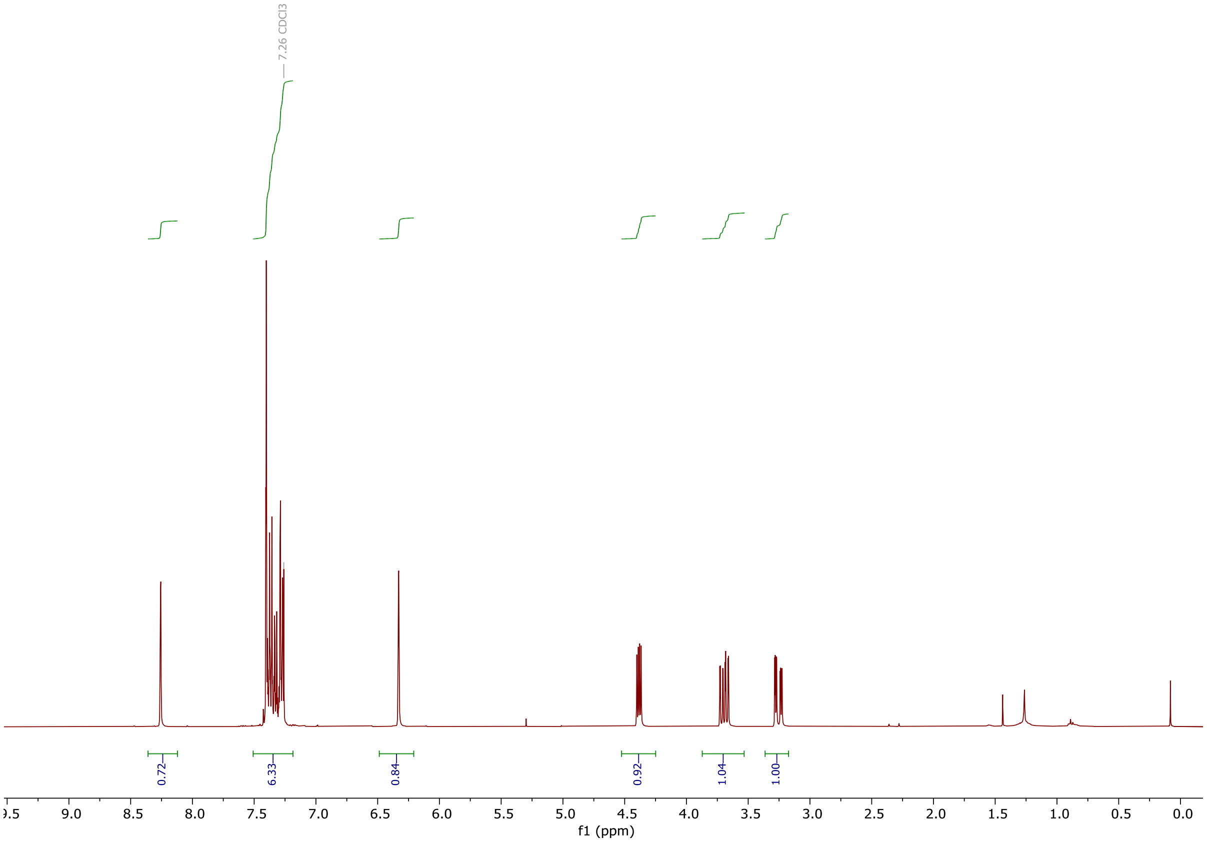

^{13}C-NMR: 6-bromo-2-phenyl-1,2-dihydro-3H-pyrrolo[1,2-a]indol-3-one (**14**)

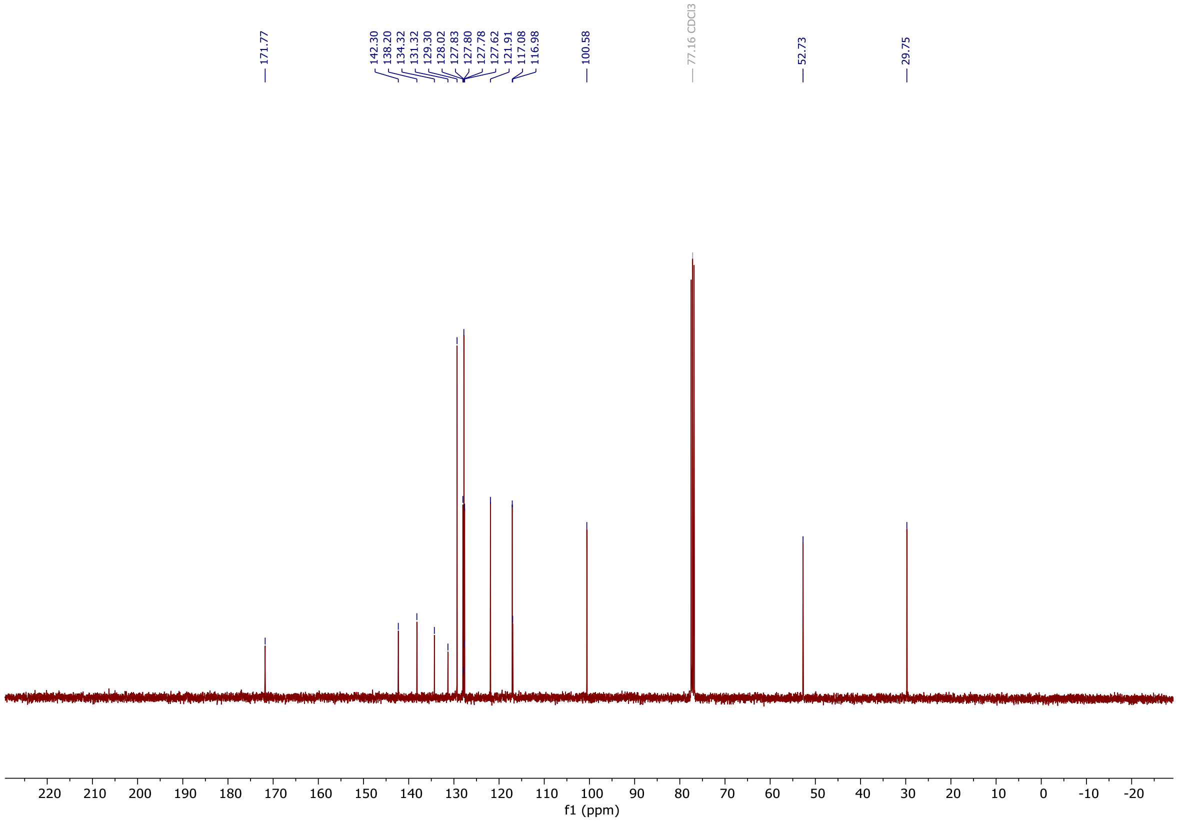

^{1}H-NMR: 2-phenyl-1-(6-(trifluoromethyl)-1H-indol-1-yl)prop-2-en-1-one (**S13**)

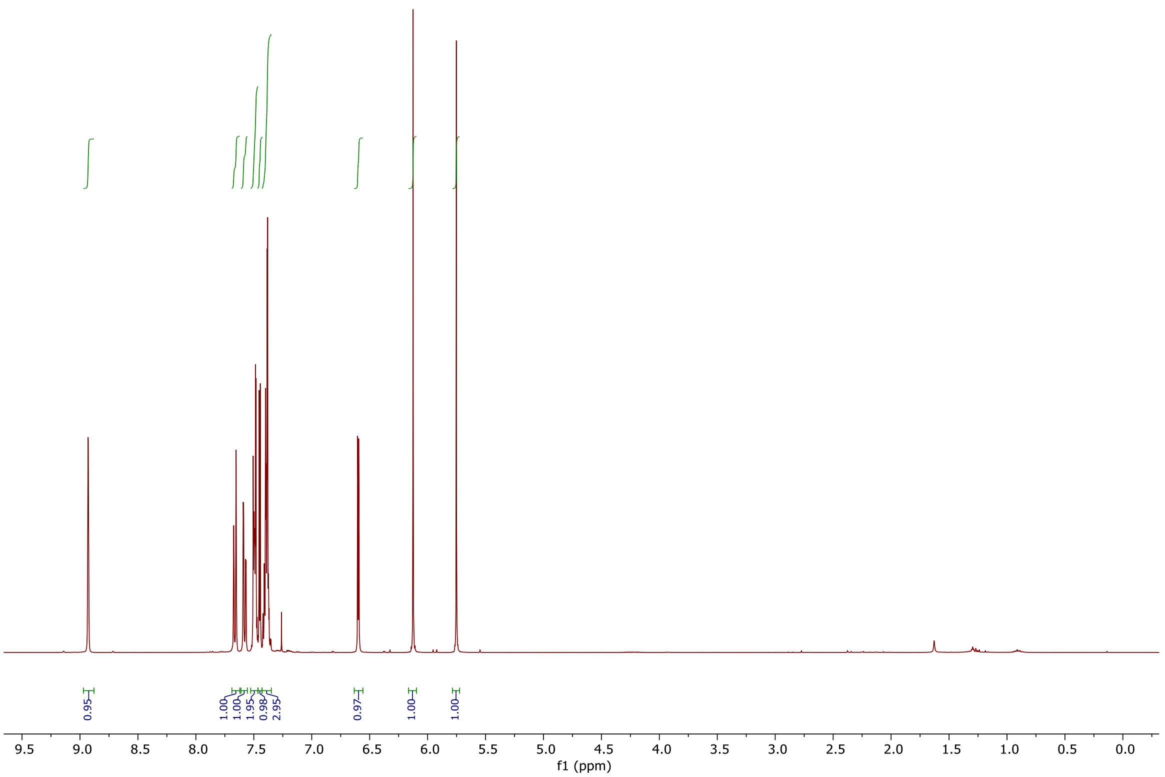

^{13}C-NMR: 2-phenyl-1-(6-(trifluoromethyl)-1H-indol-1-yl)prop-2-en-1-one (**S13**)

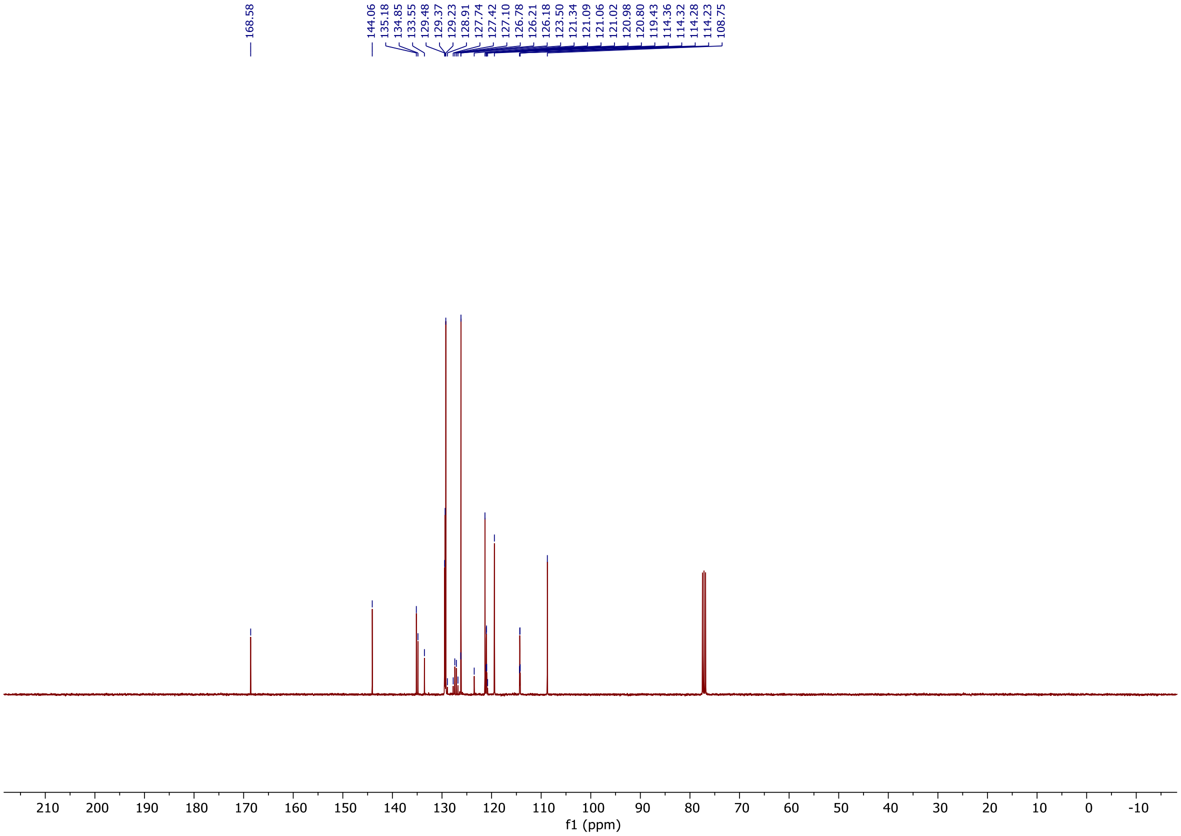

19H-NMR: 2-phenyl-1-(6-(trifluoromethyl)-1H-indol-1-yl)prop-2-en-1-one (**S13**)

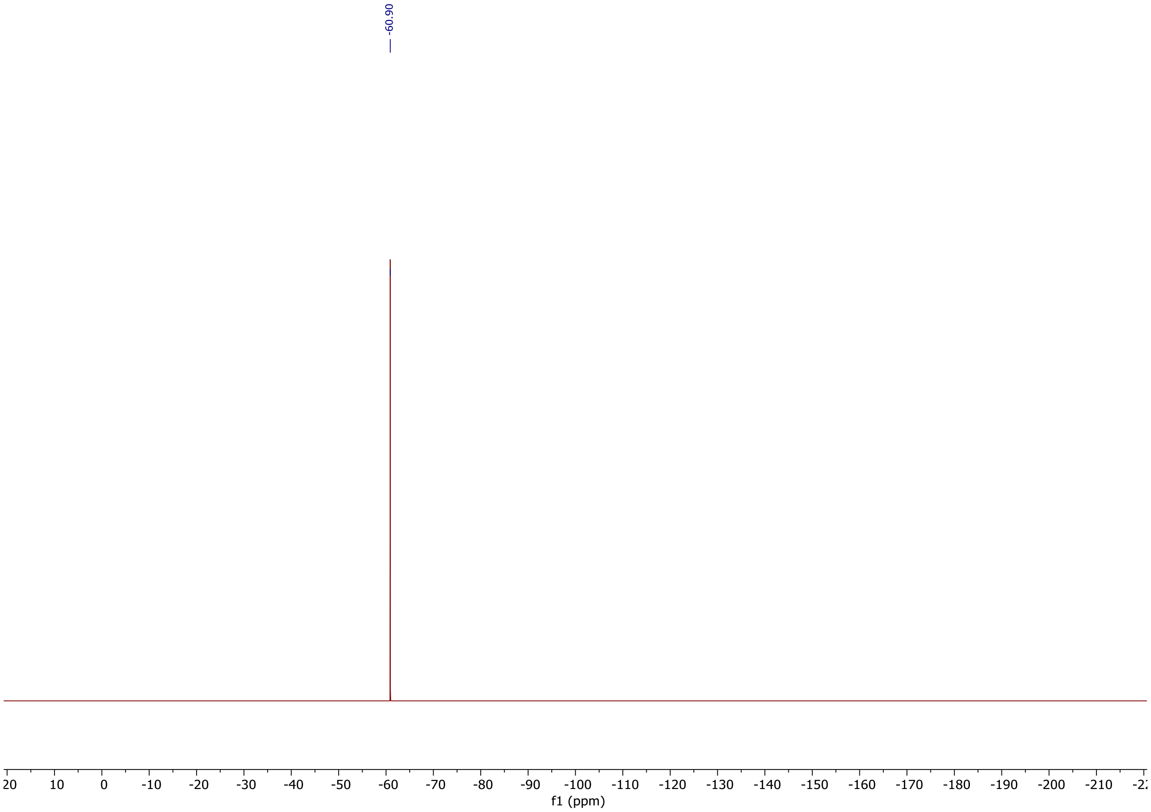

1H-NMR: 2-phenyl-1-(6-(trifluoromethyl)-1H-indol-1-yl)prop-2-en-1-one (**15**)

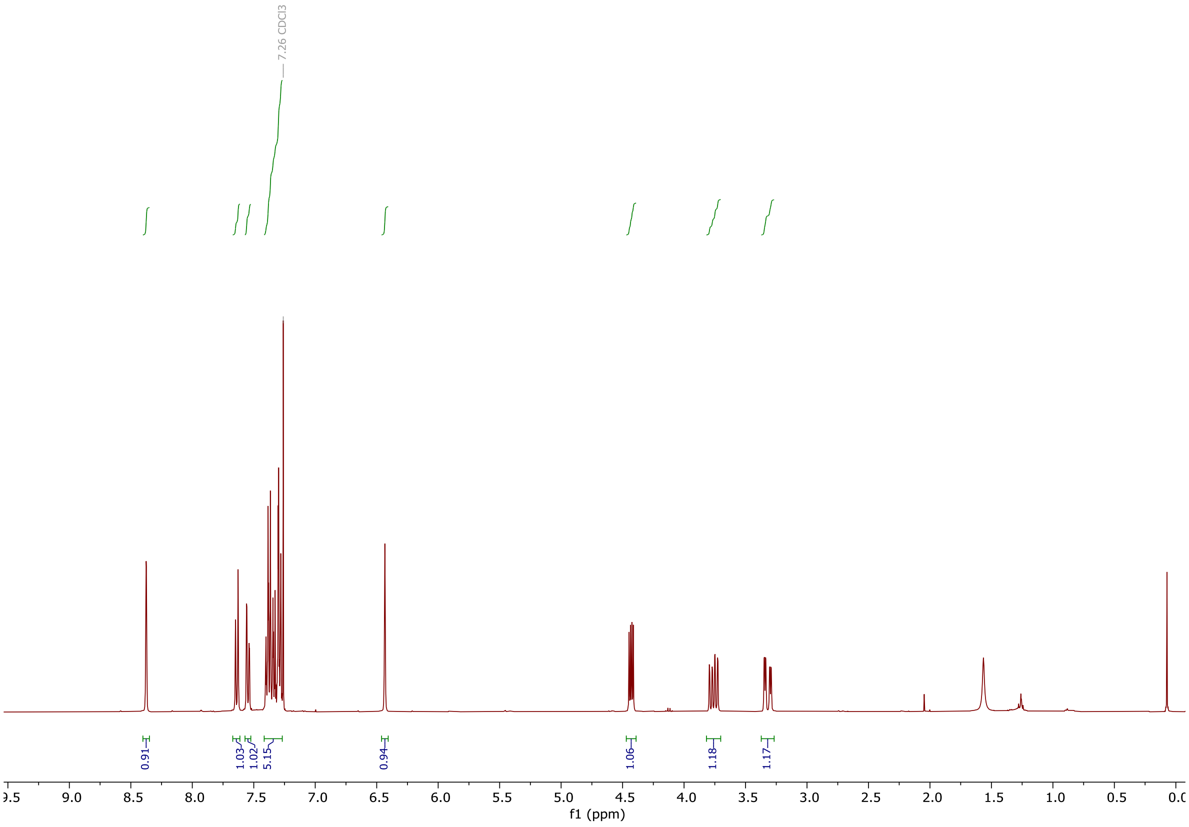

^{13}C-NMR: 2-phenyl-1-(6-(trifluoromethyl)-1H-indol-1-yl)prop-2-en-1-one (**15**)

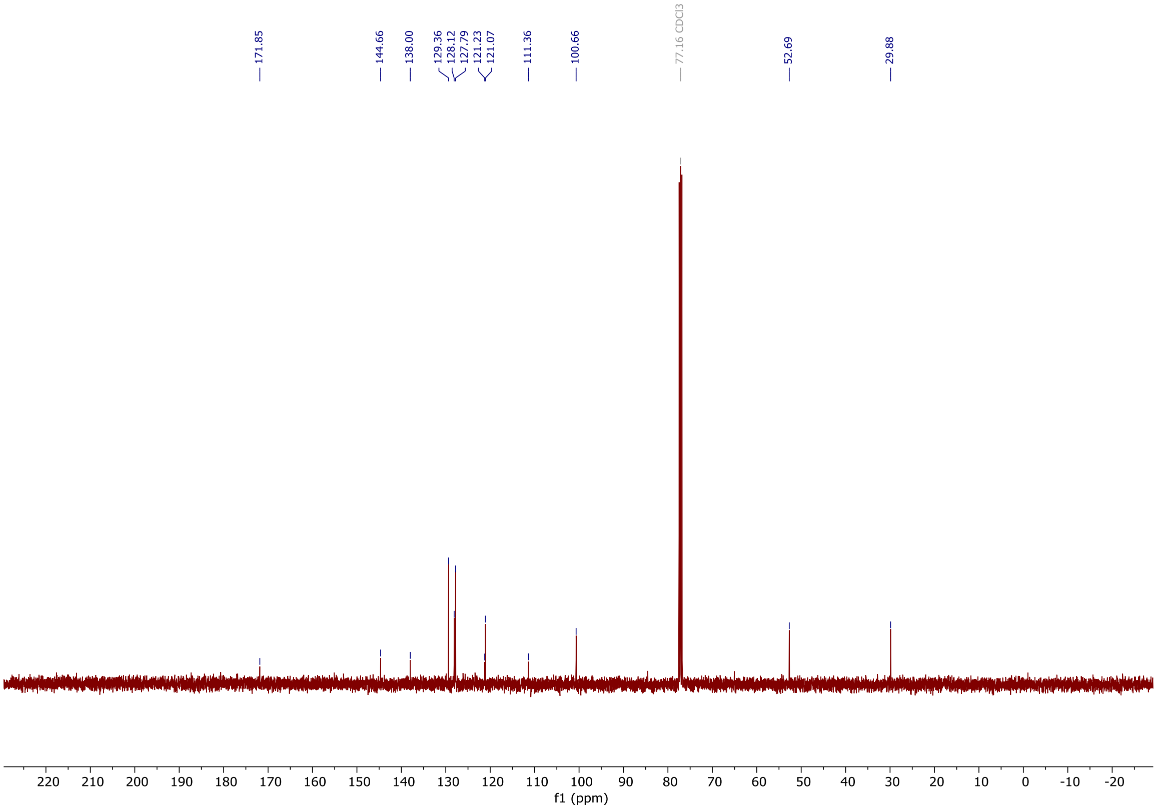

^{19}H-NMR: 2-phenyl-1-(6-(trifluoromethyl)-1H-indol-1-yl)prop-2-en-1-one (**15**)

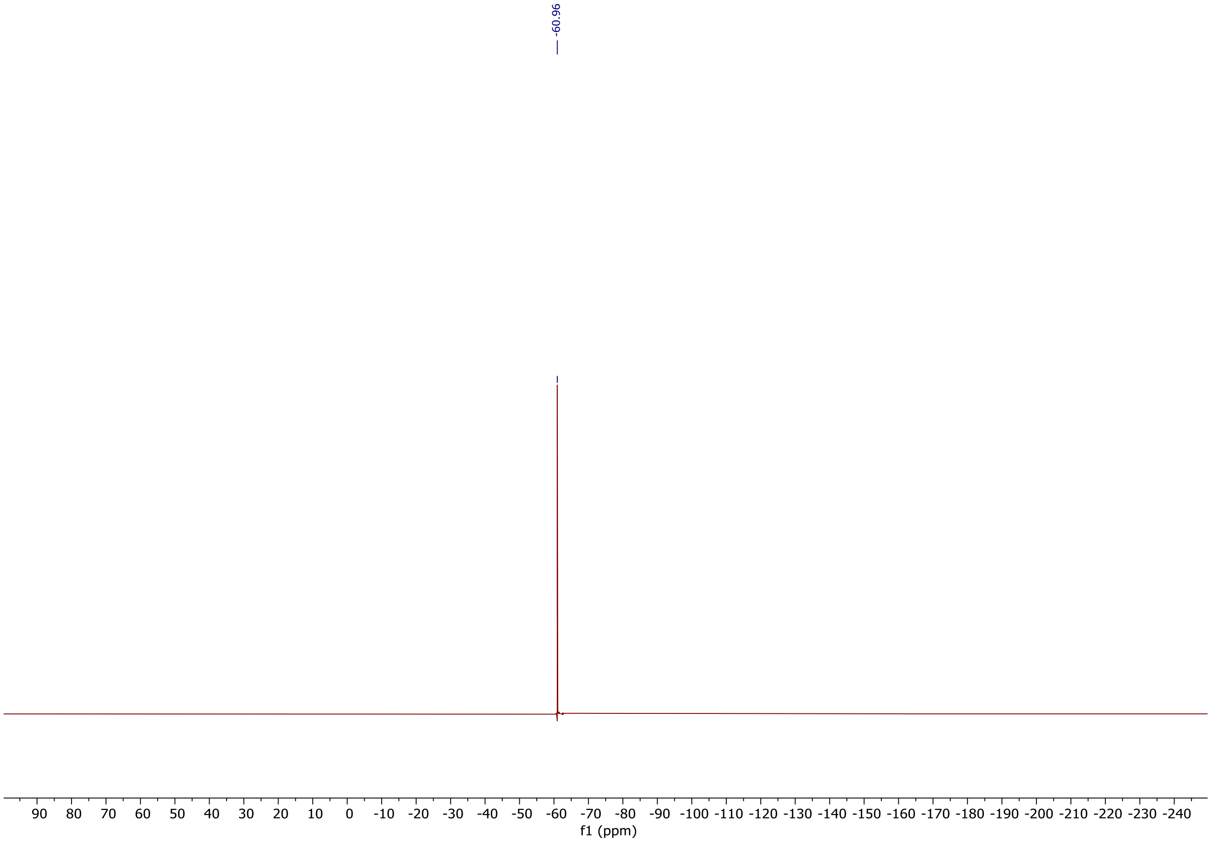

^{1}H-NMR: 1-(2-phenylacryloyl)-1H-indole-6-carbaldehyde (**S14**)

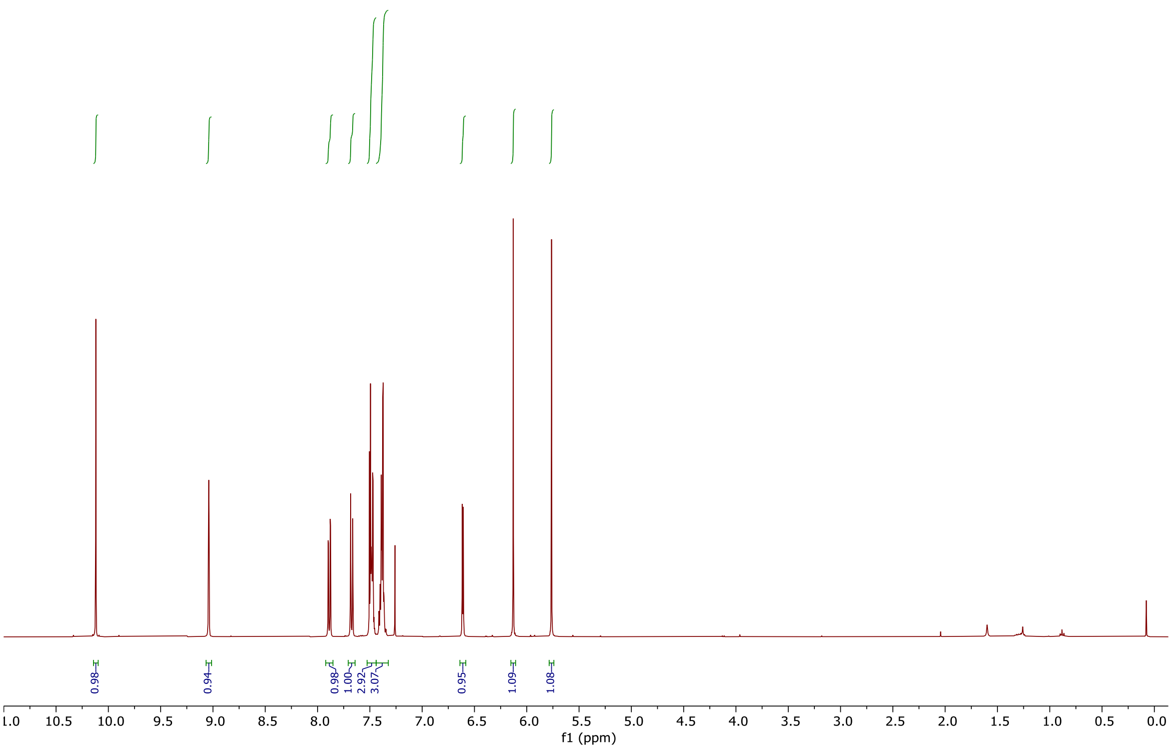

^{13}C-NMR: 1-(2-phenylacryloyl)-1H-indole-6-carbaldehyde (**S14**)

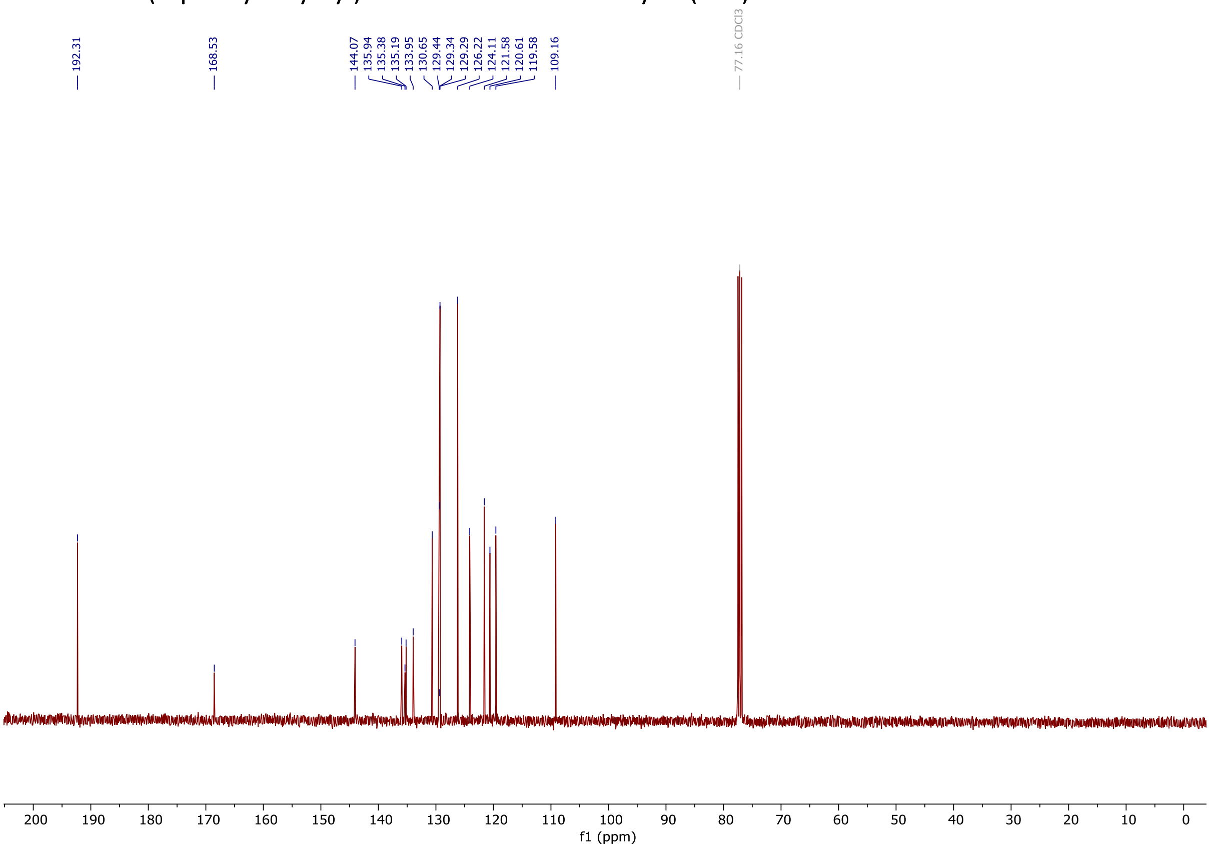

^{1}H-NMR: 3-oxo-2-phenyl-2,3-dihydro-1H-pyrrolo[1,2-a]indole-6-carbaldehyde (**16**)

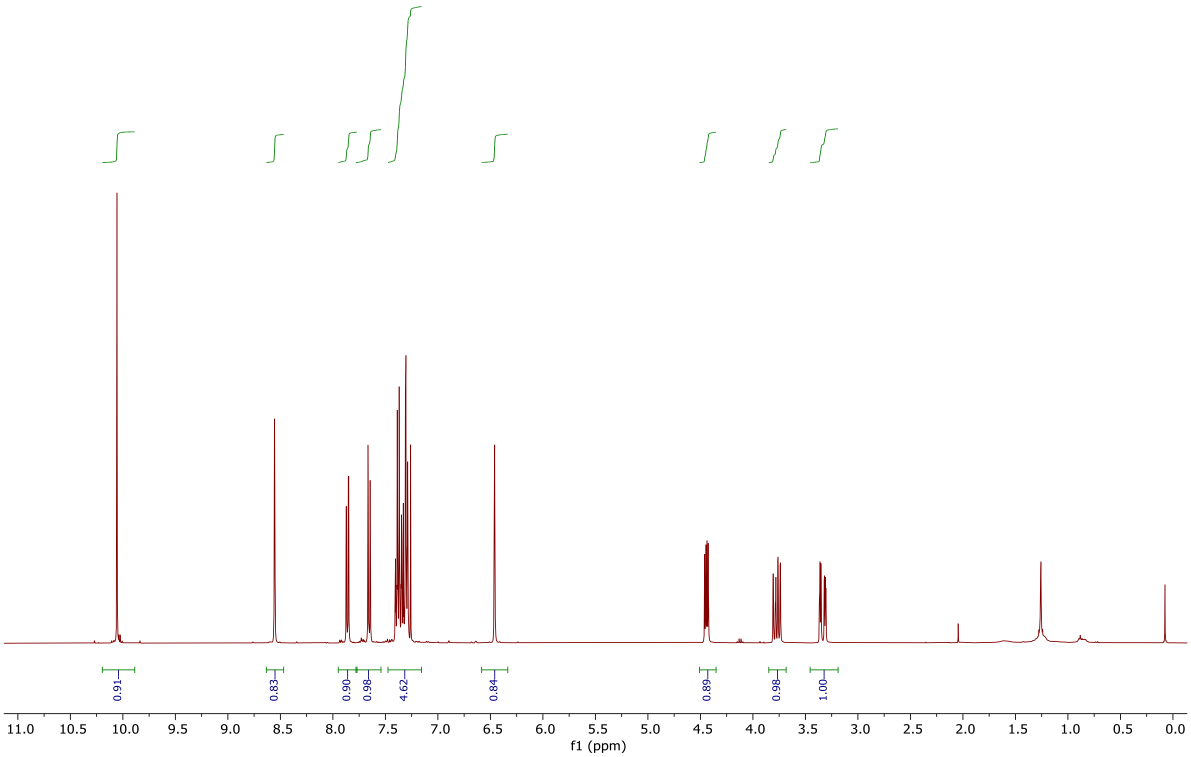

^{13}C-NMR: 3-oxo-2-phenyl-2,3-dihydro-1H-pyrrolo[1,2-a]indole-6-carbaldehyde (**16**)

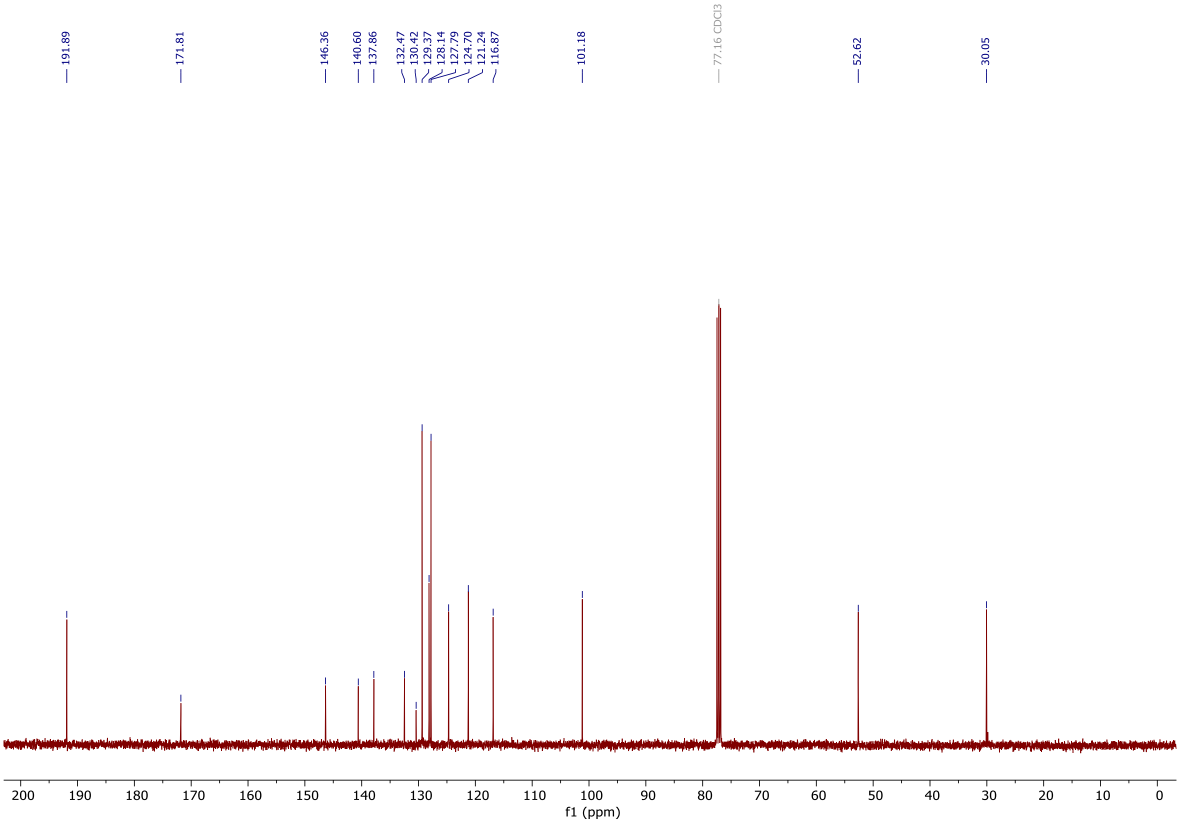

1H-NMR: 1-(7-fluoro-1H-indol-1-yl)-2-phenylprop-2-en-1-one (**S15**)

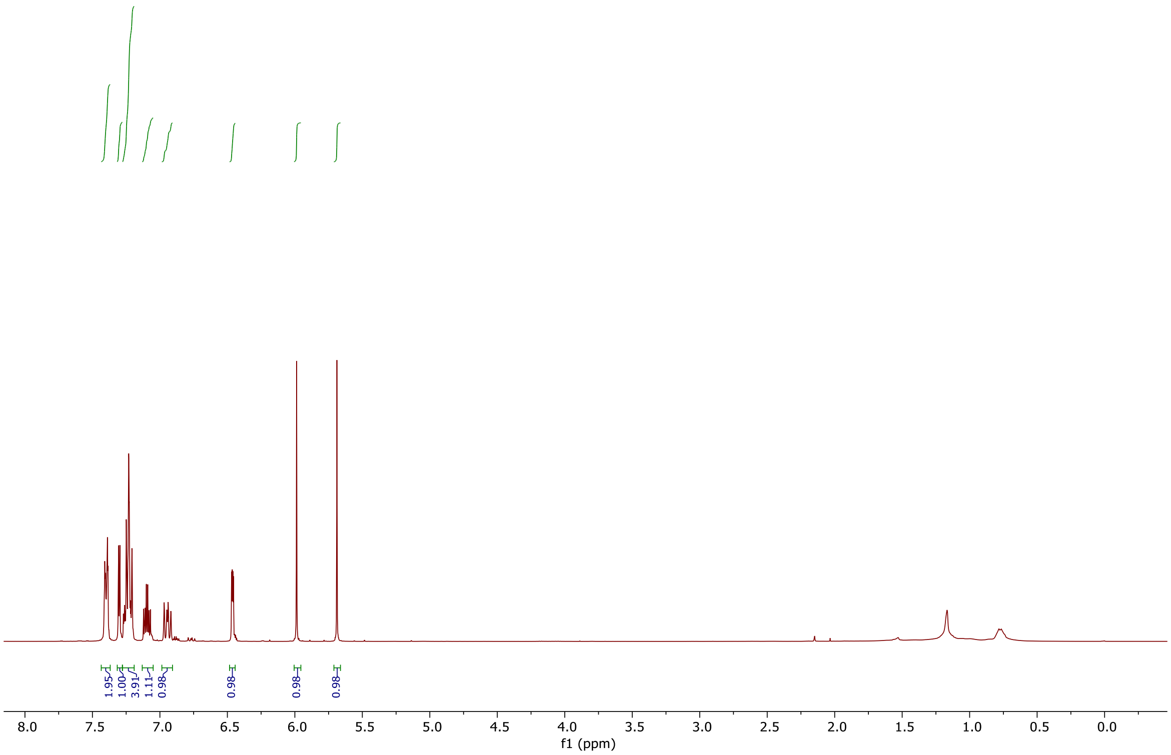

13C-NMR: 1-(7-fluoro-1H-indol-1-yl)-2-phenylprop-2-en-1-one (**S15**)

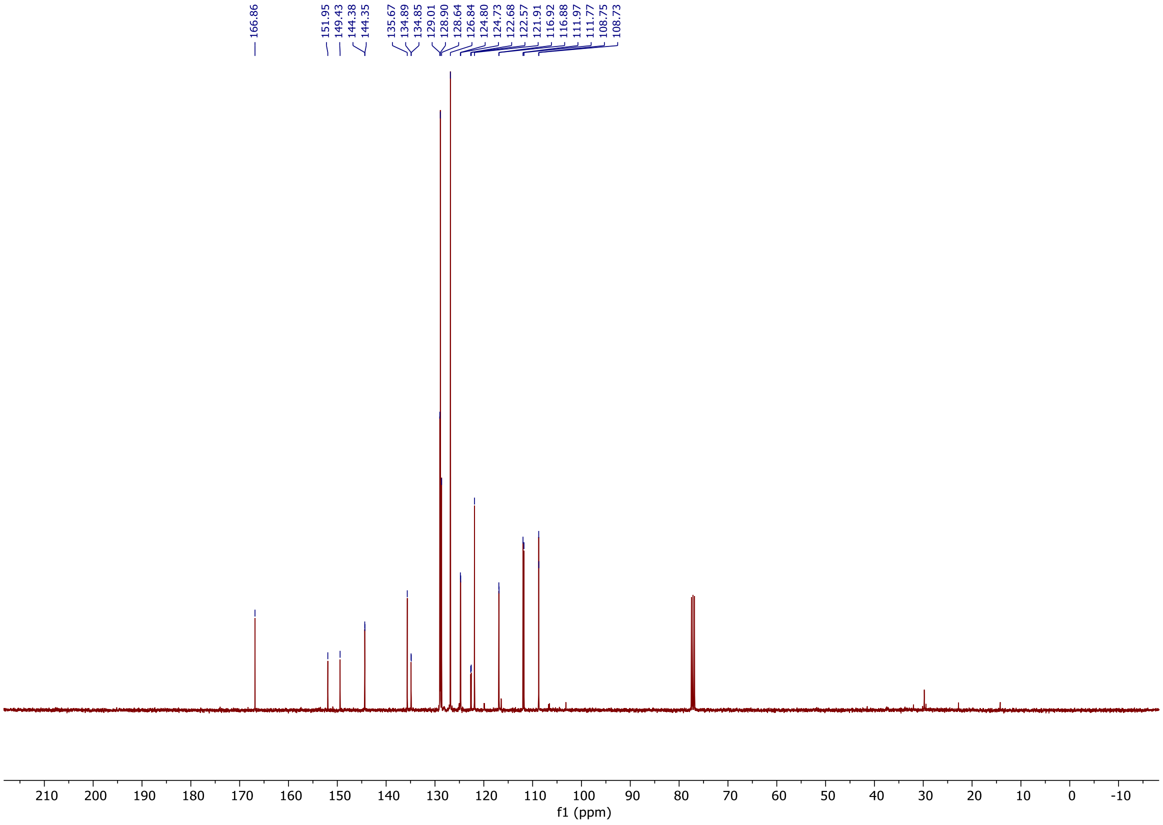

^{19}F-NMR: 1-(7-fluoro-1H-indol-1-yl)-2-phenylprop-2-en-1-one (**S15**)

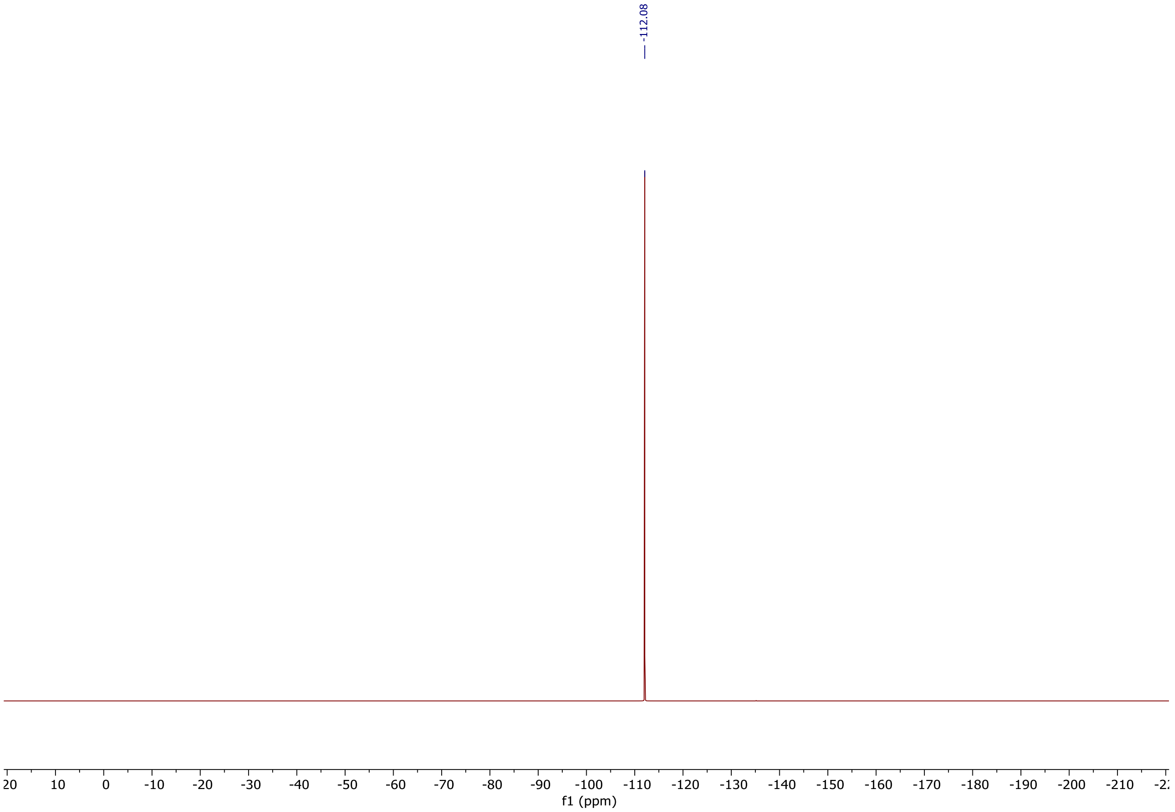

^{1}H-NMR: 5-fluoro-2-phenyl-1,2-dihydro-3H-pyrrolo[1,2-a]indol-3-one (**17**)

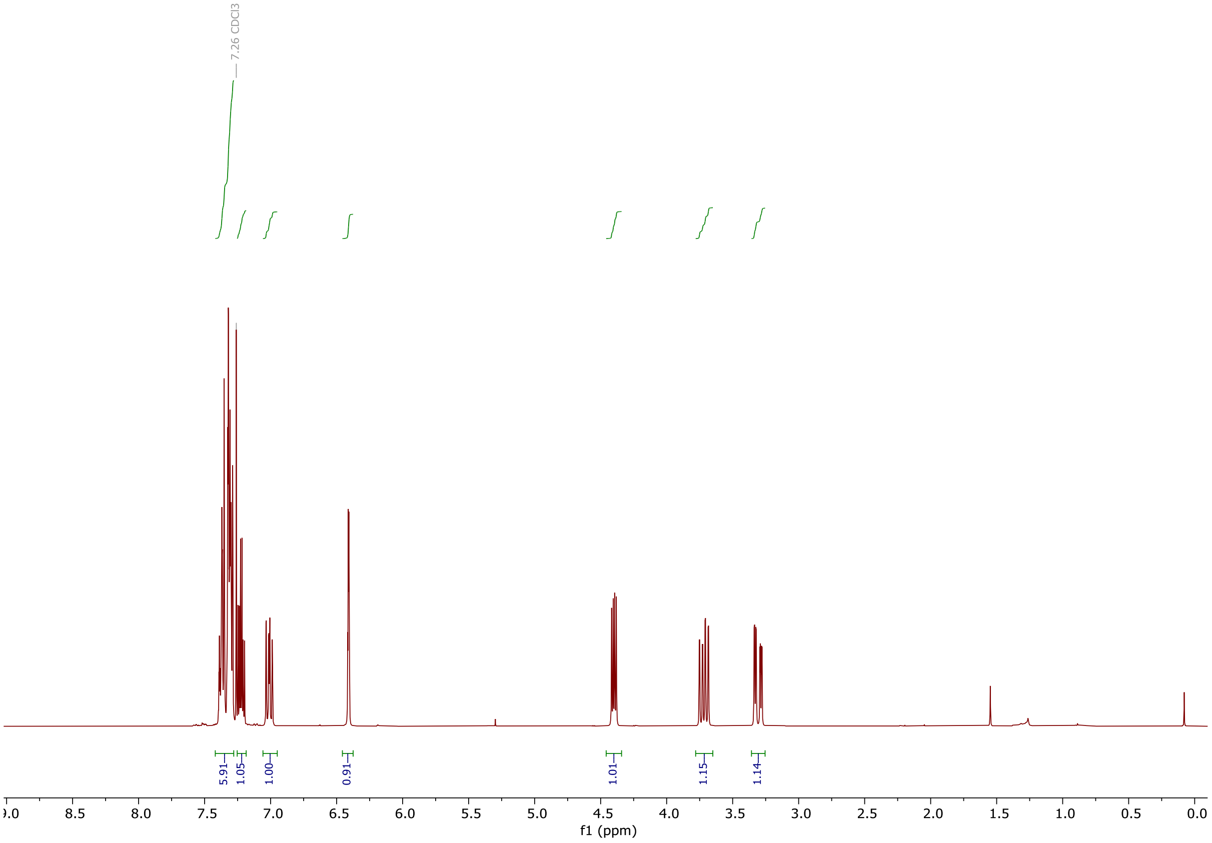

^{13}C-NMR: 5-fluoro-2-phenyl-1,2-dihydro-3H-pyrrolo[1,2-a]indol-3-one (**17**)

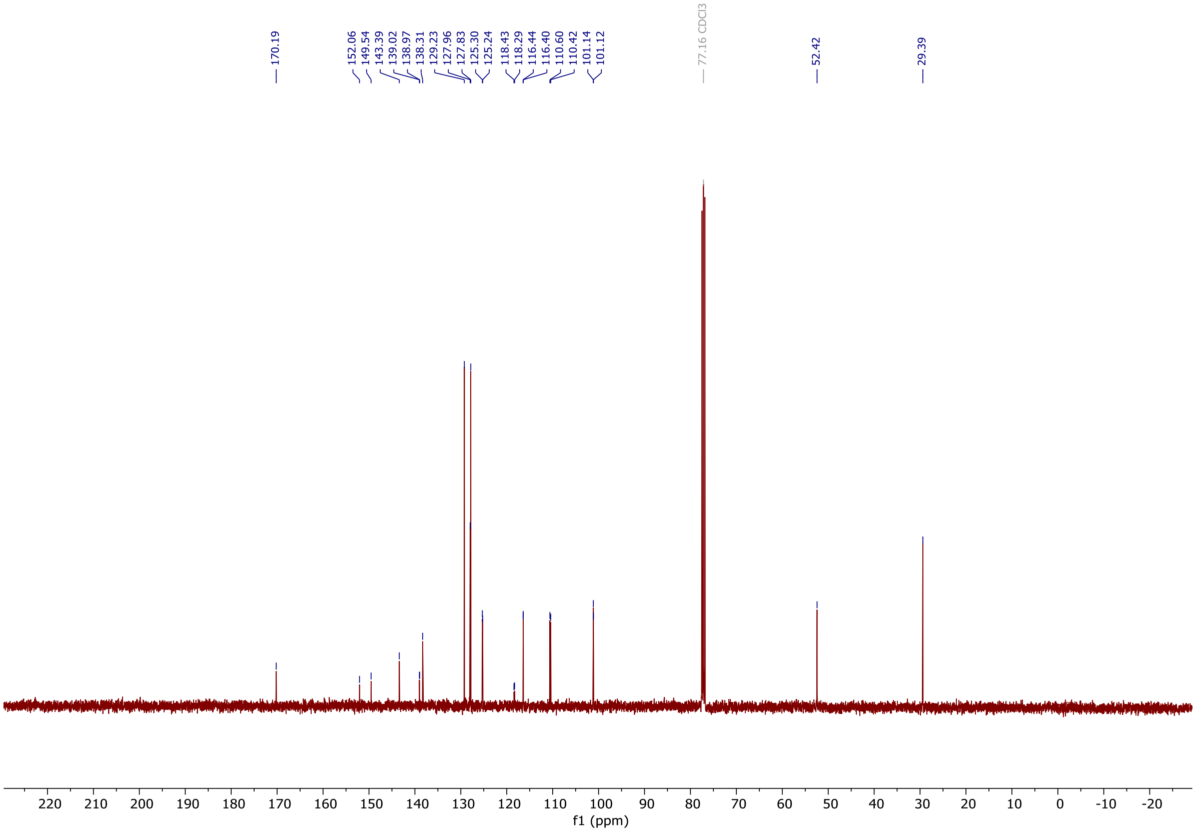

^{19}F-NMR: 5-fluoro-2-phenyl-1,2-dihydro-3H-pyrrolo[1,2-a]indol-3-one (**17**)

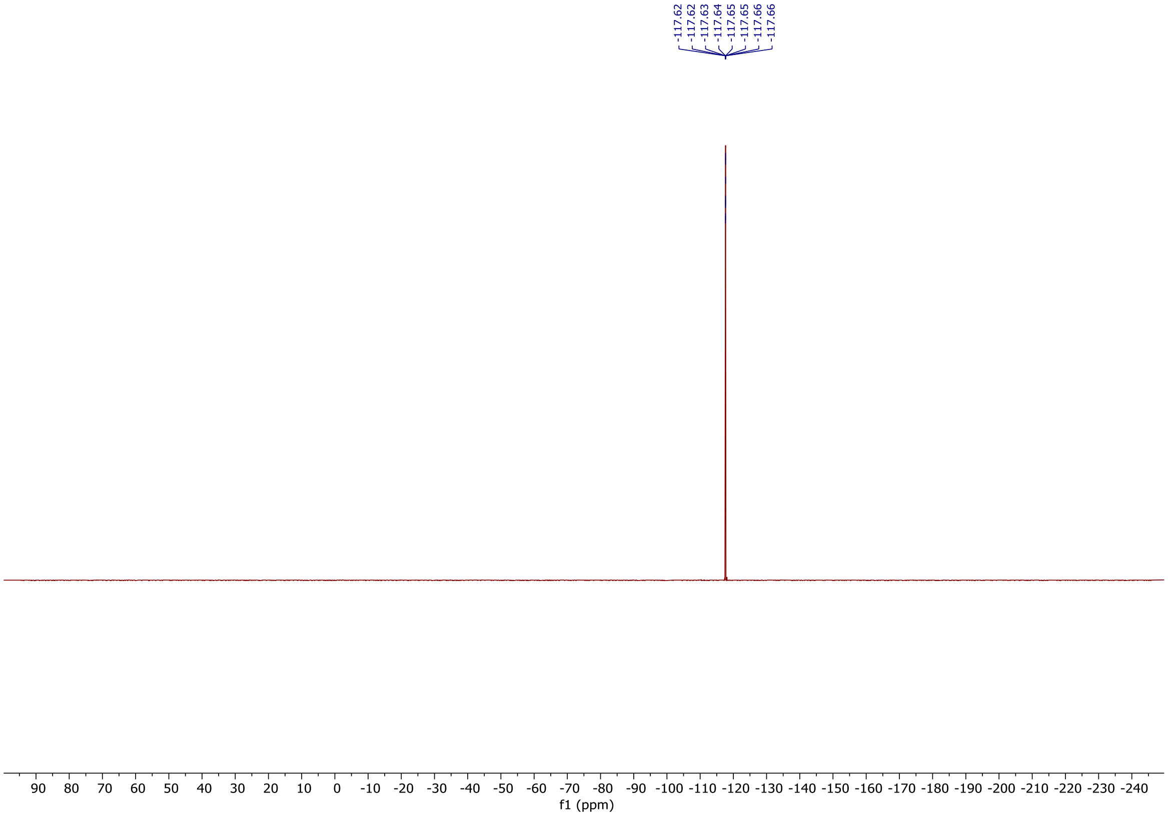

^{1}H-NMR: 2-phenyl-1-(1H-pyrrolo[2,3-c]pyridin-1-yl)prop-2-en-1-one (**S16**)

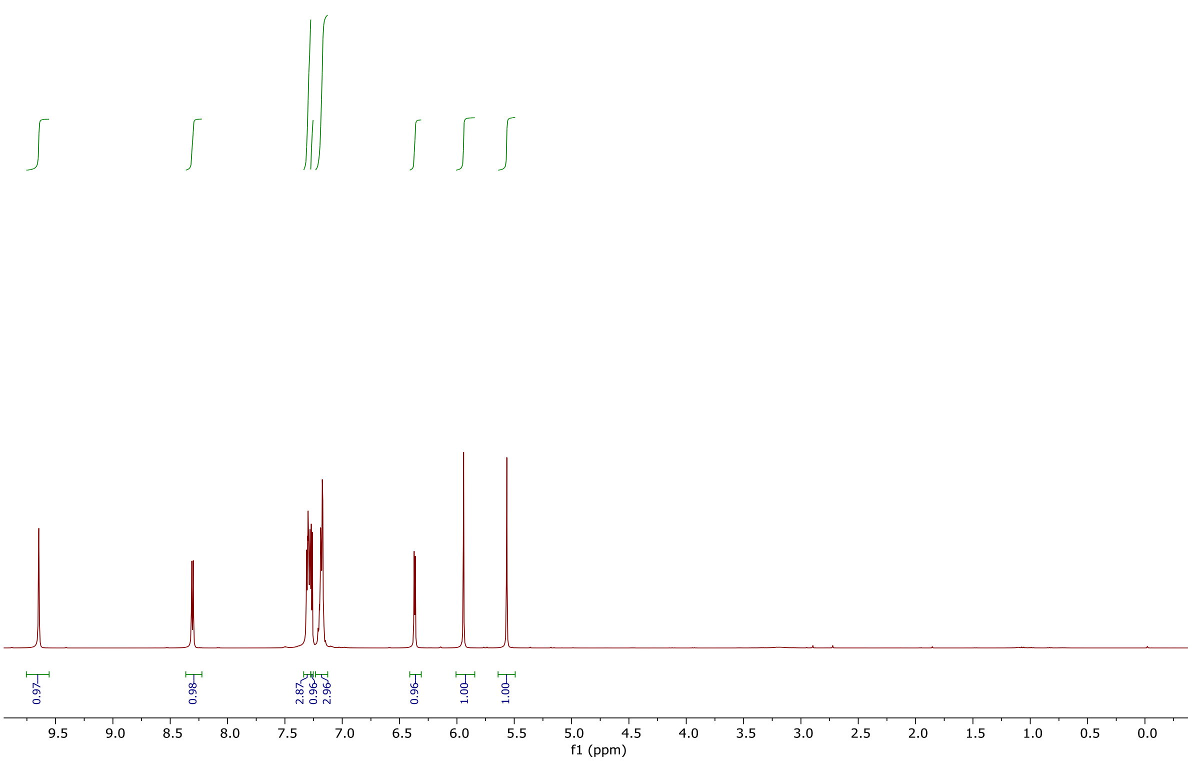

^{13}C-NMR: 2-phenyl-1-(1H-pyrrolo[2,3-c]pyridin-1-yl)prop-2-en-1-one (**S16**)

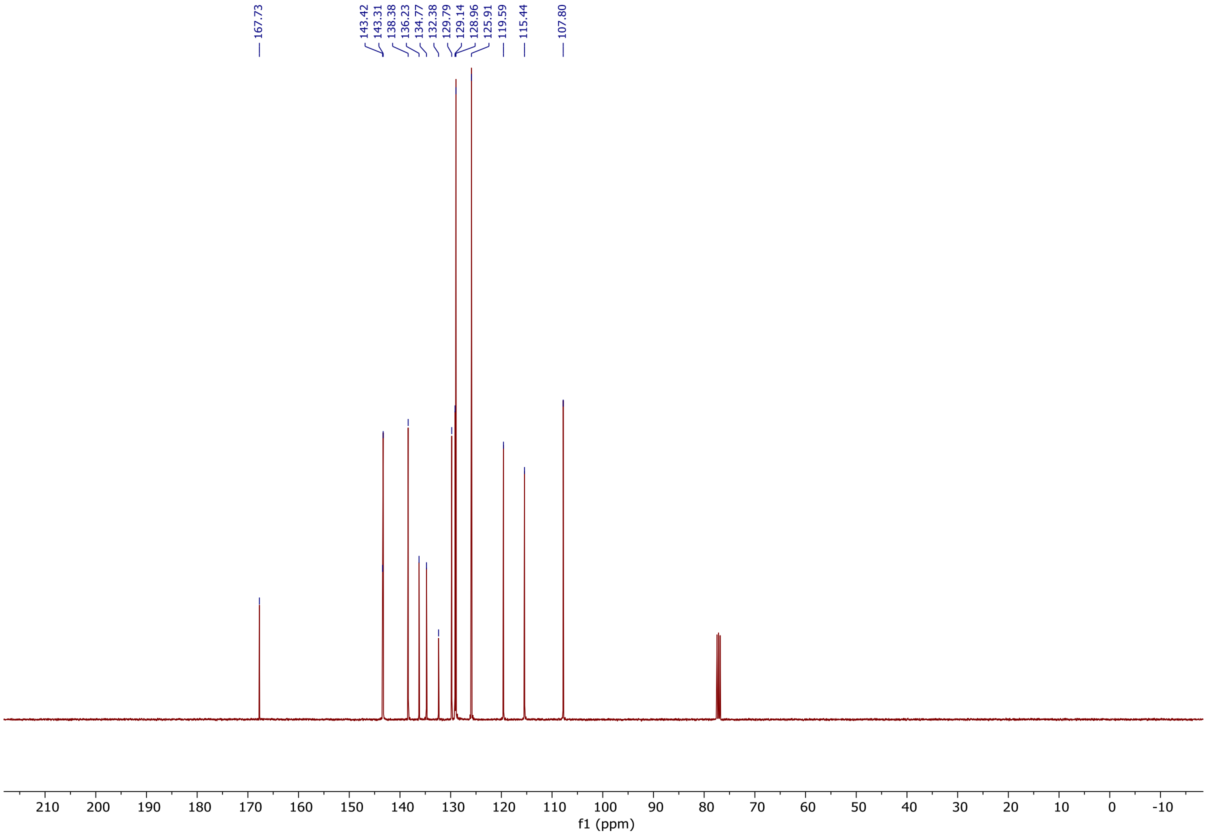

^{1}H-NMR: 7-phenyl-6,7-dihydro-8H-pyrido[4,3-b]pyrrolizin-8-one (**18**)

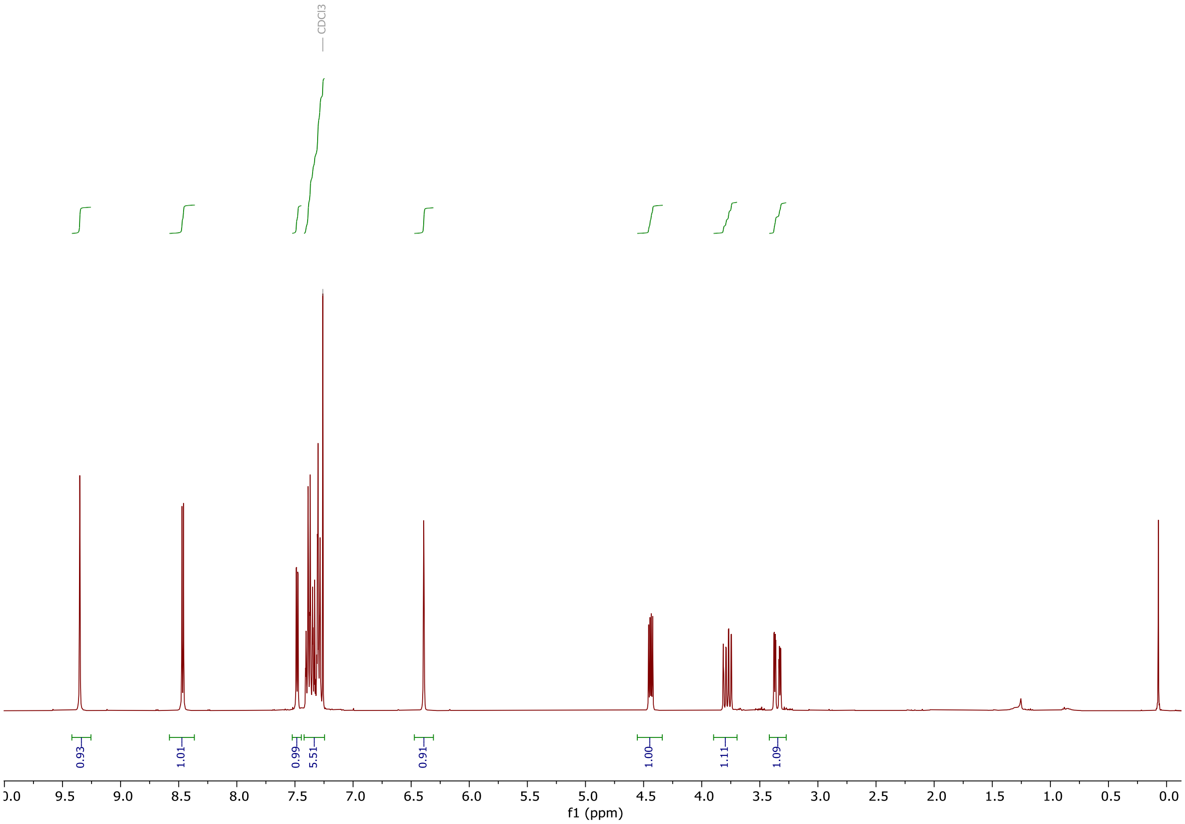

^{13}C-NMR: 7-phenyl-6,7-dihydro-8H-pyrido[4,3-b]pyrrolizin-8-one (**18**)

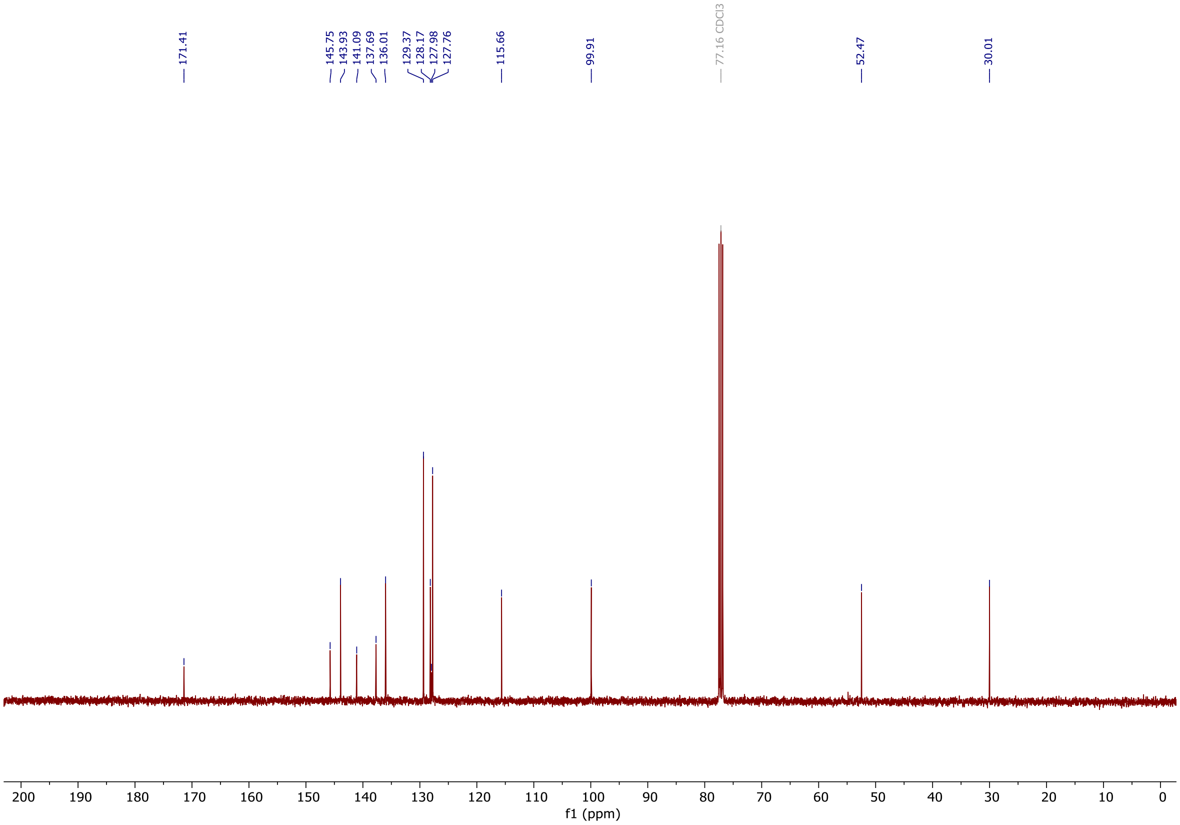

^{1}H-NMR: 2-phenyl-1-(1H-pyrrolo[3,2-c]pyridin-1-yl)prop-2-en-1-one (**S17**)

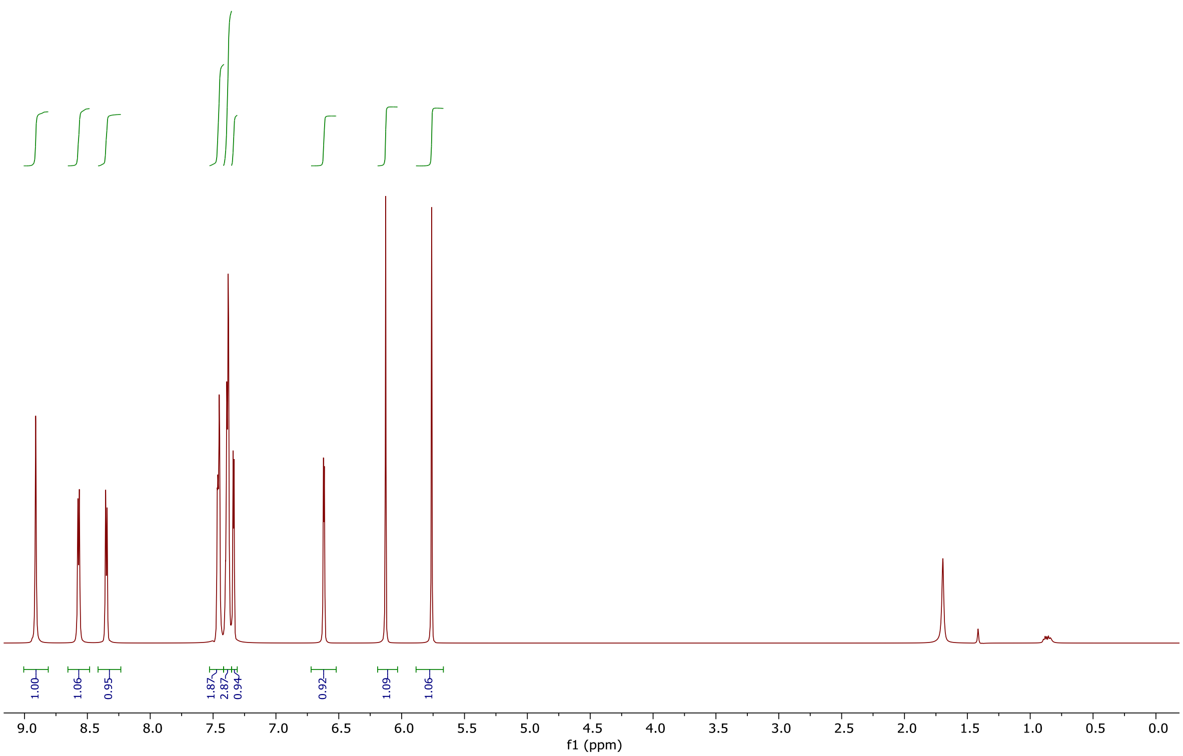

^{13}C-NMR: 2-phenyl-1-(1H-pyrrolo[3,2-c]pyridin-1-yl)prop-2-en-1-one (**S17**)

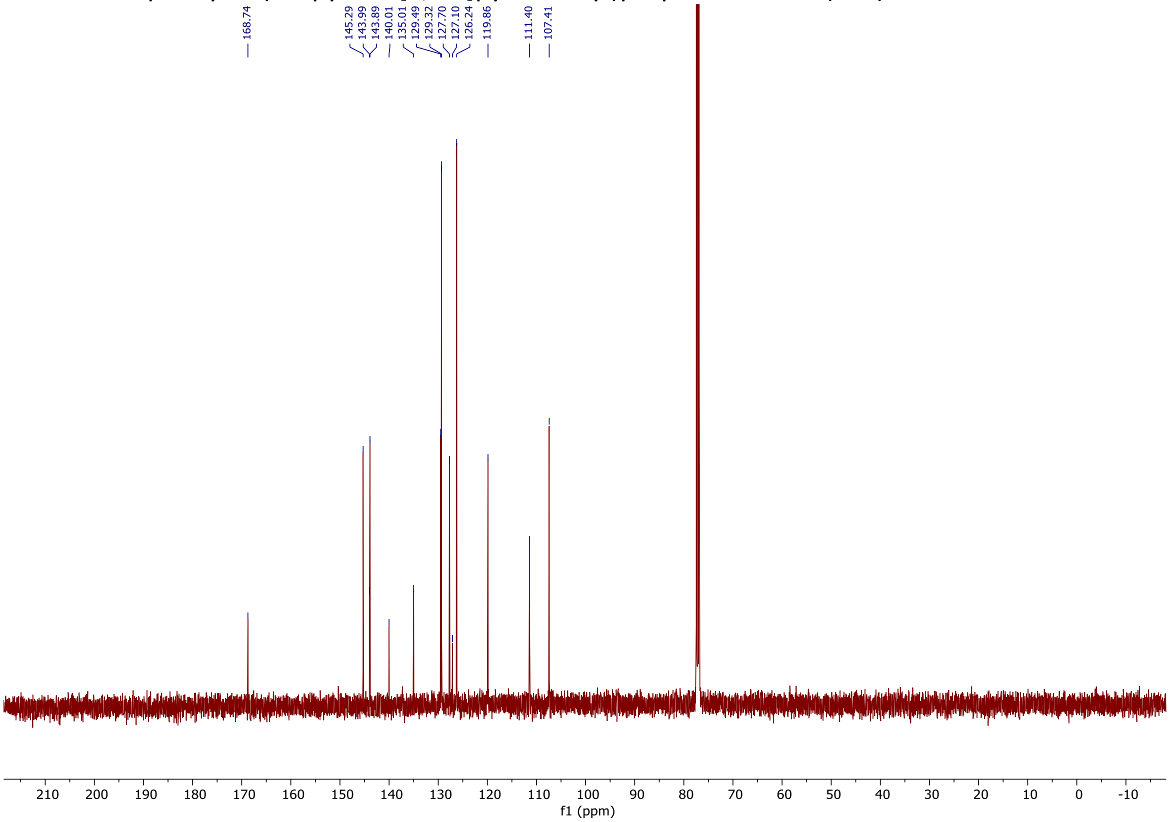

^{1}H-NMR: 7-phenyl-7,8-dihydro-6H-pyrido[3,4-b]pyrrolizin-6-one (**19**)

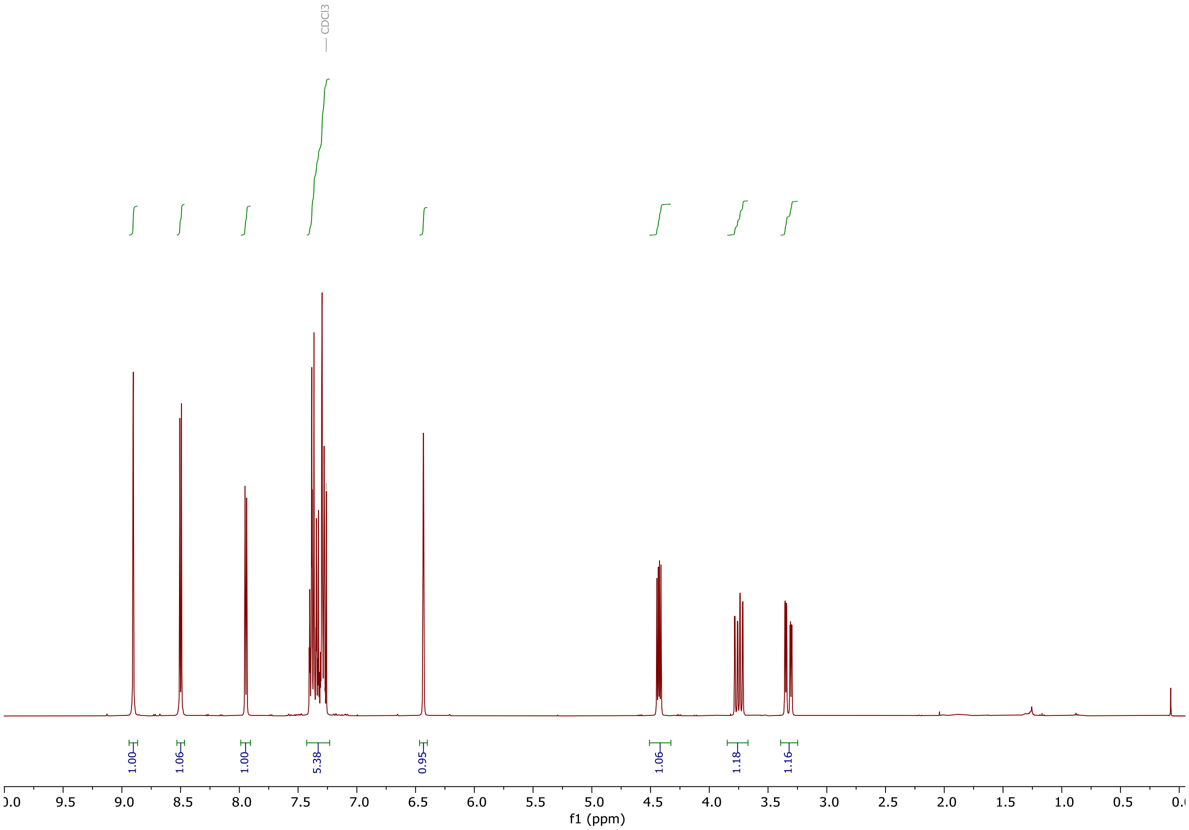

^{13}C-NMR: 7-phenyl-7,8-dihydro-6H-pyrido[3,4-b]pyrrolizin-6-one (**19**)

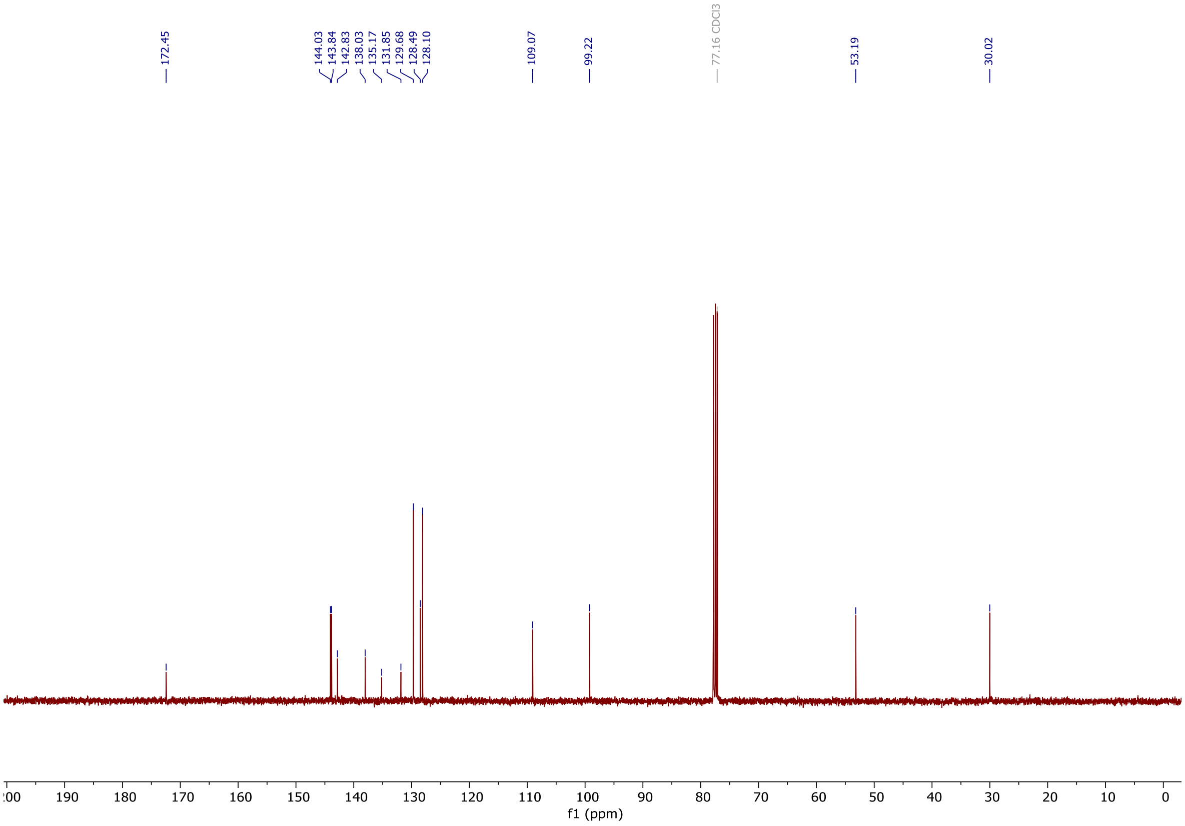

^{1}H-NMR: 2-phenyl-1-(1H-pyrrolo[3,2-b]pyridin-1-yl)prop-2-en-1-one (**S18**)

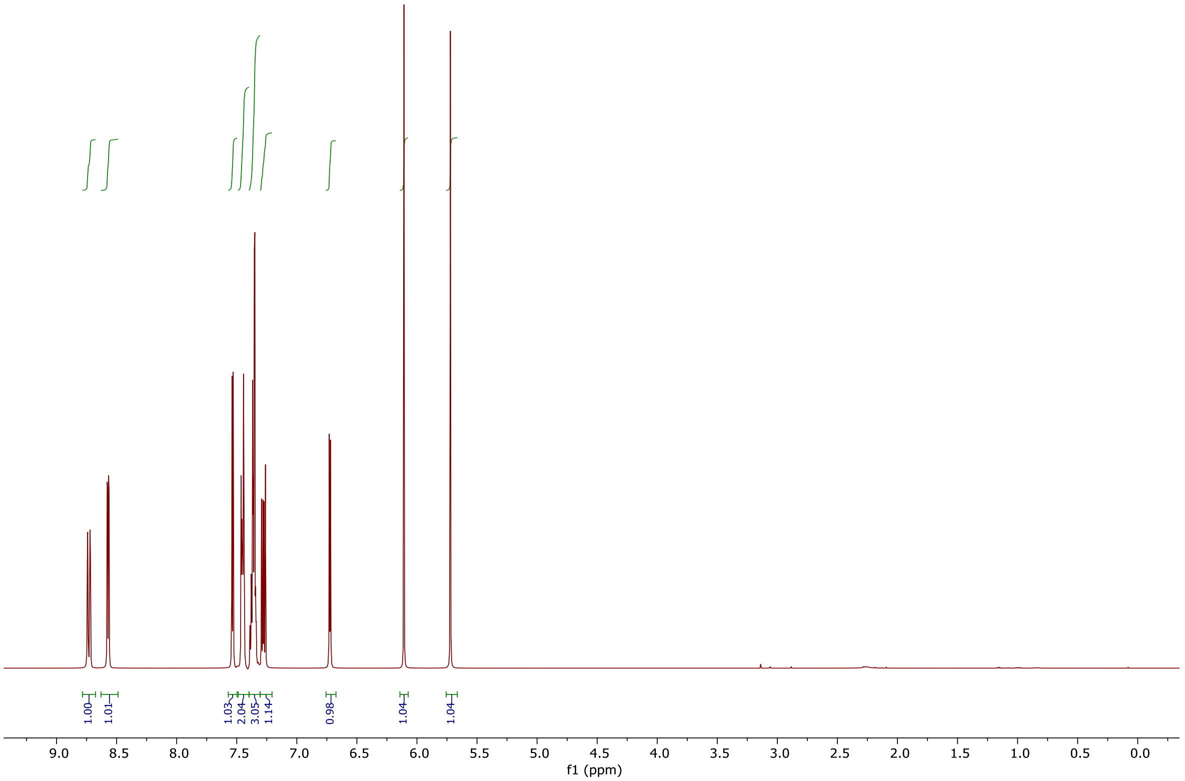

^{13}C-NMR: 2-phenyl-1-(1H-pyrrolo[3,2-b]pyridin-1-yl)prop-2-en-1-one (**S18**)

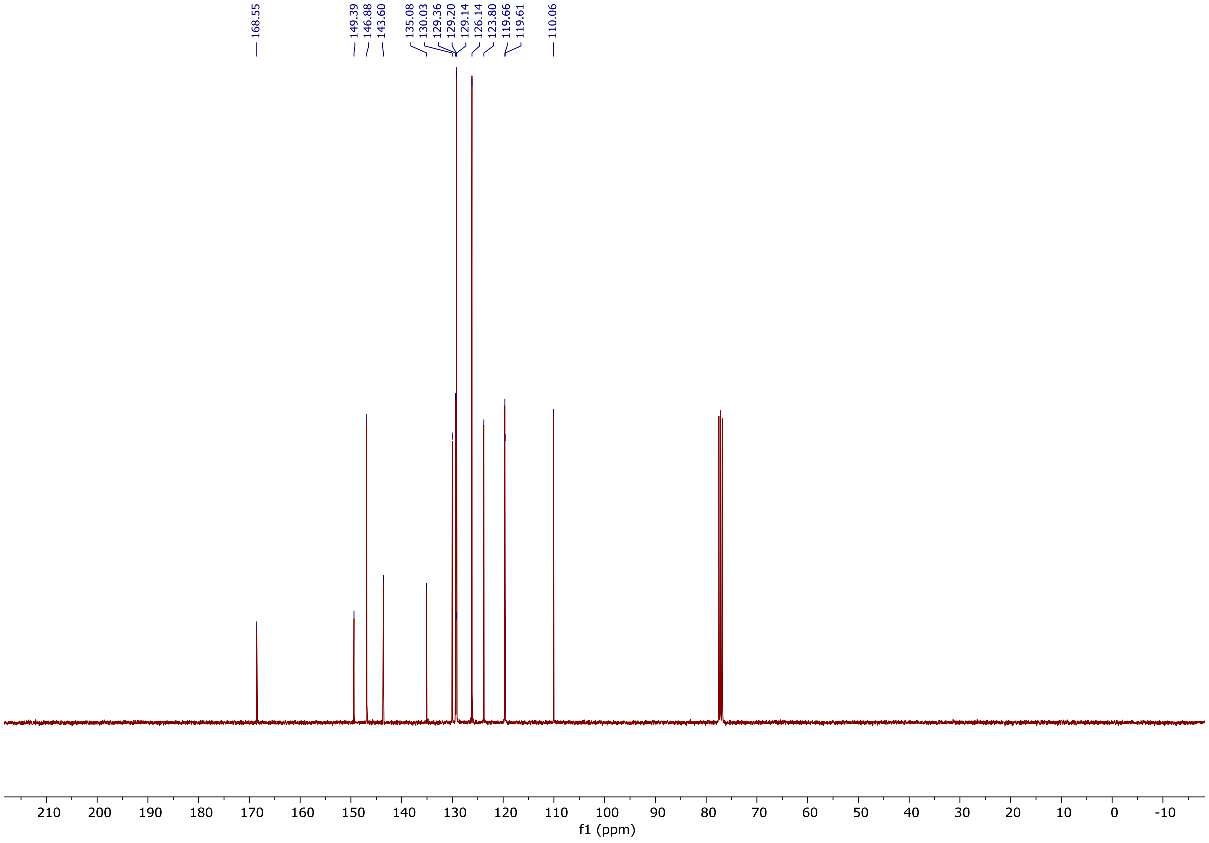

^{1}H-NMR: 7-phenyl-7,8-dihydro-6H-pyrido[2,3-b]pyrrolizin-6-one (**20**)

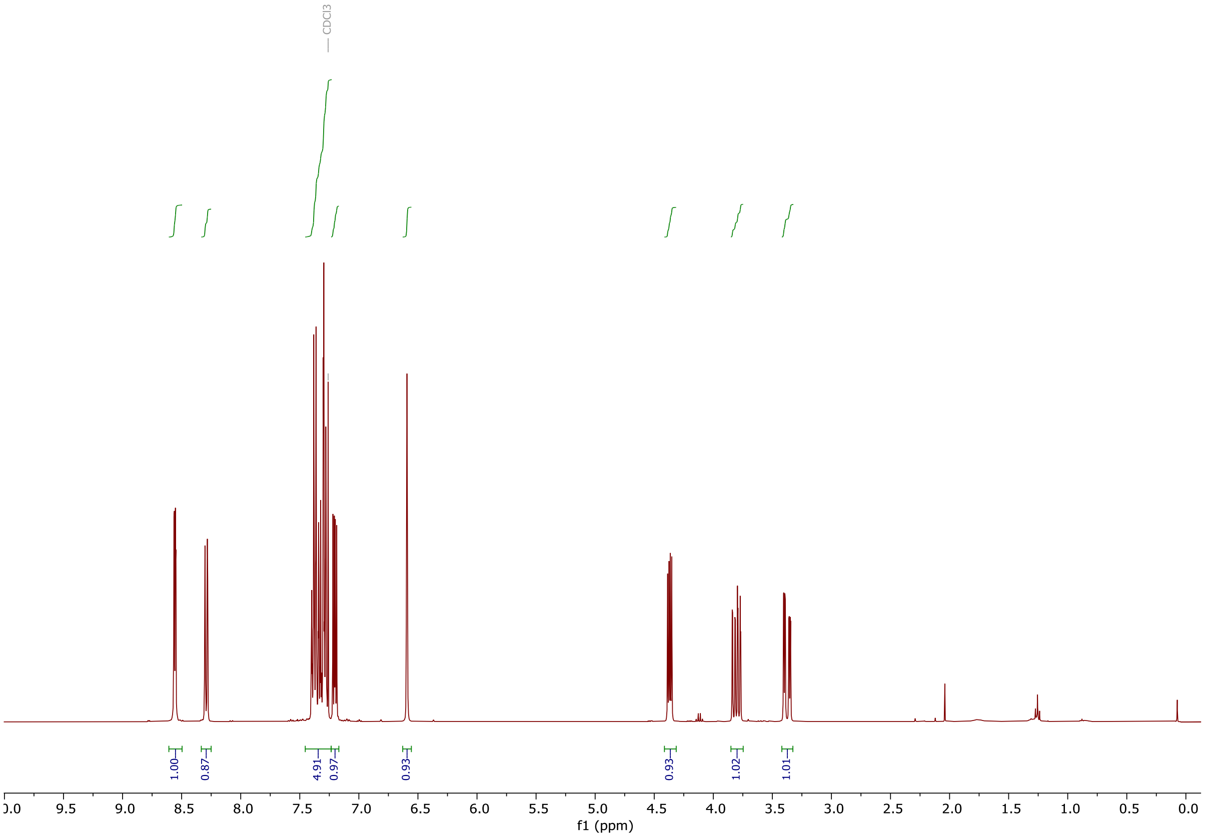

^{13}C-NMR: 7-phenyl-7,8-dihydro-6H-pyrido[2,3-b]pyrrolizin-6-one (**20**)

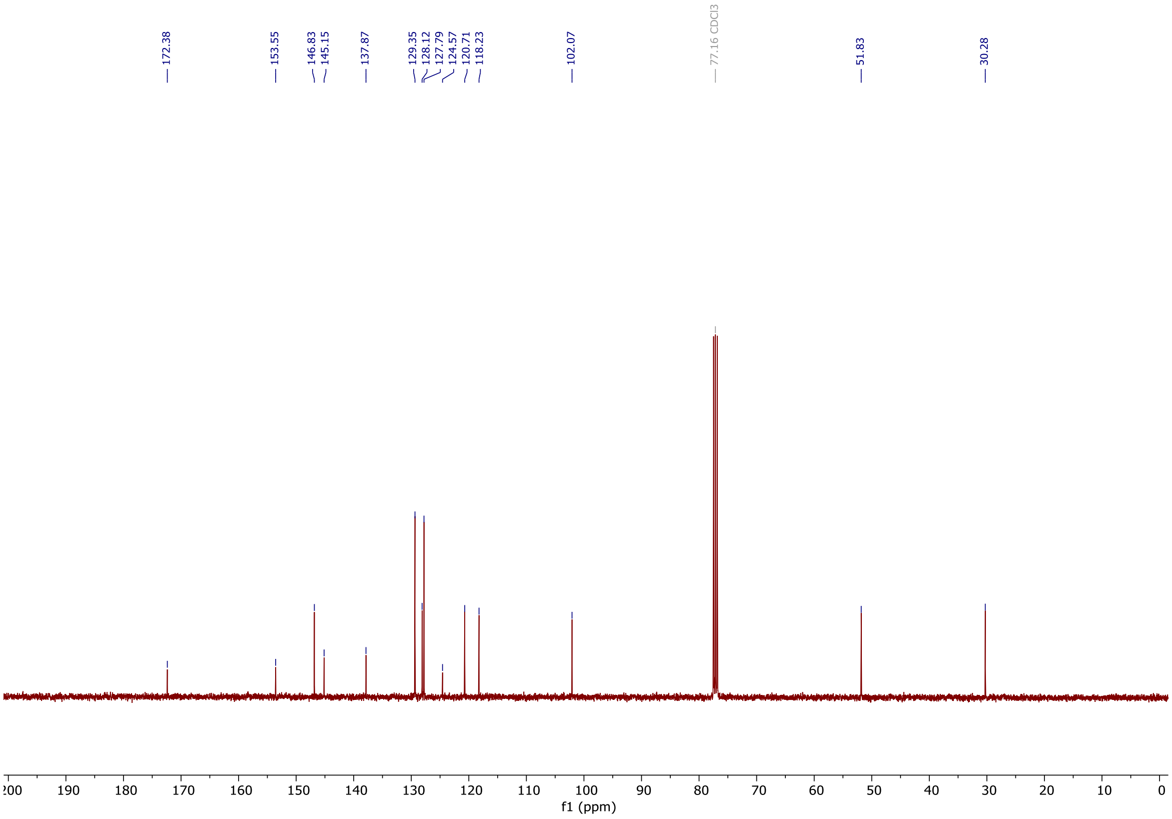

^{1}H-NMR: 2-phenylacrylamide (**S19**)

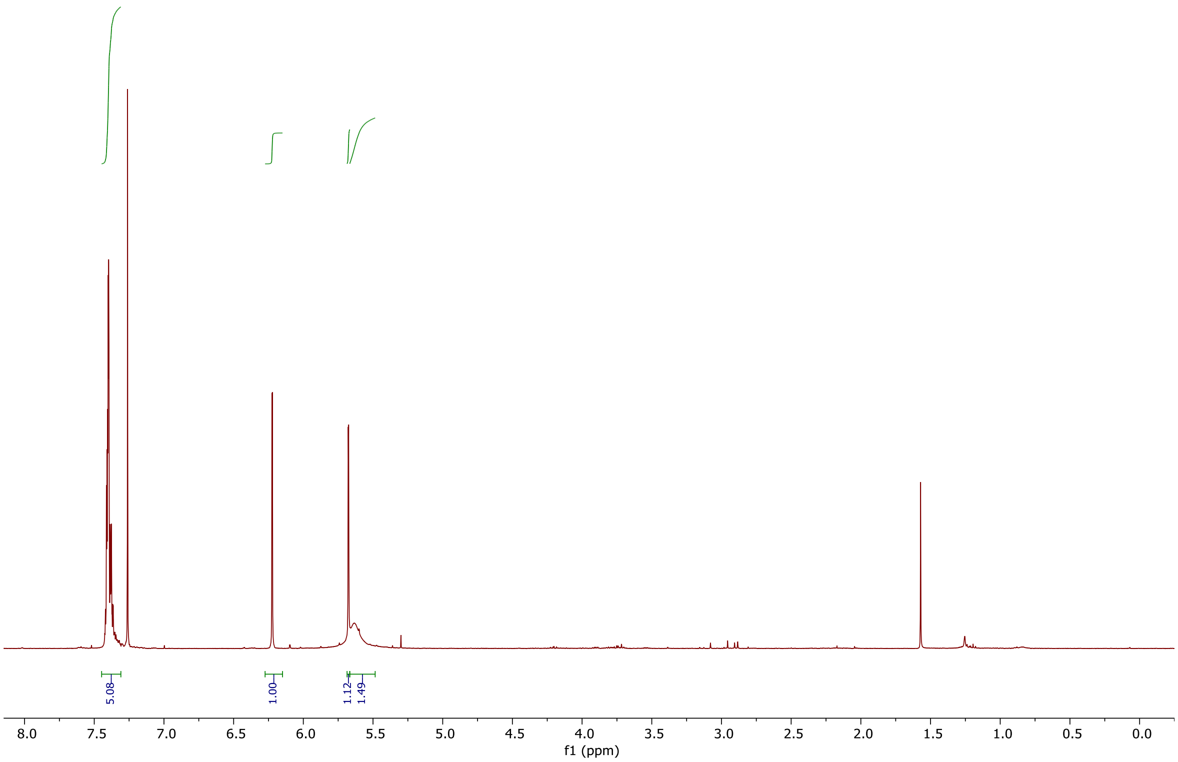

^{13}C-NMR: 2-phenylacrylamide (**S19**)

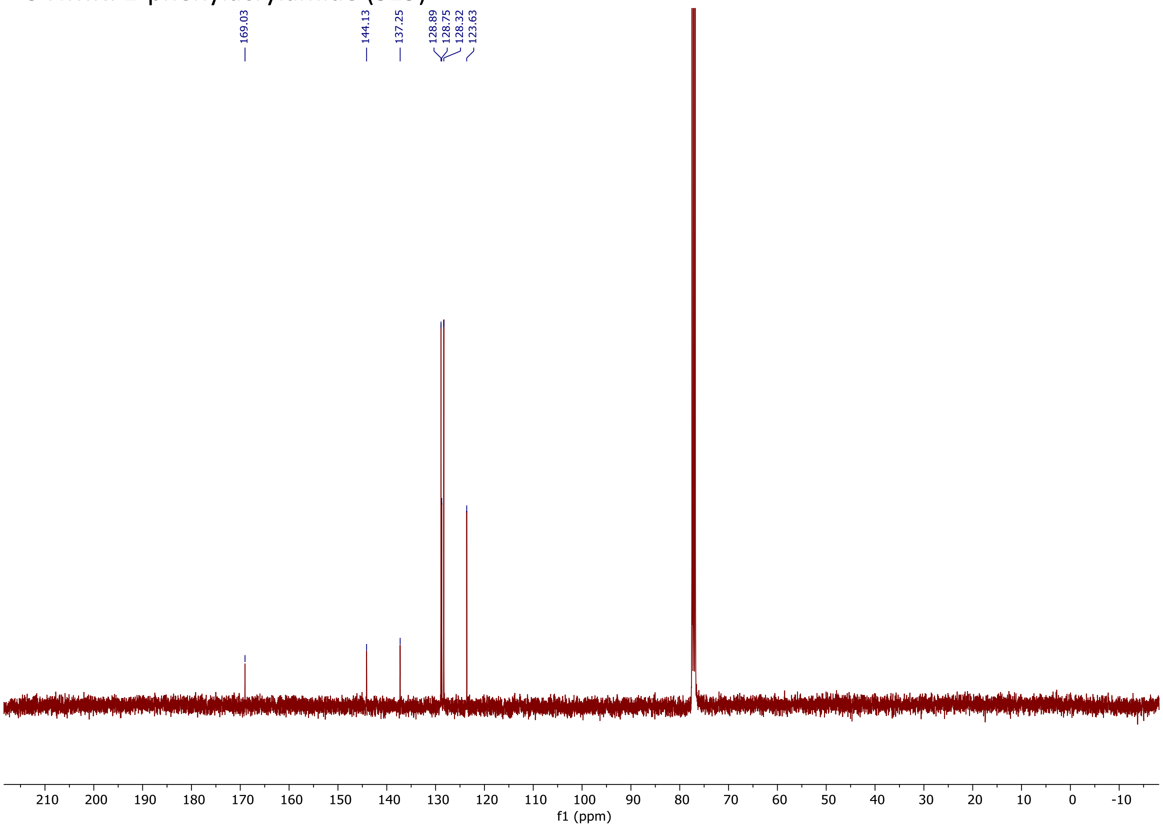

^{1}H-NMR: 2-phenyl-1-(1H-pyrrol-1-yl)prop-2-en-1-one (**S20**)

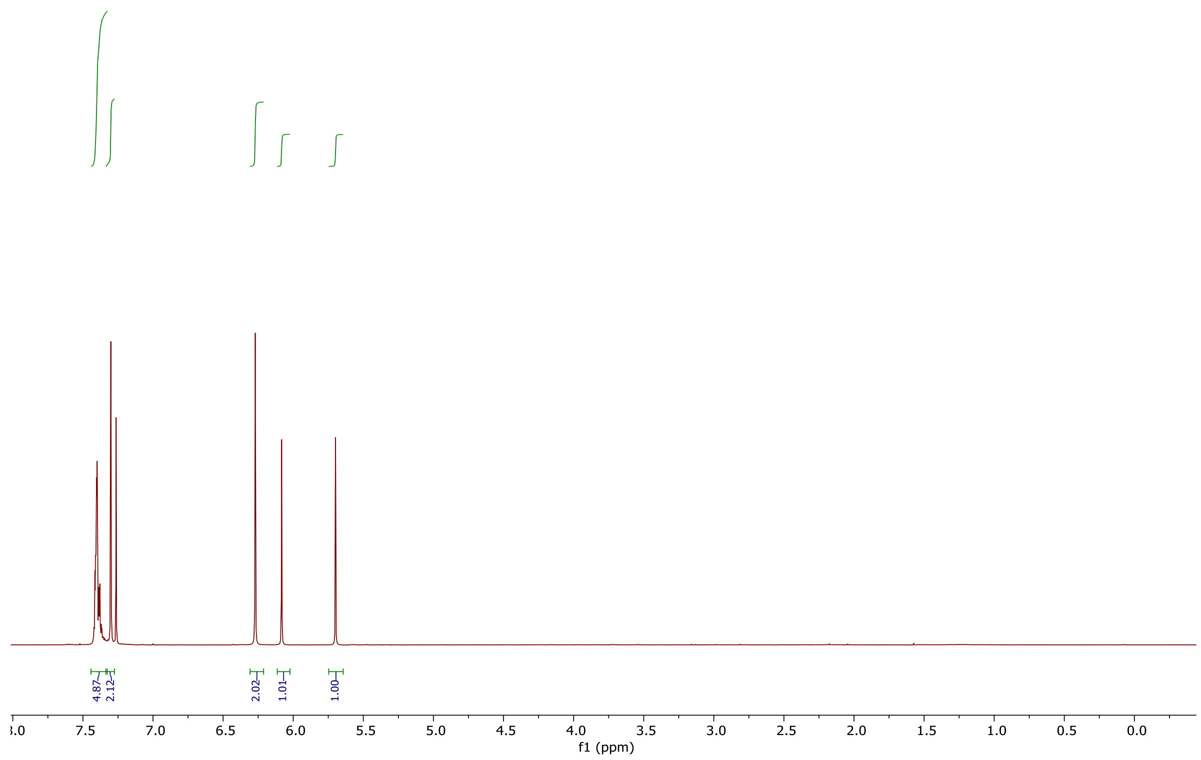

^{13}C-NMR: 2-phenyl-1-(1H-pyrrol-1-yl)prop-2-en-1-one (**S20**)

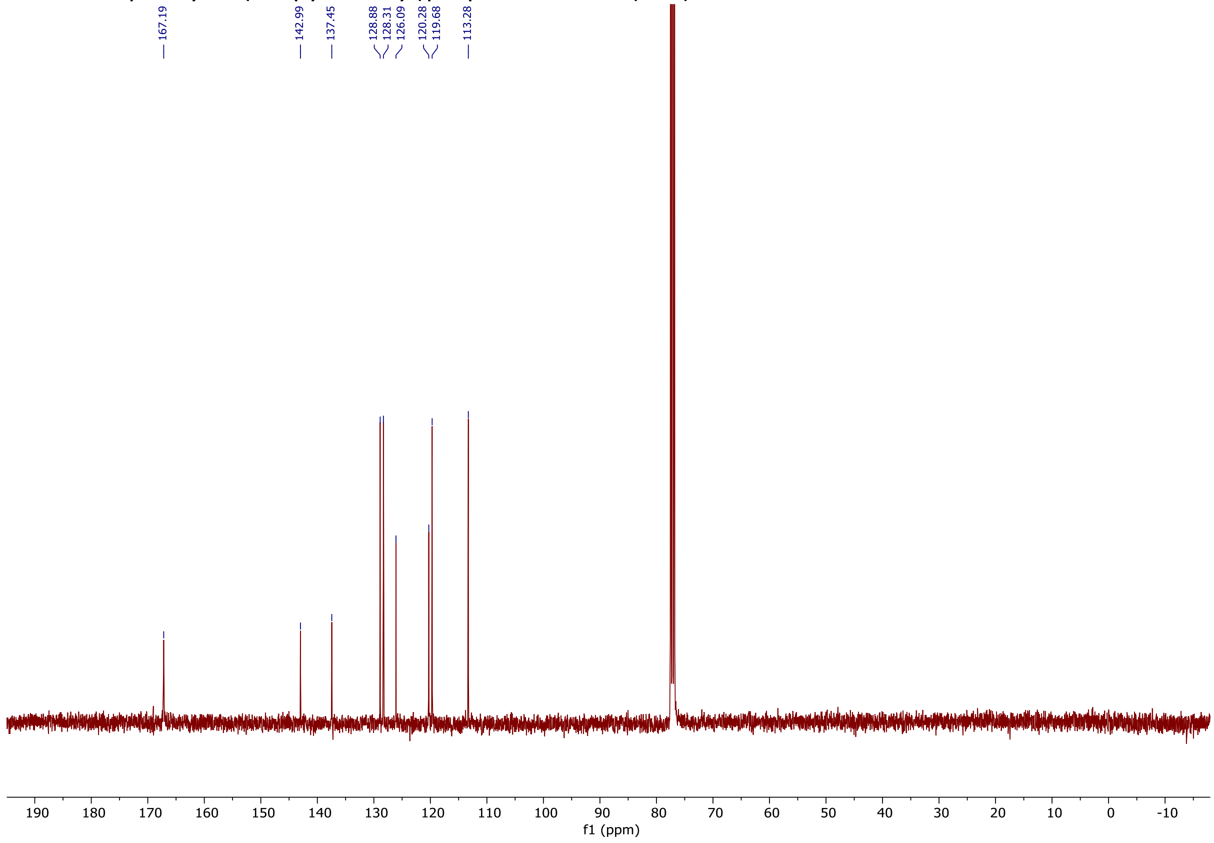

^{1}H-NMR: 2-phenyl-1,2-dihydro-3H-pyrrolizin-3-one (**21**)

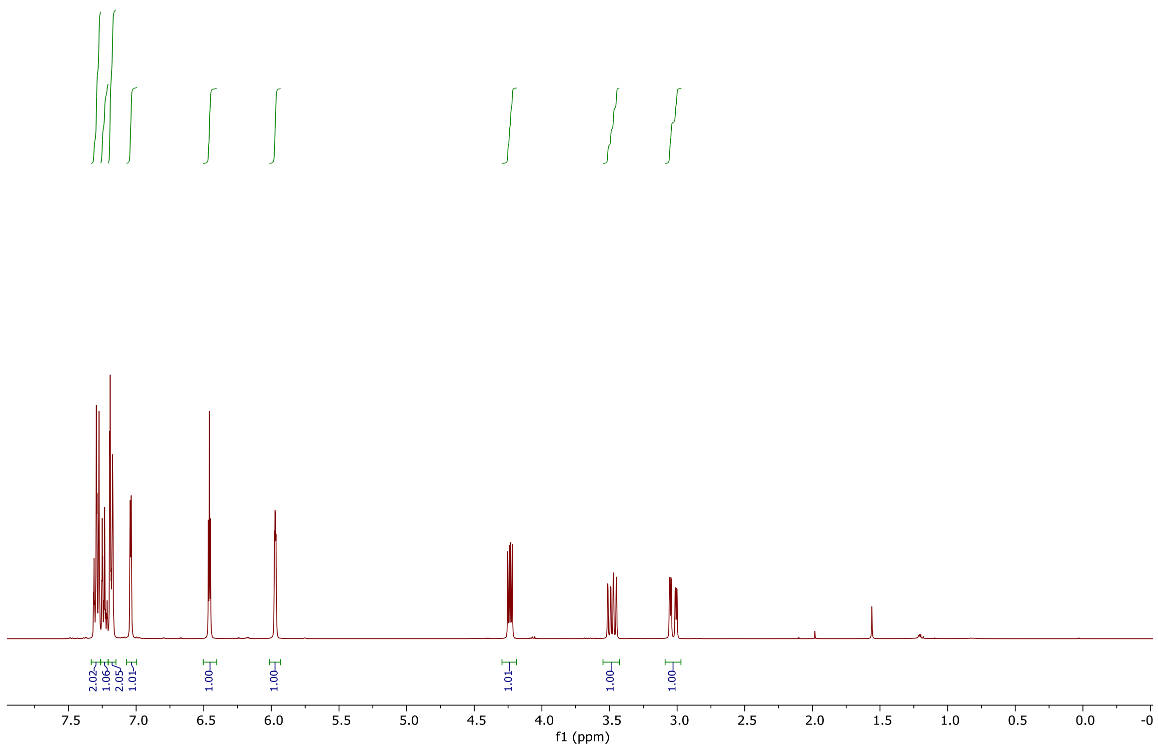

^{13}C-NMR: 2-phenyl-1,2-dihydro-3H-pyrrolizin-3-one (**21**)

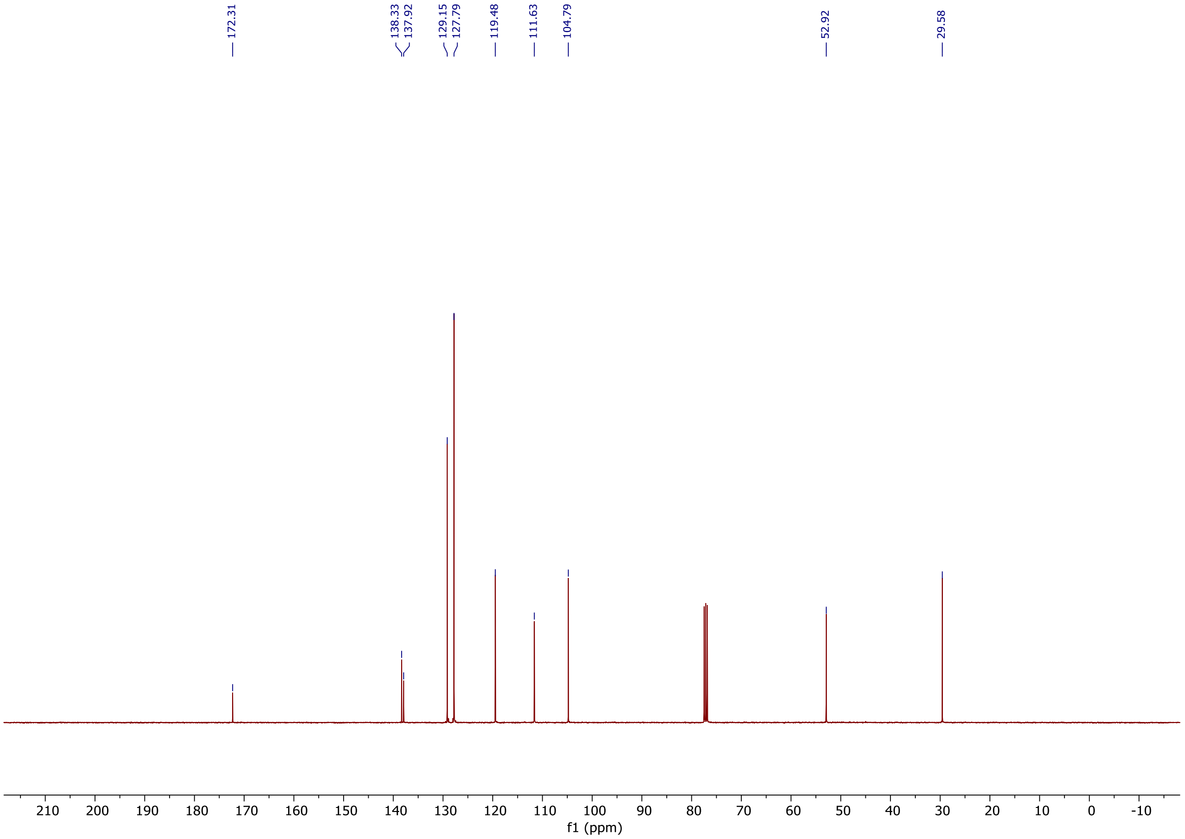

^{1}H-NMR: 1-(1H-imidazol-1-yl)-2-phenylprop-2-en-1-one (**S21**)

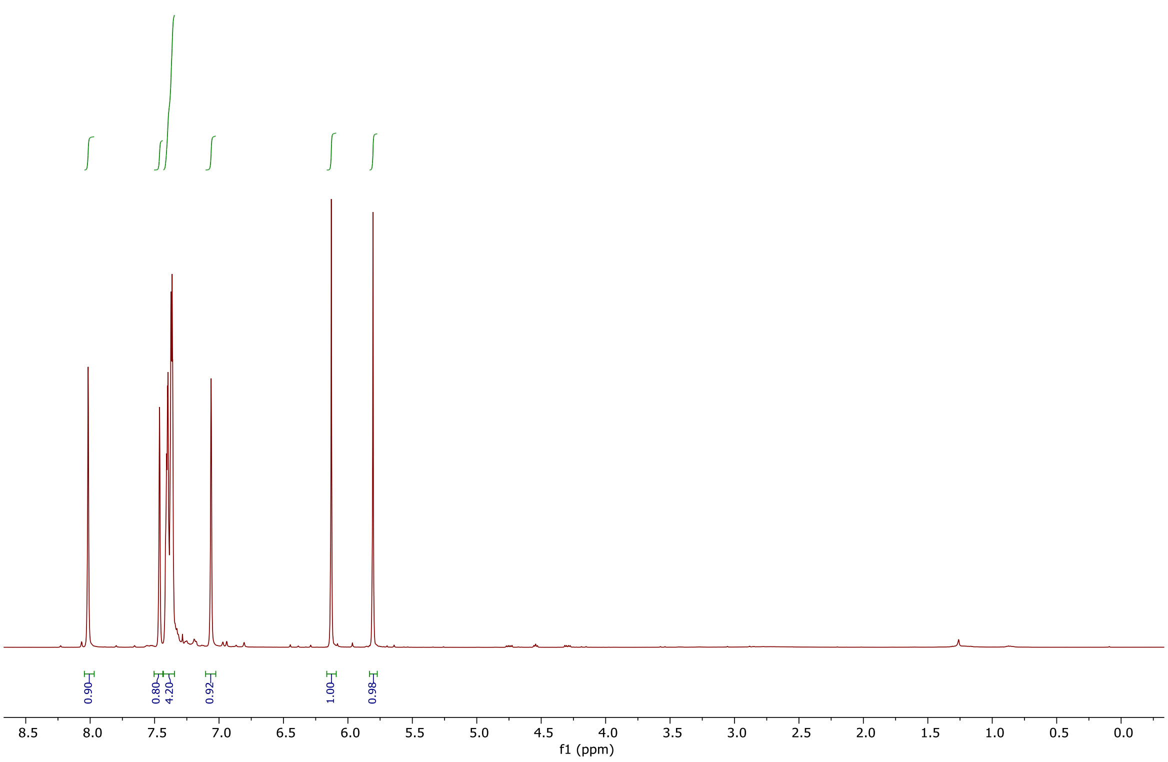

^{13}C-NMR: 1-(1H-imidazol-1-yl)-2-phenylprop-2-en-1-one (**S21**)

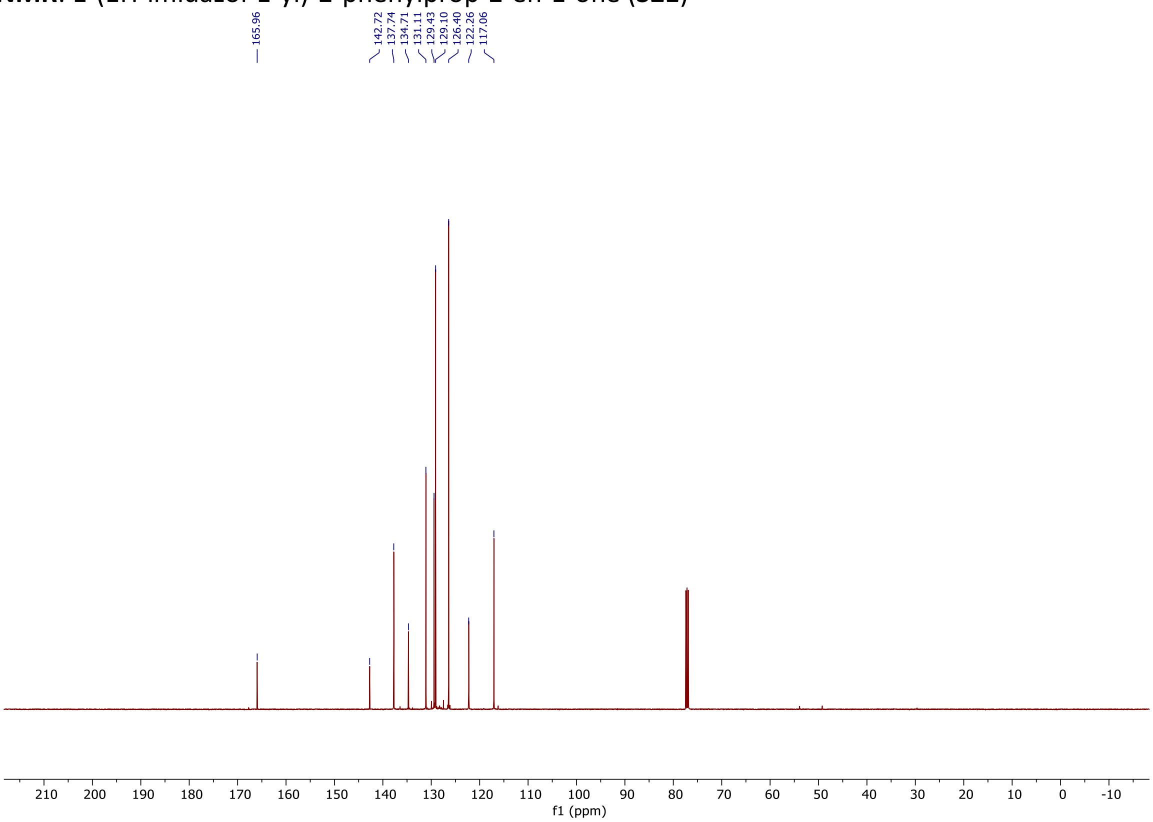

^{1}H-NMR: 6-phenyl-6,7-dihydro-5H-pyrrolo[1,2-c]imidazol-5-one and 6-phenyl-6,7-dihydro-5H-pyrrolo[1,2-a]imidazol-5-one (**22**)

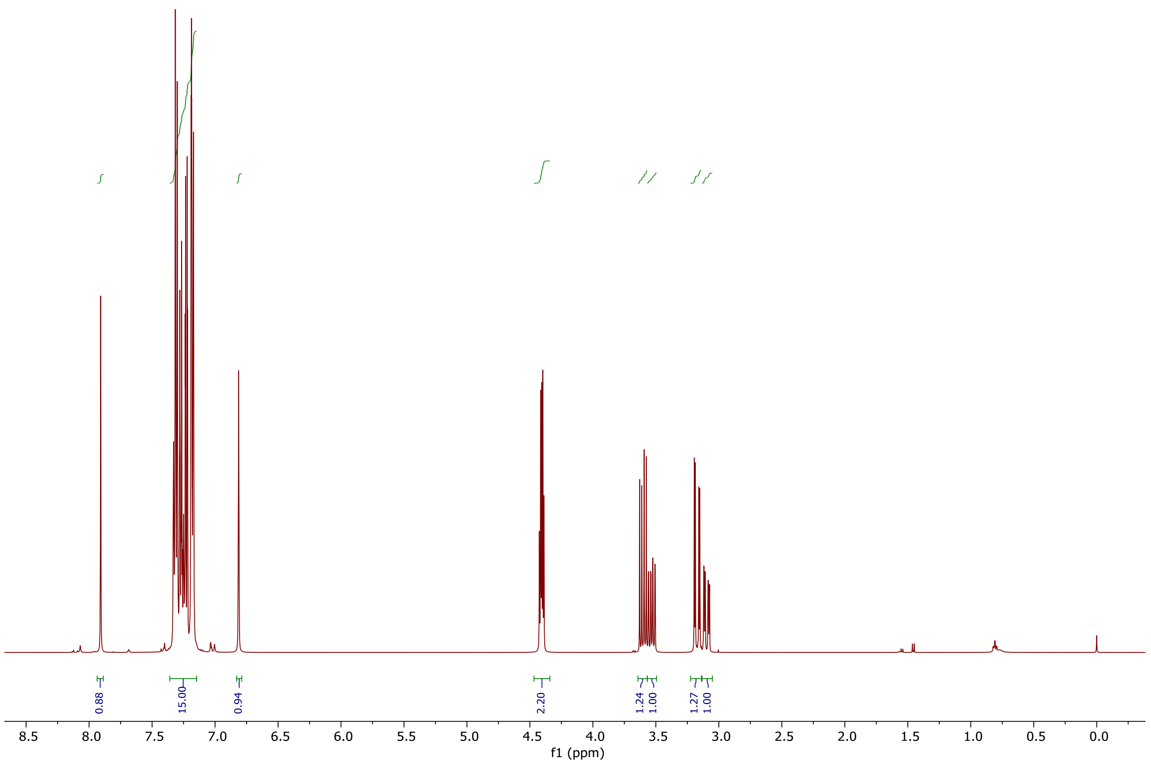

^{13}C-NMR: 6-phenyl-6,7-dihydro-5H-pyrrolo[1,2-c]imidazol-5-one and 6-phenyl-6,7-dihydro-5H-pyrrolo[1,2-a]imidazol-5-one (**22**)

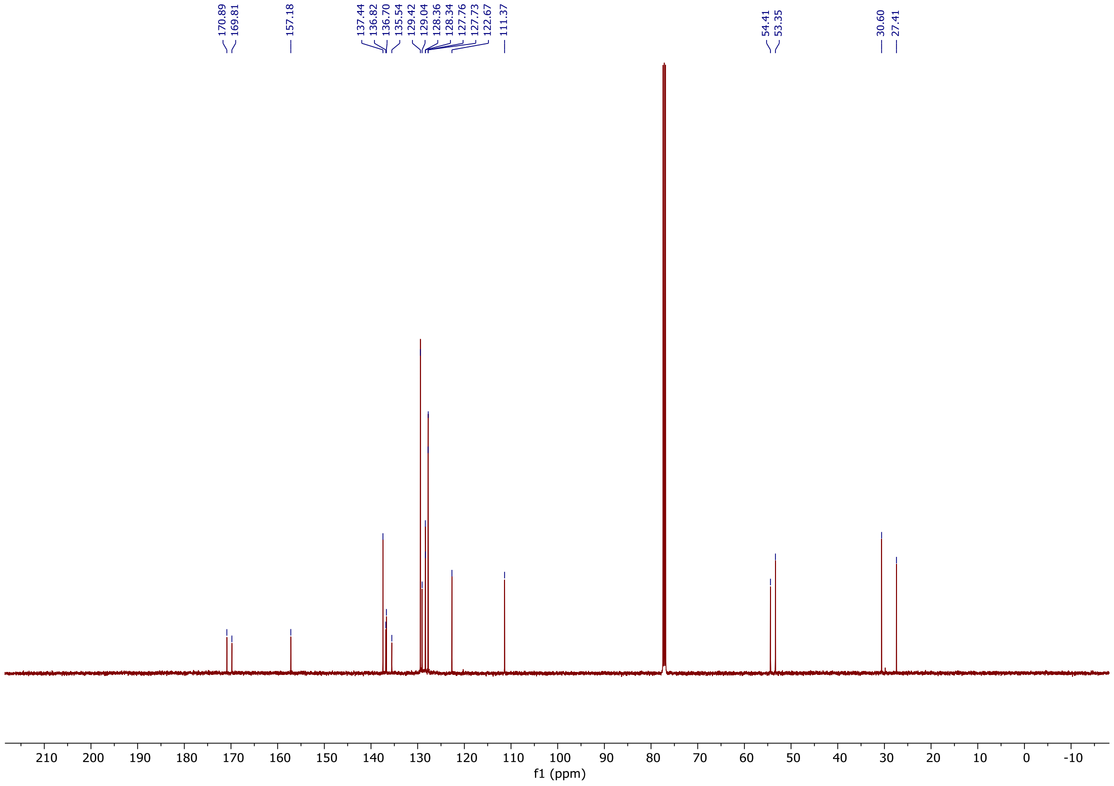

^{1}H-NMR: 2-(1-(2-phenylacryloyl)-1H-indol-3-yl)acetonitrile (**S22**)

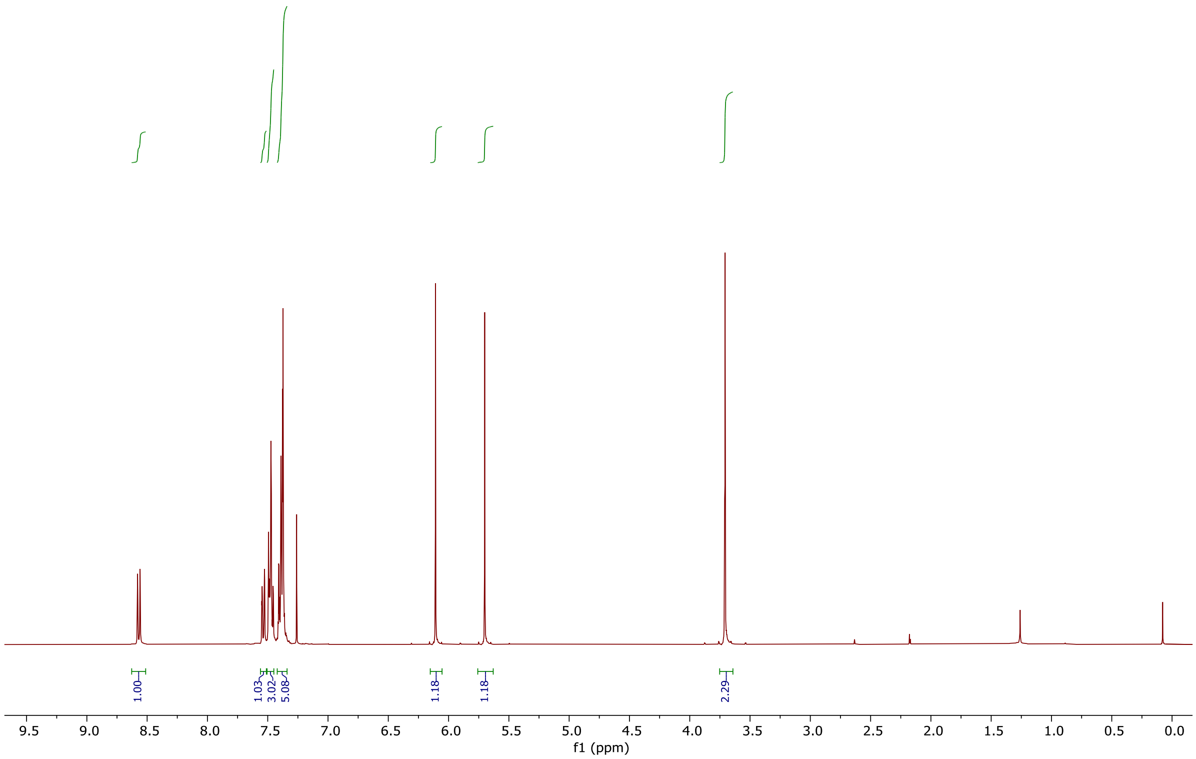

^{13}C-NMR: 2-(1-(2-phenylacryloyl)-1H-indol-3-yl)acetonitrile (**S22**)

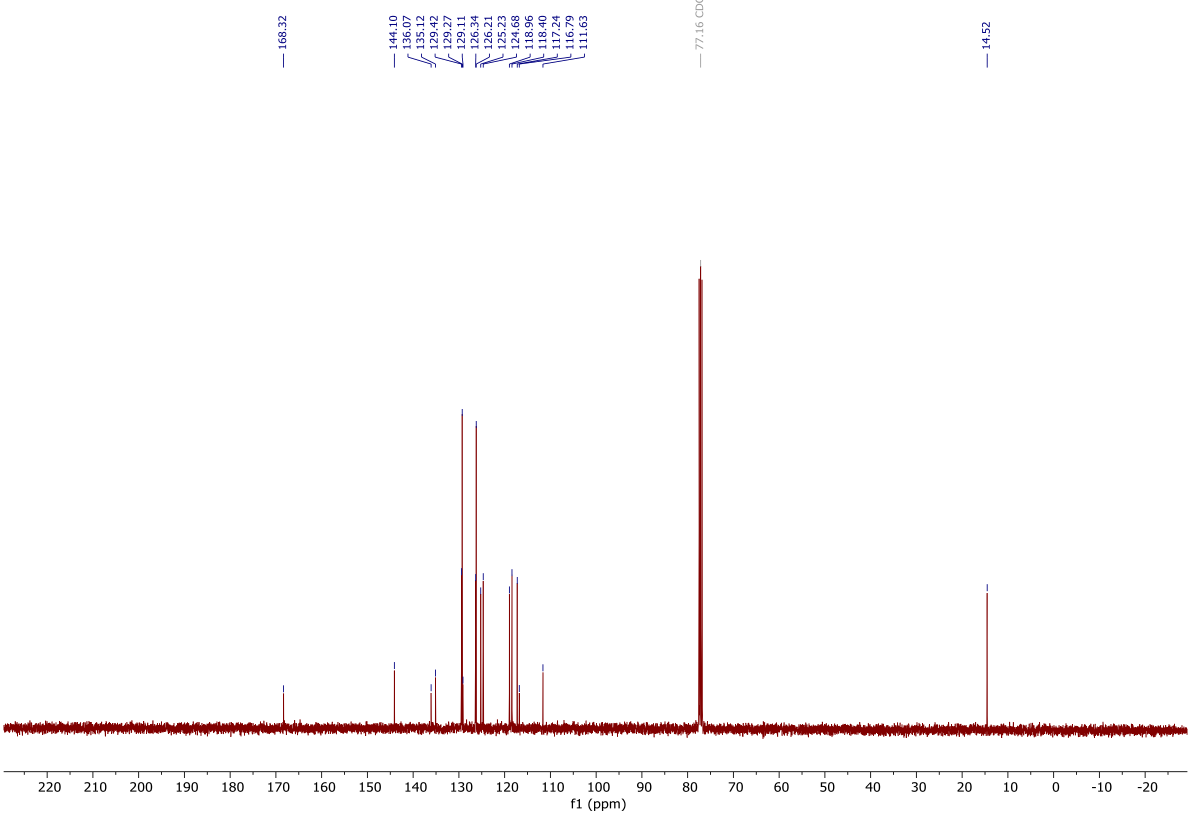

^{1}H-NMR: 2-(3-oxo-2-phenyl-2,3-dihydro-1H-pyrrolo[1,2-a]indol-9-yl)acetonitrile (**23**)

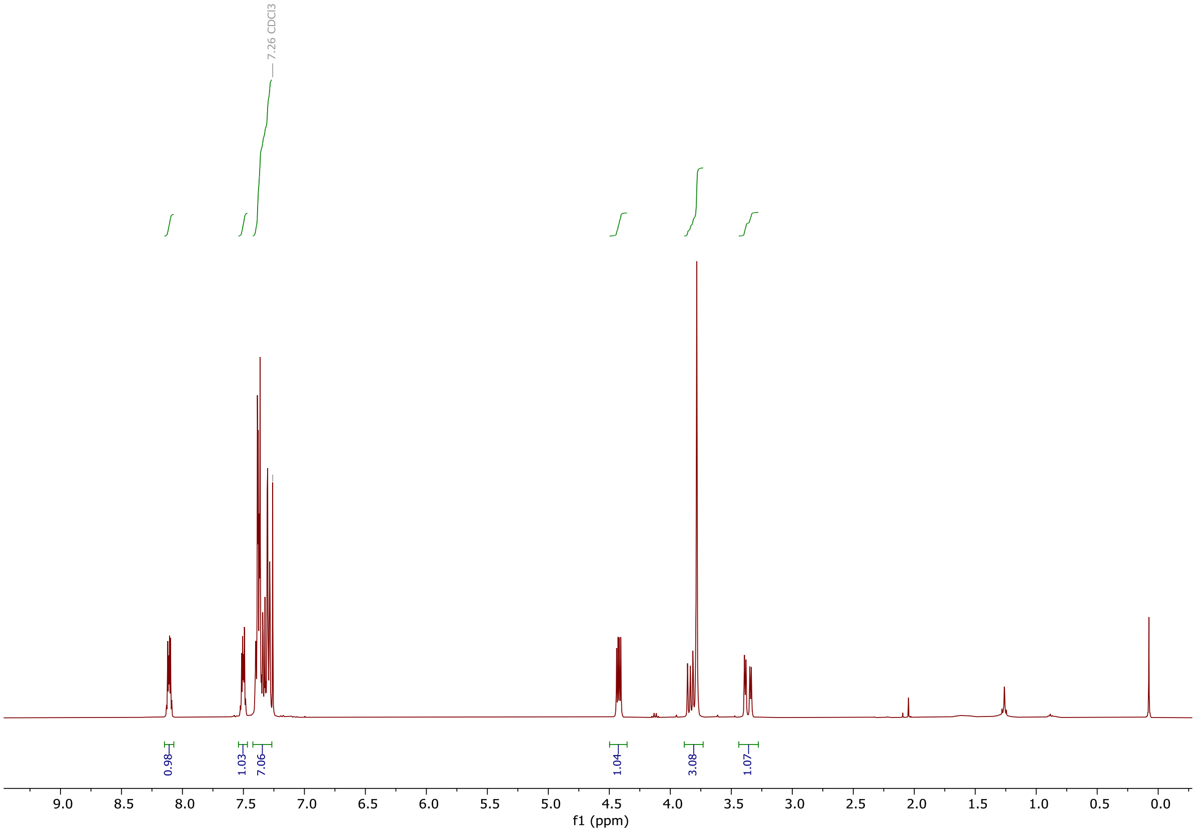

^{13}C-NMR: 2-(3-oxo-2-phenyl-2,3-dihydro-1H-pyrrolo[1,2-a]indol-9-yl)acetonitrile (**23**)

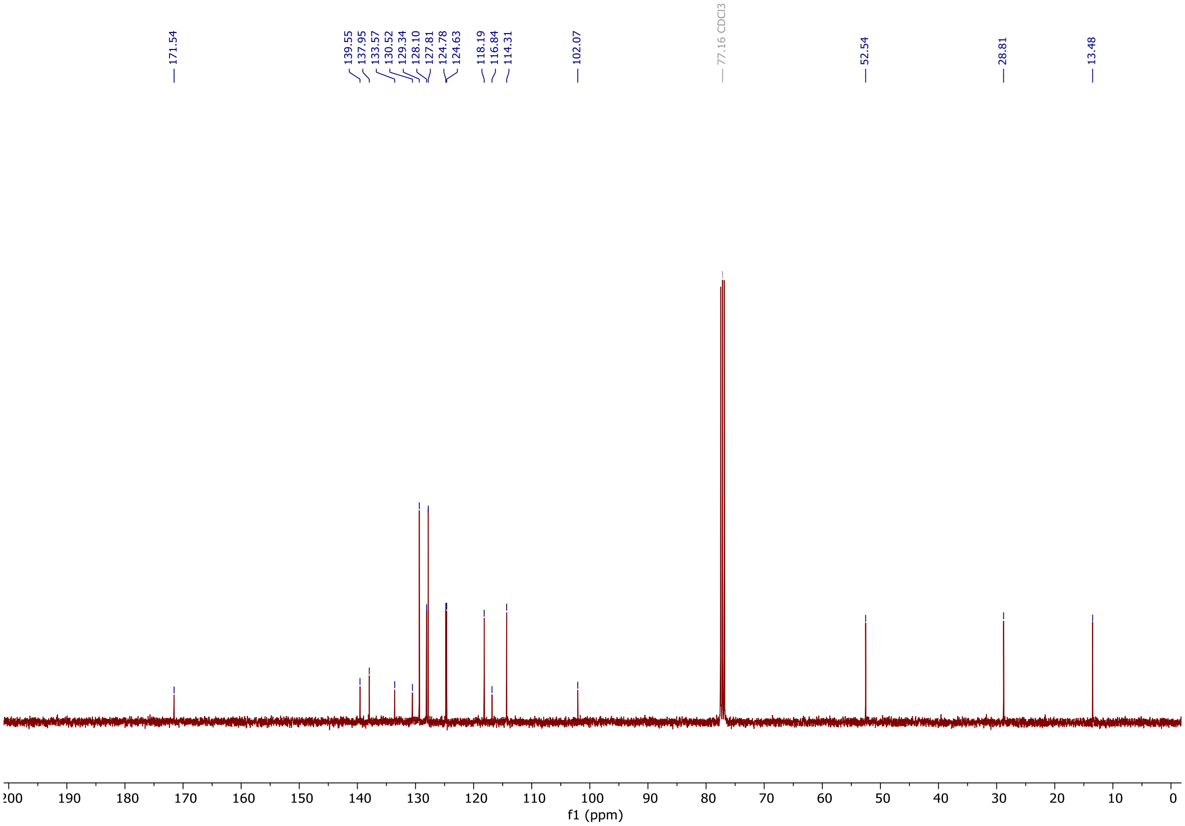

^{1}H-NMR: methyl 1-(2-phenylacryloyl)-1H-indole-3-carboxylate (**S23**)

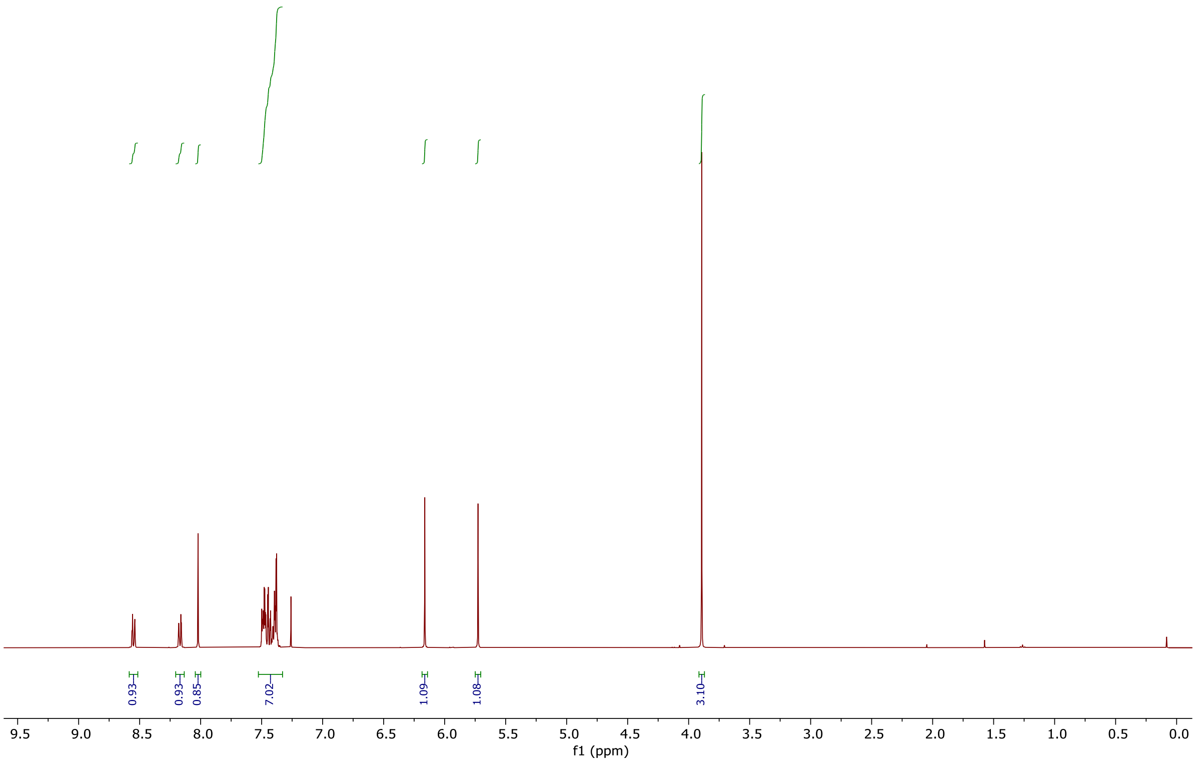

^{13}C-NMR: methyl 1-(2-phenylacryloyl)-1H-indole-3-carboxylate (**S23**)

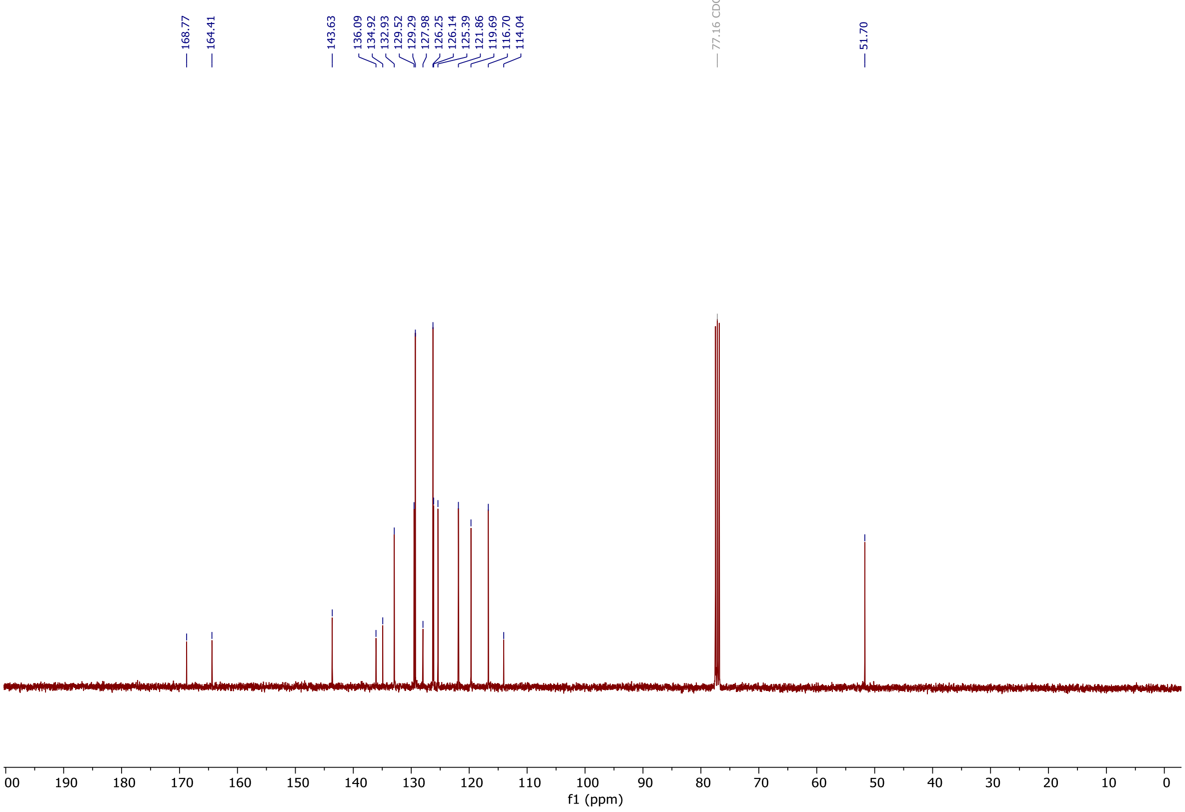

¹H-NMR: methyl 3-oxo-2-phenyl-2,3-dihydro-1H-pyrrolo[1,2-a]indole-9-carboxylate (**24**)

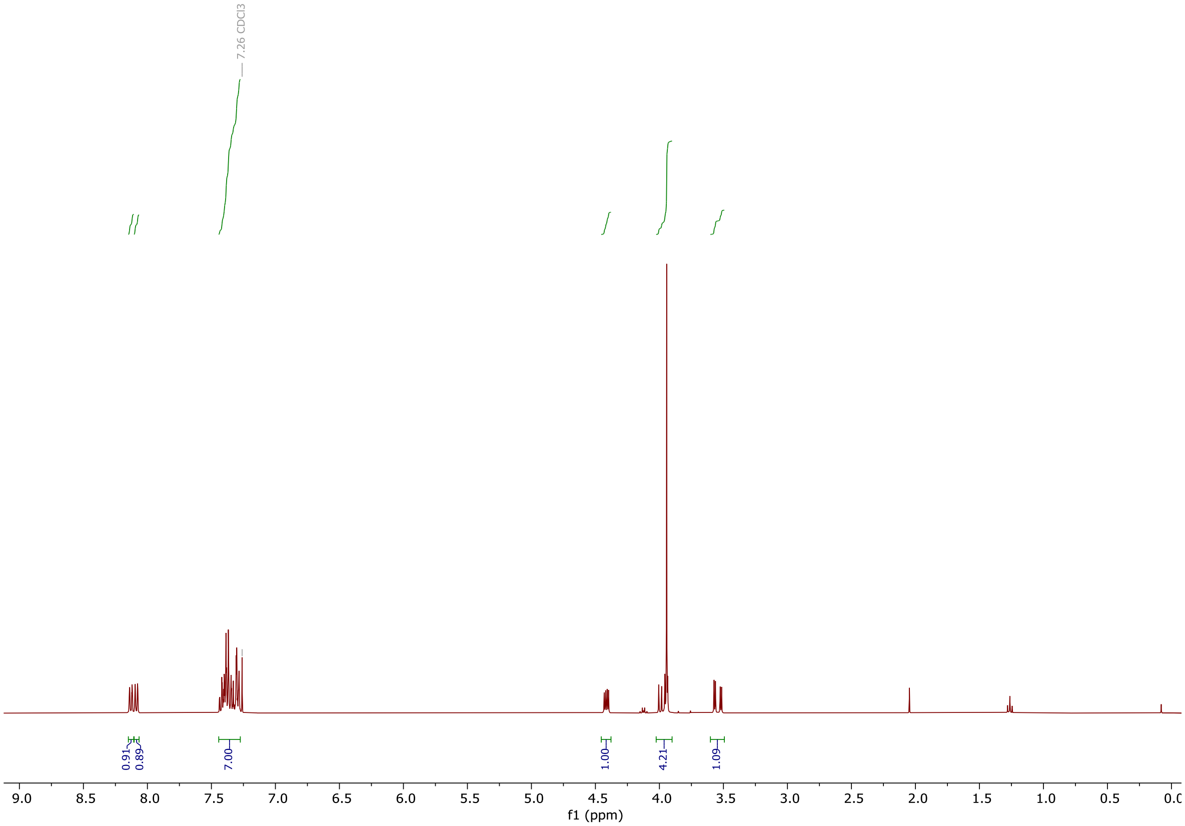

¹³C-NMR: methyl 3-oxo-2-phenyl-2,3-dihydro-1H-pyrrolo[1,2-a]indole-9-carboxylate (**24**)

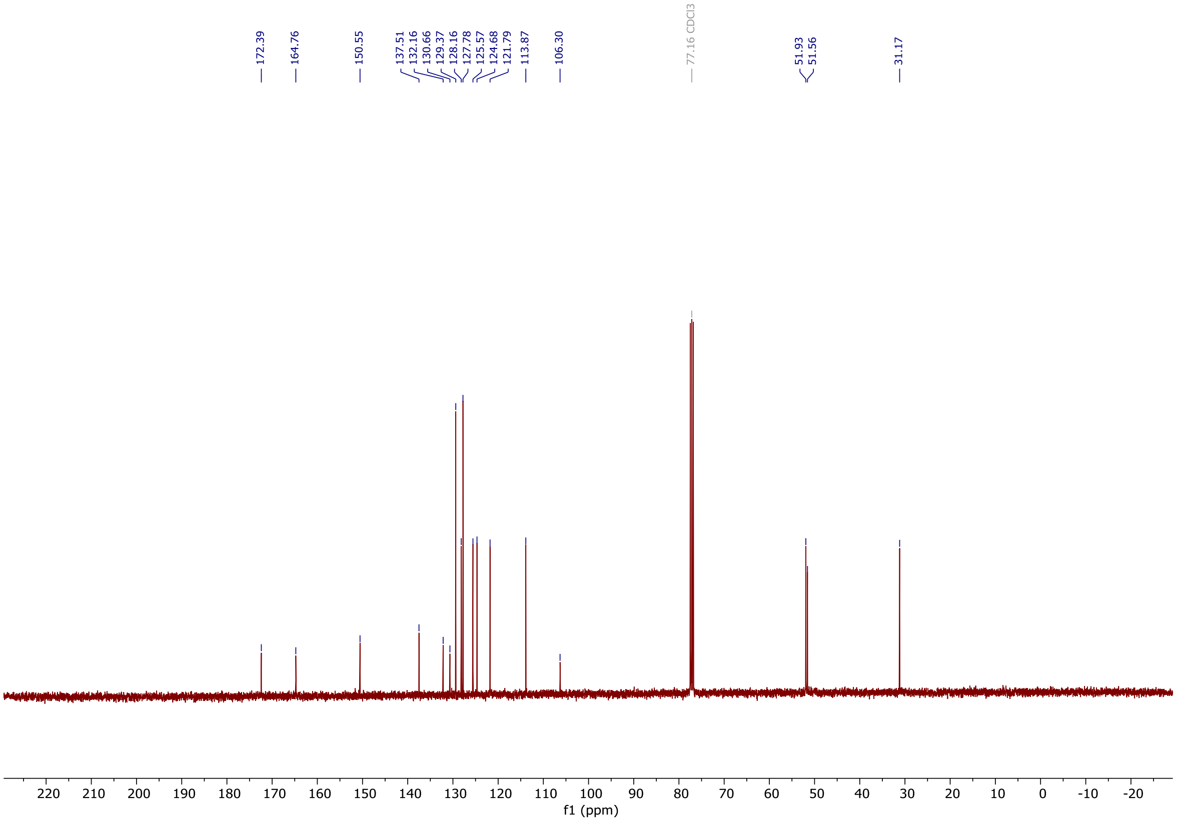

^{1}H-NMR: 2-(1-(2-methoxyacryloyl)-1H-indol-3-yl)acetonitrile (**S24**)

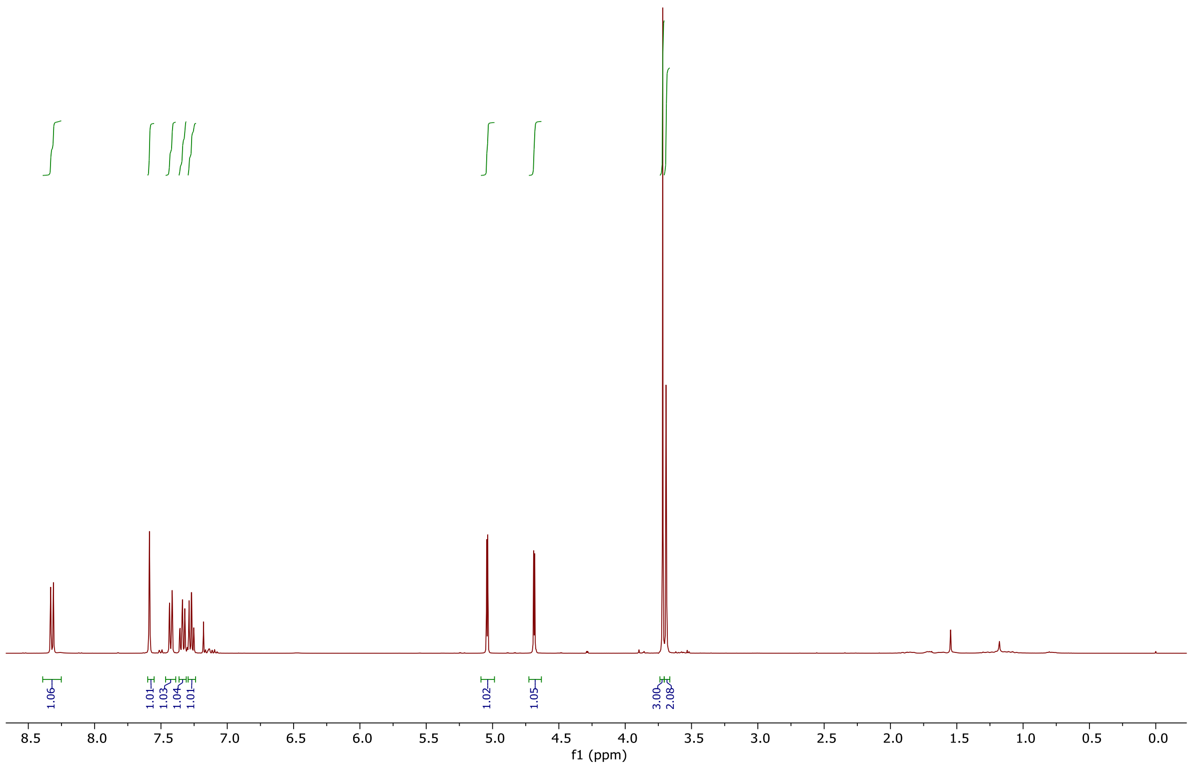

^{13}C-NMR: 2-(1-(2-methoxyacryloyl)-1H-indol-3-yl)acetonitrile (**S24**)

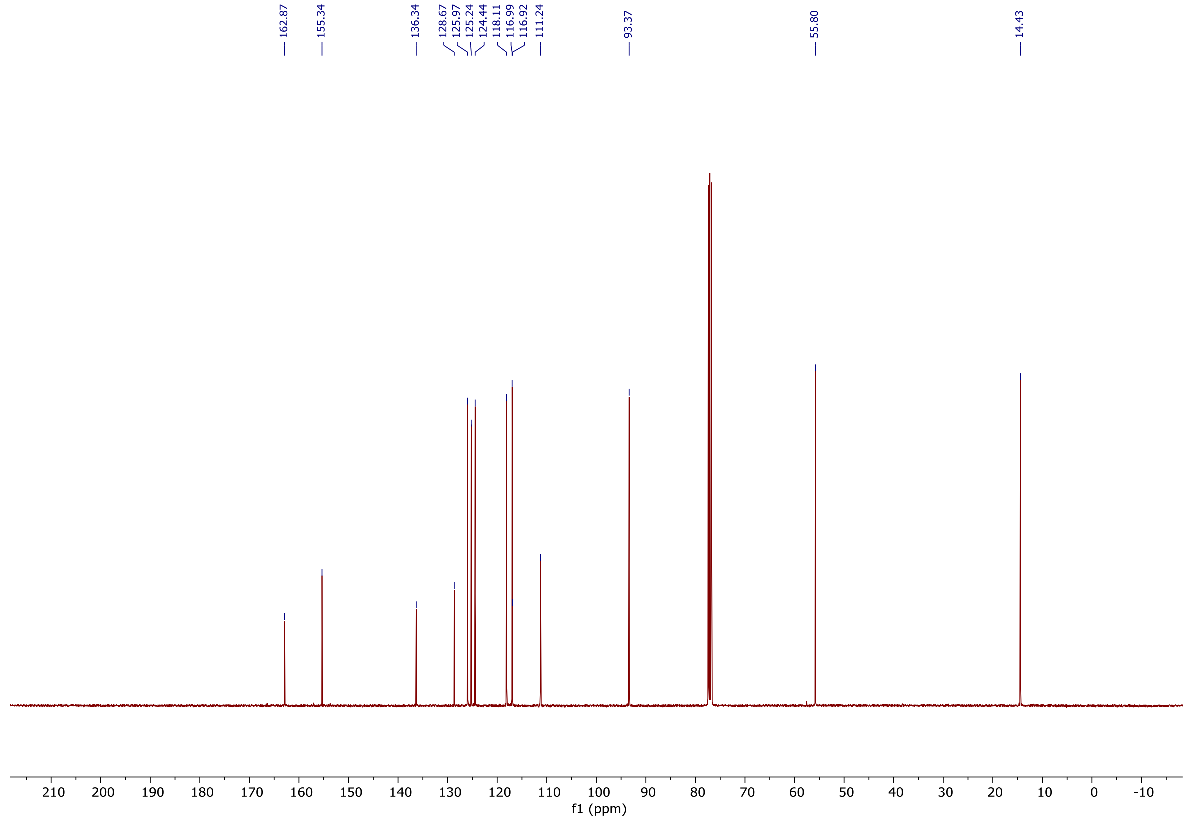

^{1}H-NMR: 2-(2-methoxy-3-oxo-2,3-dihydro-1H-pyrrolo[1,2-a]indol-8-yl)acetonitrile (**25**)

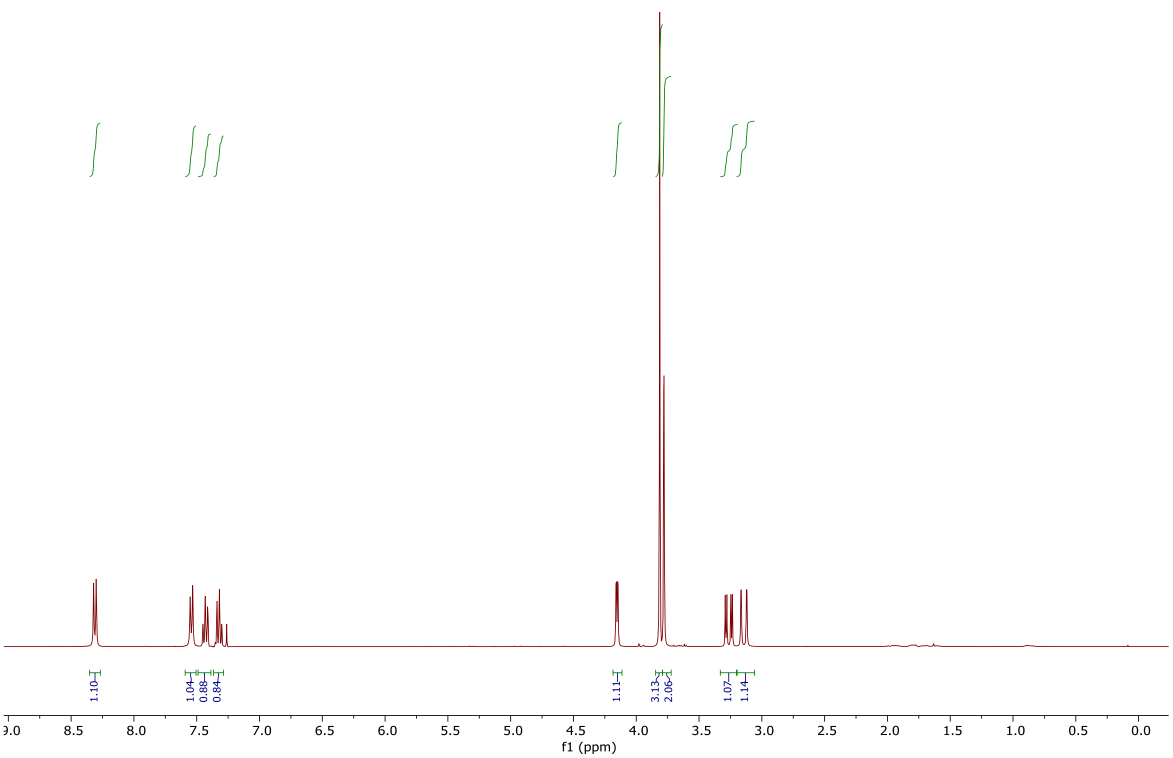

^{13}C-NMR: 2-(2-methoxy-3-oxo-2,3-dihydro-1H-pyrrolo[1,2-a]indol-8-yl)acetonitrile (**25**)

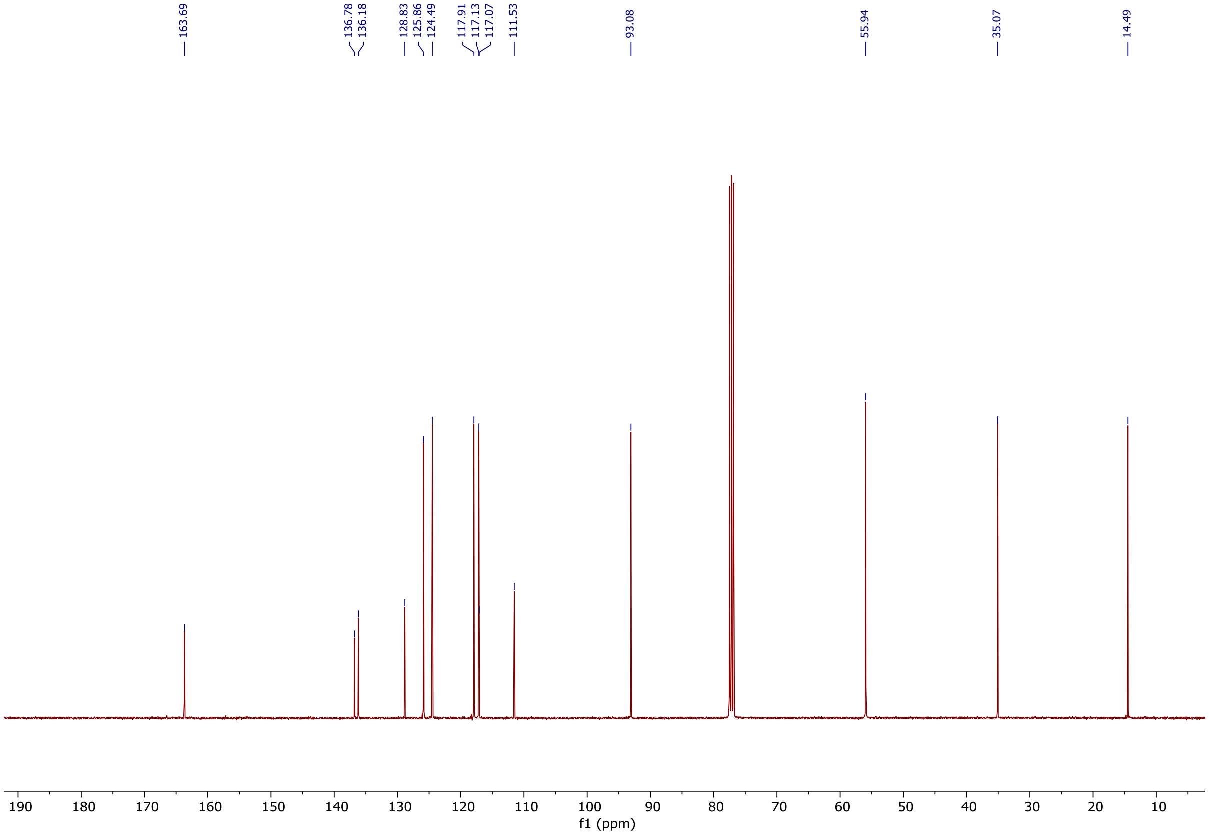

^{1}H-NMR: 2-(1-(2-(4-methoxyphenyl)acryloyl)-1H-indol-3-yl)acetonitrile (**S25**)

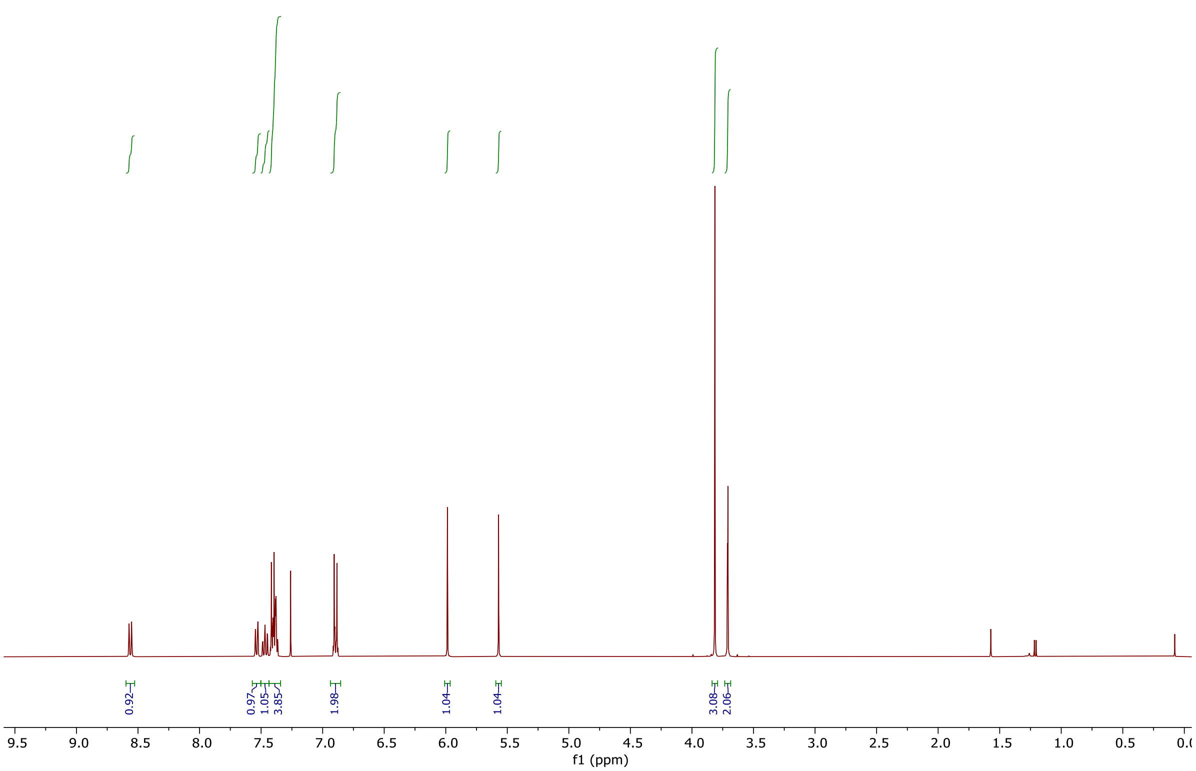

^{13}C-NMR: 2-(1-(2-(4-methoxyphenyl)acryloyl)-1H-indol-3-yl)acetonitrile (**S25**)

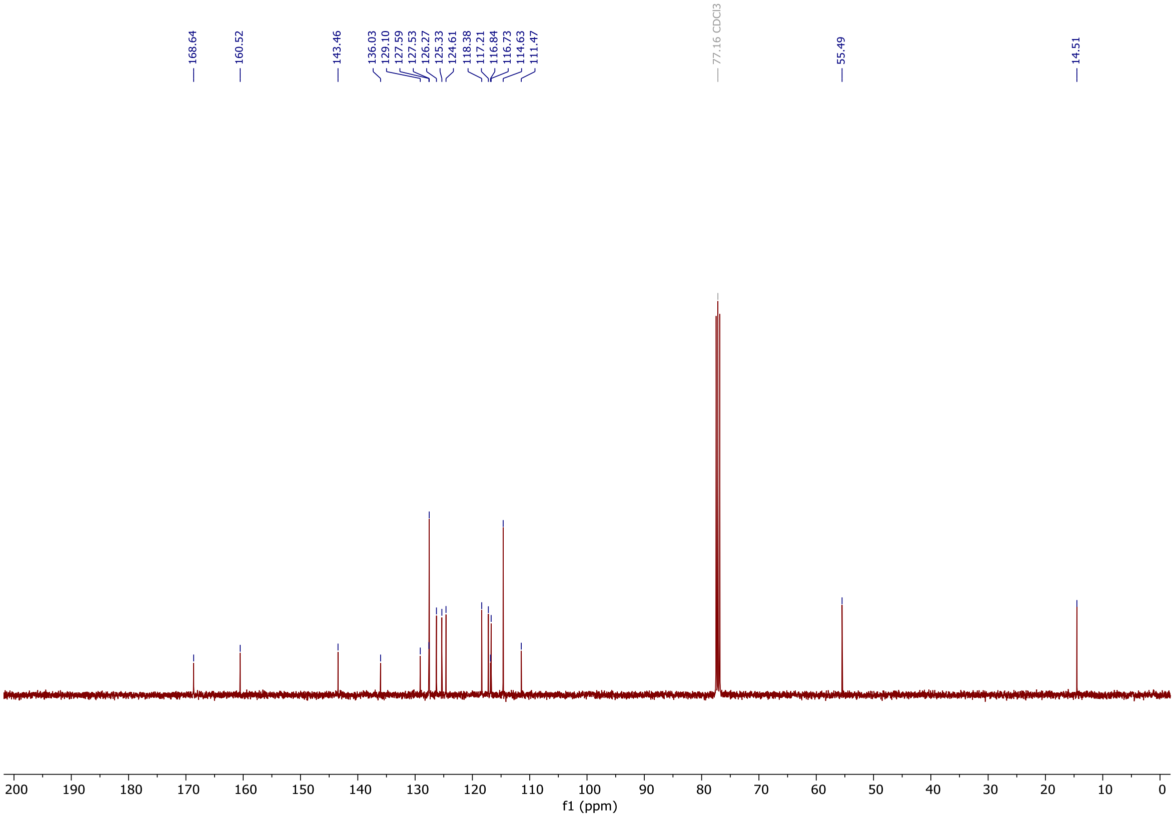

^{1}H-NMR: 2-(2-(4-methoxyphenyl)-3-oxo-2,3-dihydro-1H-pyrrolo[1,2-a]indol-9-yl)acetonitrile (**26**)

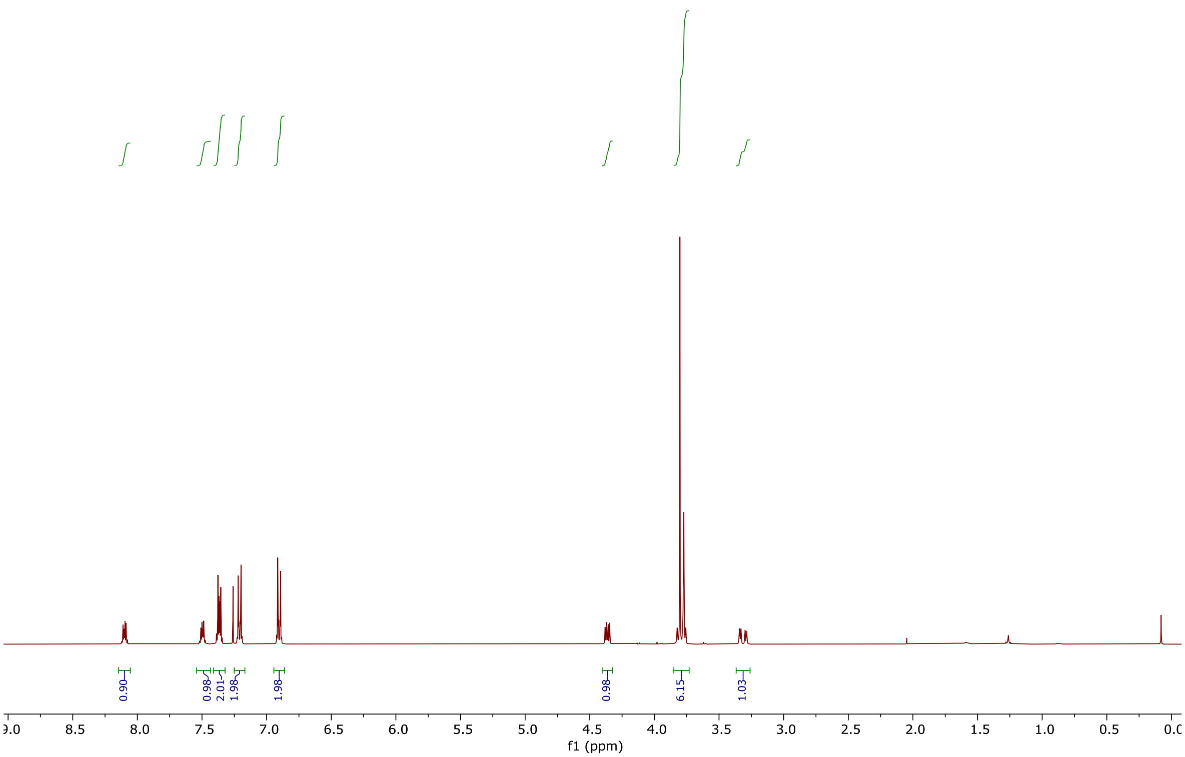

^{13}C-NMR: 2-(2-(4-methoxyphenyl)-3-oxo-2,3-dihydro-1H-pyrrolo[1,2-a]indol-9-yl)acetonitrile (**26**)

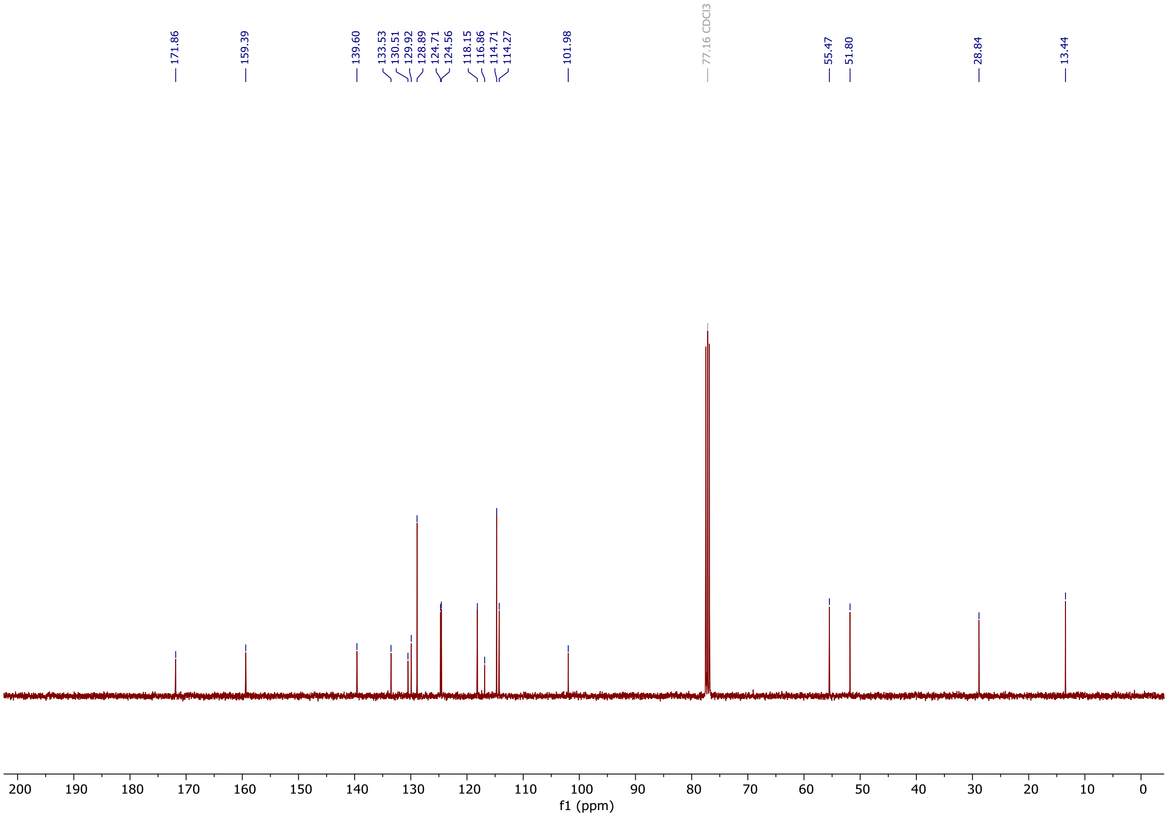

^{1}H-NMR: 1-(2-(4-(tert-butyl)phenyl)acryloyl)-1H-indole-5-carbonitrile (**S26**)

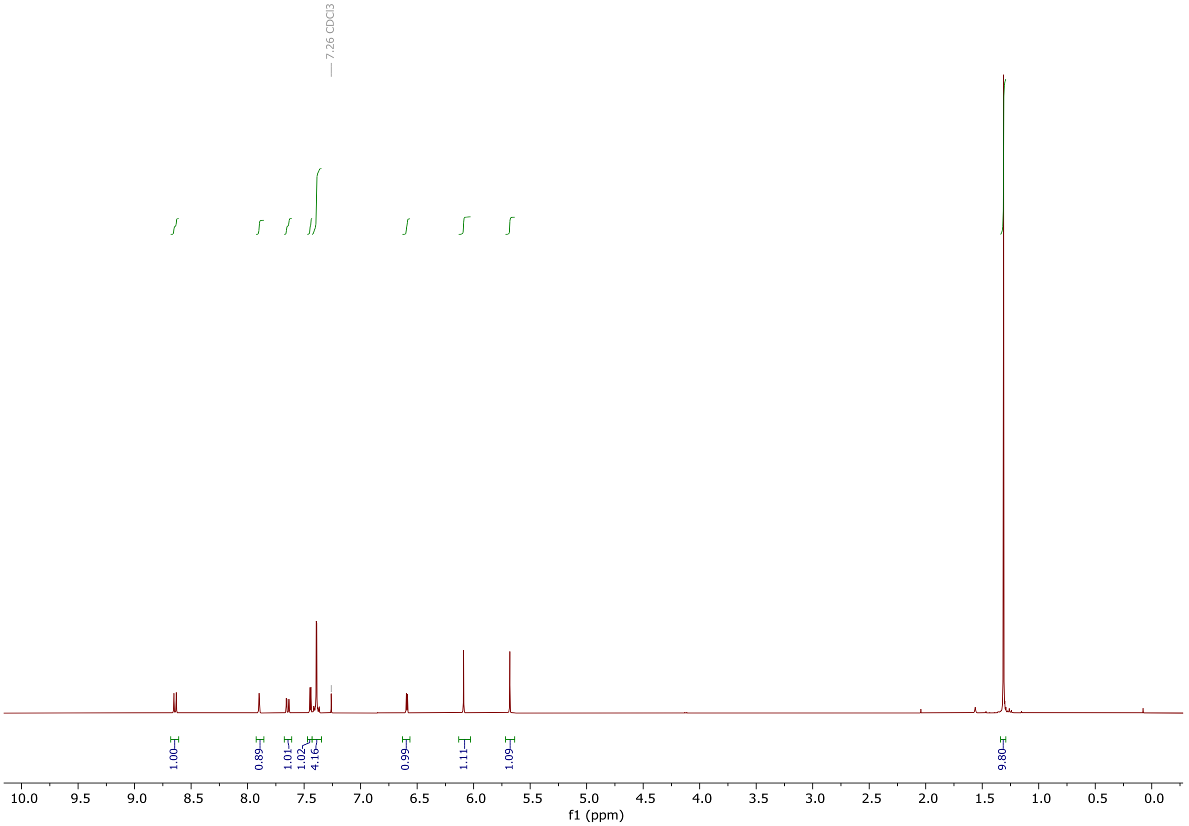

^{13}C-NMR: 1-(2-(4-(tert-butyl)phenyl)acryloyl)-1H-indole-5-carbonitrile (**S26**)

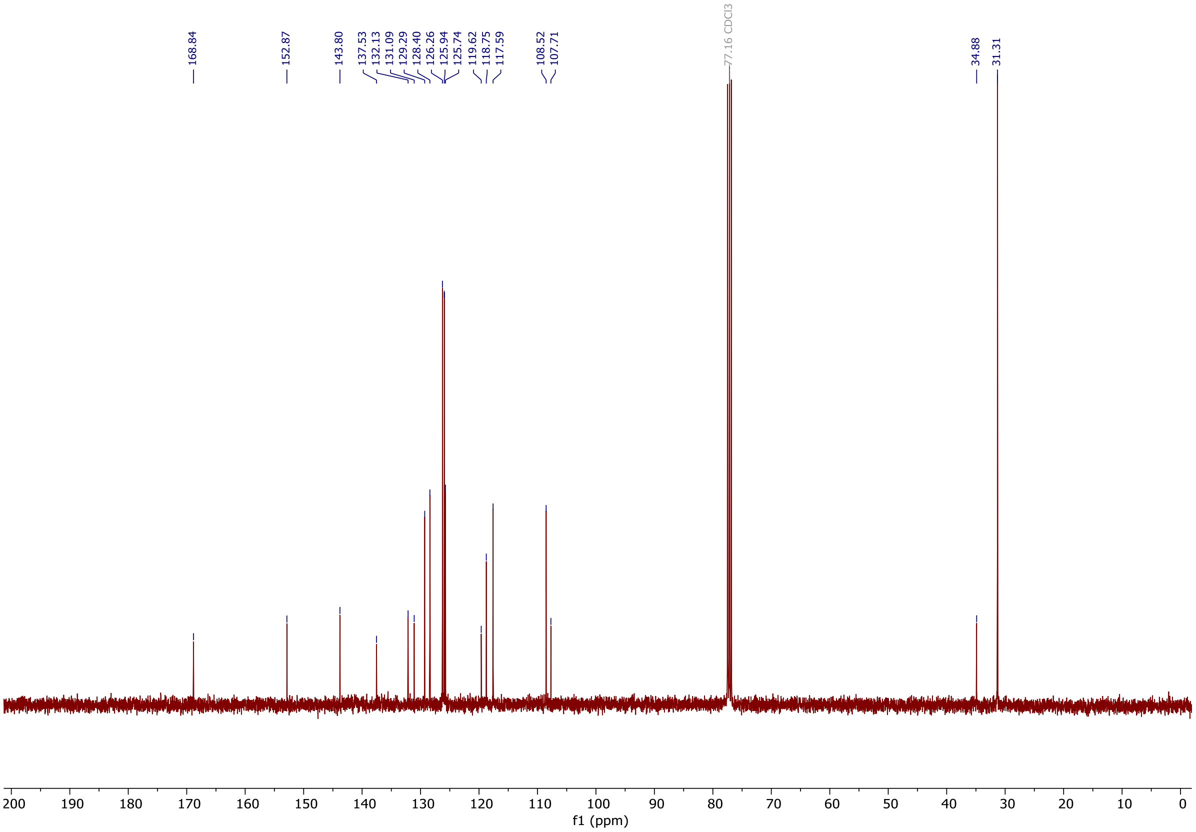

^{1}H-NMR: 2-(4-(tert-butyl)phenyl)-3-oxo-2,3-dihydro-1H-pyrrolo[1,2-a]indole-7-carbonitrile (**27**)

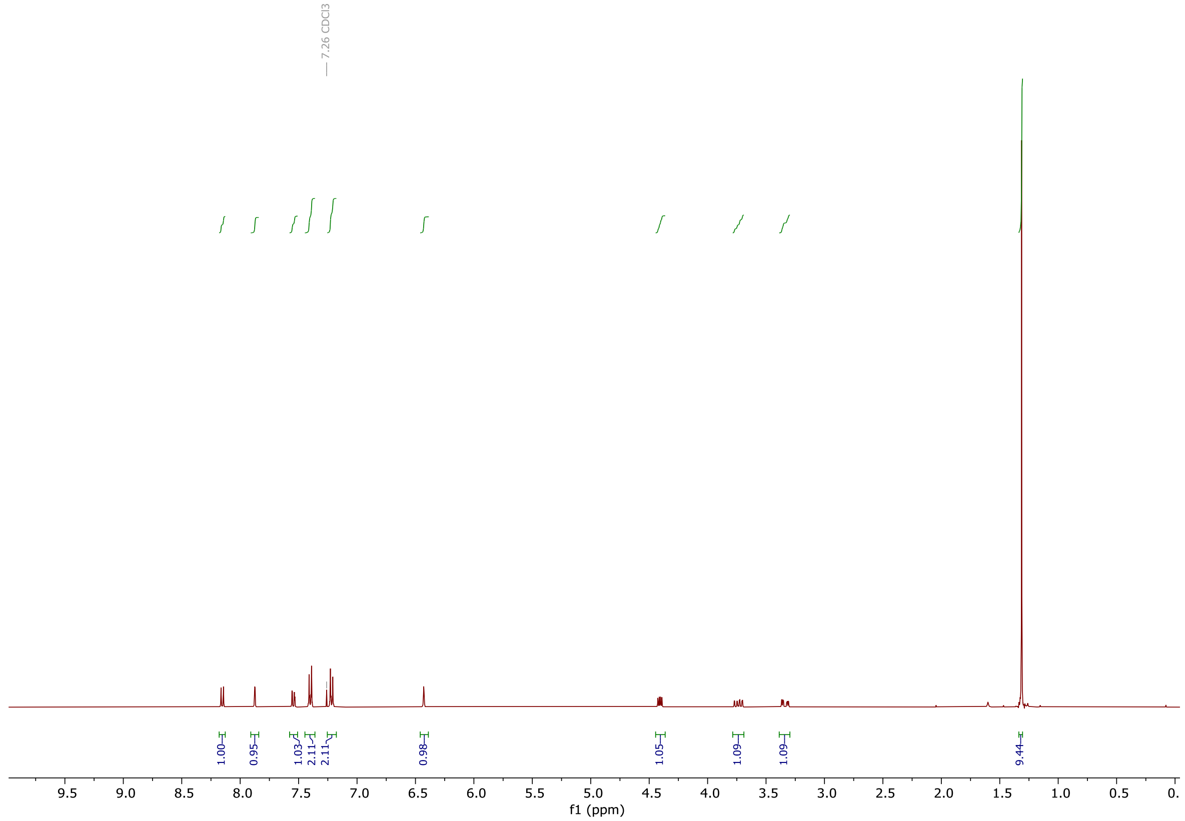

^{13}C-NMR: 2-(4-(tert-butyl)phenyl)-3-oxo-2,3-dihydro-1H-pyrrolo[1,2-a]indole-7-carbonitrile (**27**)

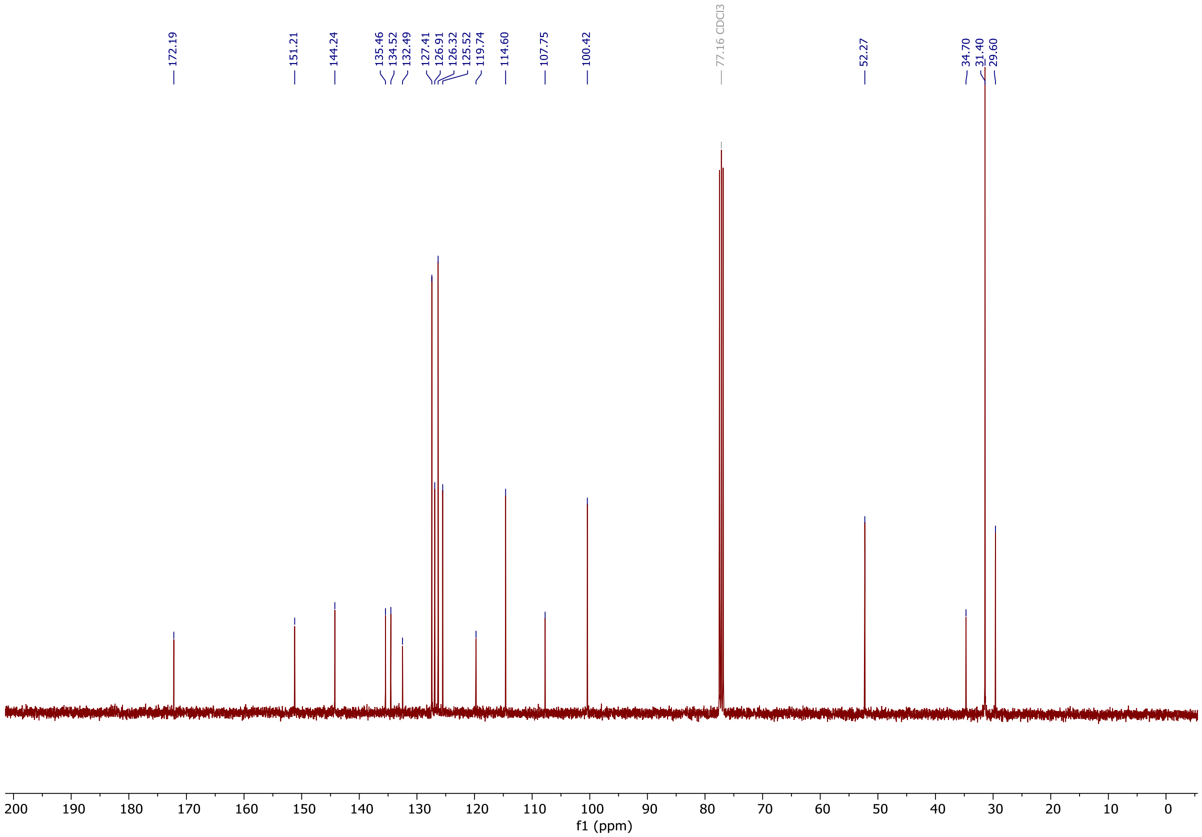

1H-NMR: 1-(2-(4-(trifluoromethyl)phenyl)acryloyl)-1H-indole-5-carbonitrile (**S27**)

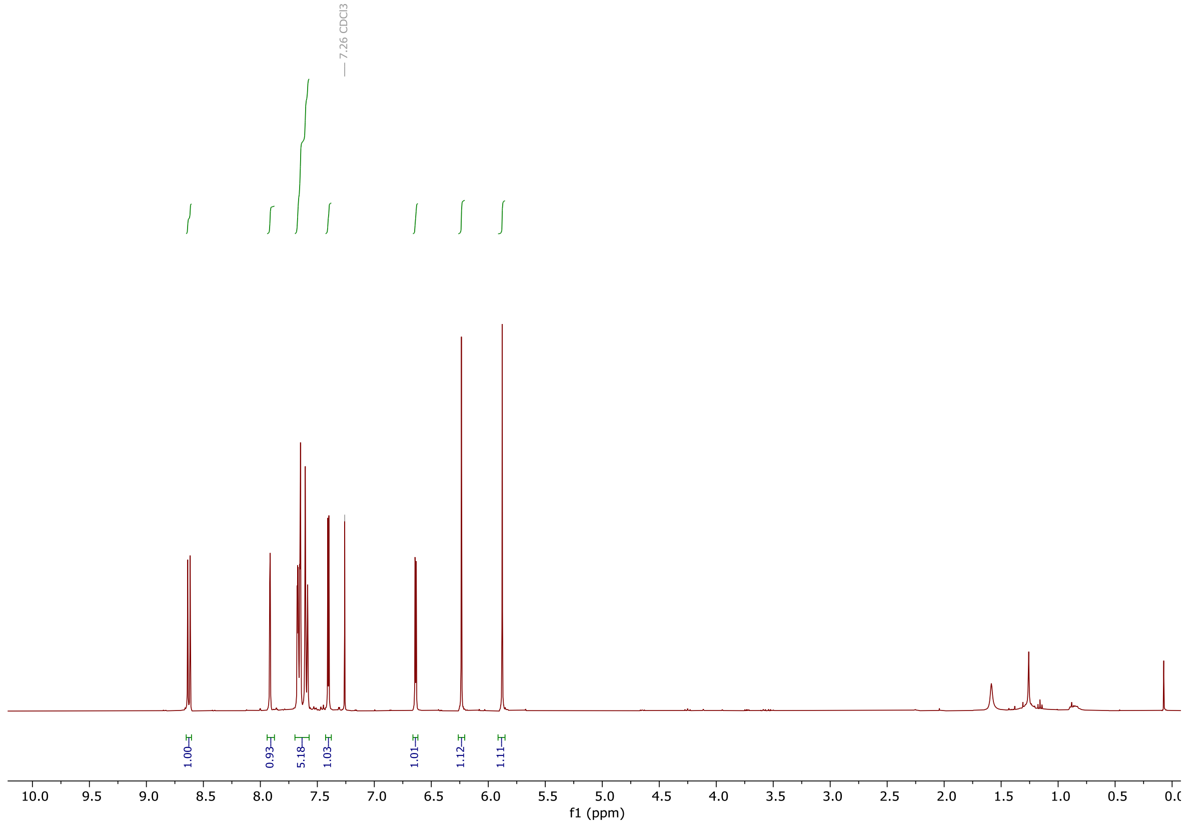

13C-NMR: 1-(2-(4-(trifluoromethyl)phenyl)acryloyl)-1H-indole-5-carbonitrile (**S27**)

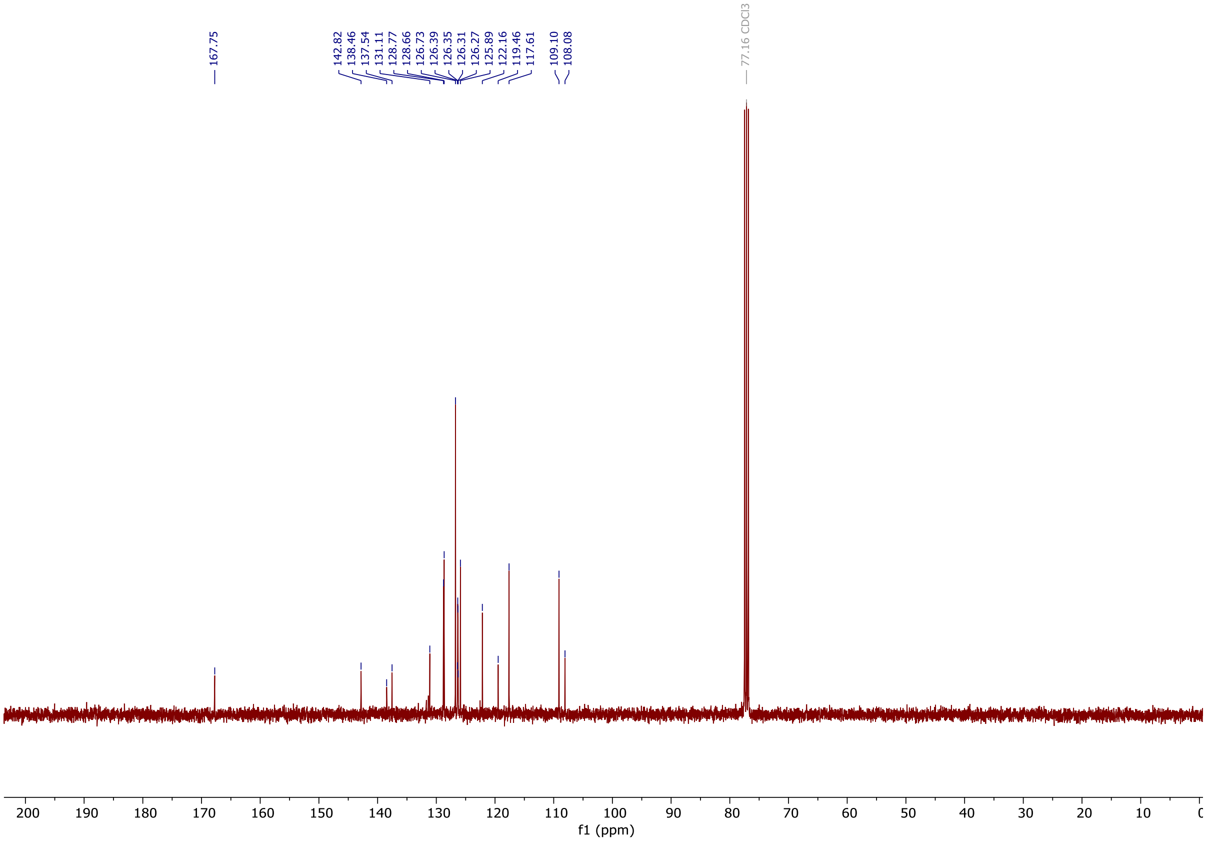

^{19}F-NMR: 1-(2-(4-(trifluoromethyl)phenyl)acryloyl)-1H-indole-5-carbonitrile (**S27**)

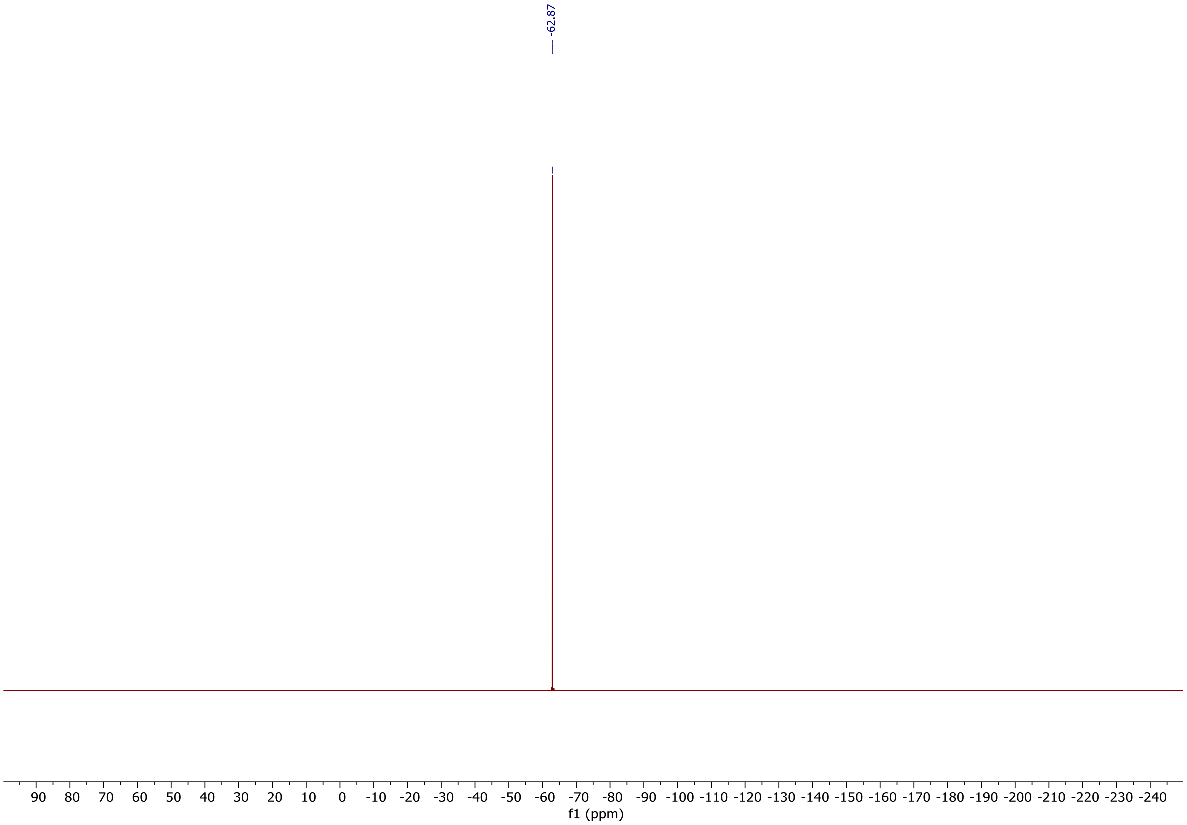

^{1}H-NMR: 3-oxo-2-(4-(trifluoromethyl)phenyl)-2,3-dihydro-1H-pyrrolo[1,2-a]indole-7-carbonitrile (**28**)

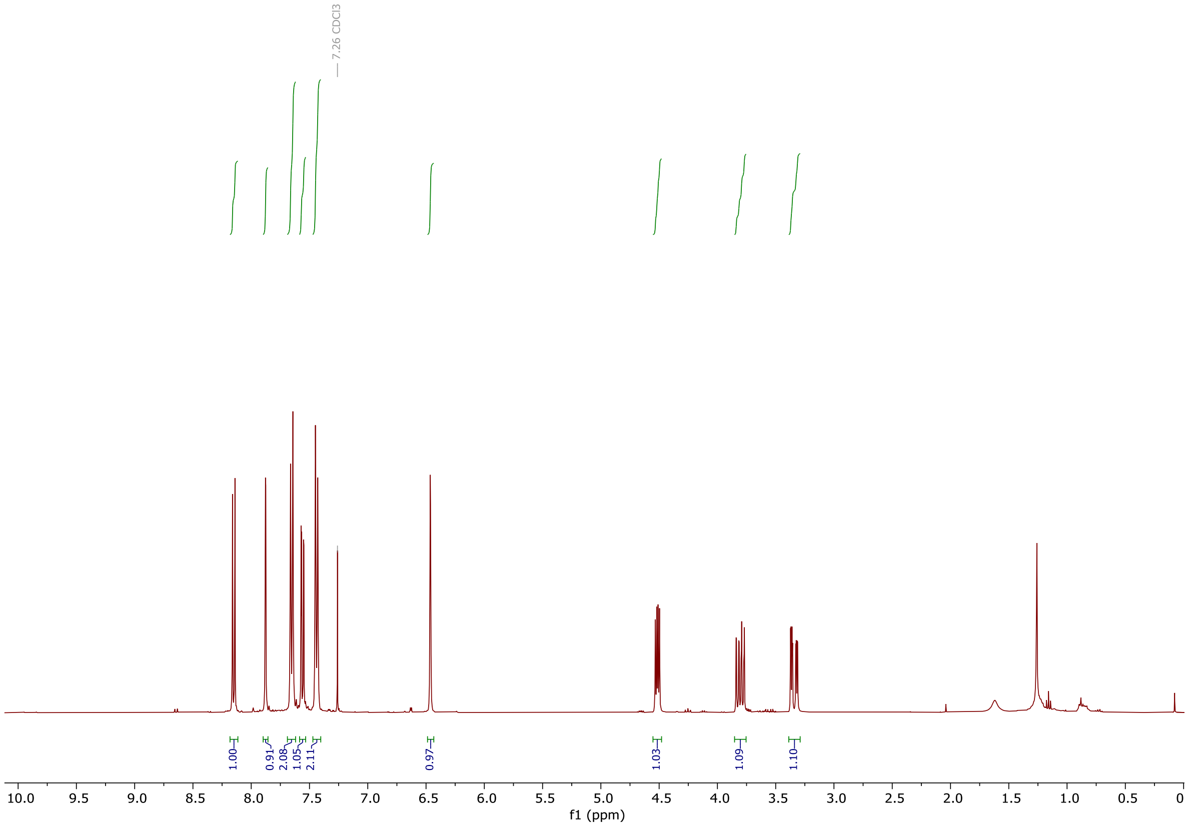

^{13}C-NMR: 3-oxo-2-(4-(trifluoromethyl)phenyl)-2,3-dihydro-1H-pyrrolo[1,2-a]indole-7-carbonitrile (**28**)

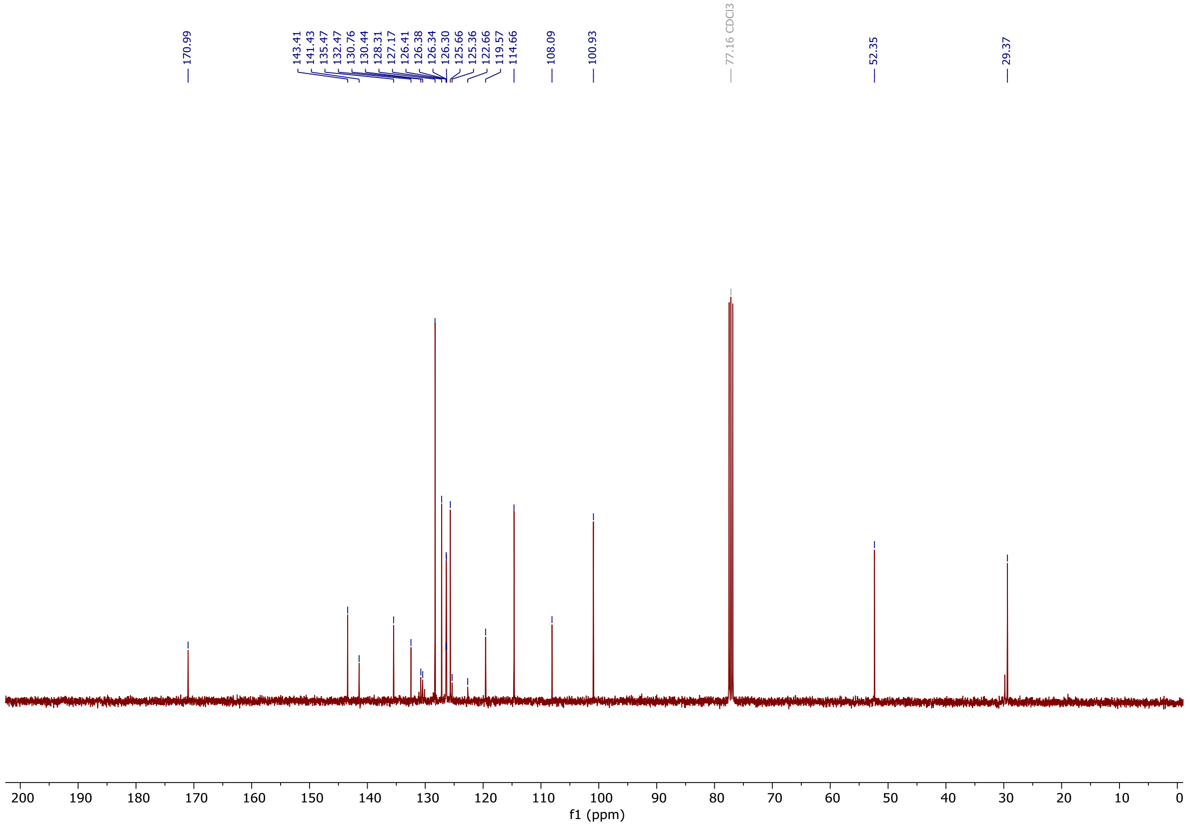

^{19}F-NMR: 3-oxo-2-(4-(trifluoromethyl)phenyl)-2,3-dihydro-1H-pyrrolo[1,2-a]indole-7-carbonitrile (**28**)

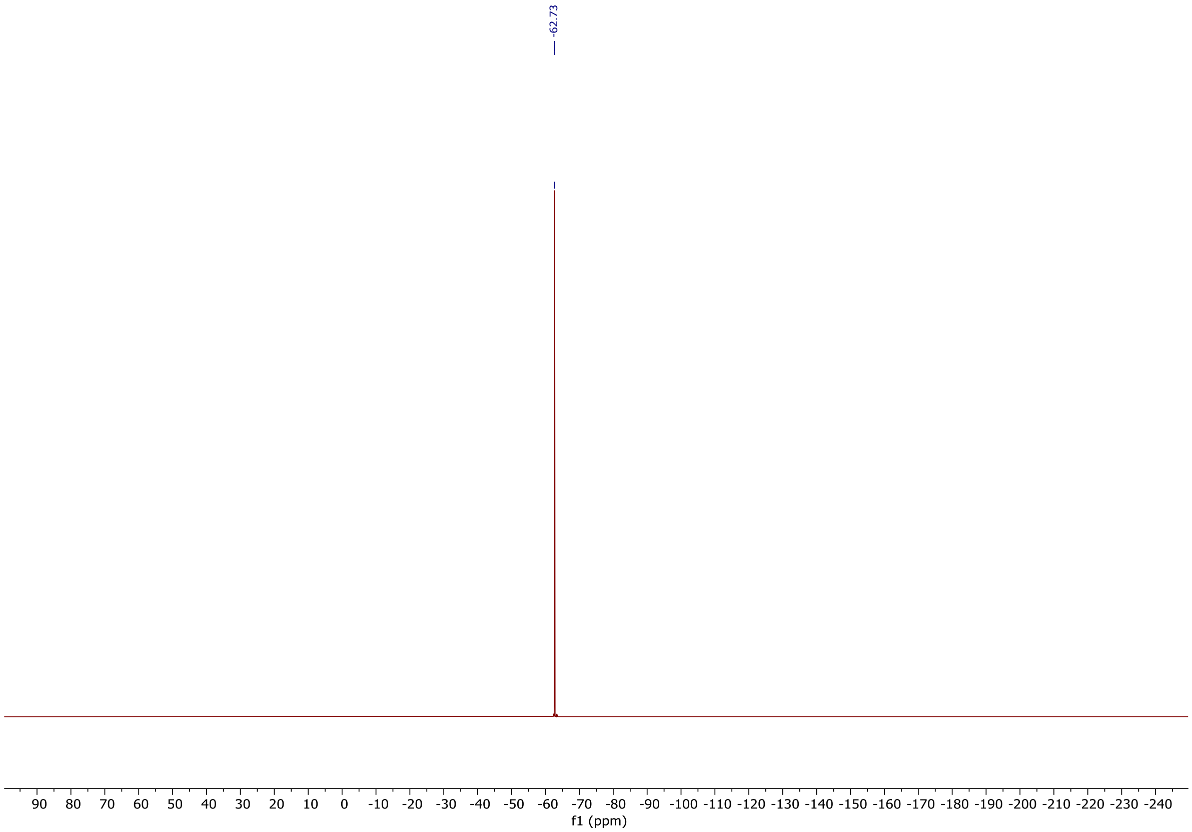

^{1}H-NMR: (E/Z)-2-phenylbut-2-enoic acid (**S28**)

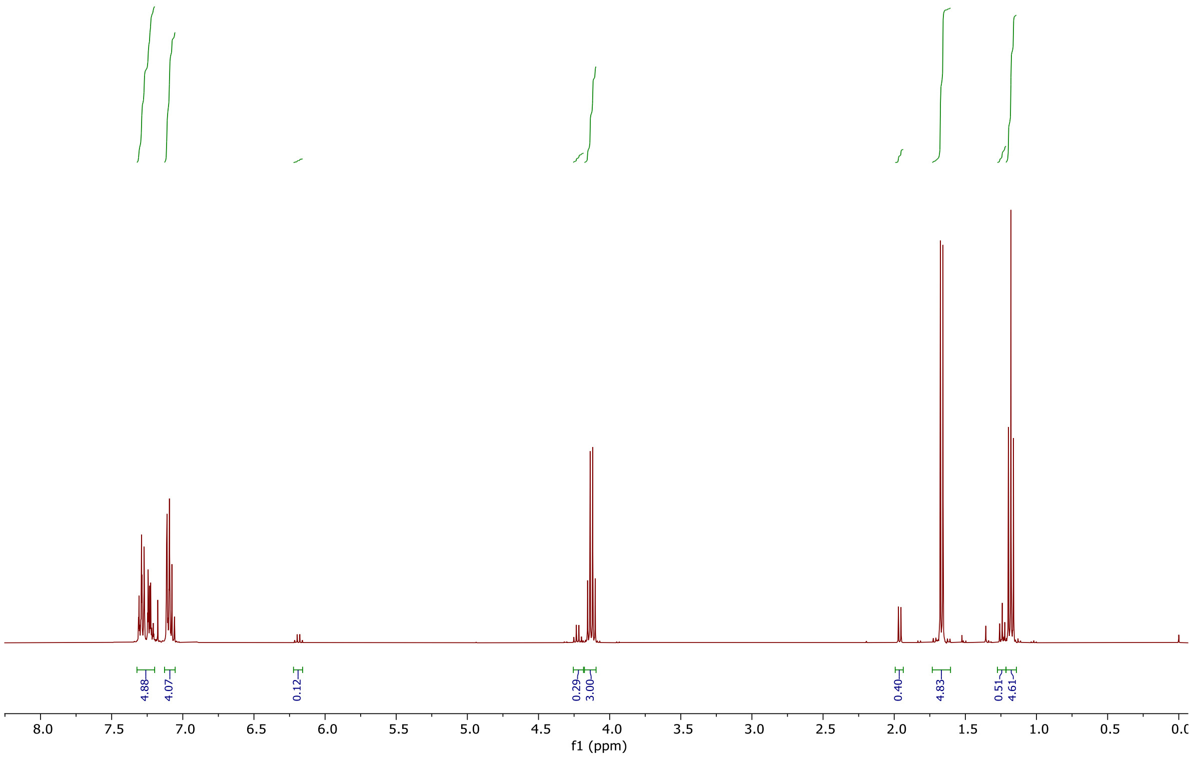

^{13}C-NMR: (E/Z)-2-phenylbut-2-enoic acid (**S28**)

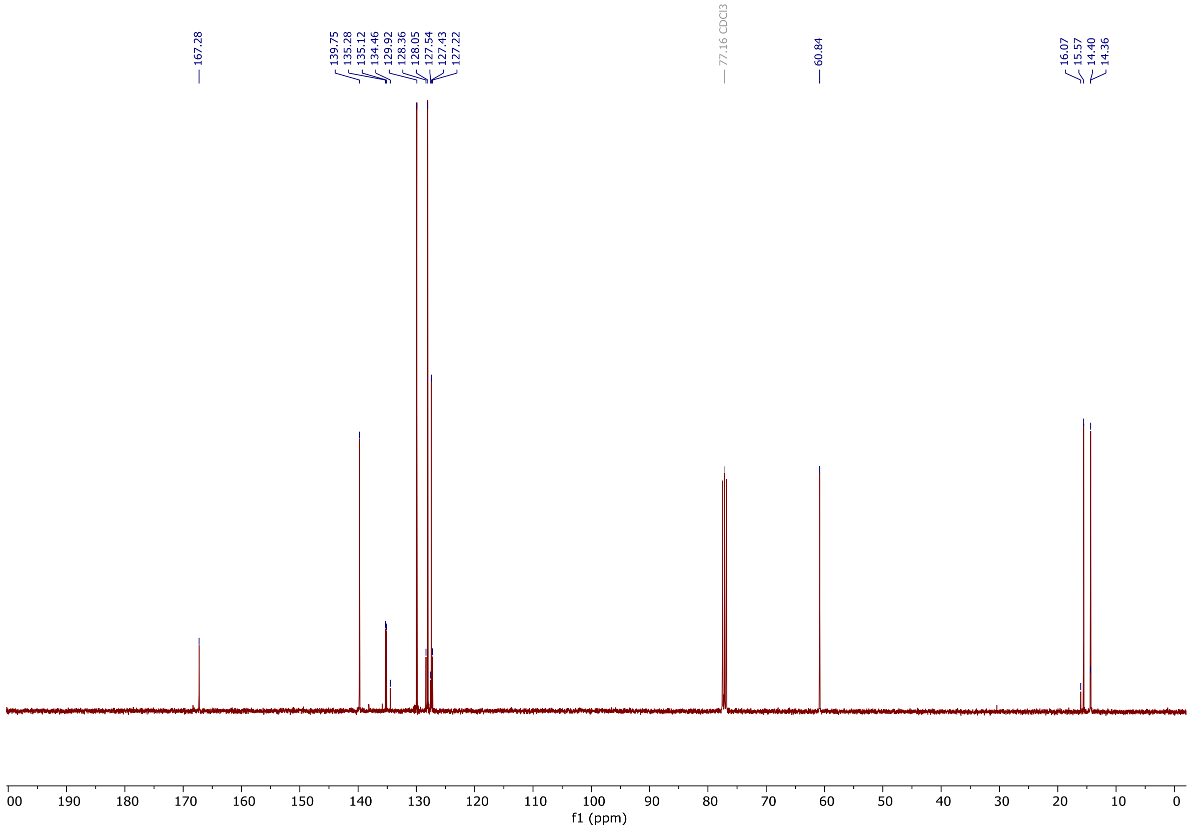

¹H-NMR: (E/Z)-2-phenylbut-2-enoic acid (**S29**)

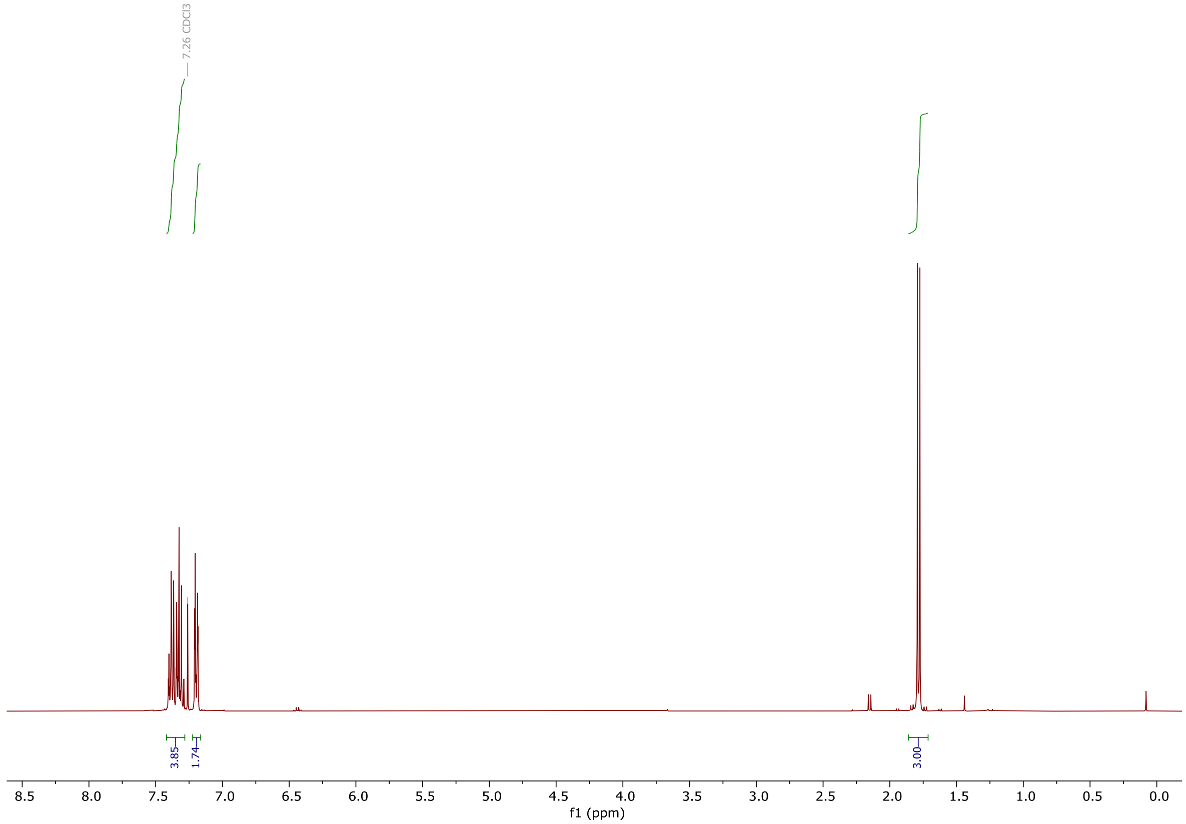

¹³C-NMR: (E/Z)-2-phenylbut-2-enoic acid (**S29**)

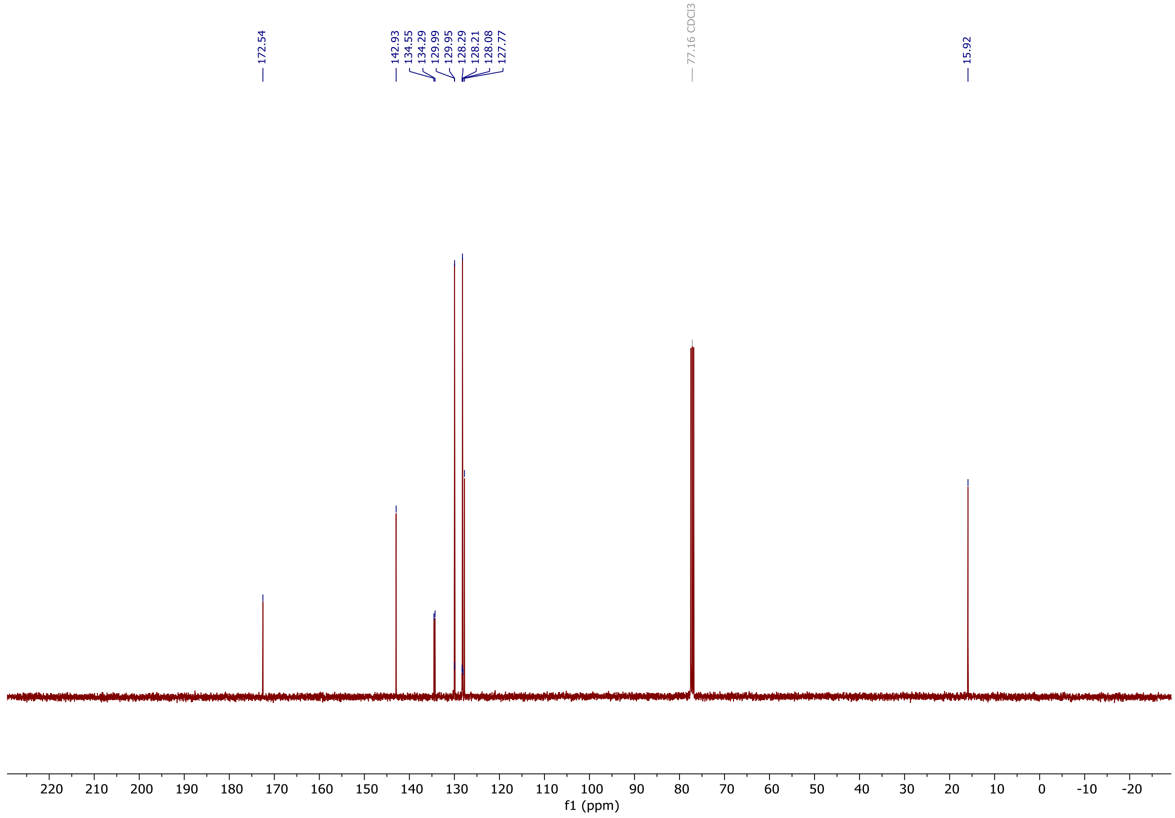

^{1}H-NMR: (E/Z)-2-(1-(2-phenylbut-2-enoyl)-1H-indol-3-yl)acetonitrile (**S30**)

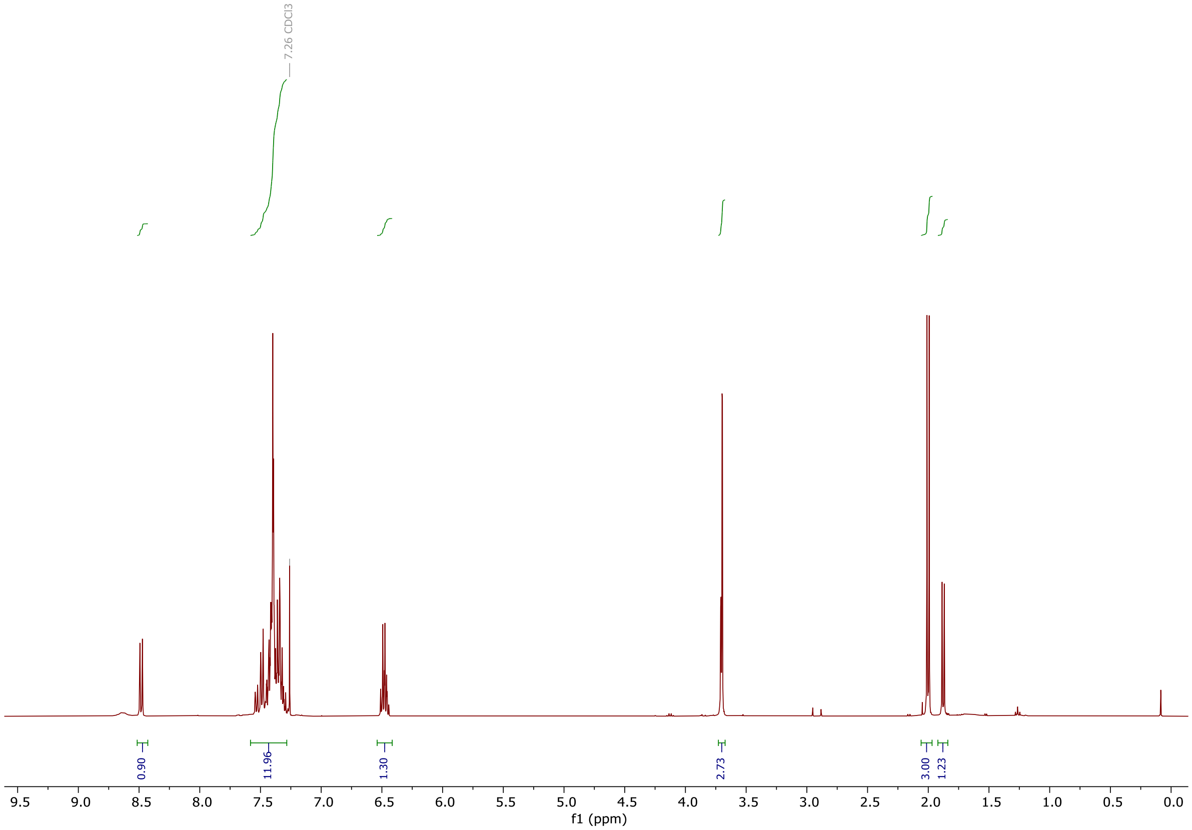

^{13}C-NMR: (E/Z)-2-(1-(2-phenylbut-2-enoyl)-1H-indol-3-yl)acetonitrile (**S30**)

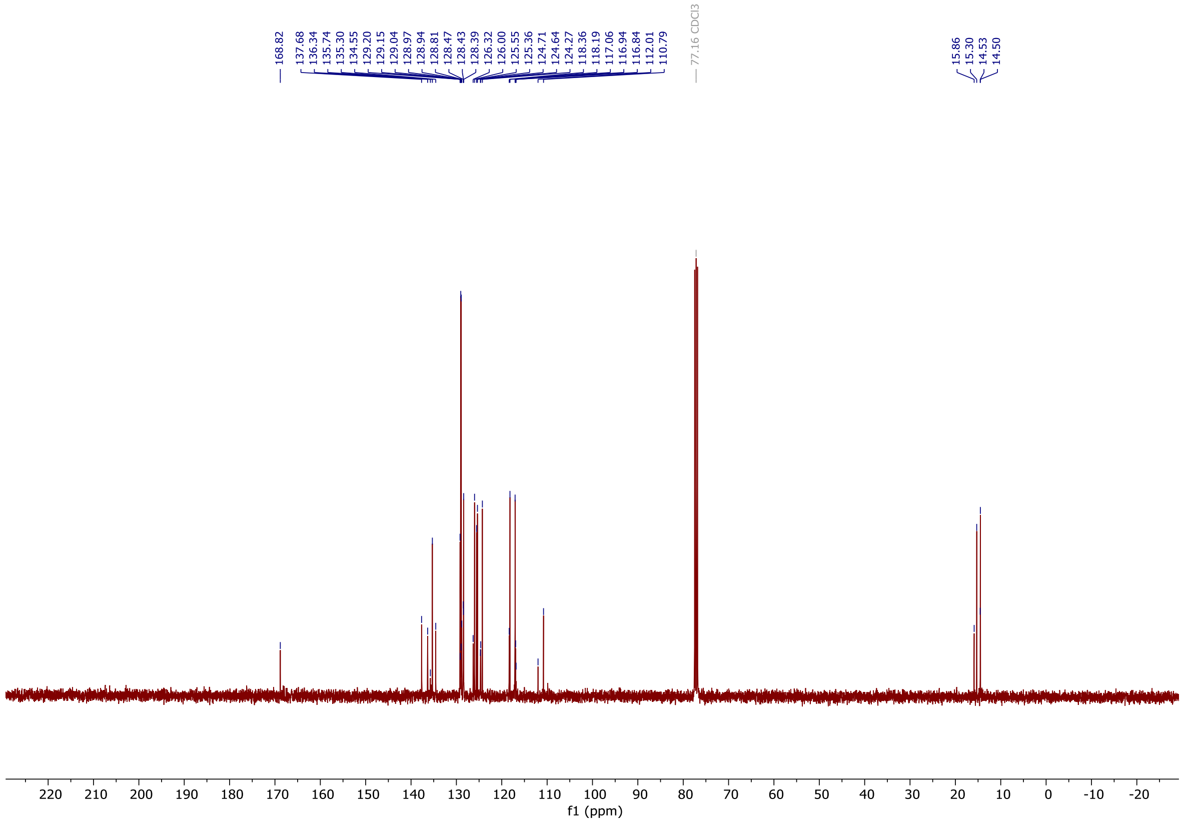

^{1}H-NMR: 2-(1-methyl-3-oxo-2-phenyl-2,3-dihydro-1H-pyrrolo[1,2-a]indol-9-yl)acetonitrile (**29**)

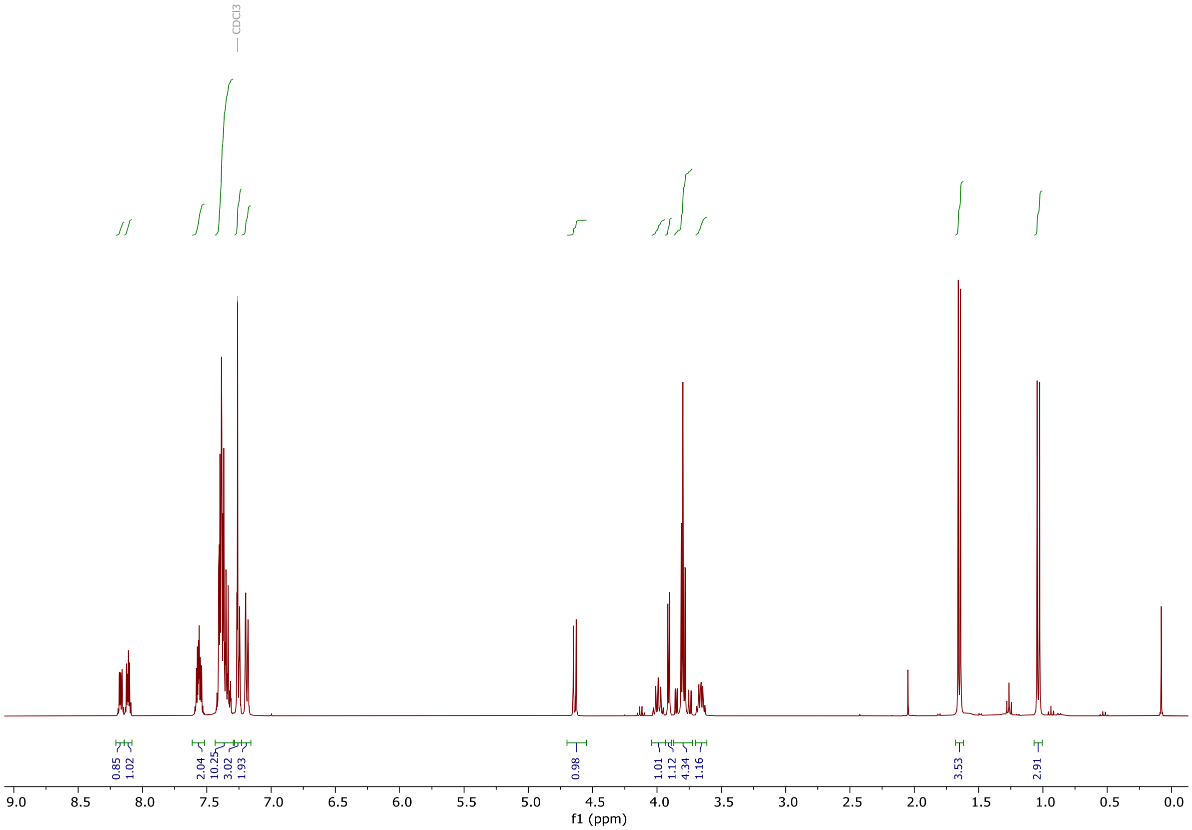

^{13}C-NMR: 2-(1-methyl-3-oxo-2-phenyl-2,3-dihydro-1H-pyrrolo[1,2-a]indol-9-yl)acetonitrile (**29**)

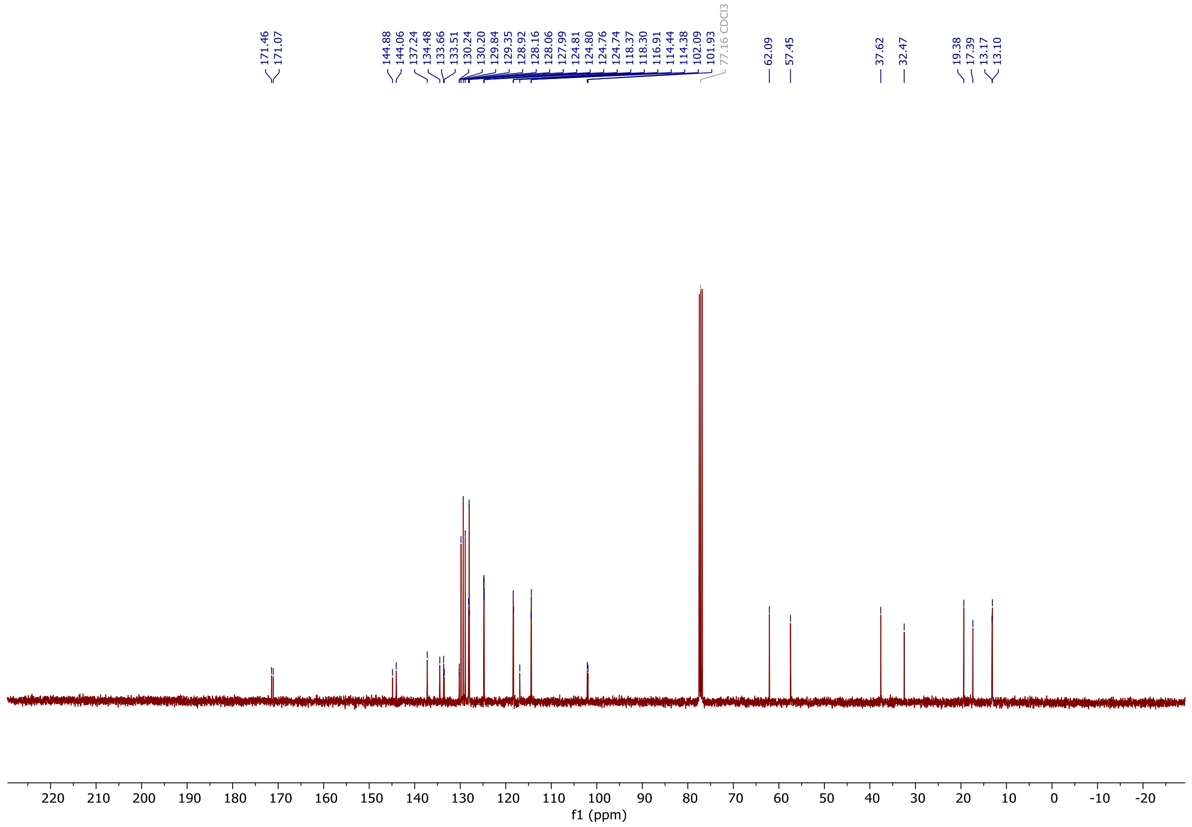

¹H-NMR: 2-phenyl-1-(3-(thiazol-2-yl)-1H-indol-1-yl)prop-2-en-1-one (**S31**)

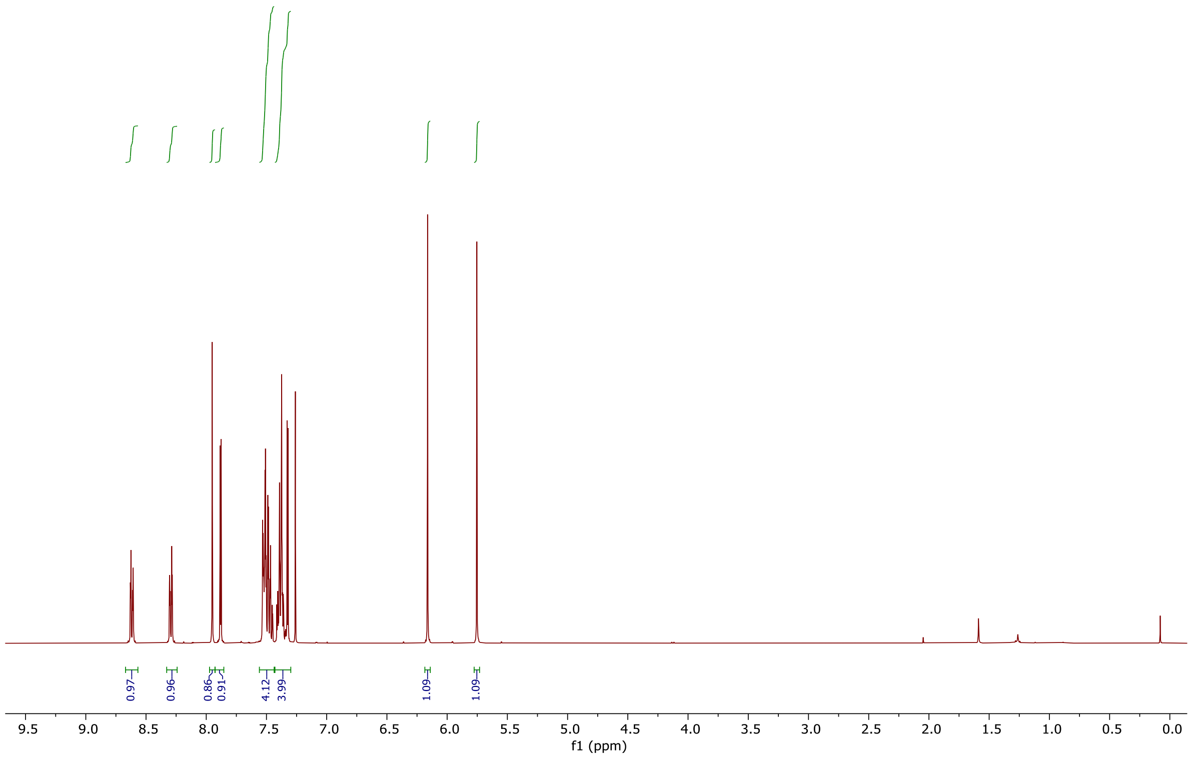

¹³C-NMR: 2-phenyl-1-(3-(thiazol-2-yl)-1H-indol-1-yl)prop-2-en-1-one (**S31**)

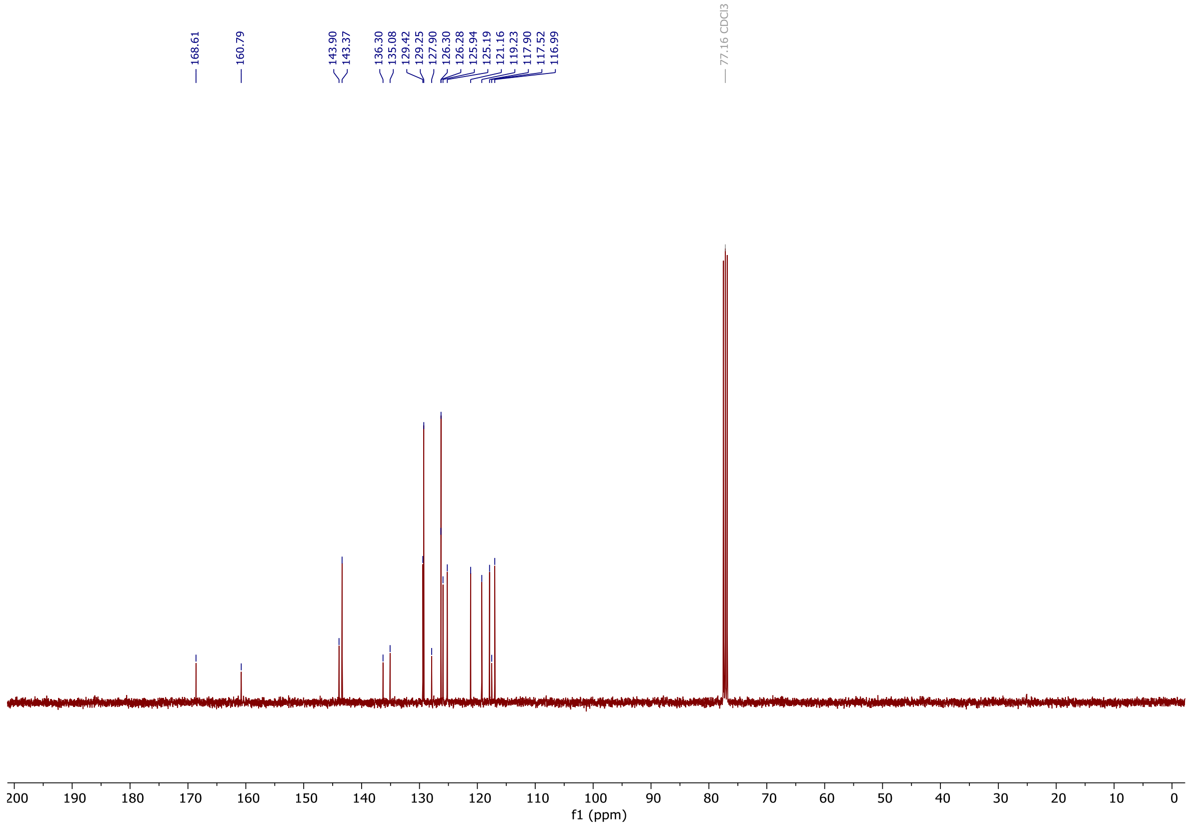

^{1}H-NMR: 2-phenyl-9-(thiazol-2-yl)-1,2-dihydro-3H-pyrrolo[1,2-a]indol-3-one (**30**)

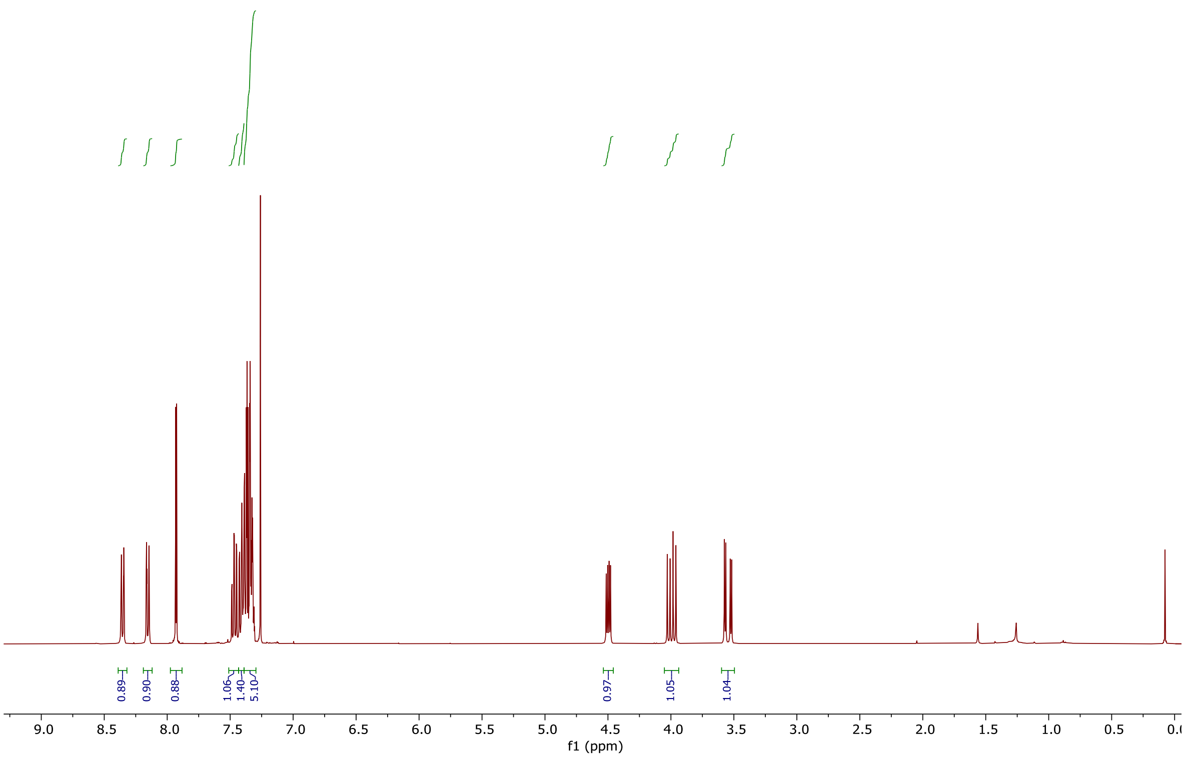

^{13}C-NMR: 2-phenyl-9-(thiazol-2-yl)-1,2-dihydro-3H-pyrrolo[1,2-a]indol-3-one (**30**)

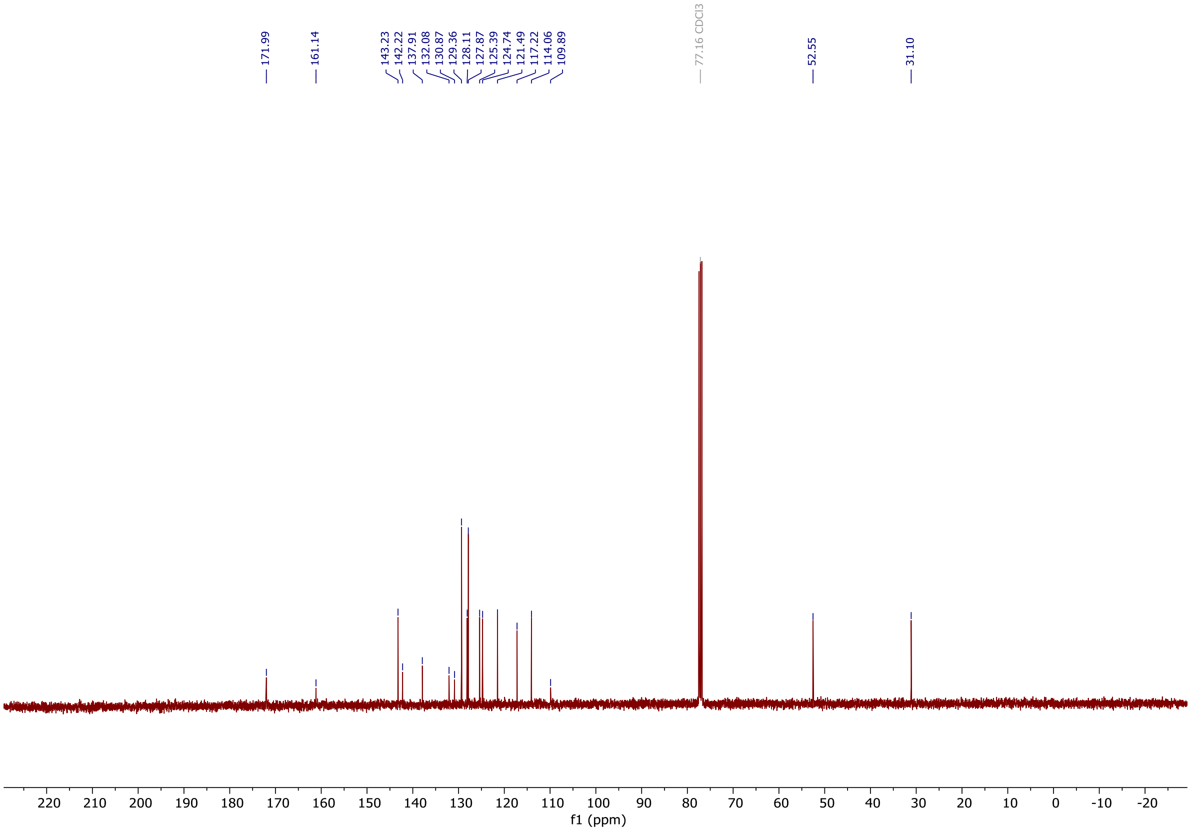

^{1}H-NMR: N-(2-(5-methoxy-1-(2-phenylacryloyl)-1H-indol-3-yl)ethyl)acetamide (**S32**)

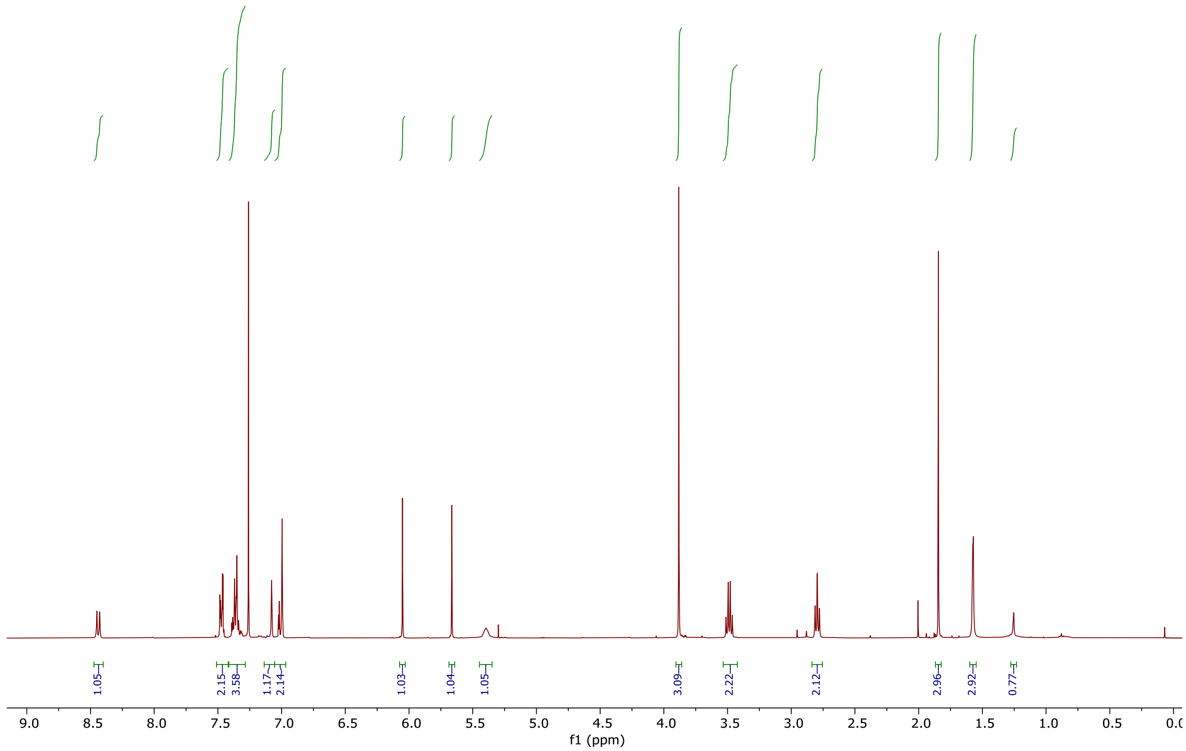

^{13}C-NMR: N-(2-(5-methoxy-1-(2-phenylacryloyl)-1H-indol-3-yl)ethyl)acetamide (**S32**)

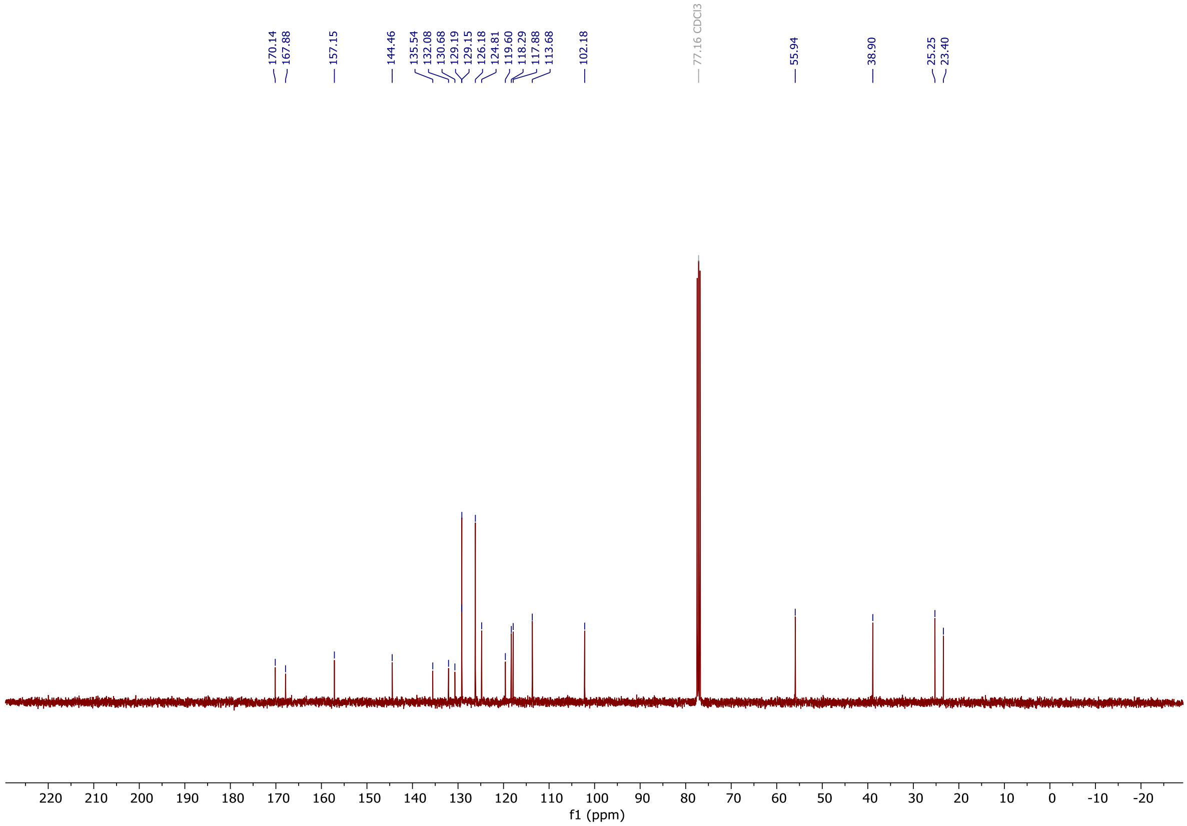

[1]**H-NMR**: N-(2-(7-methoxy-3-oxo-2-phenyl-2,3-dihydro-1H-pyrrolo[1,2-a]indol-9-yl)ethyl)acetamide (**31**)

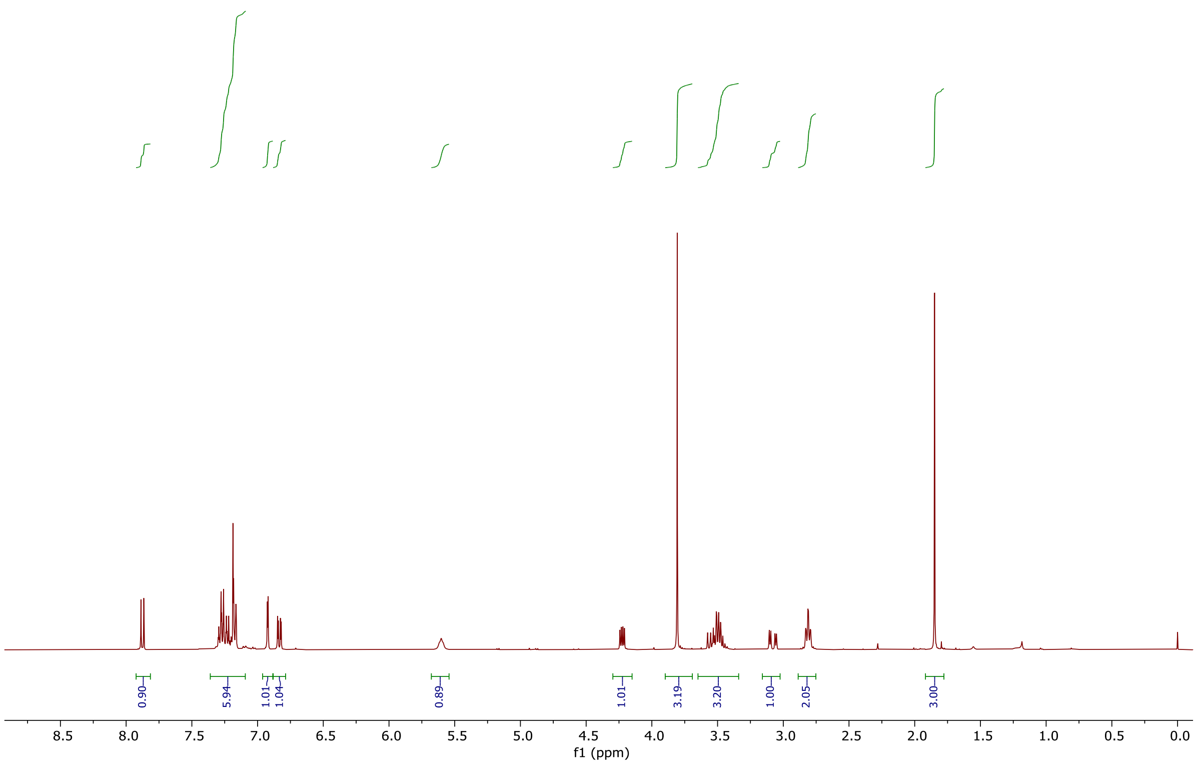

[13]**C-NMR**: N-(2-(7-methoxy-3-oxo-2-phenyl-2,3-dihydro-1H-pyrrolo[1,2-a]indol-9-yl)ethyl)acetamide (**31**)

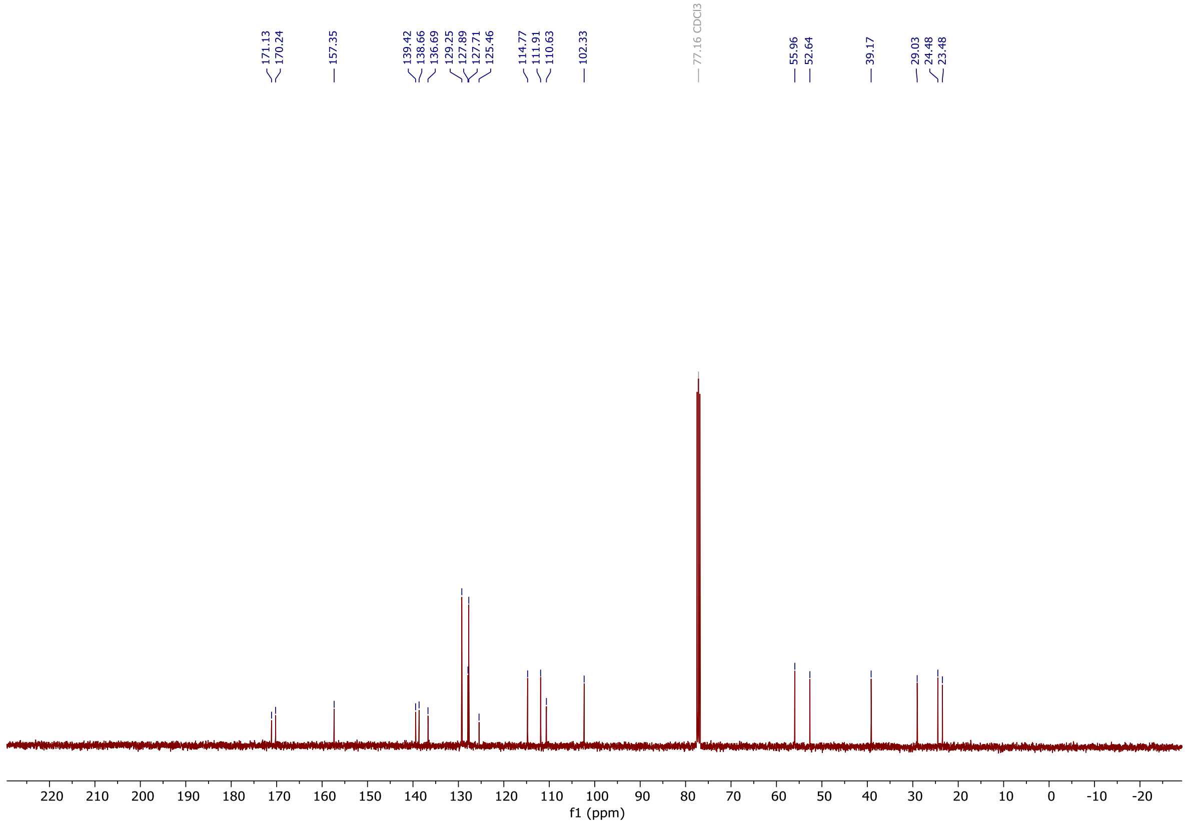

^{1}H-NMR: 1-(1H-imidazol-1-yl)-2-phenylprop-2-en-1-one (**S33**)

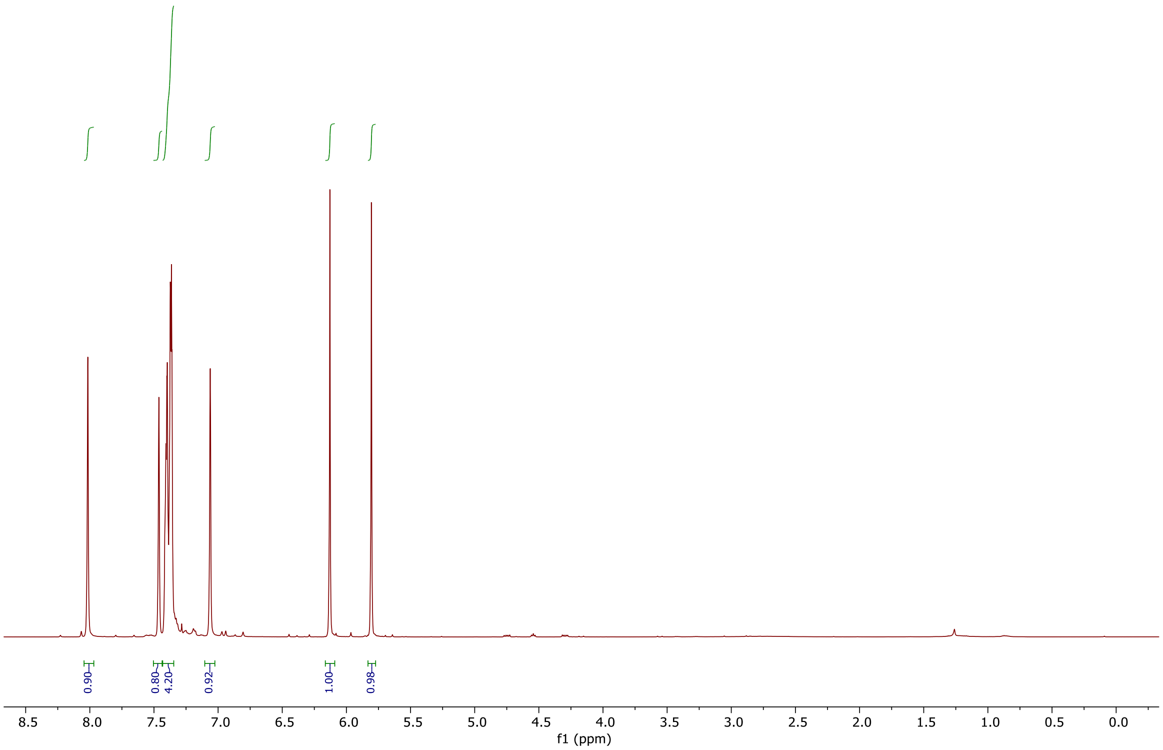

^{13}C-NMR: 1-(1H-imidazol-1-yl)-2-phenylprop-2-en-1-one (**S33**)

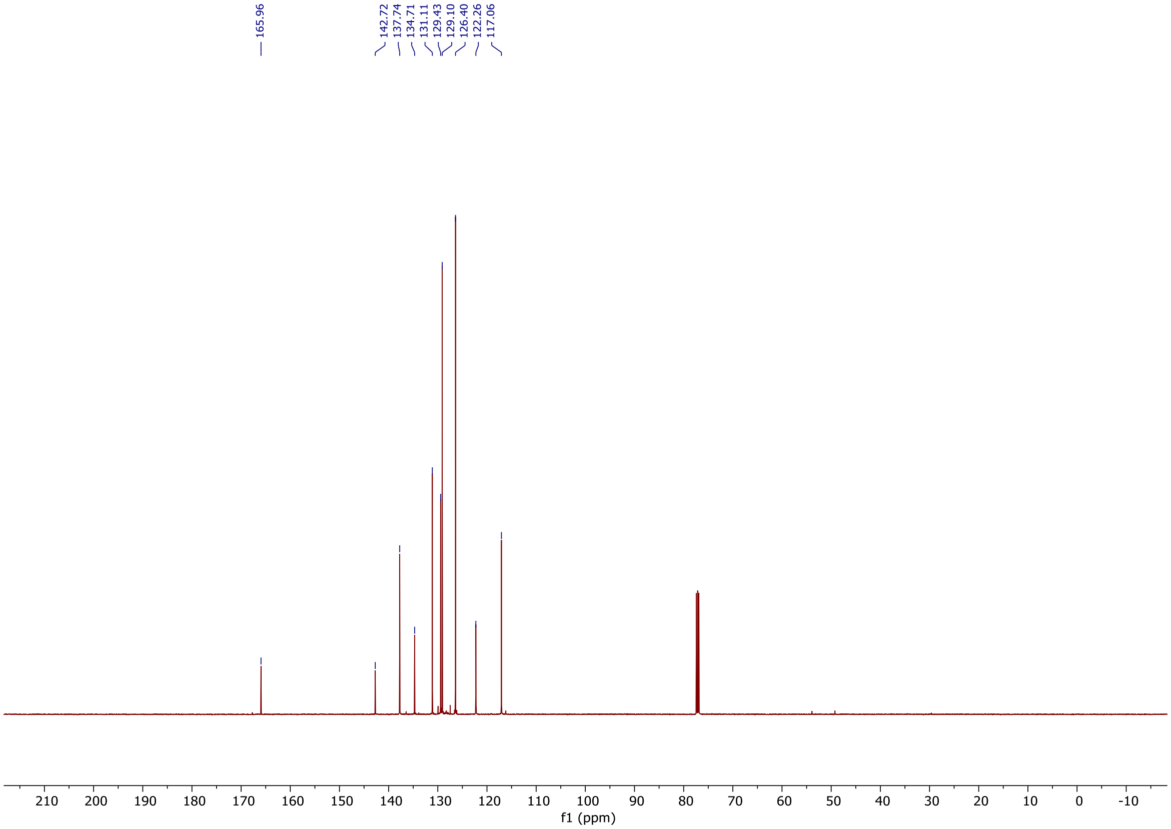

^{1}H-NMR: 1-acryloyl-1H-indole-5-carbonitrile (**S34**)

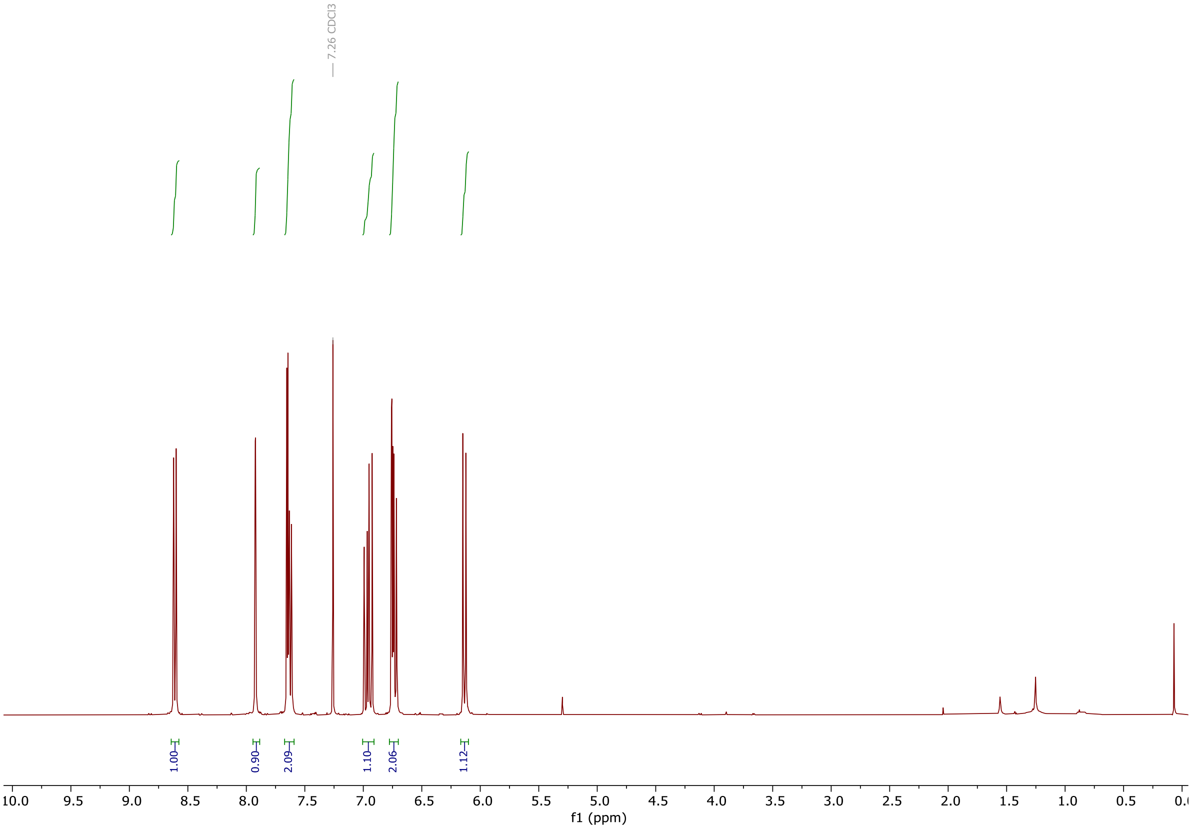

^{13}C-NMR: 1-acryloyl-1H-indole-5-carbonitrile (**S34**)

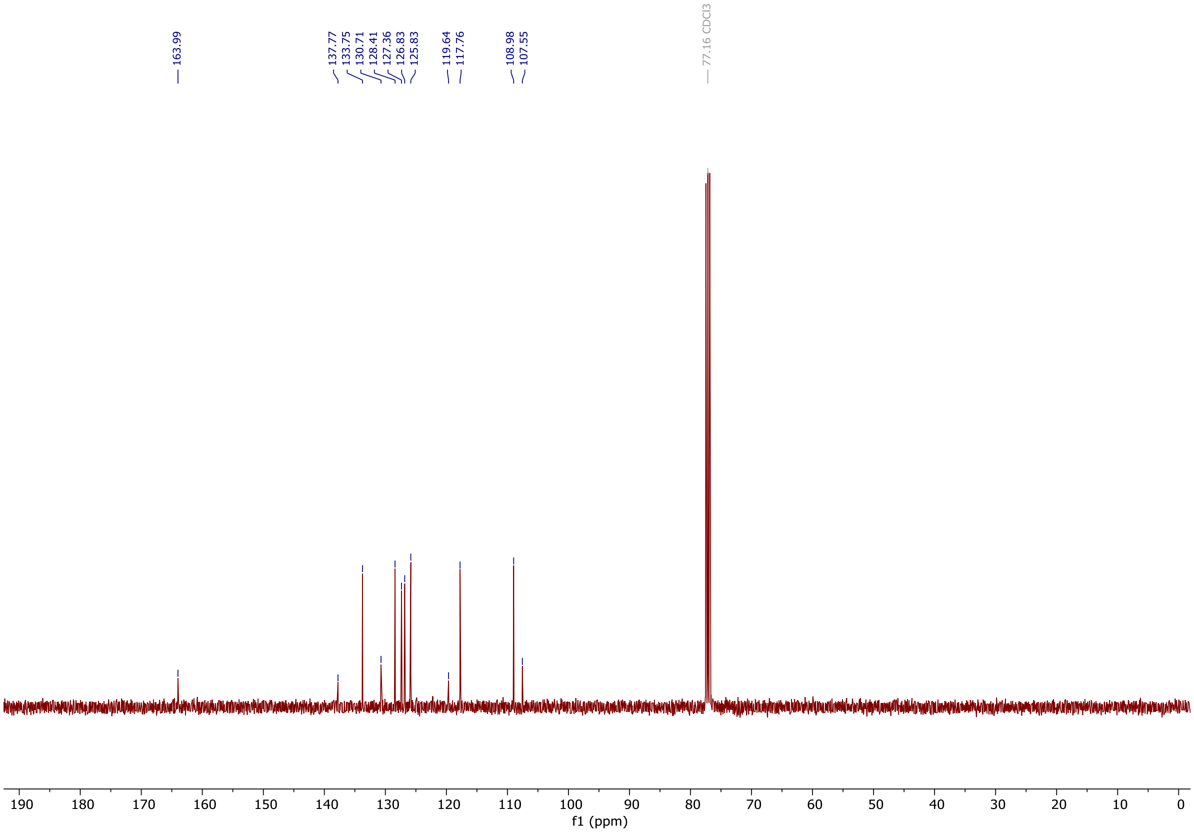

^{1}H-NMR: 1-methacryloyl-1H-indole-5-carbonitrile (**S35**)

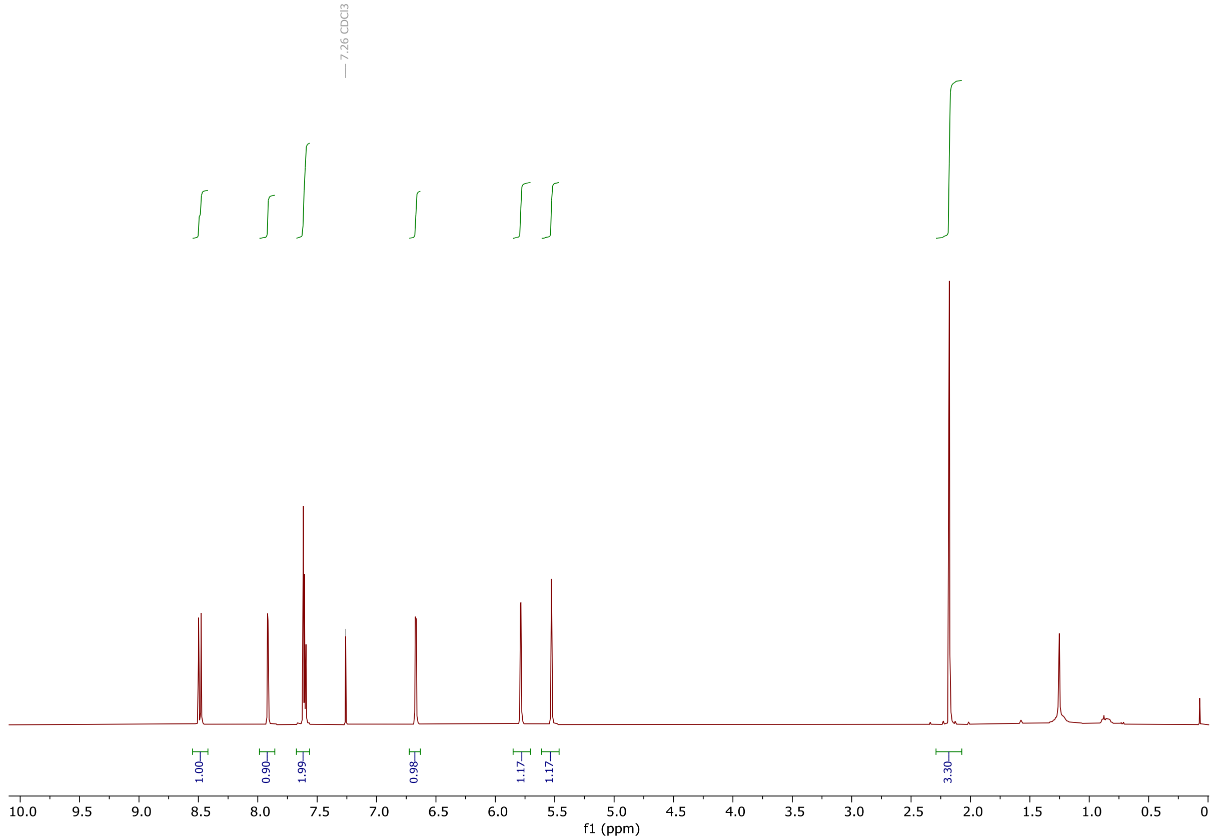

^{13}C-NMR: 1-methacryloyl-1H-indole-5-carbonitrile (**S35**)

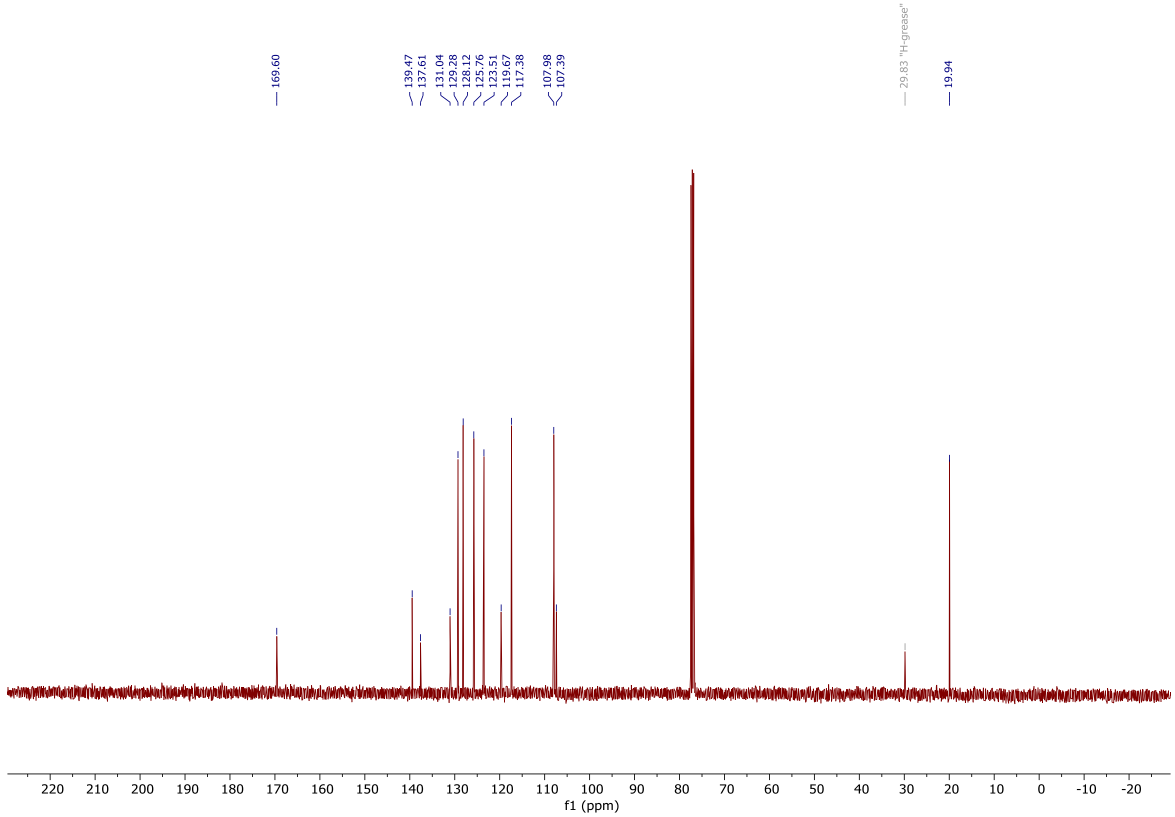

^{1}H-NMR: 1-((phenyl-2-d)sulfonyl)-1H-indole-2-d (**S38**)

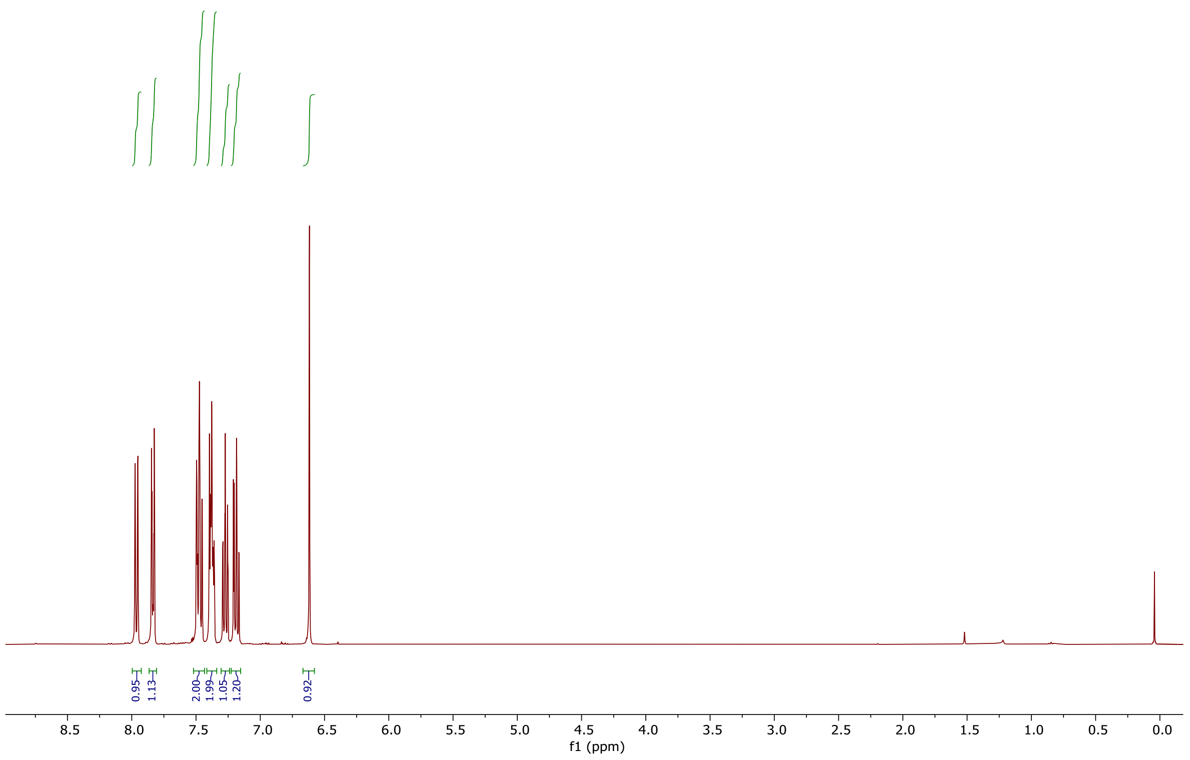

^{13}C-NMR: 1-((phenyl-2-d)sulfonyl)-1H-indole-2-d (**S38**)

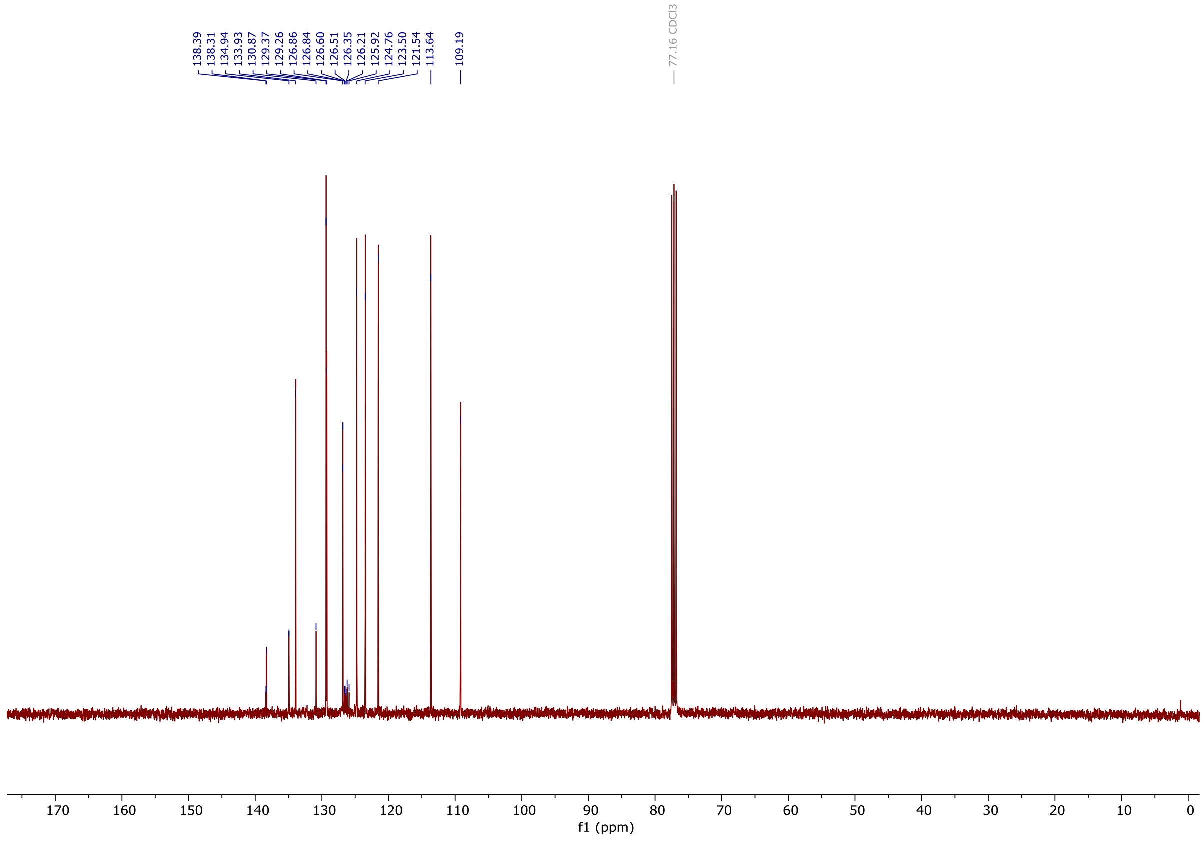

^{1}H-NMR: 1H-indole-2-d (**S39**)

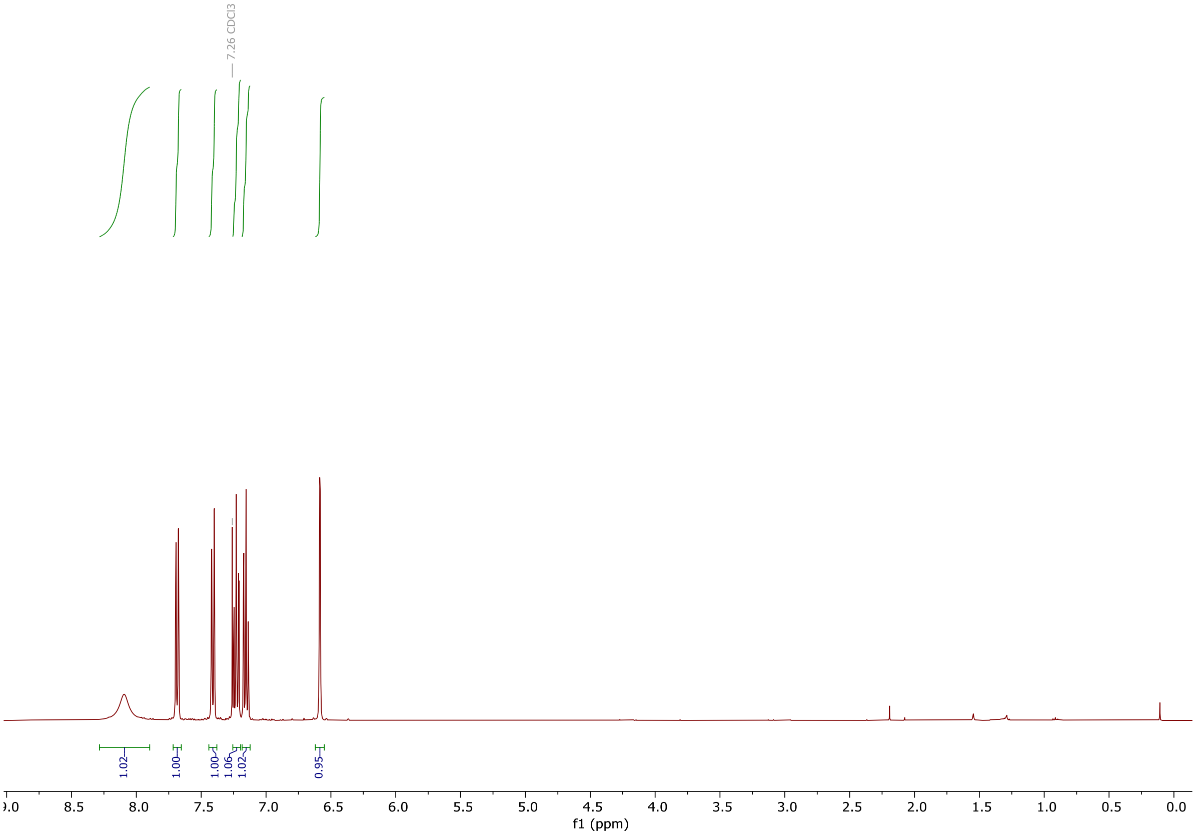

^{13}C-NMR: 1H-indole-2-d (**S39**)

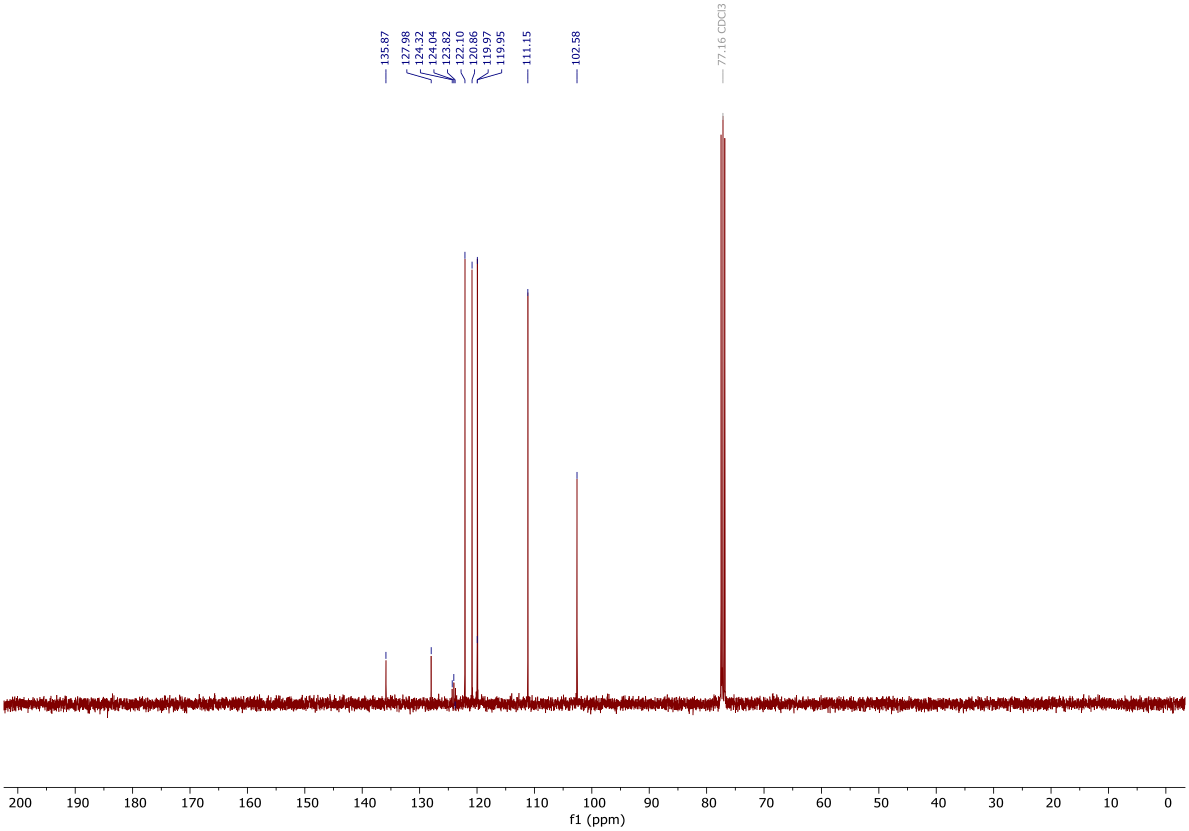

^{1}H-NMR: 1-(1H-indol-1-yl-2-d)-2-phenylprop-2-en-1-one (**33**)

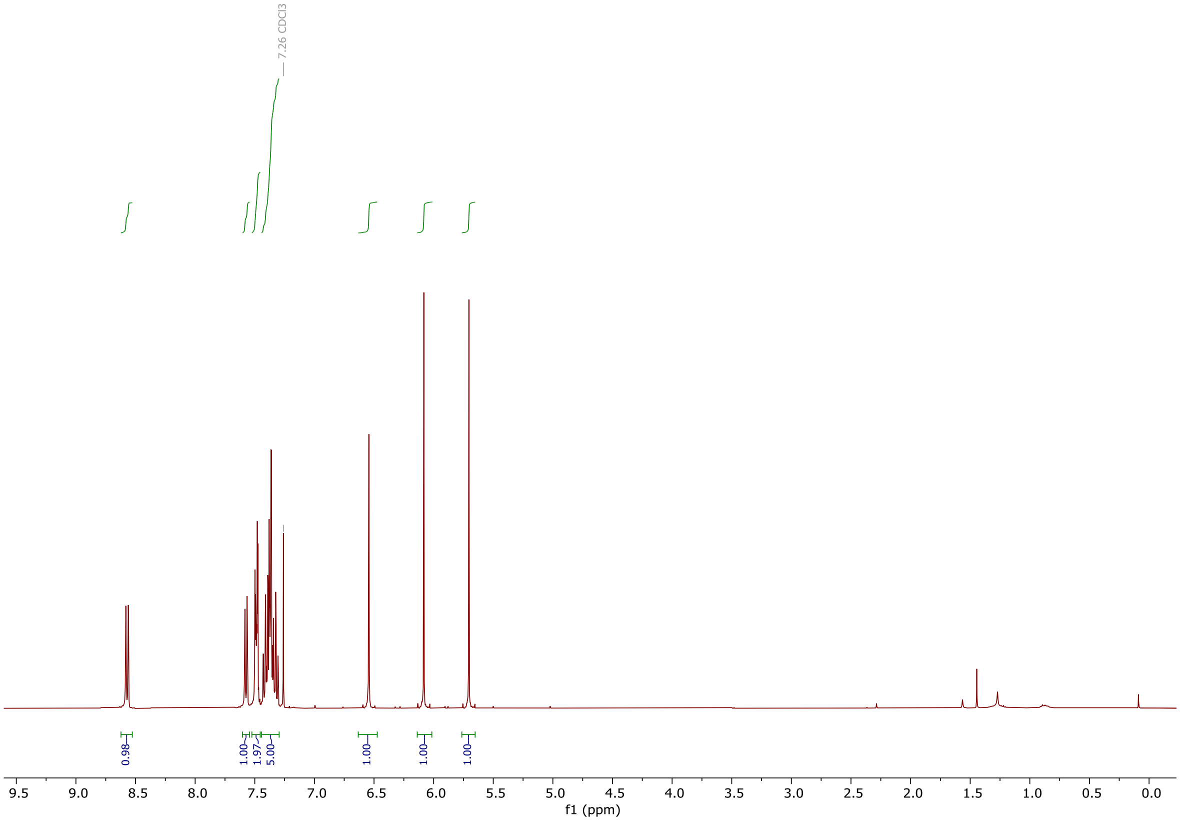

^{13}C-NMR: 1-(1H-indol-1-yl-2-d)-2-phenylprop-2-en-1-one (**33**)

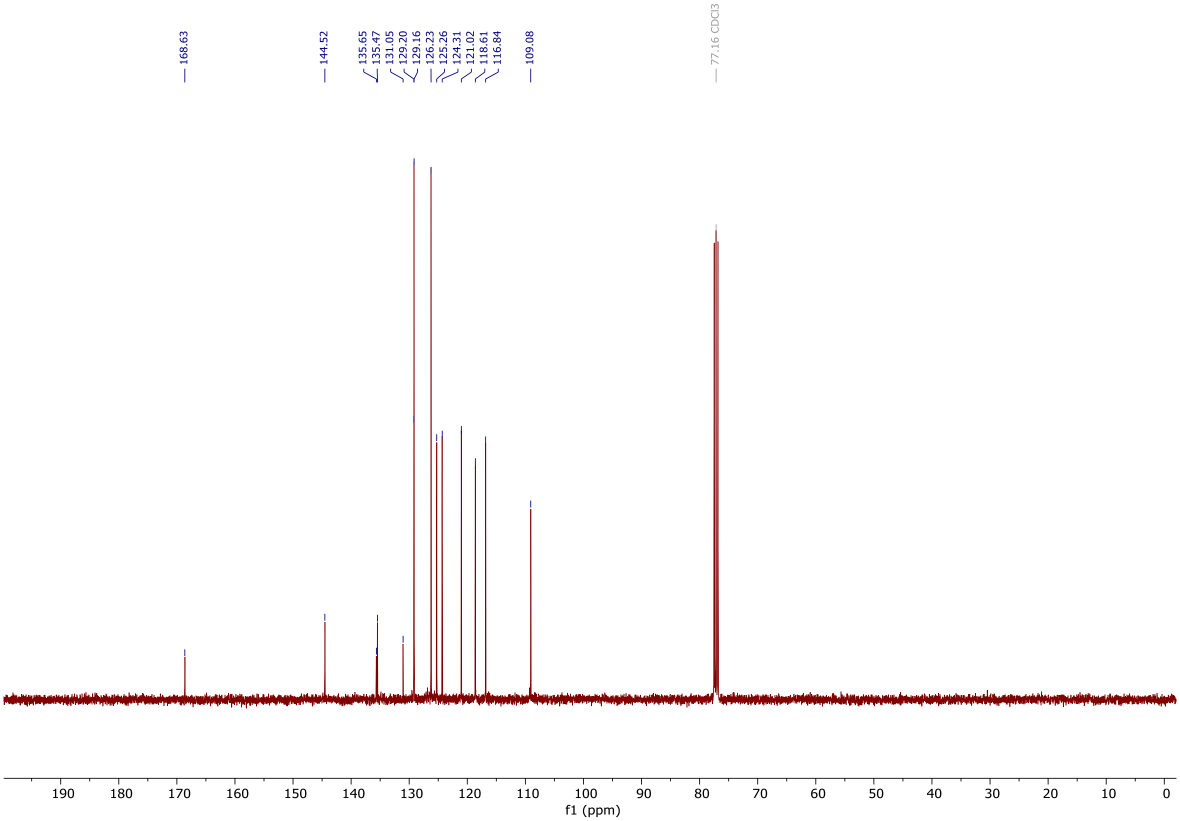

^{1}H-NMR: 2-phenyl-1,2-dihydro-3H-pyrrolo[1,2-a]indol-3-one-2,9-d2 (**34**)

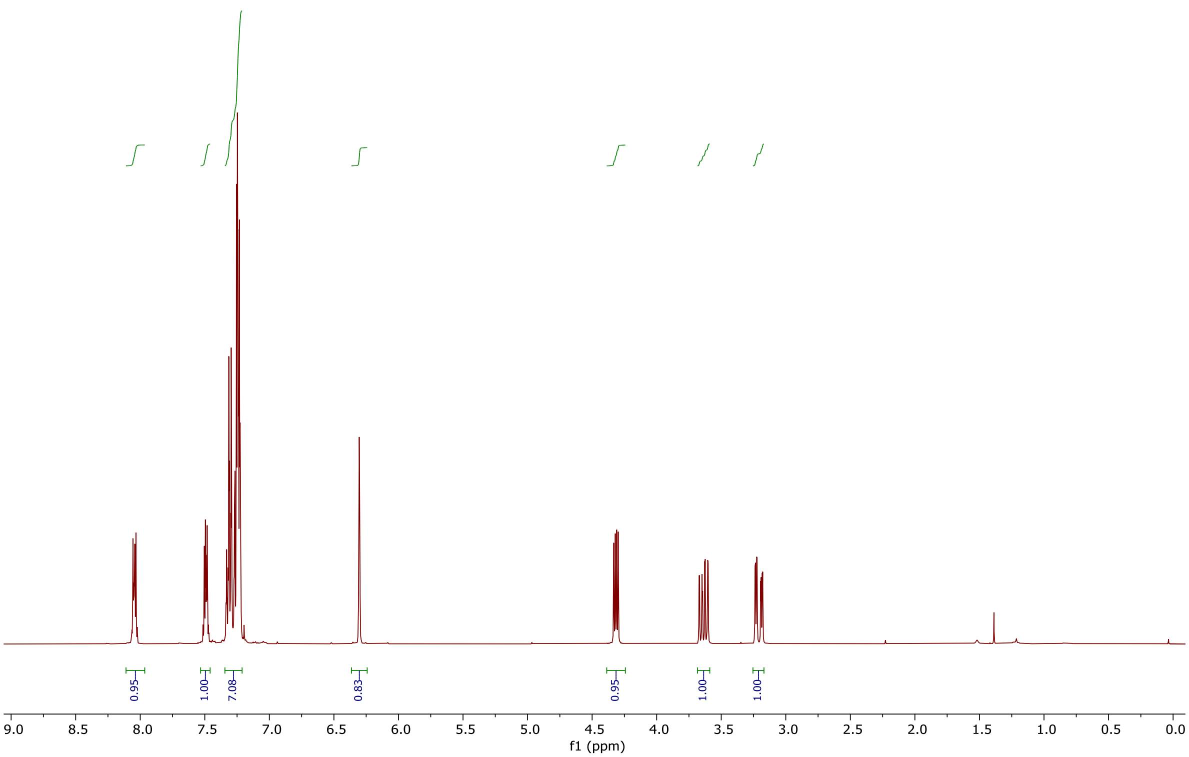

^{13}C-NMR: 2-phenyl-1,2-dihydro-3H-pyrrolo[1,2-a]indol-3-one-2,9-d2 (**34**)

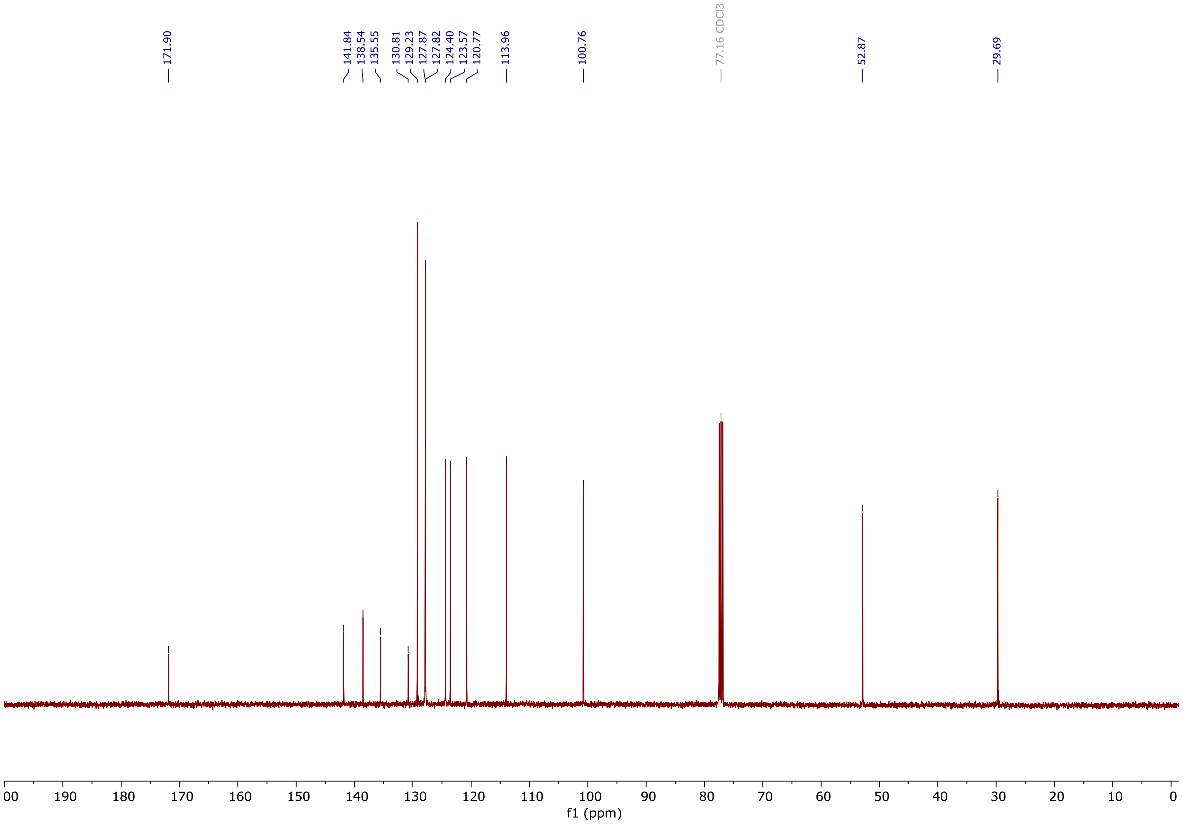

^{1}H-NMR: 7-isocyano-2-phenyl-1,2-dihydro-3H-pyrrolo[1,2-a]indol-3-one-2-d (**35**)

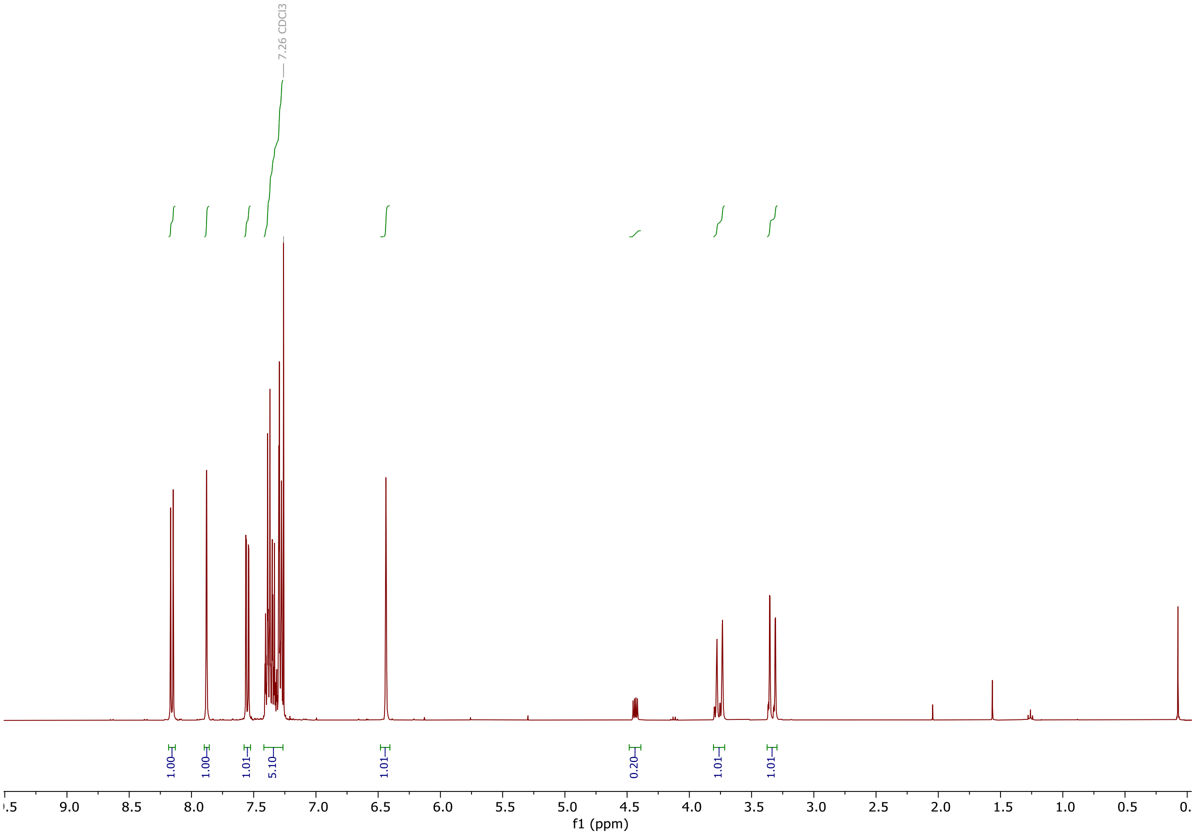

^{13}C-NMR: 7-isocyano-2-phenyl-1,2-dihydro-3H-pyrrolo[1,2-a]indol-3-one-2-d (**35**)

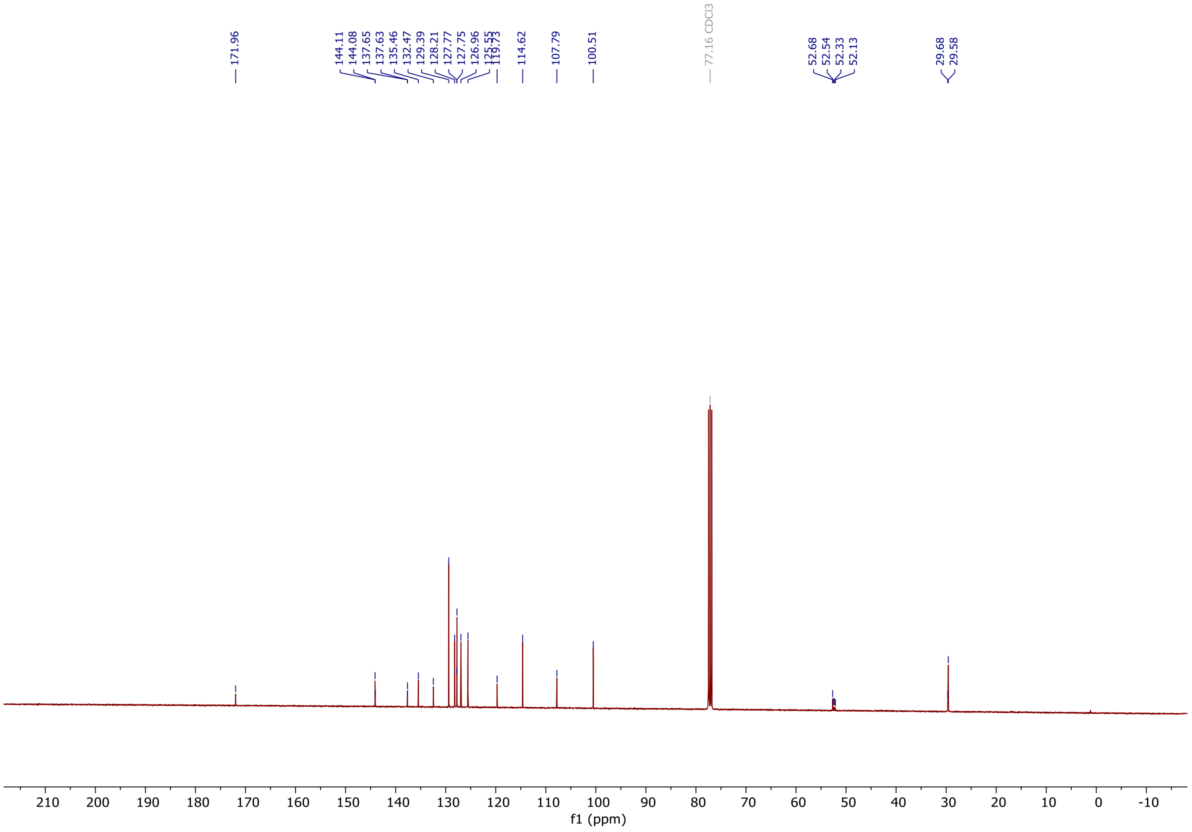

^{1}H-NMR: 2-(2-hydroxypropan-2-yl)-3-oxo-2-phenyl-2,3-dihydro-1H-pyrrolo[1,2-a]indole-7-carbonitrile (**32**)

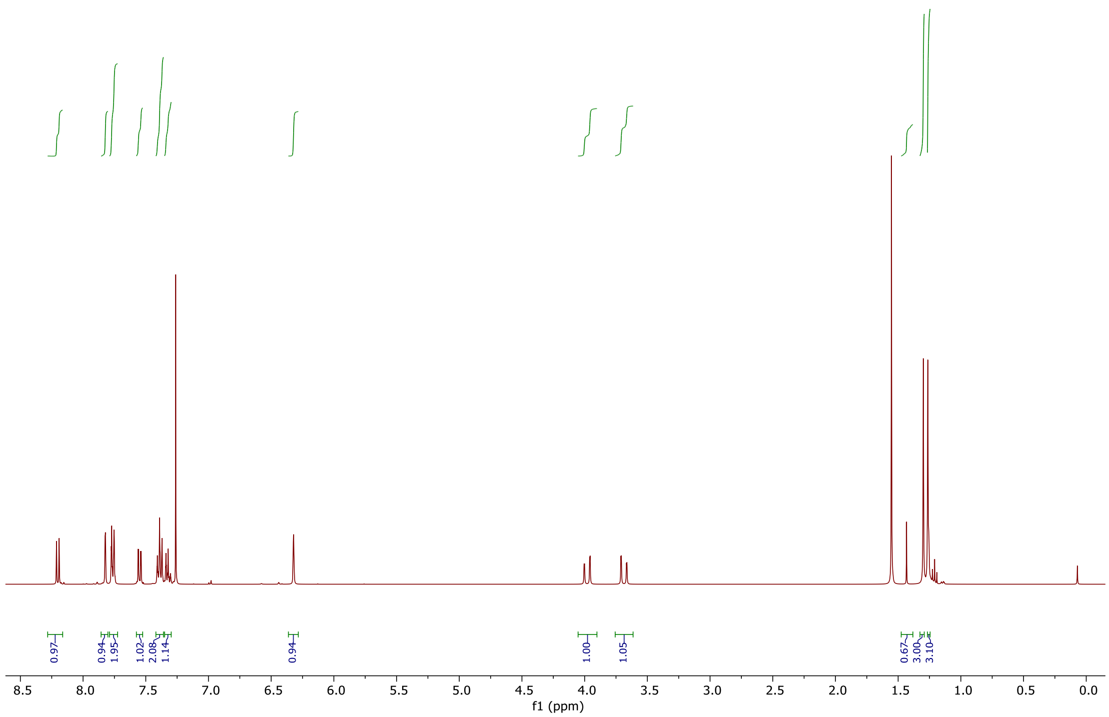

^{13}C-NMR: 2-(2-hydroxypropan-2-yl)-3-oxo-2-phenyl-2,3-dihydro-1H-pyrrolo[1,2-a]indole-7-carbonitrile (**32**)

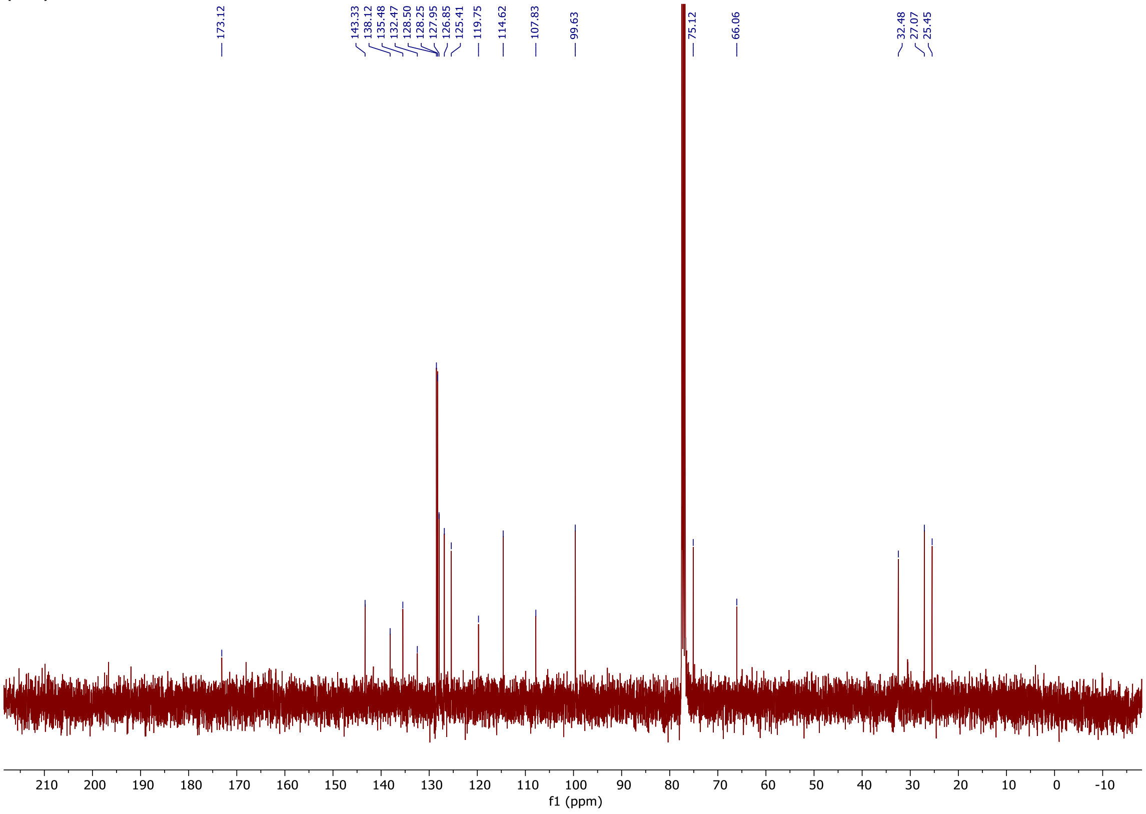

11. Computational Studies

Computational details

Methods

The range-separated dispersion-corrected M06-2X density functional and the 6-31++G(d,p) basis set were used with Grimme's D3 correction using the original D3 damping function to optimize the geometries of all stationary points. Previous studies have demonstrated that the combination of M06-2X with D3 dispersion is robust, reliable and accurate.[13] To these optimized structures, single-point energy corrections (M06-2X-D3/Def2-QZVPP) were applied. All the calculations included the integral equation formalism variant of the polarizable continuum model (IEF-PCM) with the SMD solvation model (solvent = ethylethanoate) to account for solvent effects. *Gaussian 16*[14] was employed for all density functional theory (DFT) calculations, using an "ultrafine" pruned (99,590) grid for numerical integration of the exchange-correlation functional and its derivatives. The wavefunction stability calculations of **INT-I-S_0** were carried out with the *stable* keyword in two different ways: (i) stable and (ii) guess=mix stable=opt, which lead to the same result ($\langle \hat{S}^2 \rangle$ = 0 as the most stable value).

Minimum energy crossing points (MECP) of the intersystem crossings (ISC) were calculated with MECPro, interfaced to *Gaussian 16*.[15] Molecular graphics were generated using *PyMol*;[16] Our display settings have been made openly accessible.[17]

The systems studied do not show complex conformational spaces and we were able to generate conformers manually. Multiple conformers were only found for **SM-S_0** and **SM-T_1**, and these conformers were labeled using different suffixes (i.e. **SM-S_0_a**, **SM-S_0_b**, **SM-S_0_c**, etc). The nomenclature used has been changed in the main article for simplicity and the current nomenclature can be found in Figure S5.

We performed vibrational frequency calculations to verify that stationary points were either minima or first-order saddle points on the potential energy surface, and to calculate thermal corrections to Gibbs free energies (G). Moreover, intrinsic reaction coordinate (IRC) calculations[18] were performed to ensure

---

[13] Goerigk, L.; Hansen, A.; Bauer, C.; Ehrlich, S.; Najibi, A.; Grimme, S. A look at the density functional theory zoo with the advanced GMTKN55 database for general main group thermochemistry, kinetics and noncovalent interactions. *PCCP* **2017**, *19*, 32184–32215.

[14] Gaussian 16, Revision C.01, Frisch, M. J.; Trucks, G. W.; Schlegel, H. B.; Scuseria, G. E.; Robb, M. A.; Cheeseman, J. R.; Scalmani, G.; Barone, V.; Petersson, G. A.; Nakatsuji, H.; Li, X.; Caricato, M.; Marenich, A. V.; Bloino, J.; Janesko, B. G.; Gomperts, R.; Mennucci, B.; Hratchian, H. P.; Ortiz, J. V.; Izmaylov, A. F.; Sonnenberg, J. L.; Williams-Young, D.; Ding, F.; Lipparini, F.; Egidi, F.; Goings, J.; Peng, B.; Petrone, A.; Henderson, T.; Ranasinghe, D.; Zakrzewski, V. G.; Gao, J.; Rega, N.; Zheng, G.; Liang, W.; Hada, M.; Ehara, M.; Toyota, K.; Fukuda, R.; Hasegawa, J.; Ishida, M.; Nakajima, T.; Honda, Y.; Kitao, O.; Nakai, H.; Vreven, T.; Throssell, K.; Montgomery, J. A., Jr.; Peralta, J. E.; Ogliaro, F.; Bearpark, M. J.; Heyd, J. J.; Brothers, E. N.; Kudin, K. N.; Staroverov, V. N.; Keith, T. A.; Kobayashi, R.; Normand, J.; Raghavachari, K.; Rendell, A. P.; Burant, J. C.; Iyengar, S. S.; Tomasi, J.; Cossi, M.; Millam, J. M.; Klene, M.; Adamo, C.; Cammi, R.; Ochterski, J. W.; Martin, R. L.; Morokuma, K.; Farkas, O.; Foresman, J. B.; Fox, D. J. Gaussian, Inc., Wallingford CT, 2016.

[15] MECPro Version 1.0.5: Minimum Energy Crossing Program, Snyder, J. D.; Hamill, L.-A.; Faleumu, K. E.; Ess, D. H. Brigham Young University, 2019.

[16] The PyMOL Molecular Graphics System, version 2.0.7, Schrödinger, LLC.

[17] https://gist.github.com/bobbypaton (accessed 13 April 2018).

[18] Fukui, K. The Path of Chemical Reactions - the IRC Approach. *Acc. Chem. Res.* **1981**, *14*, 363–368

that the transition structures connected to their corresponding starting and final geometries (see section *IRC calculations*). The computed thermochemistry data were corrected following Grimme's quasi-harmonic (QHA) model for entropy[19] with a frequency cut-off value of 100.0 cm^{-1} using the *GoodVibes* program at 298.15 K (25˚C). Also, *GoodVibes* applied (i) 1 M standard concentration corrections to all individual calculations to account for reactions in solution (i.e. change in standard concentration from 1 atm to 1 M)[20] and (ii) multi-conformational corrections (G_{conf}) to all final Boltzmann weighted G to include the entropic stabilization created by multiple accessible low-lying conformers.[21] G values of all the energy profiles correspond to the Boltzmann weighted G of all the conformers found in each step, calculated by GoodVibes (see the *Thermochemical data* section).

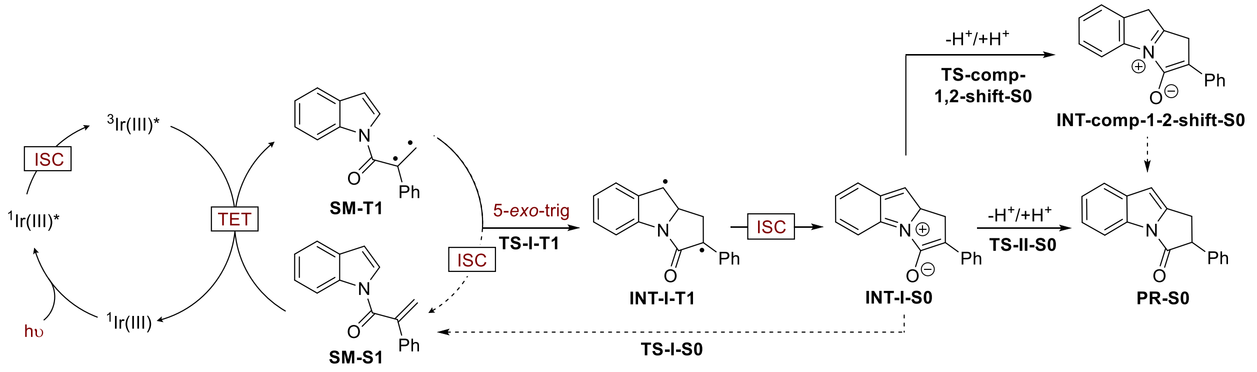

Figure S5. *Proposed main reaction pathways*

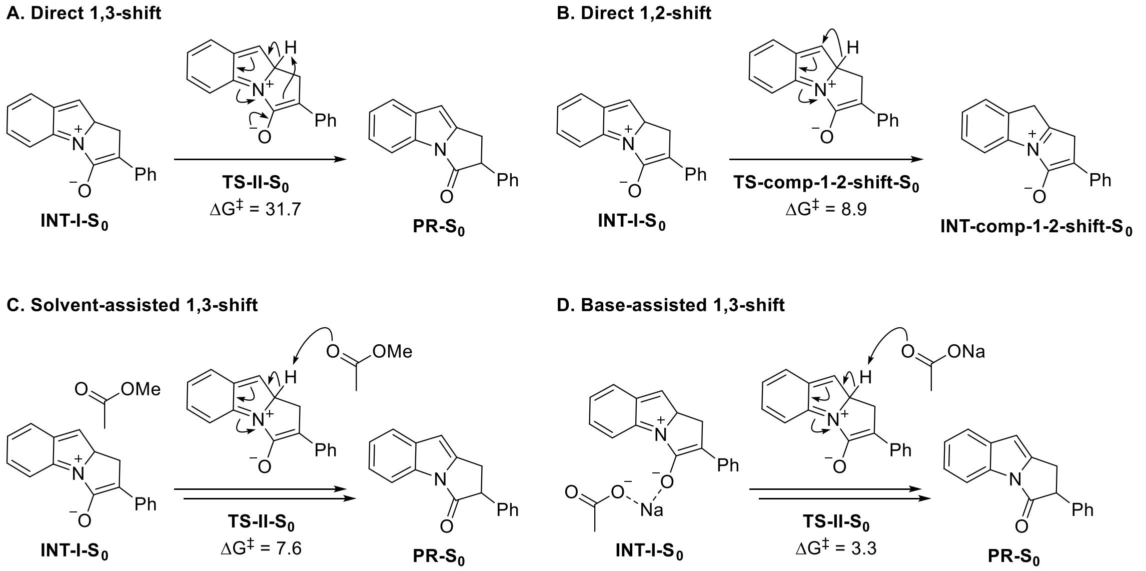

Figure S6. *Major competitive pathways found. In part C, AcOMe was used instead of AcOEt for simplicity, but similar results are expected for both solvents.*

[19] Grimme, S. Supramolecular binding thermodynamics by dispersion-corrected density functional theory. *Chem. Eur. J.* **2012**, *18*, 9955–9964.

[20] Bryantsev, V. S.; Diallo, M. S.; Goddard III, W. A. Calculation of Solvation Free Energies of Charged Solutes Using Mixed Cluster/Continuum Models. *J. Phys. Chem. B* **2008,** *112,* 9709–9719.

[21] Plata, R. E.; Singleton, D. A. A Case study of the mechanism of alcohol-mediated Morita Baylis–Hillman reactions. The importance of experimental observations. *J. Am. Chem. Soc.* **2015**, *137*, 3811–3826.

MECP absolute values and ISC molecular coordinates

<u>Input file for MECPro:</u>

```
! To begin a section we recommend using the headings for convenience
[general]
method = um062x/6-31++g(d,p) emp=gd3 scrf=(smd,solvent=ethylethanoate)
spinstates = 1/3
show_hessian = none
read_later = False
charge = 0

!This job has a max steps of 100. If it fails to converge, resubmit the new mecp file created after the job
max_steps = 100
max_stepsize = 0.1

[route]
A: force integral=ultrafinegrid scf=qc guess=mix
B: force integral=ultrafinegrid scf=qc

[geometry]
0 1/3
 C      3.00780700   -0.90045100    0.06907700
 C      2.34407100    0.31981400   -0.27324500
 C      2.93443600    1.55949300   -0.10857700
 C      4.23893500    1.59055000    0.40938100
 C      4.91891900    0.41048900    0.74366200
 C      4.32053800   -0.83479400    0.58147100
 C      2.13574200   -1.98001600   -0.17658000
 C      0.87793500   -1.45392700   -0.79791400
 H      2.40447400    2.46755700   -0.37065900
 H      4.73030000    2.54847700    0.54746400
 H      5.92997400    0.47183700    1.13455500
 H      4.84770100   -1.74579600    0.84850400
 H      2.35910500   -3.03208700   -0.05766600
 H      0.81967500   -1.75316200   -1.85659700
 N      1.05910100    0.00513800   -0.72588900
 C     -0.11579800    0.68425500   -0.42696100
 O     -0.18988200    1.90203800   -0.30969600
 C     -1.15676900   -0.33859200   -0.22641800
 C     -0.50094200   -1.68930500   -0.14552400
 H     -1.06205800   -2.47625000   -0.65524700
 H     -0.38169400   -1.98719700    0.90750200
 C     -2.54546600   -0.08382700   -0.00411600
 C     -3.10494000    1.21229600   -0.13957500
 C     -3.40918900   -1.14870800    0.35489800
 C     -4.46049100    1.41930800    0.07337800
 H     -2.46016300    2.03995900   -0.40910700
 C     -4.76183100   -0.92723000    0.56998400
 H     -3.00815900   -2.15111300    0.46971200
 C     -5.29658300    0.35673900    0.42937100
 H     -4.87167900    2.41814800   -0.03778500
 H     -5.40474500   -1.75687800    0.84806000
 H     -6.35563100    0.52766900    0.59618000
```

Output file from MECPro:

Convergence Check: actual (threshold) status
Max Gradient El.: 0.000521 (0.000700) Yes
RMS Gradient El.: 0.000207 (0.000500) Yes
Max Displacement: 0.002772 (0.004000) Yes
RMS Displacement: 0.001208 (0.002500) Yes
Difference in E: 0.000028 (0.000050) Yes

CONVERGENCE FOUND!
Initial Geometry (this geometry comes from the first job, which exceeded the max number (100) of optimization steps)

C	2.91058326	-0.90147179	-0.07527116
C	2.28361900	0.33239385	-0.40249801
C	2.84478344	1.55189119	-0.06688485
C	4.07324039	1.54679569	0.60648425
C	4.71153443	0.34000085	0.94323710
C	4.13962139	-0.88237753	0.61791688
C	2.10005758	-1.96342459	-0.52433483
C	0.83569798	-1.37742006	-1.08283546
H	2.35654990	2.47822611	-0.35021801
H	4.54453672	2.49008112	0.86336348
H	5.65909082	0.36355016	1.47473168
H	4.60935928	-1.82046224	0.89499422
H	2.34331073	-3.01757122	-0.51767801
H	0.53457842	-1.72876230	-2.07881418
N	1.08977187	0.09558003	-1.15716083
C	-0.08396015	0.77314863	-0.67454321
O	-0.22071811	1.98096199	-0.71363869
C	-1.03357973	-0.24382088	-0.16554993
C	-0.29994756	-1.55590538	-0.10248449
H	-0.88094424	-2.44256558	-0.35230154
H	0.08271215	-1.69591166	0.93519051
C	-2.42305937	-0.03729604	0.15048860
C	-3.06185581	1.21677024	-0.02281573
C	-3.22793632	-1.14473470	0.51768036
C	-4.43993446	1.32917034	0.11504530
H	-2.46876409	2.07946999	-0.29284597
C	-4.60051794	-1.01227875	0.66940371
H	-2.77472201	-2.11792406	0.66442325
C	-5.21900504	0.22106915	0.44968732
H	-4.91545621	2.29162249	-0.04479629
H	-5.20069547	-1.87710936	0.93173891
H	-6.29794653	0.31517295	0.53364166

Final Geometry

C	2.88298075	-0.89034536	0.01804777
C	2.26555606	0.30944651	-0.43740327
C	2.74370722	1.55888172	-0.09796671
C	3.89268361	1.62168937	0.71085253
C	4.52787581	0.45898774	1.15987649
C	4.03247281	-0.79917738	0.82613310
C	2.13921641	-1.99301608	-0.45555677
C	0.94485625	-1.48728821	-1.20033162
H	2.25190326	2.45840503	-0.45109175
H	4.29686823	2.59180509	0.98226882
H	5.41565779	0.53909265	1.78000366
H	4.51335967	-1.70134407	1.19192129
H	2.36786925	-3.04228567	-0.32129101
H	0.88448139	-1.86666064	-2.22787176
N	1.13976047	-0.01613543	-1.24778037

C	-0.08191491	0.64696775	-0.96131545
O	-0.20959056	1.85825374	-1.01594202
C	-1.07496024	-0.36225372	-0.54983715
C	-0.40774375	-1.69999283	-0.48911878
H	-0.97103251	-2.50446056	-0.96723586
H	-0.26543889	-1.98434582	0.56982530
C	-2.40693391	-0.09201965	-0.09435411
C	-2.98625359	1.19893384	-0.15865292
C	-3.18387952	-1.14749925	0.44318649
C	-4.28043721	1.41196408	0.29634471
H	-2.40909407	2.01882552	-0.56672883
C	-4.46993519	-0.91893968	0.91020968
H	-2.76562825	-2.14771031	0.49988927
C	-5.02711825	0.36108499	0.83545789
H	-4.70913739	2.40777411	0.23686473
H	-5.04371587	-1.73911068	1.33098202
H	-6.03642005	0.53734658	1.19396721

Energy of the final geometry (MECP) at the M06-2X-D3/Def2-QZVPP level (including SMD):

System	E (T_1) / a.u.	E (MECP) / a.u.	ΔE (T_1/MECP) / kcal·mol^{-1}
**INT-I-T_1**	-785.605542	-785.600503	3.2

Overlay of the final geometry of the ISC with **INT-I-T_1**:

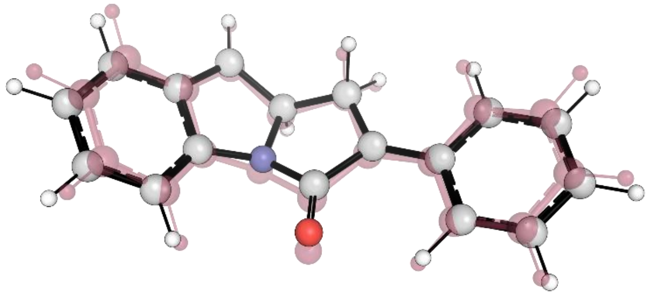

Figure S7. *Overlay of the ISC geometry and **INT-I-T_1**.*

IRC calculations

Some examples for IRC calculations of transition structures are shown in **Figure S7**.

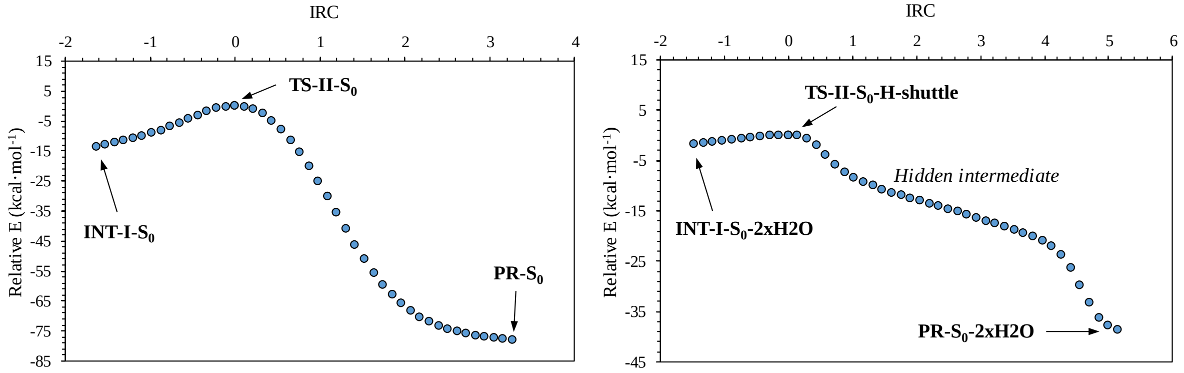

Figure S7. *IRCs of **TS-II-S_0** and **TS-II-S_0-H-shuttle**.*

Thermochemical data

Boltzmann weighted G (G_{av}) were calculated with *GoodVibes* as:

$$G_{av} = \sum_i G_i \times p_i \qquad (1)$$

where G_i is the relative Gibbs free energy of the corresponding conformers of a certain reaction step and pi is the probability of each conformer calculated as:

$$p_i = \frac{e^{\frac{-G_i}{RT}}}{\sum_i \left(e^{\frac{-G_i}{RT}}\right)} \qquad (2)$$

The **SM-S_0** reaction step was used as the reference with relative G (G_{rel}) = 0 kcal·mol^{-1}. The thermochemical data of all the systems studied including absolute energies, zero-point energies (ZPE) and T·S, among other parameters, at the M06-2X-D3/6-31++G(d,p) level, as well as the absolute energies, corrected final G and relative G obtained after single-point energy corrections with M06-2X-D3/Def2-QZVPP, were generated in an automated manner using *GoodVibes*. This automated process for creating G profiles provides a powerful method to avoid errors related to human manipulation of the data. The command lines used by *GoodVibes* are included in the raw output shown at the end of this section. Additionally, we tested that no input mistakes were made in the calculations by using the --check function of *GoodVibes* (included below).

Raw output file from *GoodVibes*:

Legend:

E_QZ = energy obtained in the single-point energy corrections
E = energy obtained in the geometry optimizations
ZPE = zero-point energy
H_QZ = enthalpy corrected with E_QZ
T.S = temperature times entropy with no correction
T.qh-S = temperature times entropy with quasi-harmonic S correction
G(T)_QZ = Gibbs free energy corrected only with E_QZ
qh-G(T)_QZ = Gibbs free energy with E_QZ and quasi-harmonic S correction *(final values used in the energy profiles)*
im freq = imaginary frequencies

GoodVibes information and input line:

GoodVibes v3.0.1 2020/05/04 18:09:22
REF: Luchini, G.; Alegre-Requena J. V.; Guan, Y.; Funes-Ardoiz, I.; Paton, R. S. (2019).
GoodVibes: GoodVibes 3.0.1 http://doi.org/10.5281/zenodo.595246
Requested: --spc QZ -c 1 --imag --pes .\Indole_cycliz.yaml --xyz --check

Temperature = 298.15 Kelvin   Concentration = 1.0 mol/L
All energetic values below shown in Hartree unless otherwise specified.

Using vibrational scale factor 1.0 for M062X/6-31++G(d,p) level of theory

Caution! Implicit solvation (SMD/CPCM) detected. Enthalpic and entropic terms cannot be safely separated. Use them at your own risk!

Entropic quasi-harmonic treatment: frequency cut-off value of 100.0 wavenumbers will be applied.

QS = Grimme: Using a mixture of RRHO and Free-rotor vibrational entropies.
REF: Grimme, S. Chem. Eur. J. 2012, 18, 9955-9964

Combining final single point energy with thermal corrections.

Absolute values in hartree:

Structure	E_QZ	E	ZPE	H_QZ	T.S	T.qh-S	G(T)_QZ	qh-G(T)_QZ	im freq
o **INT-comp-1-2-shift-S_0**	-785.645487	-785.329415	0.255524	-785.374778	0.053949	0.052276	-785.428727	-785.427054	
o **INT-I-S_0-1xAcOMe_a**	-1054.033088	-1053.598210	0.348559	-1053.662073	0.071071	0.067218	-1053.733144	-1053.729291	
o **INT-I-S_0-1xAcOMe_b**	-1054.032815	-1053.597819	0.348693	-1053.661799	0.070577	0.066890	-1053.732376	-1053.728689	
o **INT-I-S_0-1xAcOMe_c**	-1054.029319	-1053.593877	0.348223	-1053.658437	0.072891	0.068066	-1053.731328	-1053.726503	
o **INT-I-S_0-1xAcOMe_e**	-1054.030393	-1053.595654	0.348508	-1053.659356	0.071460	0.067416	-1053.730816	-1053.726772	
o **INT-I-S_0-1xAcONa_a**	-1176.492999	-1176.055065	0.308717	-1176.161874	0.071127	0.067378	-1176.233002	-1176.229253	
o **INT-I-S_0-1xAcONa_b**	-1176.500522	-1176.062659	0.308934	-1176.169115	0.072521	0.067712	-1176.241636	-1176.236827	
o **INT-I-S_0-1xAcONa_c**	-1176.498150	-1176.058990	0.308780	-1176.166620	0.074201	0.068787	-1176.240821	-1176.235406	
o **INT-I-S_0-2xH2O**	-938.510784	-938.119291	0.304962	-938.184118	0.070441	0.065902	-938.254559	-938.250020	
o **INT-I-S_0**	-785.608288	-785.293380	0.256369	-785.336952	0.053530	0.051887	-785.390481	-785.388839	
o **INT-I-T_1**	-785.605542	-785.291217	0.254653	-785.335765	0.054994	0.053209	-785.390759	-785.388974	
o **PR-S_0**	-785.691161	-785.376348	0.258127	-785.418135	0.054830	0.052238	-785.472965	-785.470373	
o **SM-S_0_a**	-785.657367	-785.340763	0.255966	-785.385759	0.056039	0.053476	-785.441798	-785.439236	
o **SM-S_0_b**	-785.655075	-785.338516	0.255740	-785.383663	0.056095	0.053539	-785.439758	-785.437202	
o **SM-S_0_c**	-785.658035	-785.341753	0.255683	-785.386614	0.056422	0.053649	-785.443036	-785.440263	
o **SM-S_0_d**	-785.656144	-785.339833	0.255418	-785.384956	0.056547	0.053685	-785.441503	-785.438641	
o **SM-T_1_a**	-785.568661	-785.253022	0.251720	-785.300751	0.057589	0.055248	-785.358341	-785.356000	
o **SM-T_1_b**	-785.536071	-785.219599	0.250307	-785.269175	0.058927	0.056189	-785.328102	-785.325364	
o **SM-T_1_c**	-785.540331	-785.224665	0.250147	-785.273754	0.058349	0.055697	-785.332103	-785.329451	
o **SM-T_1_d**	-785.542845	-785.227663	0.251385	-785.275364	0.057196	0.054869	-785.332560	-785.330233	
o **SM-T_1_e**	-785.538178	-785.221744	0.250453	-785.271205	0.058704	0.056044	-785.329909	-785.327249	
o **TS-comp-1-2-shift-S_0**	-785.591047	-785.274677	0.252960	-785.323398	0.052628	0.051284	-785.376026	-785.374682	-959.77
o **TS-I-S_0**	-785.591516	-785.276857	0.254709	-785.322120	0.052909	0.051277	-785.375028	-785.373396	-592.98
o **TS-I-T_1**	-785.552605	-785.237610	0.251563	-785.285837	0.055257	0.053354	-785.341094	-785.339191	-597.85
o **TS-II-S_0-H-shuttle**	-938.500242	-938.109479	0.301009	-938.179182	0.065136	0.061812	-938.244318	-938.240994	-589.15
o **TS-II-S_0**	-785.552798	-785.237092	0.251072	-785.287016	0.052843	0.051276	-785.339860	-785.338293	-1367.99
o **TS-II-S_0-1xAcOMe_a**	-1054.015938	-1053.580378	0.343014	-1053.650824	0.071057	0.066865	-1053.721880	-1053.717688	-748.20
o **TS-II-S_0-1xAcOMe_b**	-1054.012094	-1053.576223	0.343006	-1053.646854	0.071684	0.067272	-1053.718538	-1053.714126	-817.66
o **TS-II-S_0-1xAcONa_a**	-1176.478237	-1176.039875	0.304847	-1176.151389	0.072598	0.067474	-1176.223988	-1176.218864	-228.20
o **TS-II-S_0-1xAcONa_b**	-1176.492223	-1176.054789	0.305054	-1176.165407	0.070571	0.066295	-1176.235978	-1176.231702	-524.06

Results from --check:
Checks for thermochemistry calculations (frequency calculations):

o Using Gaussian 16 Revision C.01 in all calculations.
o Using M062X/6-31++G(d,p) in all calculations.
o Using scrf=(smd,solvent=ethylethanoate) in all calculations.
o Using a standard concentration of 1 M for solvent phase.
x Caution! Different charge and multiplicity found:
    0 1: INT-comp-1-2-shift-S_0.log, INT-I-S_0-2xH2O.log, …
    0 3: INT-I-T1.log, SM-T_1_a.log, SM-T_1_b.log, …
o No duplicates or enantiomers found
- No linear molecules found.
o Using empiricaldispersion=(gd3) in all calculations.

Checks for single-point corrections:

o Using Gaussian 16 Revision C.01 in all the single-point corrections.
o Using scrf=(smd,solvent=ethylethanoate) in all single-point corrections.
o Using M062X/def2QZVPP in all the single-point corrections.
x Caution! Different charge and multiplicity found:
    0 1: INT-comp-1-2-shift-S_0.log, INT-I-S_0-2xH2O.log, …
    0 3: INT-I-T1.log, SM-T_1_a.log, SM-T_1_b.log, …
o No potential differences found between frequency and single-point geometries (based on input coordinates).
o Using empiricaldispersion=(gd3) in all the singe-point calculations.

Relative values in kcal·mol^{-1}. GoodVibes, with the --pes option, calculates the Boltzmann weighted thermochemistry values and applies multiconfigurational entropy corrections:
G_{conf} correction requested to be applied to below relative values using quasi-harmonic Boltzmann factors

RXN: Indole photocyc. (kcal/mol)	DE_QZ	DE	DZPE	DH_QZ	T.DS	T.qh-DS	DG(T)_QZ	qh-DG(T)_QZ
o **SM-S$_0$**	0.0	0.0	0.0	0.0	0.0	0.0	0.0	0.0
o **SM-T$_1$**	55.8	55.4	-2.5	53.6	0.2	0.5	53.4	53.1
o **TS-I-T$_1$**	65.9	65.0	-2.6	63.0	-1.3	-0.7	64.2	63.7
o **INT-I-T$_1$**	32.7	31.4	-0.7	31.6	-1.4	-0.8	33.0	32.4
o **INT-I-S$_0$**	30.9	30.0	0.4	30.9	-2.3	-1.7	33.2	32.5
o **PR-S$_0$**	-21.1	-22.0	1.5	-20.1	-1.5	-1.4	-18.5	-18.6

RXN: Retro-cyclization (kcal/mol)	DE_QZ	DE	DZPE	DH_QZ	T.DS	T.qh-DS	DG(T)_QZ	qh-DG(T)_QZ
o **SM-S$_0$**	0.0	0.0	0.0	0.0	0.0	0.0	0.0	0.0
o **TS-I-S$_0$**	41.5	40.4	-0.6	40.2	-2.7	-2.0	42.9	42.2

RXN: H shuttle (kcal/mol)	DE_QZ	DE	DZPE	DH_QZ	T.DS	T.qh-DS	DG(T)_QZ	qh-DG(T)_QZ
o **INT-I-S$_0$-2xH2O**	0.0	0.0	0.0	0.0	0.0	0.0	0.0	0.0
o **TS-II-S$_0$-H-shuttle**	6.6	6.2	-2.5	3.1	-3.3	-2.6	6.4	5.7

RXN: Competitive rxns (kcal/mol)	DE_QZ	DE	DZPE	DH_QZ	T.DS	T.qh-DS	DG(T)_QZ	qh-DG(T)_QZ
o **SM-S$_0$**	0.0	0.0	0.0	0.0	0.0	0.0	0.0	0.0
o **TS-II-S$_0$**	65.8	65.4	-2.9	62.2	-2.8	-2.0	65.0	64.2
o **TS-comp-1-2-shift-S$_0$**	41.8	41.8	-1.7	39.4	-2.9	-2.0	42.3	41.4
o **INT-comp-1-2-shift-S$_0$**	7.6	7.4	-0.1	7.1	-2.1	-1.4	9.2	8.6

RXN: AcOMe as base (kcal/mol)	DE_QZ	DE	DZPE	DH_QZ	T.DS	T.qh-DS	DG(T)_QZ	qh-DG(T)_QZ
o **INT-I-S$_0$-1xAcOMe**	0.0	0.0	0.0	0.0	0.0	0.0	0.0	0.0
o **TS-II-S$_0$-1xAcOMe**	10.6	11.0	-3.5	6.9	-0.4	-0.6	7.3	7.6

RXN: AcONa as base (kcal/mol)	DE_QZ	DE	DZPE	DH_QZ	T.DS	T.qh-DS	DG(T)_QZ	qh-DG(T)_QZ
o **INT-I-S$_0$-1xAcONa**	0.0	0.0	0.0	0.0	0.0	0.0	0.0	0.0
o **TS-II-S$_0$-1xAcONa**	4.9	4.5	-2.4	2.0	-1.7	-1.3	3.7	3.3

Molecular coordinates

The generation of xyz files was automated with *GoodVibes* using the --xyz option:

32

INT-comp-1-2-shift-S_0

C	-3.149467	-0.889331	0.000135
C	-2.414724	0.303959	0.000052
C	-2.990609	1.564037	-0.000183
C	-4.387339	1.602749	-0.000274
C	-5.145258	0.427965	-0.000227
C	-4.535087	-0.831589	-0.000009
C	-2.182799	-2.052915	0.000252
C	-0.871423	-1.339148	0.000239
H	-2.380625	2.459568	-0.000247
H	-4.890952	2.564010	-0.000485
H	-6.228682	0.494034	-0.000306
H	-5.133107	-1.737797	0.000063
H	-2.279329	-2.695721	0.883223
N	-1.043507	-0.053452	0.000117
C	0.251737	0.718837	0.000095
O	0.204931	1.954579	0.000025
C	1.216032	-0.283028	0.000068
C	0.577540	-1.650896	0.000196
H	0.818911	-2.263377	0.880862
H	0.818525	-2.263318	-0.880679
C	2.649272	-0.069173	-0.000009
C	3.209806	1.228191	0.000315
C	3.541112	-1.162930	-0.000427
C	4.589449	1.409386	0.000277
H	2.544611	2.085163	0.000592
C	4.920125	-0.971858	-0.000442
H	3.148406	-2.176465	-0.000800
C	5.458837	0.315745	-0.000083
H	4.990954	2.419437	0.000527
H	5.578198	-1.836849	-0.000750
H	6.534251	0.464487	-0.000083
H	-2.279292	-2.695637	-0.882792

38

INT-I-S_0-2xH_2O

C	3.223523	0.458620	-0.659235
C	2.484066	-0.474177	0.184862
C	3.104810	-1.634601	0.730792
C	4.428958	-1.806172	0.447328
C	5.193986	-0.877077	-0.354984
C	4.623988	0.230974	-0.896607
C	2.342891	1.405734	-1.106424
C	1.033021	1.167130	-0.461477
H	2.535436	-2.325814	1.339689
H	4.943565	-2.676212	0.844323
H	6.246401	-1.085208	-0.516591
H	5.191864	0.932919	-1.498647
H	2.567439	2.263062	-1.730363
H	0.902963	1.999737	0.260970
N	1.232196	-0.061081	0.274151
C	-0.024619	-0.775870	0.526509
O	-0.027897	-1.750136	1.291889
C	-0.969651	-0.116462	-0.269382
C	-0.336305	0.944680	-1.150101
H	-0.906179	1.879210	-1.187302
H	-0.183106	0.598569	-2.179202
C	-2.370191	-0.481037	-0.369424
C	-2.955541	-1.452860	0.477007
C	-3.212742	0.156928	-1.307122
C	-4.309373	-1.757325	0.384102
H	-2.328133	-1.959941	1.201993
C	-4.568050	-0.153414	-1.389239
H	-2.799911	0.900553	-1.983129
C	-5.129995	-1.111890	-0.545117
H	-4.730117	-2.508134	1.047739
H	-5.187455	0.355662	-2.122893
H	-6.186143	-1.354398	-0.611391
O	-2.143375	1.902537	1.815775
H	-3.064725	1.965116	1.531665
H	-1.788463	1.123066	1.352190
O	-0.136974	3.676871	1.094633
H	0.193993	4.163526	1.858077
H	-0.919487	3.182593	1.406502

32

INT-I-S_0

C	3.062307	-0.919267	-0.020276
C	2.307067	0.321579	-0.179406
C	2.892480	1.591155	0.096963
C	4.199656	1.586207	0.488322
C	4.981083	0.374658	0.619275
C	4.445885	-0.848976	0.372217
C	2.213943	-1.961872	-0.267095
C	0.905400	-1.408031	-0.695791
H	2.308911	2.497151	-0.010930
H	4.688283	2.530825	0.707914
H	6.019155	0.465402	0.921558
H	5.028097	-1.759856	0.466956
H	2.457286	-3.017781	-0.263158
H	0.814928	-1.631473	-1.772870
N	1.074589	0.021044	-0.544569
C	-0.203890	0.723562	-0.399857
O	-0.226830	1.963585	-0.462658
C	-1.135432	-0.292480	-0.171976
C	-0.479813	-1.652380	-0.042550
H	-1.022022	-2.450550	-0.558283
H	-0.339030	-1.955543	1.001536
C	-2.555825	-0.086372	0.011912
C	-3.157491	1.182732	-0.163892
C	-3.399169	-1.159055	0.378910
C	-4.526125	1.354424	0.011482
H	-2.529679	2.024109	-0.435805
C	-4.769225	-0.977345	0.548197
H	-2.975376	-2.146341	0.540604
C	-5.346797	0.279815	0.366099
H	-4.959188	2.341060	-0.131392
H	-5.388530	-1.824745	0.830218
H	-6.414645	0.421631	0.501700

32

INT-I-T_1

C	3.007807	-0.900451	0.069077
C	2.344071	0.319814	-0.273245
C	2.934436	1.559493	-0.108577
C	4.238935	1.590550	0.409381
C	4.918919	0.410489	0.743662
C	4.320538	-0.834794	0.581471
C	2.135742	-1.980016	-0.176580
C	0.877935	-1.453927	-0.797914
H	2.404474	2.467557	-0.370659
H	4.730300	2.548477	0.547464
H	5.929974	0.471837	1.134555
H	4.847701	-1.745796	0.848504
H	2.359105	-3.032087	-0.057666
H	0.819675	-1.753162	-1.856597
N	1.059101	0.005138	-0.725889
C	-0.115798	0.684255	-0.426961
O	-0.189882	1.902038	-0.309696
C	-1.156769	-0.338592	-0.226418
C	-0.500942	-1.689305	-0.145524
H	-1.062058	-2.476250	-0.655247
H	-0.381694	-1.987197	0.907502
C	-2.545466	-0.083827	-0.004116
C	-3.104940	1.212296	-0.139575
C	-3.409189	-1.148708	0.354898
C	-4.460491	1.419308	0.073378
H	-2.460163	2.039959	-0.409107
C	-4.761831	-0.927230	0.569984
H	-3.008159	-2.151113	0.469712
C	-5.296583	0.356739	0.429371
H	-4.871679	2.418148	-0.037785
H	-5.404745	-1.756878	0.848060
H	-6.355631	0.527669	0.596180

32

PR-S_0

C	2.971034	0.856304	-0.252508
C	2.271763	-0.324457	0.097867
C	2.870912	-1.581430	0.146004
C	4.223946	-1.642792	-0.173694
C	4.943465	-0.487288	-0.527142
C	4.332388	0.761304	-0.569883
C	2.033278	1.965665	-0.192560
C	0.834904	1.444130	0.176181
H	2.305391	-2.465424	0.418383
H	4.732385	-2.601538	-0.150133
H	5.997933	-0.574549	-0.771070
H	4.898170	1.646970	-0.843946
H	2.249623	3.003100	-0.406289
H	-1.432446	0.609268	2.030508
N	0.963196	0.067380	0.355845
C	-0.194302	-0.560317	0.781201
O	-0.309556	-1.746821	0.990916
C	-1.256038	0.537107	0.951585
C	-0.588761	1.843745	0.435348
H	-0.659220	2.651366	1.167013
H	-1.066708	2.186769	-0.487072
C	-2.552670	0.166212	0.268343
C	-3.738730	0.055362	0.994917
C	-2.574948	-0.072260	-1.111488
C	-4.933471	-0.280409	0.354687
H	-3.728270	0.233193	2.067315
C	-3.763733	-0.410349	-1.751656
H	-1.653627	0.004565	-1.687356
C	-4.948420	-0.513375	-1.018584
H	-5.849783	-0.360880	0.931873
H	-3.766902	-0.596470	-2.821468
H	-5.876163	-0.776383	-1.517710

32

SM-S_0_a

C	3.149578	0.698934	0.607345
C	2.289265	-0.171464	-0.097404
C	2.751706	-1.350186	-0.689898
C	4.106577	-1.637349	-0.557386
C	4.977212	-0.784110	0.142326
C	4.509388	0.385357	0.726931
C	2.349943	1.807661	1.077709
C	1.080116	1.602234	0.654153
H	2.084878	-2.013942	-1.223091
H	4.496805	-2.546271	-1.004894
H	6.027843	-1.045220	0.224668
H	5.180182	1.047992	1.265885
H	2.691575	2.646667	1.667871
H	0.189184	2.188405	0.818060
N	1.007979	0.403842	-0.076106
C	-0.141832	-0.173507	-0.611785
O	-0.149275	-1.328905	-0.990263
C	-1.359374	0.701449	-0.742959
C	-1.262452	1.854346	-1.415038
H	-0.311613	2.198527	-1.812565
H	-2.132191	2.480576	-1.591840
C	-2.624844	0.153454	-0.196357
C	-2.605126	-0.626340	0.967637
C	-3.854982	0.425807	-0.808664
C	-3.790878	-1.104973	1.519819
H	-1.657957	-0.855393	1.450415
C	-5.039943	-0.054720	-0.257105
H	-3.881782	0.998254	-1.731393
C	-5.012199	-0.818735	0.910159
H	-3.759603	-1.703471	2.425137
H	-5.985541	0.158127	-0.746645
H	-5.936351	-1.197054	1.336335

32

SM-S_0_b

C	3.210433	0.088146	-0.259111
C	1.842149	-0.117817	0.030249
C	1.326480	-1.401276	0.242237
C	2.219383	-2.466322	0.202475
C	3.589070	-2.273190	-0.048456
C	4.091068	-1.002053	-0.286548
C	3.394345	1.499098	-0.511278
C	2.183033	2.092936	-0.388713
H	0.274490	-1.579638	0.425333
H	1.842415	-3.471147	0.366046
H	4.254704	-3.130581	-0.067997
H	5.144321	-0.847908	-0.502144
H	4.324447	1.995255	-0.751659

H 1.885261 3.125377 -0.495850
N 1.218555 1.142675 -0.034545
C -0.127171 1.491868 0.113810
O -0.546859 2.534404 -0.347397
C -1.002205 0.584891 0.930973
C -0.645018 0.343808 2.196843
H 0.285860 0.732391 2.601078
H -1.267999 -0.251025 2.858601
C -2.244099 0.095403 0.287711
C -2.262045 -0.152076 -1.091632
C -3.398818 -0.163342 1.038026
C -3.402505 -0.665696 -1.704983
H -1.376776 0.049947 -1.690617
C -4.538686 -0.674708 0.423648
H -3.410604 0.055042 2.102022
C -4.543532 -0.930433 -0.948536
H -3.398485 -0.857286 -2.773622
H -5.429059 -0.864103 1.015599
H -5.434876 -1.325716 -1.426088
32
SM-S$_0$_c
C 2.410492 -0.966770 -0.765108
C 2.018698 0.173475 -0.029536
C 2.865300 0.782047 0.901159
C 4.123825 0.215342 1.079118
C 4.532087 -0.917656 0.354765
C 3.683774 -1.515582 -0.568336
C 1.301159 -1.331947 -1.617896
C 0.299759 -0.451809 -1.379775
H 2.558977 1.659681 1.454255
H 4.806189 0.665153 1.793816
H 5.523586 -1.327892 0.520297
H 3.995604 -2.392871 -1.127513
H 1.269398 -2.151042 -2.322780
H -0.680572 -0.380541 -1.827296
N 0.702553 0.479330 -0.410556
C -0.055055 1.547069 0.067877
O 0.456876 2.438147 0.717978
C -1.526103 1.530060 -0.256368
C -2.042815 2.644346 -0.785673
H -1.410762 3.498641 -1.008068
H -3.103747 2.732962 -0.999839
C -2.317405 0.321839 0.090771
C -1.925799 -0.491195 1.163345
C -3.465667 -0.021825 -0.635230
C -2.675780 -1.611536 1.513634
H -1.033640 -0.246293 1.735714
C -4.214745 -1.141967 -0.283624
H -3.762087 0.578701 -1.490807
C -3.823383 -1.939633 0.792427
H -2.361491 -2.227903 2.350270
H -5.099526 -1.398423 -0.858515
H -4.404859 -2.816195 1.061226
32
SM-S$_0$_d
C 2.556657 -0.391321 0.452311
C 1.406682 -0.020151 -0.280624
C 0.882397 -0.840702 -1.283851
C 1.500655 -2.067267 -1.498079
C 2.622594 -2.467323 -0.751402
C 3.162514 -1.632923 0.217010
C 2.882323 0.707990 1.332018
C 1.966037 1.682765 1.116653
H 0.037068 -0.541299 -1.890958
H 1.108186 -2.725308 -2.267165
H 3.078004 -3.432988 -0.947951
H 4.044015 -1.926495 0.779454
H 3.701814 0.749982 2.036056
H 1.847029 2.656544 1.567876
N 1.038046 1.262126 0.156794
C -0.048444 2.066421 -0.207104
O -0.017882 3.261971 0.010524
C -1.248219 1.391957 -0.810296
C -1.745605 1.923536 -1.932791
H -1.261697 2.772063 -2.406698
H -2.645268 1.532106 -2.398250
C -1.846937 0.241456 -0.085846
C -1.707968 0.131219 1.303961
C -2.570649 -0.743308 -0.772461
C -2.289694 -0.931353 1.992774
H -1.149828 0.881854 1.859079
C -3.153726 -1.802636 -0.083482
H -2.657887 -0.692924 -1.854553
C -3.015365 -1.900451 1.301979
H -2.174306 -0.999423 3.070114
H -3.705719 -2.560799 -0.630783
H -3.464187 -2.731420 1.837416
32
SM-T$_1$_a
C -3.268499 0.748505 -0.464515
C -2.343143 -0.201977 0.023527
C -2.752801 -1.427425 0.558938
C -4.119593 -1.680087 0.593251
C -5.055421 -0.747892 0.109177
C -4.640853 0.466795 -0.419323
C -2.509545 1.885510 -0.930559
C -1.199456 1.615043 -0.704622
H -2.035808 -2.152044 0.921702
H -4.469837 -2.623880 0.999977
H -6.114175 -0.984889 0.150490
H -5.361673 1.189467 -0.790682
H -2.901627 2.781984 -1.390308
H -0.322288 2.193594 -0.952821
N -1.066095 0.354463 -0.113973
C 0.141996 -0.287021 0.204470
O 0.172538 -1.503437 0.323880
C 1.312965 0.579915 0.460012
C 1.105029 1.830174 1.201771
H 1.151558 2.800376 0.718267
H 0.991434 1.802550 2.281876
C 2.643575 0.117507 0.148623
C 2.890785 -1.015186 -0.666925
C 3.761048 0.844985 0.626361
C 4.189096 -1.397667 -0.970966
H 2.059182 -1.587782 -1.059017
C 5.056151 0.449211 0.322585
H 3.598753 1.722150 1.246082
C 5.279155 -0.674622 -0.476784
H 4.355441 -2.266863 -1.599999
H 5.896197 1.019081 0.707831
H 6.292281 -0.982086 -0.716753
32
SM-T$_1$_b
C 3.164466 0.039682 -0.309299
C 1.763297 -0.110363 0.042888
C 1.225729 -1.327106 0.353843
C 2.097141 -2.452617 0.388012
C 3.468807 -2.331898 0.102152
C 4.017504 -1.114167 -0.245229
C 3.381463 1.325436 -0.722366
C 2.090411 2.050963 -0.635290
H 0.174391 -1.462810 0.571892
H 1.682267 -3.422301 0.639958
H 4.099485 -3.214184 0.147805
H 5.072030 -1.014411 -0.480982
H 4.301853 1.757913 -1.089590
H 1.883977 3.107566 -0.682490
N 1.153111 1.183130 -0.090899
C -0.130531 1.607343 0.191992
O -0.499770 2.724536 -0.176232
C -1.027815 0.714559 0.999138
C -0.770293 0.548823 2.301178
H 0.099149 1.010613 2.761463
H -1.405056 -0.061958 2.937296
C -2.188101 0.123030 0.288365
C -2.101709 -0.136066 -1.086390
C -3.368562 -0.212862 0.965627
C -3.160840 -0.735545 -1.765078
H -1.196667 0.127351 -1.629887
C -4.426933 -0.810372 0.286965
H -3.465740 0.014333 2.023308
C -4.326009 -1.077107 -1.079748
H -3.074963 -0.933311 -2.829243
H -5.337928 -1.058045 0.823699
H -5.154566 -1.539339 -1.607662
32
SM-T$_1$_c
C 2.346535 -1.084292 -0.668963
C 2.004550 0.196213 -0.077609
C 2.888635 0.906731 0.688693
C 4.167023 0.329189 0.917575
C 4.530500 -0.917200 0.365184
C 3.653555 -1.629541 -0.422751
C 1.272525 -1.531106 -1.388861
C 0.211156 -0.513046 -1.279584
H 2.626384 1.867939 1.107633
H 4.878272 0.867849 1.534202
H 5.521513 -1.313478 0.562980
H 3.928852 -2.584211 -0.858956
H 1.181685 -2.448220 -1.954339
H -0.704474 -0.425941 -1.841062
N 0.651948 0.486433 -0.438124
C -0.054713 1.599773 -0.006198
O 0.543513 2.561158 0.497225
C -1.534601 1.571823 -0.201956
C -2.143377 2.716766 -0.549639
H -1.561658 3.612147 -0.745565
H -3.224985 2.785191 -0.619413
C -2.298285 0.329514 0.102339
C -1.894273 -0.509867 1.150808
C -3.443172 -0.010238 -0.629303
C -2.622285 -1.654989 1.463791
H -1.007716 -0.259508 1.729395
C -4.172039 -1.156306 -0.315524
H -3.752142 0.617408 -1.461033
C -3.764231 -1.982894 0.731369
H -2.297905 -2.291103 2.281957
H -5.054944 -1.407714 -0.895863
H -4.329636 -2.877838 0.972903
32
SM-T$_1$_d
C 2.446777 0.035381 0.445438
C 1.287598 0.312445 -0.329016
C 0.965704 -0.395364 -1.461679
C 1.831327 -1.452659 -1.811897
C 2.965552 -1.769708 -1.054183
C 3.293461 -1.029824 0.077525
C 2.494867 1.001783 1.467637
C 1.341514 1.834533 1.294068
H 0.093114 -0.158320 -2.060664
H 1.604631 -2.036044 -2.698513
H 3.596576 -2.596826 -1.360289
H 4.177505 -1.259136 0.663979
H 3.241013 1.121725 2.241105
H 1.025254 2.687088 1.881321
N 0.626418 1.417058 0.266765
C -0.602431 2.111387 -0.217875
O -0.449268 3.331385 -0.449700
C -1.759823 1.306137 -0.399017
C -2.869148 1.844313 -1.036549
H -2.841639 2.855351 -1.426863
H -3.793247 1.281743 -1.111472
C -1.809619 -0.095600 0.103740
C -1.398875 -0.432962 1.402659
C -2.288156 -1.123663 -0.722410
C -1.430119 -1.754381 1.846357
H -1.063620 0.350313 2.077829
C -2.328990 -2.444356 -0.278787
H -2.609694 -0.880912 -1.732174
C -1.890103 -2.768293 1.005659
H -1.106203 -1.989692 2.856279
H -2.696353 -3.223278 -0.941066
H -1.915325 -3.797514 1.350760
32
SM-T$_1$_e

C 3.153079 0.642586 0.684145
C 2.255399 -0.133653 -0.152907
C 2.661425 -1.264009 -0.804495
C 4.022920 -1.662106 -0.649794
C 4.920316 -0.931388 0.149039
C 4.516508 0.205754 0.819755
C 2.473320 1.717787 1.181334
C 1.084346 1.666675 0.659224
H 1.983274 -1.838028 -1.419127
H 4.366021 -2.551061 -1.167611
H 5.947134 -1.272371 0.238998
H 5.202586 0.772017 1.441014
H 2.850425 2.503665 1.821468
H 0.202981 2.128021 1.077608
N 0.976878 0.513995 -0.126261
C -0.152960 0.009399 -0.731690
O -0.142243 -1.081781 -1.302832
C -1.399668 0.848789 -0.683120
C -1.388595 2.097935 -1.162925
H -0.476093 2.545882 -1.547643
H -2.287730 2.707248 -1.182188
C -2.616554 0.176249 -0.161494
C -2.505943 -0.776945 0.859823
C -3.889358 0.495086 -0.652270
C -3.642311 -1.377514 1.396626
H -1.523983 -1.047295 1.240824
C -5.025493 -0.107598 -0.117447
H -3.988402 1.203198 -1.470079
C -4.906439 -1.042837 0.911239
H -3.539227 -2.109702 2.191845
H -6.004707 0.144841 -0.513277
H -5.792422 -1.515396 1.324396
32
TS-comp-1-2-shift-S$_0$
C 3.116871 0.904019 -0.019254
C 2.372161 -0.311522 0.033448
C 3.003379 -1.571954 -0.011296
C 4.376016 -1.569153 -0.120625
C 5.136467 -0.365917 -0.167142
C 4.528695 0.865509 -0.105291
C 2.185675 1.975495 0.029502
C 0.885840 1.370346 0.185282
H 2.419519 -2.483162 0.034669
H 4.902702 -2.517408 -0.167844
H 6.216323 -0.432046 -0.249102
H 5.103948 1.785339 -0.135073
H 2.373154 3.041677 0.001796
H 1.376203 1.800230 1.231162
N 1.060104 0.007174 0.161953
C -0.227946 -0.717825 0.094876
O -0.214542 -1.957392 0.113201
C -1.193028 0.286086 0.021096
C -0.582186 1.674356 -0.006711
H -0.962796 2.336100 0.779107
H -0.714910 2.190824 -0.965879
C -2.622411 0.070750 -0.015257
C -3.189638 -1.225253 0.035747
C -3.514505 1.163047 -0.106431
C -4.568335 -1.404207 0.001622
H -2.527126 -2.081488 0.100946
C -4.893618 0.972726 -0.138027
H -3.122488 2.175544 -0.156446
C -5.436233 -0.311747 -0.084310
H -4.971885 -2.412777 0.042449
H -5.548816 1.837239 -0.207800
H -6.511445 -0.459690 -0.110625
32
TS-I-S$_0$
C -3.045549 -0.905109 0.284878
C -2.315453 0.321763 0.174127
C -2.919631 1.521590 -0.248998
C -4.269736 1.475322 -0.525010
C -5.026066 0.275722 -0.391536
C -4.438370 -0.901797 0.004502
C -2.144707 -1.907169 0.686274
C -0.855156 -1.330805 0.773634
H -2.340296 2.433374 -0.333864
H -4.776876 2.380296 -0.845059
H -6.089485 0.302126 -0.607837
H -5.017332 -1.814374 0.107909
H -2.383478 -2.930072 0.945657
H -0.198726 -1.595178 1.598293
N -1.010583 0.061541 0.469723
C 0.158885 0.763851 0.038958
O 0.195739 1.989878 0.024287
C 1.121890 -0.253078 -0.305622
C 0.463823 -1.500201 -0.552668
H 1.041045 -2.420681 -0.456427
H -0.275686 -1.543930 -1.349353
C 2.561934 -0.076456 -0.190760
C 3.113591 1.063723 0.431816
C 3.453061 -1.048291 -0.691062
C 4.490428 1.208075 0.562737
H 2.450960 1.835241 0.809180
C 4.829159 -0.905304 -0.541878
H 3.063948 -1.913658 -1.219512
C 5.358511 0.223638 0.084976
H 4.889603 2.095012 1.046489
H 5.492025 -1.671426 -0.933885
H 6.432769 0.340617 0.189844
32
TS-I-T$_1$
C -3.141545 0.800888 -0.091630
C -2.294696 -0.324387 -0.249030
C -2.652263 -1.596703 0.194177
C -3.906736 -1.733523 0.784014
C -4.775093 -0.636354 0.929687
C -4.403775 0.630969 0.500048
C -2.444172 1.938380 -0.621353
C -1.190131 1.512179 -1.021509
H -1.985805 -2.441962 0.068515
H -4.223235 -2.712594 1.130450
H -5.748228 -0.785797 1.387265
H -5.070997 1.479082 0.622996
H -2.831983 2.942064 -0.729987
H -0.553747 1.990022 -1.756031
N -1.113407 0.114272 -0.858903
C 0.139644 -0.494923 -0.614111
O 0.277127 -1.705156 -0.716411
C 1.132100 0.472105 -0.123638
C 0.579230 1.772752 0.306572
H 1.001375 2.689252 -0.096354
H 0.185385 1.843164 1.321089
C 2.522454 0.135228 0.053673
C 3.109703 -1.034364 -0.491976
C 3.360116 1.021430 0.775707
C 4.461674 -1.293196 -0.314630
H 2.498214 -1.727487 -1.054584
C 4.707351 0.745494 0.958378
H 2.936291 1.926556 1.200061
C 5.267354 -0.412720 0.412862
H 4.893312 -2.190753 -0.747033
H 5.325844 1.435795 1.523775
H 6.323116 -0.625524 0.550001
38
TS-II-S$_0$-H-shuttle
C 3.138884 0.481684 -0.712260
C 2.384425 -0.565791 -0.082935
C 3.007908 -1.726150 0.435612
C 4.373556 -1.800337 0.308760
C 5.146606 -0.762134 -0.299221
C 4.554244 0.369175 -0.797438
C 2.227666 1.462817 -1.140596
C 0.940720 1.068592 -0.672324
H 2.418492 -2.507184 0.900586
H 4.891803 -2.676769 0.686282
H 6.223247 -0.882829 -0.360968
H 5.138670 1.158913 -1.259377
H 2.456685 2.408528 -1.616591
H 1.114454 1.850875 0.315556
N 1.090976 -0.199841 -0.094108
C -0.188697 -0.861598 0.154614
O -0.206639 -1.952205 0.745085
C -1.153555 0.006358 -0.377634
C -0.524139 1.203193 -1.071597
H -0.948096 2.163770 -0.752200
H -0.614366 1.156887 -2.164384
C -2.584644 -0.229101 -0.373219
C -3.160949 -1.356209 0.260600
C -3.464648 0.683473 -0.997560
C -4.539122 -1.546017 0.264925
H -2.506787 -2.076210 0.740287
C -4.842534 0.484231 -0.985149
H -3.063162 1.558338 -1.501731
C -5.395008 -0.631058 -0.354061
H -4.950828 -2.422855 0.758144
H -5.489015 1.207097 -1.475877
H -6.469492 -0.786107 -0.346344
O -1.325627 1.536699 2.253210
H -1.554821 1.081247 3.074000
H -1.447163 0.889809 1.528755
O 1.001751 2.548380 1.578092
H 1.728933 2.311941 2.173219
H 0.154452 2.213671 1.985193
32
TS-II-S$_0$
C 3.035365 -0.898974 -0.206663
C 2.384945 0.284511 0.265302
C 3.066490 1.502803 0.373802
C 4.408959 1.501009 0.030965
C 5.078900 0.334468 -0.413799
C 4.405842 -0.863088 -0.542950
C 2.059467 -1.927752 -0.272456
C 0.831567 -1.351835 0.097516
H 2.561703 2.398336 0.716156
H 4.970879 2.427108 0.107885
H 6.133518 0.394196 -0.661136
H 4.908192 -1.758792 -0.895162
H 2.205846 -2.942466 -0.621984
H 0.236472 -0.943065 -0.970912
N 1.084436 -0.031881 0.551021
C -0.170861 0.688973 0.316405
O -0.231443 1.904488 0.374210
C -1.143177 -0.348367 0.023755
C -0.584739 -1.693499 0.506322
H -0.951684 -2.545850 -0.066059
H -0.701638 -1.883616 1.583198
C -2.572087 -0.080929 -0.066964
C -3.079322 1.186596 -0.426407
C -3.501868 -1.111066 0.182259
C -4.449708 1.404838 -0.521931
H -2.386755 1.996371 -0.626552
C -4.872508 -0.884845 0.085794
H -3.147666 -2.100612 0.457478
C -5.358108 0.374309 -0.267088
H -4.812836 2.390438 -0.800052
H -5.563886 -1.698309 0.287751
H -6.426594 0.550620 -0.344604
43
INT-I-S$_0$-1xAcOMe_a
C 3.514999 -0.083807 -0.967924
C 2.784660 -0.286959 0.279465
C 3.450883 -0.663814 1.481101
C 4.807680 -0.795606 1.409756
C 5.559489 -0.566247 0.195376
C 4.947826 -0.216970 -0.966804
C 2.601890 0.182307 -1.952227
C 1.260461 0.265574 -1.331642
H 2.886478 -0.827143 2.391045
H 5.357970 -1.079683 2.301815
H 6.637924 -0.679691 0.231567
H 5.508290 -0.042237 -1.879552
H 2.808986 0.410988 -2.990962
H 0.972032 1.334190 -1.354167
N 1.496383 -0.109322 0.044887

C 0.284596 -0.585428 0.710586
O 0.288813 -0.748566 1.943736
C -0.635128 -0.763138 -0.325096
C -0.018401 -0.535655 -1.688576
H -0.664543 0.041823 -2.357523
H 0.262464 -1.467218 -2.192971
C -2.022413 -1.134449 -0.149889
C -2.640535 -1.134889 1.121959
C -2.821345 -1.472583 -1.264332
C -3.991908 -1.432221 1.256762
H -2.042698 -0.885916 1.992041
C -4.175067 -1.765685 -1.120185
H -2.376972 -1.501203 -2.255568
C -4.774340 -1.742932 0.140010
H -4.443272 -1.415818 2.245427
H -4.764020 -2.016701 -1.998383
H -5.829767 -1.971883 0.252652
C -1.164680 2.408795 0.194968
O -2.363833 2.177152 0.734760
O -0.984114 2.508316 -1.004483
C -0.108139 2.573590 1.248975
H -0.337793 3.460794 1.846801
H -0.100878 1.706260 1.915524
H 0.868094 2.696562 0.779000
C -3.452999 2.014683 -0.181064
H -3.242578 1.207105 -0.884742
H -4.319100 1.766836 0.431168
H -3.627403 2.945581 -0.726806
43

INT-I-S$_0$-1xAcOMe_b

C 3.582806 0.580798 -0.784343
C 2.913884 -0.306498 0.161712
C 3.642437 -1.245804 0.947061
C 4.998176 -1.250145 0.788842
C 5.689485 -0.357103 -0.114944
C 5.017729 0.540129 -0.883513
C 2.619705 1.295206 -1.444650
C 1.304748 0.958289 -0.855182
H 3.123321 -1.907387 1.629747
H 5.594865 -1.948598 1.367913
H 6.771527 -0.417908 -0.166988
H 5.532520 1.213035 -1.561732
H 2.774589 2.064102 -2.192295
H 0.976548 1.847732 -0.283524
N 1.611985 -0.093548 0.088040
C 0.445232 -0.914105 0.408260
O 0.504021 -1.718548 1.353457
C -0.513578 -0.544877 -0.539550
C 0.040493 0.415555 -1.570916
H -0.645971 1.233849 -1.810538
H 0.331397 -0.081067 -2.503601
C -1.880834 -1.016756 -0.559026
C -2.410008 -1.825852 0.474714
C -2.750790 -0.657800 -1.612121
C -3.744170 -2.216737 0.462802
H -1.755161 -2.136176 1.281873
C -4.086002 -1.052056 -1.614196
H -2.375032 -0.062663 -2.440106
C -4.598011 -1.828869 -0.574042
H -4.124195 -2.831832 1.274369
H -4.729573 -0.753124 -2.437631
H -5.639136 -2.136948 -0.576243
C -1.613868 1.883404 1.194933
O -2.924729 1.651925 1.078017
O -1.010195 2.634938 0.451366
C -1.020660 1.154948 2.363455
H -1.421699 1.580590 3.288696
H -1.290511 0.096403 2.327571
H 0.064447 1.261382 2.353068
C -3.590649 2.326470 0.006034
H -3.106533 2.102281 -0.946513
H -4.612404 1.949316 0.008263
H -3.582427 3.406142 0.176708
43

INT-I-S$_0$-1xAcOMe_c

C 3.791240 -0.366644 -0.894202
C 3.026613 0.113698 0.254435
C 3.665490 0.493568 1.470106
C 5.027449 0.413039 1.492586
C 5.811664 -0.023950 0.357105
C 5.228175 -0.402193 -0.809742
C 2.903496 -0.736968 -1.865487
C 1.535083 -0.403247 -1.398910
H 3.076592 0.828684 2.315132
H 5.558000 0.693534 2.397642
H 6.891732 -0.042312 0.458211
H 5.812588 -0.728613 -1.663906
H 3.135887 -1.110595 -2.855707
H 1.199942 0.451358 -2.012127
N 1.741499 0.064905 -0.045022
C 0.531682 -0.041712 0.772560
O 0.496999 0.514015 1.885634
C -0.330748 -0.836042 0.017243
C 0.328968 -1.366402 -1.238097
H -0.324060 -1.332477 -2.116056
H 0.698381 -2.392091 -1.125713
C -1.691112 -1.168601 0.381797
C -2.362327 -0.519303 1.443681
C -2.404656 -2.156449 -0.332051
C -3.677083 -0.843139 1.761145
H -1.833575 0.238577 2.012683
C -3.720784 -2.474940 -0.007278
H -1.918148 -2.687374 -1.146007
C -4.369898 -1.821366 1.041254
H -4.169694 -0.320919 2.577006
H -4.240718 -3.242078 -0.575133
H -5.395764 -2.069918 1.295634
C -2.450411 2.341355 -0.420594
O -2.409679 1.458798 -1.428173
O -3.482448 2.669057 0.126309
C -1.088187 2.847528 -0.047389
H -0.389024 2.767908 -0.881303
H -1.167364 3.882254 0.288860
H -0.712343 2.240003 0.784906
C -3.653665 0.826298 -1.746025
H -4.377072 1.564961 -2.100496
H -3.429062 0.109054 -2.534877
H -4.051712 0.313565 -0.866579
43

INT-I-S$_0$-1xAcOMe_e

C 2.119755 -1.546965 -0.056165
C 1.403167 -0.577536 0.767105
C 1.930784 -0.113104 2.007066
C 3.159937 -0.588579 2.360093
C 3.911876 -1.515792 1.541741
C 3.421194 -1.992375 0.367781
C 1.333481 -1.845522 -1.135298
C 0.130195 -0.980550 -1.087890
H 1.369050 0.589865 2.610464
H 3.606901 -0.257254 3.292674
H 4.890089 -1.829402 1.891371
H 3.982119 -2.688846 -0.247625
H 1.580202 -2.503097 -1.960647
H 0.278988 -0.236205 -1.891729
N 0.262533 -0.283480 0.170556
C -1.011324 0.247859 0.652023
O -1.013915 1.051715 1.601681
C -1.972800 -0.375873 -0.146222
C -1.362068 -1.398903 -1.083408
H -1.782378 -1.370318 -2.093004
H -1.450558 -2.423845 -0.704822
C -3.398482 -0.141833 -0.061339
C -3.944166 0.859155 0.777142
C -4.302845 -0.916094 -0.821853
C -5.317275 1.070167 0.836161
H -3.270130 1.460062 1.377679
C -5.675857 -0.693854 -0.759137
H -3.925981 -1.705917 -1.465688
C -6.197043 0.300582 0.069529
H -5.706918 1.847228 1.488639
H -6.343168 -1.307080 -1.358967
H -7.268058 0.471264 0.120434
C 2.437076 1.819337 -0.935035
O 3.688315 1.427516 -0.656735
O 1.863108 1.496338 -1.956091
C 1.857840 2.710245 0.125237
H 2.484908 2.733634 1.016300
H 1.782272 3.720351 -0.289251
H 0.848886 2.373353 0.380076
C 4.347064 0.660589 -1.671896
H 4.514226 1.278529 -2.557758
H 5.298563 0.353275 -1.240100
H 3.750137 -0.211519 -1.946597
40

INT-I-S$_0$-1xAcONa_a

C -3.480837 -0.180846 0.960918
C -2.772016 -0.351355 -0.301375
C -3.466111 -0.586788 -1.522106
C -4.829351 -0.622535 -1.449770
C -5.558126 -0.428580 -0.216894
C -4.918232 -0.208858 0.962691
C -2.545349 -0.044028 1.954389
C -1.209057 -0.019718 1.332073
H -2.918871 -0.720852 -2.447242
H -5.401125 -0.797722 -2.356207
H -6.642118 -0.459439 -0.251862
H -5.461760 -0.059194 1.890035
H -2.734163 0.130092 3.007124
H -0.814362 1.014968 1.418004
N -1.473515 -0.284772 -0.061852
C -0.282854 -0.733928 -0.774540
O -0.288389 -0.782485 -2.013112
C 0.639245 -1.028370 0.240948
C 0.023362 -0.919422 1.618035
H 0.683245 -0.435725 2.343678
H -0.309679 -1.880785 2.024220
C 2.053795 -1.192589 0.030938
C 2.650719 -1.004788 -1.244615
C 2.928220 -1.430807 1.122643
C 4.033626 -1.002897 -1.395718
H 2.006514 -0.825513 -2.098331
C 4.313902 -1.425506 0.958172
H 2.517082 -1.611962 2.110934
C 4.882585 -1.198337 -0.298163
H 4.456597 -0.833036 -2.381931
H 4.951780 -1.600726 1.820120
H 5.960303 -1.189193 -0.424143
C 1.015088 2.491076 -0.085591
O 1.987718 2.600446 -0.882051
O 1.116559 2.011949 1.084747
C -0.355238 2.925013 -0.584336
H -0.278690 3.886288 -1.098199
H -0.705863 2.189310 -1.317117
H -1.081419 2.996087 0.228776
Na 3.165437 1.211918 0.418386
40

INT-I-S$_0$-1xAcONa_b

C 2.452020 -1.743258 -0.140194
C 1.771715 -0.693685 0.617997
C 2.395753 -0.051486 1.727582
C 3.665086 -0.449801 2.025300
C 4.371174 -1.468469 1.275976
C 3.797946 -2.104096 0.221640
C 1.595329 -2.191465 -1.106006
C 0.373289 -1.353589 -1.063266
H 1.877373 0.725487 2.275409
H 4.184004 0.020339 2.855157
H 5.384879 -1.713085 1.575551
H 4.324638 -2.864507 -0.345916
H 1.796975 -2.941848 -1.861001
H 0.441601 -0.674721 -1.935495
N 0.573517 -0.519145 0.098079
C -0.668843 0.076993 0.542030
O -0.633064 1.062495 1.331133
C -1.669761 -0.659270 -0.071804

C -1.101114 -1.793960 -0.905520
H -1.587819 -1.899617 -1.878616
H -1.147510 -2.759547 -0.389457
C -3.095039 -0.413817 0.058051
C -3.604153 0.678199 0.797155
C -4.027740 -1.277356 -0.554468
C -4.974623 0.893657 0.899343
H -2.907853 1.345400 1.292556
C -5.397811 -1.052551 -0.449388
H -3.676992 -2.138393 -1.116122
C -5.883930 0.035185 0.276563
H -5.337724 1.741638 1.473920
H -6.089530 -1.735829 -0.934492
H -6.952372 0.209011 0.360014
C 2.104021 1.962830 -1.206711
O 2.328971 2.722527 -0.221530
O 0.956197 1.776554 -1.704992
C 3.293195 1.232624 -1.815392
H 3.924937 1.958734 -2.337816
H 3.900470 0.782782 -1.023107
H 2.973170 0.466994 -2.525238
Na 0.116473 2.805314 0.165263
40

INT-I-S_0-1xAcONa_c

C -4.278305 -0.415984 0.317125
C -3.150360 0.470609 0.039742
C -3.334027 1.731755 -0.596299
C -4.618429 2.078532 -0.899802
C -5.757413 1.237388 -0.600293
C -5.610058 0.024795 -0.006921
C -3.783688 -1.579583 0.835436
C -2.318692 -1.425595 1.012603
H -2.483226 2.364574 -0.815993
H -4.805936 3.031577 -1.385498
H -6.745176 1.600950 -0.863088
H -6.460016 -0.610184 0.220854
H -4.351431 -2.438671 1.172748
H -2.154422 -1.341632 2.101451
N -2.039711 -0.132769 0.424168
C -0.650870 -0.005349 0.025954
O -0.216127 1.138725 -0.275550
C -0.132021 -1.289701 0.065124
C -1.193449 -2.313451 0.428421
H -0.859482 -3.048182 1.165339
H -1.563101 -2.852669 -0.450550
C 1.241274 -1.660771 -0.241405
C 2.178428 -0.730394 -0.741257
C 1.685467 -2.987001 -0.054921
C 3.494168 -1.099233 -0.999316
H 1.868284 0.283704 -0.957135
C 3.003903 -3.351442 -0.315789
H 0.994738 -3.744680 0.302062
C 3.922563 -2.410204 -0.781777
H 4.182728 -0.347934 -1.377155
H 3.313730 -4.380276 -0.154042
H 4.950835 -2.695508 -0.982008
C 4.015952 2.051272 0.410697
O 3.359237 2.035341 1.492136
O 3.517196 2.359540 -0.709673
C 5.487031 1.669329 0.478485
H 6.007456 2.349607 1.159519
H 5.578785 0.660352 0.892298
H 5.956192 1.707975 -0.505855
Na 1.486974 2.377427 0.290702
43

TS-II-S_0-1xAcOMe_a

C 2.724700 -1.083973 -1.010238
C 1.915874 -1.451773 0.114977
C 2.439648 -2.191617 1.200744
C 3.767568 -2.539972 1.127276
C 4.595508 -2.170072 0.023927
C 4.097792 -1.446449 -1.030729
C 1.898999 -0.386526 -1.915721
C 0.632614 -0.236272 -1.287569
H 1.807090 -2.463955 2.037195
H 4.208895 -3.113935 1.936608
H 5.638168 -2.470896 0.030210
H 4.726406 -1.163614 -1.869603
H 2.192321 0.074600 -2.850844
H 1.090684 0.930203 -0.962363
N 0.680656 -0.952845 -0.089156
C -0.646170 -1.163395 0.493541
O -0.737370 -1.710466 1.606533
C -1.538928 -0.623970 -0.435804
C -0.833158 -0.075214 -1.661556
H -1.087157 0.974391 -1.863234
H -1.044100 -0.640116 -2.578203
C -2.966030 -0.501425 -0.244397
C -3.609440 -0.935822 0.940671
C -3.781800 0.072958 -1.246818
C -4.982968 -0.791376 1.103665
H -3.008280 -1.387594 1.722182
C -5.156223 0.213445 -1.072399
H -3.332716 0.407922 -2.178162
C -5.772639 -0.215513 0.103895
H -5.445086 -1.134893 2.025830
H -5.750492 0.659124 -1.866076
H -6.844591 -0.107707 0.238197
C 1.038029 2.659439 0.577736
O 1.420887 3.834847 1.013567
O 1.457641 2.218703 -0.500900
C 0.099823 1.934929 1.481503
H 0.621344 1.069240 1.903991
H -0.240616 2.583126 2.287244
H -0.751433 1.562700 0.903854
C 2.347574 4.575680 0.192231
H 1.900726 4.774257 -0.782930
H 2.526931 5.503499 0.730748
H 3.273409 4.010408 0.076236
43

TS-II-S_0-1xAcOMe_b

C 2.863794 -0.841901 -0.984448
C 2.081327 -1.421679 0.068883
C 2.658036 -2.259589 1.052413
C 4.007987 -2.494939 0.949345
C 4.808623 -1.917575 -0.083563
C 4.260832 -1.095189 -1.035505
C 1.990788 -0.088772 -1.795244
C 0.712664 -0.132425 -1.175549
H 2.045955 -2.687669 1.837204
H 4.489541 -3.138803 1.679273
H 5.870938 -2.138652 -0.105389
H 4.868906 -0.654714 -1.819861
H 2.251205 0.511797 -2.658284
H 1.052533 0.992419 -0.622284
N 0.812662 -0.999627 -0.086233
C -0.492210 -1.331223 0.490437
O -0.541989 -2.021047 1.522964
C -1.423272 -0.726230 -0.358925
C -0.761529 -0.036161 -1.536450
H -1.089187 1.003034 -1.663190
H -0.936999 -0.547942 -2.491908
C -2.854792 -0.731249 -0.167363
C -3.464102 -1.337889 0.958813
C -3.713272 -0.121996 -1.112291
C -4.844946 -1.322900 1.121111
H -2.829757 -1.818995 1.695304
C -5.094968 -0.111551 -0.938882
H -3.291584 0.343155 -1.999559
C -5.677313 -0.710139 0.179209
H -5.279552 -1.797524 1.997398
H -5.721683 0.365885 -1.687984
H -6.754712 -0.703488 0.312993
C 0.780108 3.146581 0.064753
O 0.782199 4.089084 0.974012
O 1.197297 2.011911 0.324985
C 0.219573 3.570579 -1.251905
H -0.872840 3.543013 -1.181024
H 0.521484 4.594306 -1.476780
H 0.546668 2.893684 -2.041657
C 1.252421 3.732630 2.293037
H 2.296034 3.419803 2.240889
H 1.149357 4.638638 2.885711
H 0.636495 2.929552 2.699845
40

TS-II-S_0-1xAcONa_a

C -2.732771 -1.132079 0.972562
C -1.892011 -1.537155 -0.130387
C -2.348346 -2.444898 -1.123172
C -3.632017 -2.903950 -0.992267
C -4.496314 -2.493828 0.080247
C -4.073417 -1.623237 1.044029
C -1.979273 -0.299695 1.791079
C -0.716332 -0.061477 1.127832
H -1.694472 -2.744517 -1.933247
H -4.021581 -3.602271 -1.727036
H -5.505514 -2.891653 0.112191
H -4.725446 -1.313078 1.854830
H -2.316533 0.214288 2.683427
H -1.000769 1.031010 0.694798
N -0.712318 -0.930169 0.006286
C 0.630923 -1.192854 -0.504039
O 0.764430 -1.830546 -1.563987
C 1.490275 -0.604269 0.426860
C 0.745267 0.016598 1.591359
H 1.050122 1.053106 1.783006
H 0.867677 -0.541453 2.527757
C 2.928006 -0.532026 0.296520
C 3.608632 -1.039289 -0.837487
C 3.715901 0.058927 1.311206
C 4.992713 -0.951801 -0.939298
H 3.027741 -1.503134 -1.627091
C 5.101087 0.143560 1.197728
H 3.235948 0.449674 2.204460
C 5.755019 -0.360349 0.072481
H 5.484611 -1.351109 -1.822715
H 5.673978 0.604104 1.998544
H 6.835433 -0.296833 -0.014065
C -0.855520 2.867663 -0.935645
O -1.134997 4.006087 -1.369687
O -1.345766 2.437436 0.177139
C 0.061695 1.952054 -1.707056
H -0.489319 1.055234 -2.009547
H 0.451755 2.454772 -2.591468
H 0.887630 1.624564 -1.067481
Na -2.442316 4.381208 0.407874
40

TS-II-S_0-1xAcONa_b

C 2.747348 -1.372541 -0.411134
C 1.983379 -0.801167 0.674409
C 2.601141 -0.364986 1.875859
C 3.959639 -0.520037 1.958075
C 4.741302 -1.084700 0.893511
C 4.163023 -1.500778 -0.272489
C 1.867471 -1.653537 -1.453363
C 0.576073 -1.149353 -1.066549
H 2.011184 0.071716 2.672749
H 4.474365 -0.198640 2.858603
H 5.814381 -1.171222 1.029428
H 4.753091 -1.920762 -1.081253
H 2.120819 -2.040703 -2.432776
H 0.660940 -0.036792 -1.560952
N 0.707139 -0.759385 0.289335
C -0.564990 -0.354950 0.838991
O -0.584658 0.386388 1.865103
C -1.525756 -0.827029 -0.042653
C -0.895802 -1.538021 -1.229271
H -1.299080 -1.190048 -2.185289
H -1.007444 -2.628122 -1.189519
C -2.951058 -0.563393 0.054849
C -3.509757 0.173109 1.125761
C -3.837572 -1.043721 -0.933344
C -4.877932 0.423448 1.184732
H -2.858396 0.528669 1.916602
C -5.204150 -0.788253 -0.865645

H	-3.453008	-1.631408	-1.761952
C	-5.737615	-0.048811	0.191029
H	-5.277345	0.990754	2.021304
H	-5.857312	-1.173705	-1.643850
H	-6.803813	0.148877	0.243303
C	1.519936	2.158249	-1.105992
O	1.458283	3.021581	-0.205189
O	0.487955	1.484625	-1.477193
C	2.847109	1.896193	-1.787452
H	3.156713	2.805941	-2.310740
H	3.607161	1.676474	-1.031859
H	2.782771	1.074497	-2.502612
Na	-0.552580	2.239189	0.517795
